# Synthesis and reactivity of azole-based iodazinium salts

**DOI:** 10.3762/bjoc.19.27

**Published:** 2023-03-16

**Authors:** Thomas J Kuczmera, Annalena Dietz, Andreas Boelke, Boris J Nachtsheim

**Affiliations:** 1 Institute for Organic and Analytical Chemistry, University of Bremen, 28359 Bremen, Germany

**Keywords:** building block, heterocycles, hypervalent compounds, iodonium salts, one-pot synthesis

## Abstract

A systematic investigation of imidazo- and pyrazoloiodazinium salts is presented. Besides a robust synthetic protocol that allowed us to synthesize these novel cyclic iodonium salts in their mono- and dicationic forms, we gained in-depth structural information through single-crystal analysis and demonstrated the ring opening of the heterocycle-bridged iodonium species. For an exclusive set of dicationic imidazoiodaziniums, we show highly delicate post-oxidation functionalizations retaining the hypervalent iodine center.

## Introduction

**Figure 1:**
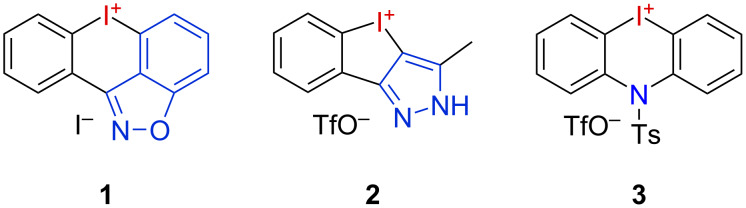
Nitrogen-containing iodolium and iodonium salts.

The chemistry of hypervalent iodine compounds, in particular aryl-λ^3^-iodanes, is highly versatile, and a wide range of applications is meanwhile established in organic synthesis [1–5]. They can be applied as mild oxidants [6–8], in phenol dearomatizations [9] or in α-oxygenation reactions [10]. In a complemental reactivity, diaryliodonium salts are potent electrophilic aryl donors [11–16]. Their cyclic derivatives have a proven utility as precursors for the synthesis of hetero- and carbocycles [17–21], and their pronounced σ-holes [22] render them efficient halogen-bond donors (XB donors in XB catalysis) [23]. Despite their great potential in organic synthesis and catalysis, their structural variation is still limited. In particular, heteroarene-bridged cyclic iodonium salts are rare. Examples include the benzisoxazole-containing iodonium salt **1** described by Lisichkina and Tolstaya (Figure 1) [24–25]. Our group is interested in the chemistry of hypervalent iodine species in all their variety, particularly those containing *N-*heterocycles either as tethered stabilizing ligands or as an inclusive part of a cyclic iodonium salt [26–31]. We prepared five-membered, *N*-heterocycle-containing iodoliums **2** and investigated their reactivity and utility in XB catalysis. We also established one-pot methods for generating six-membered carbon-, oxygen-, and nitrogen-bridged iodonium salts, such as the iodazinium triflate **3** [32–33]. Based on these promising findings, we further wanted to elaborate this chemistry and herein we present the first synthesis and application of more sophisticated imidazo- and pyrazoloiodazinium salts.

## Results and Discussion

Initially, we focused on developing a mild oxidation procedure starting from iodoarene precursors. Previous studies on five-membered heteroaromatic iodonium salts revealed *m*-chloroperoxybenzoic acid (*m*CPBA) as the oxidant of choice in the presence of triflic acid (TfOH) [27,29]. Based on these promising results, the conditions were optimized using *o*-benzimidazole-substituted iodoarenes **4aa** and **4ah** (Table 1).

**Table 1:** Optimization of reaction conditions for the synthesis of azoiodazinium salts **5aa** and **5ah**.^a^

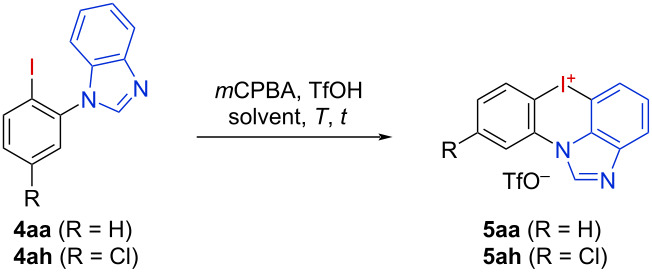							

Entry	R	*m*CPBA (equiv)	TfOH (equiv)	*T* (°C)	*t* (d)	Solvent	Yield (%)

1	H	1.1	3.0	50	3	MeCN	0
2	H	1.1	3.0	50	3	DCE	23
3	H	1.1	2.5	50	3	DCE	69
4	H	1.1	2.0	50	3	DCE	19
**5**	**H**	**1.1**	**2.5**	**40**	**3**	**DCM**	**69**
6	H	1.5	2.5	40	3	DCM	53
7	Cl	1.1	2.5	40	3	DCM	9^b^
**8**	**Cl**	**1.3**	**5.0**	**65**	**14**	**DCM**	**52**

^a^Iodoarene **4aa** or **4ah** (200 µmol) and *m*CPBA were dissolved in the given solvent (1 mL) in a screw cap vial, TfOH was added, and the reaction mixture was stirred under the corresponding conditions. For full table, see Supporting Information File 1. ^b^Incomplete conversion, product not clean.

While running the reaction in MeCN as solvent resulted in no product formation, the reaction of **4aa** in DCE at 50 °C gave the product **5aa** in 23% yield (Table 1, entries 1 and 2). A larger amount of TfOH turned out to increase the solubility of the product and therefore impeded the purification process. However, an excess of acid is required for the electrophilic aromatic substitution to take place. With 2.5 equivalents of TfOH as the optimum amount of acid the product **5aa** was obtained in a yield of 69% (Table 1, entry 3). Similar results were observed with DCM at 40 °C (Table 1, entry 5). A higher amount of *m*CPBA did not lead to a better yield due to more washing required to remove the *m*-chlorobenzoic acid (Table 1, entry 6). When we employed the chlorinated, electron-deficient iodoarene **4ah**, the yield of the product **5ah** dropped significantly (Table 1, entry 7). Combining an electron-deficient heterocycle and an iodoarene with electron-withdrawing substituents results in a significantly decreased reactivity. Thus, for those substrates, harsher reaction conditions were required. A slight adaption of the original conditions to elevated temperatures (65 °C) and prolonged reaction times of 14 d finally resulted in the formation of product **5ah** in 52% yield using DCM as the solvent (Table 1, entry 8).

**Figure 2:**
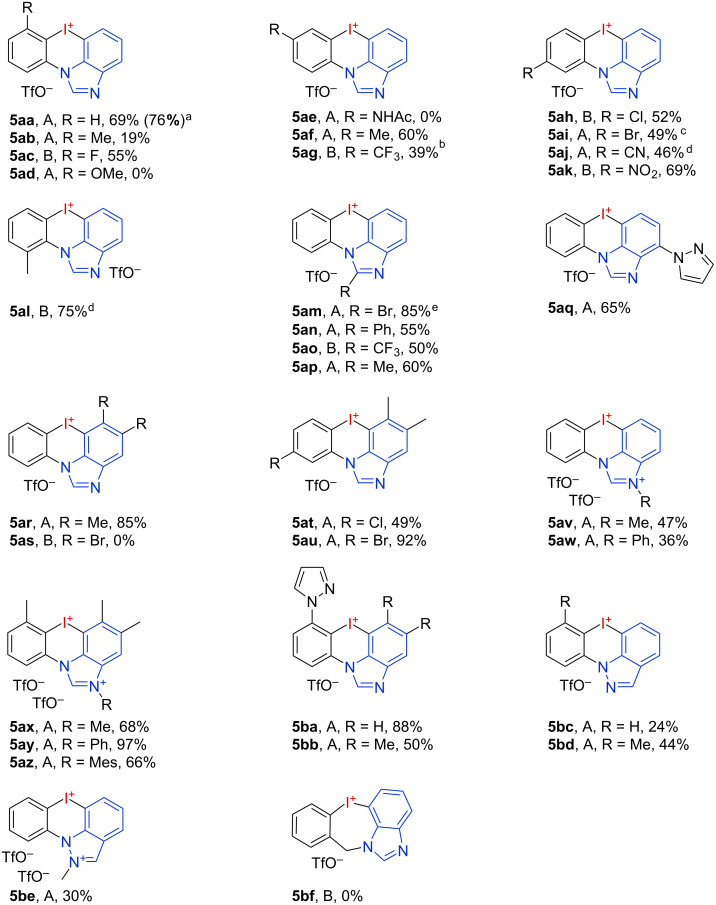
Synthesis of a set of azoiodazinium salts **5**. Method A: Iodoarene **4** (200 µmol) and *m*CPBA (1.1 equiv) were dissolved/suspended in DCM (1 mL), TfOH (2.5 equiv) was added, and the reaction mixture was stirred for 72 h at 40 °C. Method B: Iodoarene **4** (200 µmol) and *m*CPBA (1.3 equiv) were dissolved/suspended in DCM (1 mL), TfOH (5.0 equiv) was added, and the reaction mixture was stirred for 14 d at 65 °C. ^a^6.00 mmol scale, *T* = 50 °C, ^b^*T* = 40 °C, ^c^*T* = 80 °C, *t* = 6 d, ^d^*t* = 7 d, ^e^0.3 equiv of DCM were included in the product.

Next, various substituted iodoarenes **4** were oxidized and cyclized using the optimized conditions to generate a diverse set of azoiodazinium salts **5** (Figure 2). The *ortho*-methylated salt **5ab** was obtained in a low yield of 19%, and the fluorinated derivative **5ac** could be obtained in 55%. Unfortunately, the MeO-substituted derivative **5ad** did not form. Except for the acetamide **5ae**, which could not be obtained due to decomposition, other *meta-* and *para*-substituted derivatives **5af**–**ak**, among them derivatives with strong electron-withdrawing functionalities, could be synthesized in 39–69% yield. The electron-rich salt **5al** was obtained in 75% yield using modified reaction conditions B. The harsher conditions were probably required due to a sterically hindered rotation of the benzimidazole moiety in the plane of the iodophenyl, which could also be observed in two rotamers of the starting iodoarene **4al** (see Supporting Information File 1).

Next, we investigated substrates **4** having various substituents in the benzimidazole motif. Starting from 2-bromo-derivative **4am**, the moisture-sensitive, brominated salt **5am** was obtained in an excellent yield of 85%. Also, the C2-alkyl- and phenyl-substituted benzimidazoles gave the expected products **5an–ap** in 50–60% yield. The pyrazole-substituted salt **5aq** was obtained with 65% yield. Using an electron-rich 5,6-dimethylbenzimidazole substrate yielded dimethylated product **5ar** in 85% yield. Unfortunately, even under harsher reaction conditions, the corresponding electron-deficient dibrominated salt **5as** could not be obtained. This further demonstrated the crucial influence of the electronic properties on the reactivity of those substrates. Especially, electron deficiency is particularly counterproductive for the final cyclization step. To prove the influence of the electron-rich dimethylated benzimidazole, this moiety combined with the chlorinated and brominated iodoarene gave the corresponding salts **5at** and **5au** in good yields under milder reaction conditions, in particular in direct comparison to the unsubstituted analogs **5ah**–**aj**. *N*-Substituted iodoarenes were then used to create dicationic iodonium salts. The *N*-Me and *N*-Ph-iodonium-benzimidazolium salts **5av** and **5aw** were obtained in 47% and 36% yield, respectively. The introduction of additional *ortho*-methyl groups resulted in the formation of the σ-hole-protected *N*-substituted salts **5ax–az** in up to 97% yield. Next, the iodonium center was stabilized through an additional *N*-coordination via *ortho*-pyrazole substitution, giving the iodonium salts **5ba** and **5bb** in 88% and 50% yield. When replacing imidazoles by indazoles the oxidation was not as efficient giving the products **5bc** and **5bd** with only 24% and 44% yields. In the latter case, the initially generated hydroxy-iodonium salt is stabilized via the indazole nitrogen [26] and the steric hindrance by the methyl group is likely destabilizing this intermediate by an out-of-plane distortion [28,34] and hence accelerating the cyclization. The dicationic indazole salt **5be** was isolated in 30% yield and the benzyl-bridged, seven-membered salt **5bf** could not be obtained under our optimized reaction conditions.

**Figure 3:**
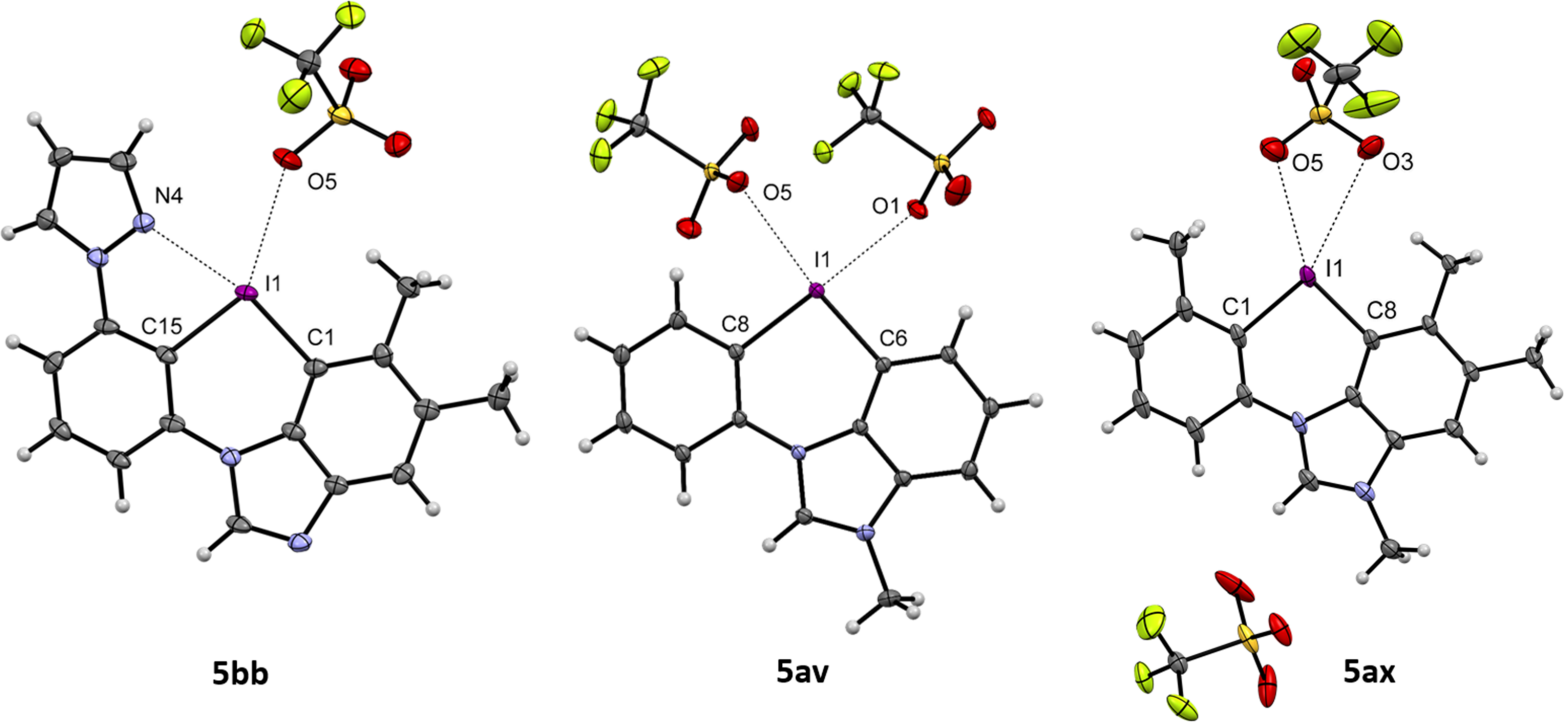
Single crystal structures (ORTEP drawing with 50% probability) of the pyrazole-coordinated salt **5bb** (dimer, a second structure was omitted for clarity. For full structure, see Supporting Information File 1; CCDC 2216124) and the two *N*-methylated, dicationic salts **5av** (CCDC 2216134) and **5ax** (CCDC 2216127). Selected bond lengths and angles: For **5bb**: N4–I1: 2.540 Å, I1–O5: 2.905 Å, N4–I1–C1: 185.74°, C15–I1–O5: 145.50°; for **5av**: I1–O1: 2.705 Å, I1–O5: 2.898 Å, C8–I1–O1: 173.80°, C6–I1–O5: 167.75°, C6–I1–C8: 94.05°; for **5ax**: I1–O3: 3.354 Å, I1–O5, 3.078 Å, C1–I1–O3: 154.71°, C8–I1–O5: 148.91°, C1–I1–C8: 94.53°.

Single crystal structures of selected salts were obtained to gain a better understanding of the bonding situation and the coordination states in these novel azoiodazinium salts (Figure 3). An N4–I1 distance of 2.540 Å with a typical T-shape structure (N4–I1–C1 angle 185.74°) implies a significant interaction between the *N*-heterocycle and the iodine atom for the *ortho*-pyrazole-substituted derivative **5bb** [35]. However, the presence of an *ortho*-methyl group significantly disturbs the triflate coordination to the other iodine σ-hole, which results in a C15–I1–O5 angle of 145.50°. In contrast to other six-membered iodonium salts, this molecule is nearly in plane with an I1–C1–C15–N4 dihedral angle of 2.03° [30,32]. For the dicationic salt **5av**, we observed a coordination of the triflates along the C–I axis with distances of 2.705 Å (I1–O1) and 2.898 Å (I1–O5). For the *ortho*-methyl-substituted analogue **5ax**, no halogen bonding to the triflates was observed, indicating an effective steric protection of the σ-holes [36]. Instead, there were only two weak interactions with one of the triflates (I1–O3: 3.354 Å, I1–O5, 3.078 Å).

**Scheme 1:**
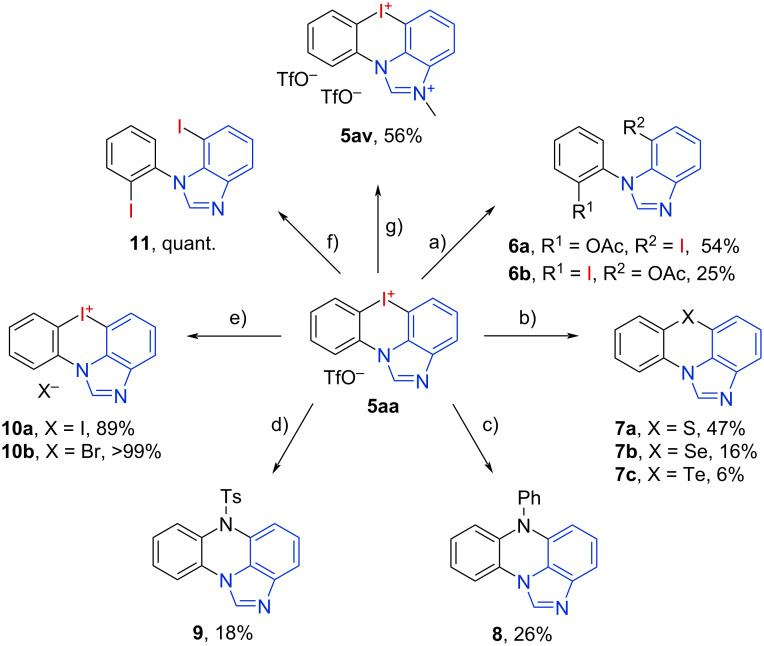
Derivatizations of the iodonium salt **5aa**. a) Ac_2_O, CuSO_4_·5H_2_O, NaOAc, AcOH, 120 °C, 5 h; b) S_8_/Se/Te, Cs_2_CO_3_, DMSO, rt–100 °C, 2.5–24 h; c) I: PhNH_2_, Cu(OAc)_2_·H_2_O, Na_2_CO_3_, iPrOH, 40 °C, 17 h, II: CuI, Cs_2_CO_3_, DMF, 120 °C, 40 h; d) TsNH_2_, (CH_2_OH)_2_, iPrOH, Na_2_CO_3_, 100 °C, 24 h, e) KX, H_2_O, EtOH, reflux; f) CuI, DMEDA, 1,4-dioxane, TBAI, rt, 24 h; g) MeOTf, DMF, 40 °C, 24 h.

We finally investigated the further reactivity of the synthesized azoiodazinium salts to elaborate their potential as synthetic building blocks (Scheme 1). Treatment of **5aa** with Ac_2_O led to a non-selective ring opening at both C–I bonds giving the iodinated *N*-arylbenzimidazoles **6a** and **6b** as a mixture with 54% and 25% yield [37]. A ring opening/closing cascade reaction with elemental sulfur resulted in the formation of the imidazo[4,5,1-*kl*]phenothiazine (**7a**) in 47% yield.

The corresponding phenoselenazine **7b** and the phenotellurazine **7c** were isolated in lower yields of 16% and 6%, likely due to undesired oxidations of selenium and tellurium [38]. Substitutions with nitrogen nucleophiles were performed, giving the *N-*phenylphenazine **8** in 26% yield [39–40] and the *N*-tosyl derivative **9** in 18% yield [41]. Anion exchange reactions to iodide and bromide were performed giving the salts **10a** and **10b** in excellent yields [27]. A copper-catalyzed iodination gave the diiodinated product **11** in quantitative yield [42]. Finally, *N*-methylation of **5aa** was performed, to yield the dicationic salt **5av** in 56% yield without decomposition of the iodonium center [43]. In this reaction, however, no complete conversion could be achieved, even by adding excess MeOTf.

**Scheme 2:**
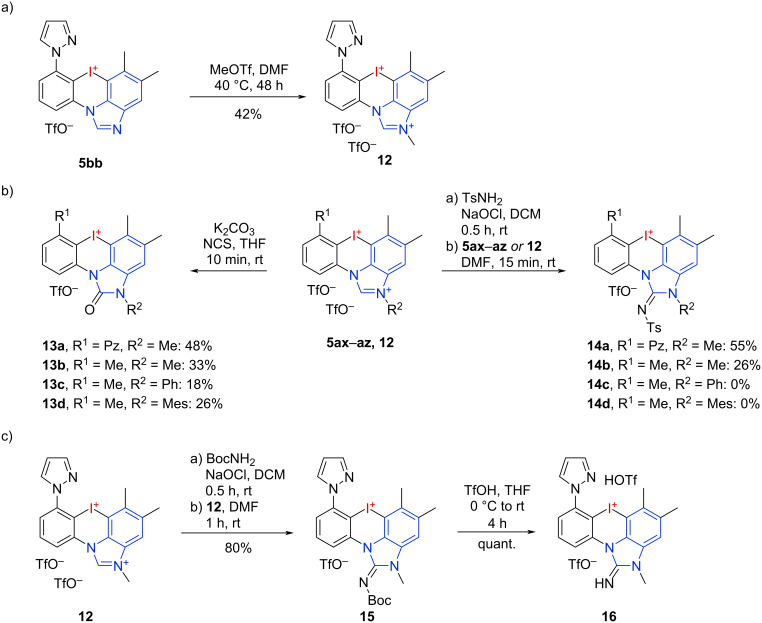
Post-functionalization of mono- and dicationic iodonium salts under preservation of the hypervalent iodine center.

Inspired by the latter results, we were interested to investigate other post-oxidation functionalizations on the benzimidazole ring while keeping the highly reactive hypervalent iodine center intact. Treatment of the *ortho*-pyrazole-substituted salt **5bb** with MeOTf resulted in a selective benzimidazole *N*-methylation. A reaction on the pyrazole nitrogen is impeded due to its coordination with the iodane’s σ-hole (Scheme 2a). Besides nitrogen-substitution, the benzimidazole C-2 position of the dicationic salts is a reactive site for oxidative transformations [44–45]. The reaction of the iodine-protected benzimidazolium salts **5ax**–**az** and **12** with different oxygen sources revealed K_2_CO_3_ and NCS as the optimal system to form the benzimidazole-2-ones **13a**–**d** in 18–48% yield, with the best result obtained when using the stabilized salt **12** (Scheme 2b) [44]. Here, no counter-ion exchange to chloride was observed. The favored counter ion is determined by the p*K*_a_ value of the corresponding acids but not by halogen bonding due to the steric hindrance at the iodines’ σ-holes. The reaction of TsNH_2_ in combination with NaOCl as an oxidant was investigated next. Under these conditions, the *N*-Me salts **5ax** and **12** gave the desired products **14a** and **14b** in 55% and 26% yield, respectively. The corresponding *N*-Ph- and *N*-Mes-derivatives **5ay** and **5az** failed to give products **14c** and **14d** and only underwent undesired ring openings.

Treating **12** with BocNH_2_ resulted in the formation of protected guanidine **15** in 80% yield (Scheme 2c), which would not be possible to obtain via an oxidative cyclization of the corresponding iodine(I) species due to a carbamate cleavage with acid. The other dicationic salts underwent ring openings in this reaction. This reactivity demonstrates the highly stabilizing effect of *N*-heterocycles on hypervalent iodine species. Furthermore, the formed 2-aminobenzimidazoles reveal new access to potential bioactive compounds [46–47]. Even the formation of the free guanidine **16** via cleavage of the Boc-group was possible in quantitative yield.

## Conclusion

In this work, we prepared azoiodaziniums as a new class of six-membered heterocyclic iodonium salts with a wide range of substituents. Derivatizations of the reactive iodonium center allow for the formation of new heterocyclic compounds based on azol-based iodazinium as reactive intermediates. Most interestingly, functionalization of the heteroarene salts was achieved without an undesired attack of the delicate C–I bond at the hypervalent iodine center.

## Experimental

General procedure for the synthesis of azoiodazinium salts (method A): To a stirred solution of the corresponding (2-iodophenyl)-1*H*-benzo[*d*]imidazole or -indazole (200 µmol, 1.0 equiv) and *m*CPBA (85%, 44.8 mg, 220 µmol, 1.1 equiv) in DCM (1 mL) was added TfOH (44.2 µL, 500 µmol, 2.5 equiv) and the resulting solution was stirred for 72 h at 40 °C. The solvent was removed under reduced pressure. The residue was suspended in Et_2_O (1 mL) or another solvent if necessary, stored at 4 °C for 30 min, filtered, washed with Et_2_O (3 × 1 mL) and dried in vacuo.

## Supporting Information

File 1 Experimental procedures, characterization data and copies of spectra.
