# Supplementary material for: Synthesis and reactivity of azole-based iodazinium salts
Source: Beilstein J Org Chem. 2023 Mar 16;19:317–24. doi: 10.3762/bjoc.19.27 (PMC10028571; doi:10.3762/bjoc.19.27)
Supplement: File 1 — Experimental procedures, characterization data and copies of spectra. [file Beilstein_J_Org_Chem-19-317-s001.pdf]

BEILSTEIN JOURNAL OF ORGANIC CHEMISTRY

Supporting Information

for

Synthesis and reactivity of azole-based iodazinium salts

Thomas J. Kuczmera, Annalena Dietz, Andreas Boelke and Boris J. Nachtsheim

Beilstein J. Org. Chem. **2023,** *19,* 317–324. doi:10.3762/bjoc.19.27

Experimental procedures, characterization data and copies of spectra

License and Terms: This is a supporting information file under the terms of the Creative Commons Attribution License (https://creativecommons.org/licenses/by/4.0). Please note that the reuse, redistribution and reproduction in particular requires that the author(s) and source are credited and that individual graphics may be subject to special legal provisions.
The license is subject to the *Beilstein Journal of Organic Chemistry* terms and conditions: (https://www.beilstein-journals.org/bjoc/terms)

Table of contents

1 General information S2

2 Synthesis of 1-(2-iodophenyl)-1*H*-benzo[*d*]imidazoles S3

3 Oxidative cyclization reactions S23

4 Reactions of iodonium salt **5aa** S35

5 Post-functionalizations of iodonium salts under preservation of the hypervalent iodine S41

6 References S45

7 NMR spectra S47

8 Crystal structures S136

1 General information

Unless otherwise stated, all reactions with moisture- or oxygen-sensitive reagents were performed using standard Schlenk techniques under a nitrogen or argon atmosphere. Reagents were used as received from their commercial supplier (abcr, Acros Organics, Alfa Aesar, Apollo Scientific, Carbolution Chemicals, Sigma Aldrich, TCI, fluorochem, BLD pharm). *m*CPBA was dried under vacuum (10^{-3} mbar) for 2 h before use. Anhydrous dichloromethane (DCM), acetonitrile (MeCN), tetrahydrofuran (THF) and toluene were obtained from an *inert* PS-MD-6 solvent purification system. All other solvents were dried using standard methods.[1] Unless otherwise stated, all yields refer to isolated yields of compounds estimated to be >95% pure as determined by ^{1}H NMR spectroscopy.

Thin layer chromatography was performed on fluorescence indicator marked precoated silica gel 60 plates (Macherey-Nagel, ALUGRAM Xtra SIL G/UV_{254}) and visualized by UV light (254 nm/366 nm). Flash column chromatography was performed on silica gel (0.040–0.063 mm) with the solvents given in the procedures.

^{1}H-, ^{13}C-, ^{19}F- and ^{77}Se NMR spectra were recorded on Bruker Avance *Neo* 600-spectrometers. Chemical shifts for ^{1}H NMR spectra were reported as δ (parts per million) relative to the residual proton signal in $CDCl_3$ at 7.26 ppm (s), d_4-MeOH at 3.31 ppm (quin.), d_6-DMSO at 2.50 ppm (quin) or d_3-MeCN at 1.94 ppm (quin). Chemical shifts for ^{13}C NMR spectra were reported as δ (parts per million) relative to the signal of $CDCl_3$ at 77.0 ppm (t), d_4-MeOH at 49.0 ppm (sept.), d_6-DMSO at 39.5 ppm (sept.) or d_3-MeCN at 118.26 ppm (s). ^{19}F NMR spectra were reported as δ (parts per million) relative to $CFCl_3$ at 0.00 ppm as the external standard. The following abbreviations were used to describe splitting patterns: br = broad, s = singlet, d = doublet, t = triplet, q = quartet, quin = quintet, sext. = sextet, sept = septet, m = multiplet. Coupling constants *J* are given in hertz.

High resolution (HR) EI mass spectra were recorded on a double focussing mass spectrometer ThemoQuest MAT 95 XL from *Finnigan MAT*. HR-ESI mass spectra were recorded on a Bruker impact II. APCI mass spectra were recorded on an Advion Expression CMS^{L} via ASAP probe or direct inlet. EI mass spectra were obtained from an Agilent 7890B GC System with an Agilent 5977A MSD mass spectrometer. All signals were reported with the quotient from mass to charge *m/z*. Many iodonium salts undergo reductive ring-opening reactions during HRMS measurement.

IR spectra were recorded on a Nicolet Thermo iS10 scientific spectrometer with a diamond ATR unit. The absorption bands were reported in cm^{-1}.

Melting points were determined on a Büchi M-5600 Melting Point apparatus with a heating rate of 5 °C/min. The melting points were reported in °C. Most of the hypervalent iodine compounds underwent changes in appearance (e.g. softening) before final melting/decomposition.

Single crystals were grown from MeCN solution. A suitable crystal was selected and measured on a Bruker D8 Venture diffractometer. The crystal was kept at 100 K during data collection. Using Olex2,[2] the structure was solved with the ShelXT[3] structure solution program using Intrinsic Phasing and refined with the XL[4] refinement package using Least Squares minimization. The ORTEP drawing was made using the program Mercury from the CCDC.

2 Synthesis of 1-(2-iodophenyl)-1*H*-benzo[*d*]imidazoles

2-Methyl-1*H*-benzo[*d*]imidazole (S2a)

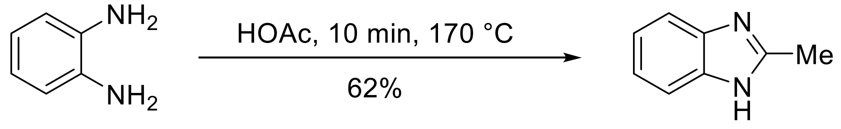

Following a literature procedure[5] *o*-phenylenediamine (**S1**, 54.0 mg, 500 µmol) was dissolved in HOAc (2 mL) and heated in a microwave (0–600 W) for 10 min at 170 °C. H_2O (2 mL) was added and extracted with EtOAc (3 × 2 mL). The combined organic layers were dried over Na_2SO_4, filtered and the solvent was removed under reduced pressure. The residue was purified via flash column chromatography on silica (Cy 1:1 EtOAc), so that 2-methyl-1*H*-benzo[*d*]imidazole (**S2**, 40.9 mg, 310 µmol, 62%) was obtained as a yellow solid.

^{1}H NMR (601 MHz, $CDCl_3$) δ (ppm) 8.75 (s, 1H), 7.56 (dd, *J* = 6.0, 3.2 Hz, 2H), 7.22 (dd, *J* = 6.0, 3.1 Hz, 2H), 2.65 (s, 3H). **^{13}C NMR** (151 MHz, $CDCl_3$) δ (ppm) 151.4, 138.6, 122.4, 114.6, 15.0. **MS** (ESI) *m/z* 133.2 $[M+H]^+$. **IR** (ATR) v (cm^{-1}) 3063, 2984, 2721, 2677, 1556, 1439, 1386, 1270, 833, 729. **M$_P$** (°C) 180. Analytical data is in accordance to those reported in the literature.[5]

2-(Trifluoromethyl)-1*H*-benzo[*d*]imidazol (S2b)

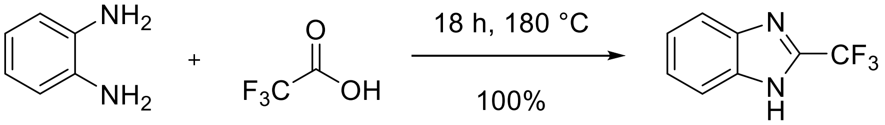

Following a modified literature procedure,[5] a pressure vial was filled with *o*-phenylenediamine (**S1**, 1.62 g, 15.0 mmol) 2,2,2-trifluoroacetic acid (**S3**, 30 mL) and stirred for 11 h at 180 °C. The solvent was removed under reduced pressure, and the residue was filtered through silica with EtOAc. The solution was washed with sat. aq. $NaHCO_3$ solution (30 mL), brine (30 mL), dried over Na_2SO_4, filtered and the solvent was removed under reduced pressure to obtain 2-(trifluoromethyl)-1*H*-benzo[*d*]imidazole (**S4**, 2.79 g, 15.0 mmol, 100%) as a colorless solid.

^{1}H NMR (601 MHz, $CDCl_3$) δ (ppm) 7.73 (dd, *J* = 6.1, 3.3 Hz, 2H), 7.42 (dd, *J* = 6.2, 3.1 Hz, 2H). **^{13}C NMR** (151 MHz, $CDCl_3$) δ (ppm) 140.7 (q, *J* = 40.5 Hz), 137.4, 125.1, 118.9 (q, *J* = 270.8 Hz), 116.7 ppm. **^{19}F NMR** (565 MHz, $CDCl_3$) δ (ppm) -64.2. **MS** (ESI) *m/z* 187.1 $[M+H]^+$. **IR** (ATR) v (cm^{-1}) 2968, 2873, 2756, 2655, 1550, 1499, 1462, 1400, 1317, 1286, 1129, 980, 739. **M$_P$** (°C) 209. Analytical data is in accordance with those reported in the literature.[6]

2-Phenyl-1*H*-benzo[*d*]imidazole (S2c)

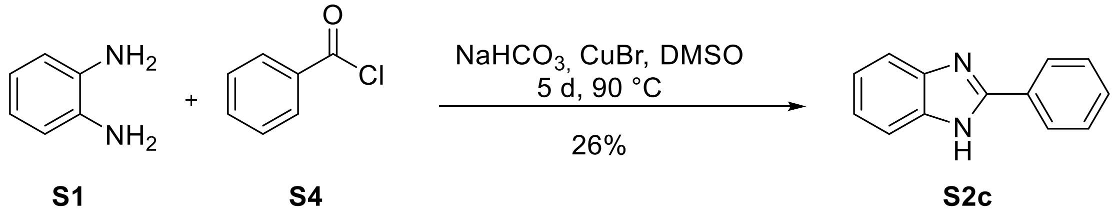

Following a literature procedure,[7] *o*-phenylenediamine (**S1**, 1.08 g, 10.0 mmol), benzyl chloride (**S4**, 1.27 g, 10.0 mmol), $NaHCO_3$ (1.00 g, 12.0 mmol) and CuBr (71.1 mg, 500 µmol) in DMSO (40 mL) were stirred for 5 d at 90 °C. H_2O (50 mL) and brine (30 mL) were added, and the mixture was extracted with EtOAc (3 × 50 mL). The combined organic layers were washed with brine (30 mL), dried over Na_2SO_4, filtered, and the solvent was removed under reduced pressure. The residue was purified via column chromatography on silica (DCM 10:1 MeOH) and recrystallization from DCM, so that 2-phenyl-1*H*-benzo[*d*]imidazole (**S2c**, 513 mg, 2.64 mmol, 26%) was obtained as colorless crystals.

^{1}H NMR (601 MHz, DMSO-d_6) δ (ppm) 12.90 (s, 1H), 8.26 – 8.09 (m, 2H), 7.57 (s, 2H), 7.55 (t, J = 7.5 Hz, 2H), 7.49 (t, J = 7.3 Hz, 1H), 7.20 (dd, J = 6.2, 3.1 Hz, 2H). **^{13}C NMR** (151 MHz, $CDCl_3$) δ (ppm) 151.2, 130.2, 129.8, 129.0, 126.4, 122.2, 118.8, 111.3. **MS** (ESI) *m/z* 195.1 $[M+H]^+$. **IR** (ATR) ν (cm^{-1}) 2625, 1462, 1443, 1409, 1276, 1119, 970, 737, 701. **M$_P$** (°C) 287. Analytical data is in accordance with those reported in the literature.[8]

5,6-Dimethyl-1*H*-benzo[*d*]imidazole (S2d)

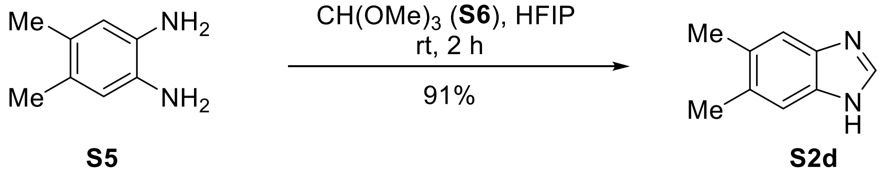

Following a literature procedure,[9] 4,5-dimethylbenzene-1,2-diamine (**S5**, 1.36 g, 10.0 mmol) and $CH(OMe)_3$ (**S6**, 1.12 mL, 10.2 mmol) were stirred in 1,1,1,3,3,3-hexafluoroisopropanol (10 mL) at room temperature for 2 h. The solvent was removed under reduced pressure and purified via column chromatography on silica (DCM 25:1 MeOH -> 10:1), so that 5,6-dimethyl-1*H*-benzo[*d*]imidazole (**S2d**, 1.34 g, 9.14 mmol, 91%) was obtained as off-white solid.

^{1}H NMR (400 MHz, $CDCl_3$) δ (ppm) 8.77 (s, 1H), 8.01 (s, 1H), 7.44 (s, 2H), 2.37 (s, 6H). **^{13}C NMR** (100 MHz, $CDCl_3$) δ (ppm) 140.3, 136.3, 132.0, 115.6, 20.4. **MS** (ESI) *m/z* 147.1 $[M+H]^+$. **IR** (ATR) ν (cm^{-1}) 2963, 2706, 2632, 2552, 1470, 1446, 1394, 1269, 1159, 999, 860, 843. **M$_P$** (°C) 199-200. Analytical data is in accordance with those reported in the literature.[10]

General procedure for synthesis of substituted 1-(2-iodophenyl)-1*H*-benzo[*d*]imidazoles 4 from *o*-fluoroiodobenzenes (GP1)

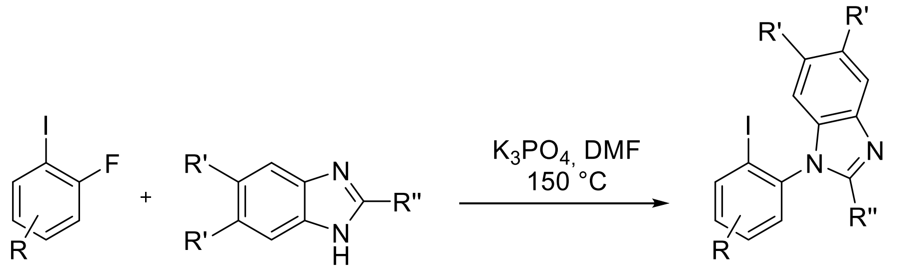

S10 **S2** **4**

Following a modified literature procedure,[11] the corresponding *o*-fluoroiodobenzene (**S10**), the corresponding 1*H*-benzo[*d*]imidazole (**S2**), and K_3PO_4 were stirred in DMF at 150 °C for the indicated time in a pressure vial under conventional heating or under microwave irradiation. Water (50 mL/mmol) was added, and the mixture was extracted with Et_2O (4 × 25 mL/mmol). The combined organic layers were washed with brine (50 mL/mmol), dried over Na_2SO_4, filtered, and the solvent was removed under reduced pressure. The residue was purified via column chromatography on silica.

1-(2-Iodophenyl)-1*H*-benzo[*d*]imidazole (4aa)

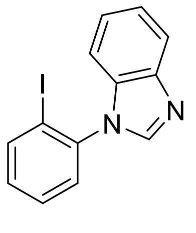

Following **GP1** *o*-fluoroiodobenzene (**S10a**, 700 µL, 6.00 mmol), 1*H*-benzo[*d*]imidazole (**S2f**, 473 mg, 4.00 mmol) and K_3PO_4 (4.25 g, 20.0 mmol) in DMF (20 mL) gave 1-(2-iodophenyl)-1*H*-benzo[*d*]imidazole (**4aa**, 1.12 g, 3.49 mmol, 87%) as a colorless solid after 3 h of conventional heating and column chromatography (Cy 2:1 EtOAc).

^{1}H NMR (601 MHz, $CDCl_3$) δ (ppm) 7.99 (d, J = 8.0 Hz, 1H), 7.97 (s, 1H), 7.86 (dd, J = 8.1, 1.1 Hz, 1H), 7.47 (t, J = 7.7 Hz, 1H), 7.32 (dt, J = 7.8, 1.3 Hz, 1H), 7.29 (t, J = 7.6 Hz, 1H), 7.24 (t, J = 7.6 Hz, 1H), 7.19 (d, J = 7.7 Hz, 1H), 7.10 (dd, J = 8.1, 1.1 Hz, 1H). **^{13}C NMR** (151 MHz, $CDCl_3$) δ (ppm) 143.3, 142.8, 140.5, 138.6, 134.3, 131.0, 129.6, 128.8, 123.7, 122.8, 120.5, 110.7, 97.0. **MS** (ESI) *m/z* 321.1 $[M+H]^+$. **IR** (ATR) ν (cm^{-1}) 3046, 3019, 1942, 1900, 1861, 1829, 1688, 1613, 1579, 1488, 1309, 1228, 1162, 1144, 1106, 1053, 1023, 1004, 989, 976, 931, 884, 849, 787, 760, 745, 717. **M_p** (°C) 131. Analytical data is in accordance with those reported in the literature.[12]

1-(2-Iodo-3-methylphenyl)-1*H*-benzo[*d*]imidazole (4ab)

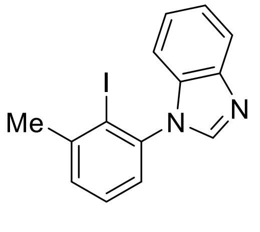

Following **GP1** 1-fluoro-2-iodo-3-methylbenzene (**S10b**, 1.42 g, 6.00 mmol), 1*H*-benzo[*d*]imidazole (**S2f**, 709 mg, 6.00 mmol) and K_3PO_4 (2.10 g, 10.0 mmol) in DMF (40 mL) gave 1-(2-iodo-3-methylphenyl)-1*H*-benzo[*d*]imidazole (**4ab**, 688 mg, 2.00 mmol, 60%) as a colorless solid after 0.5 h of microwave heating and column chromatography (Cy 3:1 EtOAc).

^{1}H NMR (601 MHz, $CDCl_3$) *δ* (ppm) 7.99 (s, 1H), 7.89 (d, *J* = 8.1 Hz, 1H), 7.42 – 7.38 (m, 2H), 7.33 (ddd, *J* = 8.2, 7.2, 1.2 Hz, 1H), 7.28 (ddd, *J* = 8.2, 7.2, 1.1 Hz, 1H), 7.19 (dd, *J* = 6.4, 2.8 Hz,

1H), 7.11 (d, *J* = 8.0 Hz, 1H), 2.60 (s, 3H). **^{13}C NMR** (151 MHz, $CDCl_3$) *δ* (ppm) 144.8, 143.2, 143.0, 139.2, 134.5, 130.7, 129.0, 126.1, 123.7, 122.8, 120.5, 110.8, 104.7, 29.6. **HRMS** (ESI) Calculated for $C_{14}H_{12}IN_2^+$ [M+H]$^+$: *m/z* 335.00197, found *m/z* 335.00383. **IR** (ATR) *v* (cm^{-1}) 3120, 2917, 1484, 1307, 1286, 1021, 840, 749, 711. **M$_P$** (°C) 160.

1-(3-Fluoro-2-iodophenyl)-1*H*-benzo[*d*]imidazole (4ac)

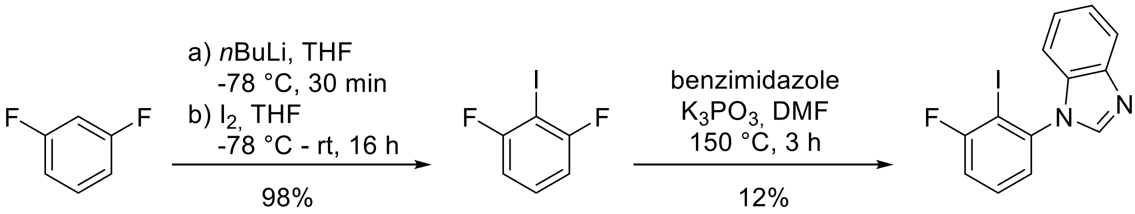

To a solution of 1,3-difluorobenzene (**S11**, 981 µL, 10.0 mmol) in dry THF (9.5 mL) at −78 °C was added *n*-butyllithium (2.5 M in *n*-hexane, 4.04 mL, 10.1 mmol) dropwise over 10 min. The solution was stirred at this temperature for 30 min, before a solution of I_2 (1.29 g, 5.01 mmol) in THF (5 mL) was added dropwise. The solution was allowed to reach room temperature over 16 h. Then, the solvent was removed (200 mbar, 45 °C) and the residue was purified by Kugelrohr distillation (100 °C, 12 mbar), so that 1,3-difluoro-2-iodobenzene (**S10c**, 1.17 g, 4.90 mmol, 98%) was isolated as a colorless liquid.

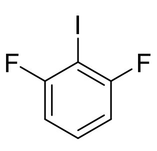

^{1}H NMR (601 MHz, $CDCl_3$) δ (ppm) 7.30 (tt, *J* = 8.4, 6.3 Hz, 1H), 6.89 (dd, *J* = 8.3, 6.0 Hz, 2H). **^{13}C NMR** (151 MHz, $CDCl_3$) δ (ppm) 163.7 (d, *J* = 5.5 Hz), 162.1 (d, *J* = 5.6 Hz), 130.7 (t, *J* = 9.5 Hz), 111.5 (dd, *J* = 23.4, 3.8 Hz). **^{19}F NMR** (565 MHz, $CDCl_3$) δ (ppm) -92.15 (q, *J* = 5.6 Hz). **MS** (EI) *m/z* 240.04 [M]$^{+\cdot}$. **IR** (ATR) v (cm^{-1}) 1589, 1460, 1284, 1234, 1034, 987, 772, 690. Analytical data is in accordance to those reported in literature.[13]

Following **GP1** 1*H*-benzo[*d*]imidazole (**S2f**, 354 mg, 3.00 mmol), 1,3-difluoro-2-iodobenzene (**S10c**, 720 mg, 3.00 mmol), K_3PO_4 (2.55 g, 12.0 mmol) and DMF (20 mL) gave 1-(3-fluoro-2-iodophenyl)-1*H*-benzo[*d*]imidazole (**4ac**, 119 mg, 350 µmol, 12%) after a short column chromatography (Cy 1:1 EtOAc) and further recrystallization of the obtained solid from cyclohexane with a few drops of EtOAc.

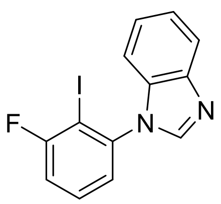

^{1}H NMR (600 MHz, $CDCl_3$) δ (ppm) 8.05 (s, 1H), 7.92 (d, *J* = 8.1 Hz, 1H), 7.54 (td, *J* = 8.0, 5.8 Hz, 1H), 7.38 (t, *J* = 7.6 Hz, 1H), 7.33 (t, *J* = 7.6 Hz, 1H), 7.30 – 7.22 (m, 2H), 7.17 (d, *J* = 8.0 Hz, 1H). **^{13}C NMR** (151 MHz, $CDCl_3$) δ (ppm) 163.1 (d, *J* = 248.2 Hz), 143.3, 142.7, 140.6 (d, *J* = 3.3 Hz), 134.2, 130.8 (d, *J* = 9.0 Hz), 124.6 (d, *J* = 3.3 Hz), 124.0, 123.1, 120.7, 116.6 (d, *J* = 24.5 Hz), 110.7, 85.9 (d, *J* = 26.7 Hz). **^{19}F NMR** (565 MHz, $CDCl_3$) δ (ppm) -86.82 (t, *J* = 6.6 Hz). **HRMS** (ESI) Calculated for $C_{13}H_9FIN_2^+$ [M+H]$^+$ *m/z* 338.97890, found *m/z* 338.97866. **IR** (ATR) *v* (cm^{-1}) 3122, 1583, 1489, 1399, 1219, 1167, 1016, 804, 751. **Mp** (°C) 245-246.

1-(2-Iodo-4-methylphenyl)-1*H*-benzo[*d*]imidazole (4af)

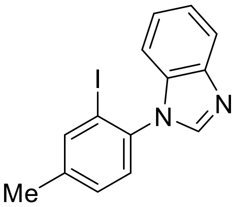

Following **GP1** 1-fluoro-2-iodo-4-methylbenzene (**S10d**, 944 mg, 4.00 mmol), 1*H*-benzo[*d*]imidazole (**S2f**, 709 mg, 6.00 mmol) and K_3PO_4 (4.24 g, 20.0 mmol) in DMF (40 mL) gave 1-(2-iodo-4-methylphenyl)-1*H*-benzo[*d*]imidazole (**4af**, 549 mg, 1.64 mmol, 41%) as a colorless solid after 18 h of conventional heating and column chromatography (Cy 2:1 EtOAc).

^{1}H NMR (601 MHz, $CDCl_3$) δ (ppm) 8.01 (s, 1H), 7.92 – 7.87 (m, 2H), 7.37 – 7.29 (m, 3H), 7.28 (d, J = 7.9 Hz, 1H), 7.14 (dt, J = 8.0, 1.0 Hz, 1H), 2.44 (s, 3H). **^{13}C NMR** (151 MHz, $CDCl_3$) δ (ppm) 143.0, 141.7, 140.9, 136.1, 134.5, 130.4, 128.4, 123.9, 122.9, 120.5, 110.9, 96.9, 20.9 (two signals are overlapping). **HRMS** (ESI) Calculated for $C_{14}H_{12}IN_2^+$ [M+H]$^+$ *m/z* 335.00397, found *m/z* 335.00382. **IR** (ATR) *ν* (cm^{-1}) 2915, 1612, 1495, 1455, 1308, 1283, 1228, 1053, 1004, 813, 714. **M$_P$** (°C) 122.

1-(5-Chloro-2-iodophenyl)-1*H*-benzo[*d*]imidazole (4ah)

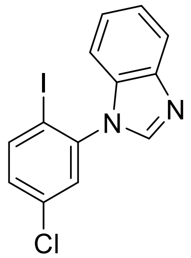

Following **GP1** 4-chloro-2-fluoro-1-iodobenzene (**S10e**, 1.30 g, 5.07 mmol), 1*H*-benzo[*d*]imidazole (**S2f**, 829 mg, 7.02 mmol) and K_3PO_4 (5.31 g, 25.0 mmol) in DMF (100 mL) gave 1-(5-chloro-2-iodophenyl)-1*H*-benzo[*d*]imidazol (**4ah**, 756 mg, 2.13 mmol, 43%) as a colorless solid after 3 h of conventional heating and column chromatography (Cy 3:1 EtOAc).

^{1}H NMR (600 MHz, $CDCl_3$) δ (ppm) 8.03 (s, 1H), 7.99 (d, J = 8.5 Hz, 1H), 7.92 (d, J = 7.9 Hz, 1H), 7.45 (s, 1H), 7.37 (dt, J = 22.6, 7.3 Hz, 2H), 7.29 (s, 1H), 7.18 (d, J = 7.9 Hz, 1H). **^{13}C NMR** (151 MHz, DMSO-d_6) δ (ppm) 144.1, 143.3, 141.5, 140.2, 134.5, 134.3, 131.7, 129.7, 124.0, 122.9, 120.2, 111.3, 97.2. **HRMS** (ESI) Calculated for $C_{13}H_9ClIN_2^+$ [M+H]$^+$ *m/z* 354.94935, found *m/z* 354.94885. **IR** (ATR) ν (cm^{-1}) 3050, 2808, 1570, 1487, 1400, 1245, 996, 929, 707. **Mp** (°C) 127.

1-(5-Bromo-2-iodophenyl)-1*H*-benzo[*d*]imidazole (4ai)

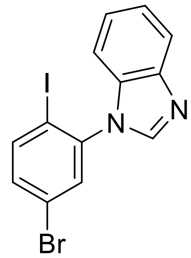

Following **GP1** 4-bromo-2-fluoro-1-iodobenzene (**S10f**, 903 mg, 3.00 mmol), 1*H*-benzo[*d*]imidazole (**S2f**, 496 mg, 4.20 mmol) and K_3PO_4 (1.91 g, 9.00 mmol) in DMF (30 mL) gave 1-(5-bromo-2-iodophenyl)-1*H*-benzo[*d*]imidazol (**4ai**, 904 mg, 2.26 mmol, 75%) as a colorless solid after 4 h of conventional heating and column chromatography (Cy 1:1 EtOAc).

^{1}H NMR (600 MHz, $CDCl_3$) δ (ppm) 8.00 (s, 1H), 7.89 (t, J = 7.1 Hz, 2H), 7.57 (d, J = 1.4 Hz, 1H), 7.39 (dd, J = 8.4, 1.7 Hz, 1H), 7.36 (t, J = 7.6 Hz, 1H), 7.32 (t, J = 7.6 Hz, 1H), 7.16 (d, J = 7.9 Hz, 1H). **^{13}C NMR** (151 MHz, $CDCl_3$) δ (ppm) 143.3, 142.5, 141.5, 140.1, 134.2, 134.0, 131.9, 124.1, 123.2, 123.1, 120.8, 110.6, 95.2. **HRMS** (ESI) Calculated for $C_{13}H_9BrIN_2^+$ [M+H]$^+$ *m/z* 398.89883, found *m/z* 398.89841. **IR** (ATR) ν (cm^{-1}) 3012, 2998, 1654, 1539, 1206, 680. **Mp** (°C) 152-154.

3-(1*H*-Benzo[*d*]imidazol-1-yl)-4-iodobenzonitrile (4aj)

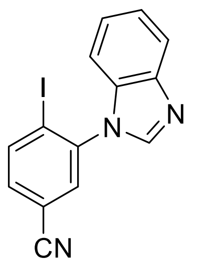

Following **GP1** 3-fluoro-4-iodo-benzonitrile (**S10g**, 370 mg, 1.50 mmol), 1*H*-benzo[*d*]imidazole (**S2f**, 248 mg, 2.10 mmol) and K_3PO_4 (1.06 g, 4.99 mmol) in DMF (1 mL) gave 3-(1*H*-benzo[*d*]imidazol-1-yl)-4-iodobenzonitrile (**4ai**, 249 mg, 720 µmol, 48%) as a colorless solid after 2 h of conventional heating at 50 °C and column chromatography (Cy 1:1 EtOAc).

^{1}H NMR (601 MHz, $CDCl_3$) δ (ppm) 8.23 (d, *J* = 8.3 Hz, 1H), 8.02 (s, 1H), 7.89 (d, *J* = 7.9 Hz, 1H), 7.71 – 7.66 (m, 1H), 7.52 (dd, *J* = 8.3, 1.9 Hz, 1H), 7.37 (td, *J* = 7.6, 1.3 Hz, 1H), 7.34 (td, *J* = 7.7, 1.1 Hz, 1H) 7.13 (d, *J* = 7.7 Hz, 1H). **^{13}C NMR** (151 MHz, $CDCl_3$) δ (ppm) 143.1, 142.1, 141.9, 140.1, 133.5, 131.5, 124.4, 123.5, 120.8, 116.8, 114.0, 110.2, 103.7. **HRMS** (ESI) Calculated for $C_{14}H_9IN_3^+$ $[M+H]^+$ *m/z* 345.98357, found *m/z* 345.98289. **IR** (ATR) ν (cm^{-1}) 3396, 3338, 3221, 3052, 2230, 1683, 1509, 1254, 744, 716. **Mp** (°C) 173.

1-(2-Iodo-5-nitrophenyl)-1*H*-benzo[*d*]imidazole (4ak)

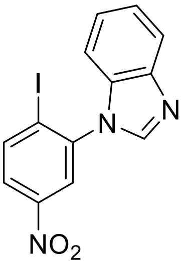

Following **GP1** 2-fluoro-1-iodo-4-nitrobenzene (**S10h**, 533 mg, 2.00 mmol), 1*H*-benzo[*d*]imidazole (**S2f**, 259 mg, 2.19 mmol) and K_3PO_4 (1.06 g, 4.99 mmol) in DMF (1 mL) gave 1-(2-iodo-5-nitrophenyl)-1*H*-benzo[*d*]imidazole (**4ak**, 249 mg, 720 µmol, 48%) as a colorless solid after 2 h of conventional heating at 50 °C and column chromatography (Cy 1:1 EtOAc).

^{1}H NMR (600 MHz, $CDCl_3$) δ (ppm) 8.33 (d, *J* = 8.7 Hz, 1H), 8.29 (s, 1H), 8.13 (d, *J* = 8.7 Hz, 1H), 8.09 (s, 1H), 7.95 (d, *J* = 8.0 Hz, 1H), 7.42 (t, *J* = 7.6 Hz, 1H), 7.38 (t, *J* = 7.6 Hz, 1H), 7.17 (d, *J* = 8.0 Hz, 1H). **^{13}C NMR** (151 MHz, $CDCl_3$) δ (ppm) 148.8, 143.2, 142.1, 141.6, 140.1, 133.7, 125.0, 124.4, 123.5, 123.4, 120.9, 110.2, 105.7. **HRMS** (ESI) Calculated for $C_{13}H_9IN_3O_2^+$ $[M+H]^+$ *m/z* 365.97340, found *m/z* 365.97307. **IR** (ATR) ν (cm^{-1}) 3086, 2922, 2853, 1612, 1571, 1523, 1344, 1225, 1092, 1037, 864, 707. **Mp** (°C) 204.

2-Bromo-1-(2-iodophenyl)-1*H*-benzo[*d*]imidazole (4am)

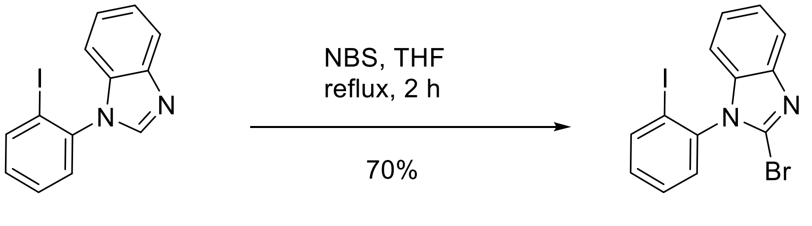

4aa **4am**

A slightly modified literature procedure was used.[14] NBS (182 mg, 1.02 mmol) was added to a solution of 1-(2-iodophenyl)-1*H*-benzo[*d*]imidazole (**4aa**, 320 mg, 1.00 mmol) in THF (4 mL) and the reaction mixture was refluxed for 2 h. The solvent was removed under reduced pressure, the residue was dissolved in EtOAc (10 mL) and H_2O (10 mL) and the phases were separated. The aqueous layer was extracted with EtOAc (2 × 10 mL), and the combined organic phases were washed with brine (10 mL), dried over Na_2SO_4, filtered, and the solvent was removed under reduced pressure. The residue was purified via column chromatography

(Cy 7:1 EtOAc) to obtain 2-bromo-1-(2-iodophenyl)-1*H*-benzo[*d*]imidazole (**4am**, 279 mg, 699 µmol, 70%) as a colorless solid.

^{1}H NMR (600 MHz, $CDCl_3$) δ (ppm) 8.05 (dd, *J* = 8.0, 1.4 Hz, 1H), 7.79 (dt, *J* = 8.2, 0.9 Hz, 1H), 7.57 (td, *J* = 7.6, 1.4 Hz, 1H), 7.38 (dd, *J* = 7.8, 1.6 Hz, 1H), 7.30 (tdd, *J* = 7.8, 2.4, 1.4 Hz, 2H), 7.24 (ddd, *J* = 8.3, 7.3, 1.1 Hz, 1H), 6.94 (dt, *J* = 8.1, 0.9 Hz, 1H). **^{13}C NMR** (151 MHz, $CDCl_3$) δ (ppm) 143.1, 140.4, 138.2, 136.6, 131.7, 130.1, 129.8, 129.7, 123.9, 123.2, 119.5, 110.5, 98.9. **HRMS** (EI) Calculated for $C_{13}H_8BrIN_2^{+\cdot}$ $[M]^{\cdot+}$ *m/z* 397.89101, found *m/z* 397.89211. **IR** (ATR) ν (cm^{-1}) 2922, 1435, 1370, 1300, 1263, 981, 757. **Mp** (°C) 96-97.

1-(2-Iodophenyl)-2-methylbenzo[*d*]imidazole (4ap)

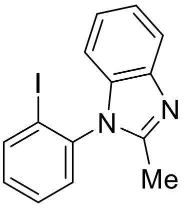

Following **GP1** 2-fluoroiodobenzene (**S10a**, 700 µL, 6.00 mmol), 2-methyl-1*H*-benzo[*d*]imidazole (**S2a**, 638 mg, 4.00 mmol) and K_3PO_4 (4.24 g, 20.0 mmol) in DMF (60 mL) gave 1-(2-iodophenyl)-2-methylbenzo[*d*]imidazole (**4ap**, 1.05 g, 3.29 mmol, 56%) as a colorless solid after 18 h of conventional heating and column chromatography (Cy 2:1 EtOAc).

^{1}H NMR (601 MHz, $CDCl_3$) *δ* (ppm) 8.07 (dd, *J* = 8.0, 1.4 Hz, 1H), 7.77 (dt, *J* = 8.1, 0.9 Hz, 1H), 7.57 (td, *J* = 7.6, 1.4 Hz, 1H), 7.37 (dd, *J* = 7.8, 1.6 Hz, 1H), 7.32 – 7.26 (m, 2H), 7.20 (ddd, *J* = 8.2, 7.2, 1.1 Hz, 1H), 6.90 (dt, *J* = 8.0, 0.9 Hz, 1H), 2.43 (s, 3H). **^{13}C NMR** (151 MHz, $CDCl_3$) *δ* (ppm) 151.3, 142.4, 140.4, 138.9, 135.9, 131.2, 129.9, 129.5, 122.8, 122.6, 119.1, 110.0, 98.6, 14.5. **HRMS** (ESI) Calculated for $C_{14}H_{12}IN_2^+$ $[M+H]^+$ *m/z* 335.00397, found *m/z* 335.00377. **IR** (ATR) *ν* (cm^{-1}) 2988, 1518, 1473, 1389, 1321, 1240, 1011, 771, 743. **Mp** (°C) 134. Analytical data is in accordance with those reported in the literature.[15]

1-(2-Iodophenyl)-5,6-dimethyl-1*H*-benzo[*d*]imidazole (4ar)

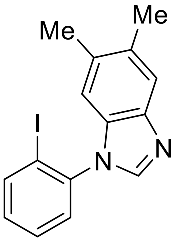

Following **GP1** 5,6-dimethyl-1*H*-benzo[*d*]imidazole (**S2d**, 731 mg, 5.00 mmol), 2-fluoroiodobenzene (**S10a**, 875 µL, 7.50 mmol), K_3PO_4 (5.30 g, 25.0 mmol) and DMF (50 mL) gave 1-(2-iodophenyl)-5,6-dimethyl-1*H*-benzo[*d*]imidazole (**4ar**, 1.56 g, 4.49 mmol, 90%) as a colorless solid after column chromatography (Cy 1:1 EtOAc),

^{1}H NMR (601 MHz, $CDCl_3$) δ (ppm) 8.05 (dd, *J* = 8.0, 1.4 Hz, 1H), 7.90 (s, 1H), 7.65 (s, 1H), 7.53 (td, *J* = 7.6, 1.4 Hz, 1H), 7.39 (dd, *J* = 7.8, 1.6 Hz, 1H), 7.26 – 7.23 (m, 1H), 6.91 (s, 1H), 2.40 (s, 3H), 2.34 (s, 3H). **^{13}C NMR** (151 MHz, $CDCl_3$) δ (ppm) 142.2, 142.0, 140.6, 139.2, 133.1, 133.0, 131.8, 130.9, 129.6, 128.9, 120.6, 110.8, 97.2, 20.7, 20.5. **HRMS** (ESI) Calculated for $C_{15}H_{14}IN_2^+$ $[M+H]^+$ *m/z* 349.01962, found *m/z* 349.01898. **IR** (ATR) *ν* (cm^{-1}) 3088, 2964, 2944, 2918, 1484, 1224, 972, 869, 841, 759. **Mp** (°C) 151-152.

1-(5-Chloro-2-iodophenyl)-5,6-dimethyl-1*H*-benzo[*d*]imidazole (4at)

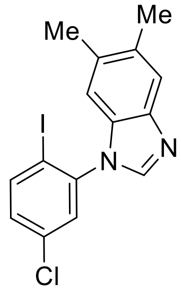

Following **GP1** 4-chloro-2-fluoro-1-iodobenzene (**S10e**, 770 mg, 3.00 mmol), 5,6-dimethyl-1*H*-benz[*d*]imidazole (**S2d**, 205 mg, 1.40 mmol) and K_3PO_4 (1.59 g, 7.49 mmol) in DMF (10 mL) gave 1-(5-chloro-2-iodophenyl)-5,6-dimethyl-1*H*-benzo[*d*]imidazole (**4at**, 280 mg, 731 µmol, 52%) as a colorless solid after 2 h of conventional heating and column chromatography (Cy 3:1 EtOAc).

^{1}H NMR (600 MHz, CD_3OD) δ (ppm) 8.11 (s, 1H), 8.06 (d, *J* = 8.6 Hz, 1H), 7.57 (d, *J* = 2.4 Hz, 1H), 7.52 (s, 1H), 7.37 (dd, *J* = 8.6, 2.4 Hz, 1H), 6.91 (s, 1H), 2.38 (s, 3H), 2.33 (s, 3H). ^{13}C NMR (151 MHz, CD_3OD) δ (ppm) 143.5, 142.6, 142.1, 141.3, 136.5, 134.9, 133.8, 133.5, 132.4, 130.3, 120.4, 111.9, 95.8, 20.5, 20.3. **HRMS** (ESI) Calculated for $C_{15}H_{13}ClIN_2^+$ $[M+H]^+$ *m/z* 382.97824, found *m/z* 382.98021. **IR** (ATR) ν (cm^{-1}) 1679, 1574, 1553, 1486, 1467, 1408, 1215, 1150, 1094, 1023, 984, 807, 752, 704. **MP** (°C) 146.

1-(5-Bromo-2-iodophenyl)-5,6-dimethyl-1*H*-benzo[*d*]imidazole (4au)

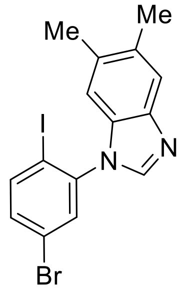

Following **GP1** 4-bromo-2-fluoro-1-iodobenzene (**S10f**, 159 mg, 527 µmol), 5,6-dimethyl-1*H*-benz[*d*]imidazole (**S2d**, 322 mg, 2.20 mmol) and K_3PO_4 (1.06 g, 5.00 mmol) in DMF (12 mL) gave 1-(5-bromo-2-iodophenyl)-5,6-dimethyl-1*H*-benzo[*d*]imidazole (**4au**, 129 mg, 302 µmol, 57%) as a colorless solid after 3 h of conventional heating and column chromatography (Cy 1:1 EtOAc -> EtOAc).

^{1}H NMR (601 MHz, $CDCl_3$) δ (ppm) 7.90-7.92 (m, 2H), 7.66 (s, 1H), 7.56 (s, 1H), 7.40 (d, *J* = 10.8 Hz, 1H), 6.94 (s, 1H), 2.42 (s, 3H), 2.38 (s, 3H). **^{13}C NMR** (151 MHz, $CDCl_3$) δ (ppm) 141.7, 141.4, 140.3, 138.9, 133.9, 133.4, 132.5, 132.0, 131.8, 123.0, 120.6, 110.5, 95.2, 20.6, 20.3. **HRMS** (ESI) Calculated for $C_{15}H_{13}BrIN_2^+$ $[M+H]^+$ *m/z* 426.93013, found *m/z* 426.93001. **IR** (ATR) ν (cm^{-1}) 2961, 1488, 1404, 1258, 1027, 841, 703. **MP** (°C) 160.

1-(2-Iodophenyl)-3-methyl-1*H*-benzo[*d*]imidazol-3-ium triflate (4av)

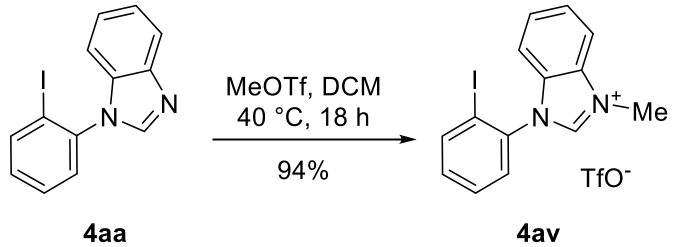

4aa **4av**

Following a literature procedure,[16] a solution of 1-(2-iodophenyl)-1*H*-benzo[*d*]imidazole (**4aa**, 320 mg, 1.00 mmol) and MeOTf (170 µL, 1.50 mmol) in dry DCM (5 mL) was stirred at room temperature for 18 h. The solvent was removed under reduced pressure and the residue was suspended in Et_2O (2 mL), filtered, and washed with Et_2O (2 × 2 mL), so that 1-(2-iodophenyl)-3-methyl-1*H*-benzo[*d*]imidazol-3-ium triflate (**4av**, 455 mg, 940 µmol, 94%) was obtained as a colorless powder.

^{1}H NMR (601 MHz, DMSO-d_6) *δ* (ppm) 10.13 (s, 1H), 8.23 (d, *J* = 7.5 Hz, 1H), 8.19 (d, *J* = 8.4 Hz, 1H), 7.83 – 7.79 (m, 2H), 7.76 (td, *J* = 7.7, 1.4 Hz, 1H), 7.72 (t, *J* = 7.8 Hz, 1H),

7.53 (td, J = 7.8, 1.7 Hz, 1H), 7.50 (d, J = 8.3 Hz, 1H), 4.24 (s, 3H). **^{13}C NMR** (151 MHz, DMSO-d_6) δ (ppm) 143.6, 140.3, 135.4, 133.1, 131.4, 131.3, 130.2, 129.3, 127.7, 127.1, 120.7 (q, J = 322.4 Hz), 114.2, 113.7, 97.3, 33.8. **^{19}F NMR** (565 MHz, DMSO-d_6) δ (ppm) -77.80. **HRMS** (ESI) Calculated for $C_{14}H_{12}IN_2^+$ [M-OTf]$^+$ *m/z* 335.00397, found *m/z* 335.00380. **IR** (ATR) *v* (cm^{-1}) 3157, 3105, 1567, 1247, 1157, 1025, 757. **M$_P$** (°C) 150.

1-(2-Iodophenyl)-3-phenyl-1*H*-benzo[*d*]imidazol-3-ium triflate (4aw)

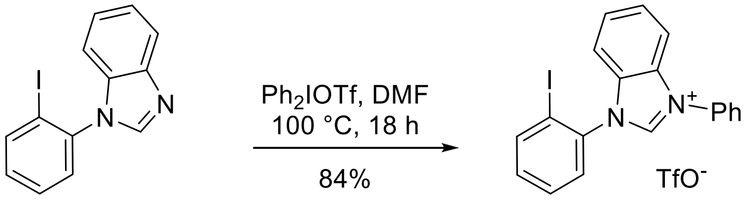

4aa **4aw**

Following a literature procedure[17] 1-(2-iodophenyl)-1*H*-benzo[*d*]imidazole (**4aa**, 320 mg, 1.00 mmol), Ph_2IOTf (645 mg, 1.50 mmol) and CuOTf (18.1 mg, 50.0 µmol) were stirred in DMF (5 mL) for 18 h at 100 °C. The solvent was removed under reduced pressure. The residue was purified via column chromatography on silica (DCM -> DCM 90:10 MeOH), suspended in Et_2O (1 mL), and filtered to obtain 1-(2-iodophenyl)-3-phenyl-1*H*-benzo[*d*]imidazol-3-ium triflate (**4aw**, 457 mg, 836 µmol, 84%) was obtained as a colorless solid with 90% purity.

^{1}H NMR (600 MHz, $CDCl_3$) δ (ppm) 9.76 (s, 1H), 8.07 (dd, J = 8.0, 1.3 Hz, 1H), 8.00 (dd, J = 7.9, 1.5 Hz, 1H), 7.86-7.88 (m, 2H), 7.79 (d, J = 7.8 Hz, 1H), 7.76 – 7.61 (m, 6H), 7.41 (dd, J = 7.9, 1.3 Hz, 2H). **^{13}C NMR** (151 MHz, $CDCl_3$) δ 141.9, 140.5, 134.9, 133.5, 132.5, 132.0, 131.5, 131.3, 131.3, 130.9, 130.8, 130.8, 129.9, 128.6, 128.5, 125.4, 120.5 (q, J = 320.3 Hz), 114.2, 114.0, 95.3. **^{19}F NMR** (565 MHz, $CDCl_3$) δ (ppm) -78.44. **HRMS** (ESI) Calculated for $C_{19}H_{14}IN_2^+$ [M-OTf]$^+$ *m/z* 397.01962, found *m/z* 397.01905. **IR** (ATR) *v* (cm^{-1}) 3108, 3010, 1550, 1486, 1249, 1138, 1028, 787, 748, 690. **M$_p$** (°C) 102-104

1-(2-Iodo-3-methylphenyl)-3,5,6-trimethyl-1*H*-benzo[*d*]imidazol-3-ium triflate (4ax)

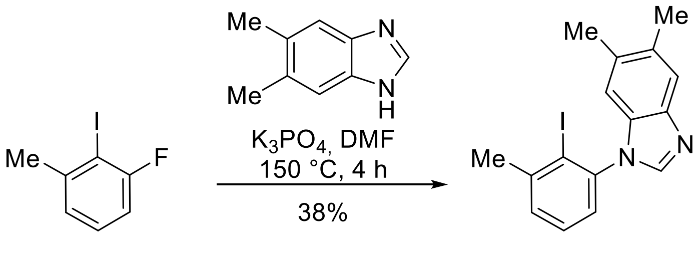

S10b **S12**

Following **GP1** 1-fluoro-2-iodo-3-methylbenzene (**S10b**, 3.89 g, 16.5 mmol), 5,6-dimethyl-1*H*-benzo[*d*]imidazole (**S2a**, 2.19 g, 15.0 mmol) and K_3PO_4 (12.7 g, 60.0 mmol) in DMF (100 mL) gave 1-(2-iodo-3-methylphenyl)-5,6-dimethyl-1*H*-benzo[*d*]imidazole (**S12**, 2.63 g, 7.25 mmol, 48%) as a colorless solid after 4 h of conventional heating and column chromatography (Cy 2:1 EtOAc).

^{1}H NMR (601 MHz, $CDCl_3$) δ (ppm) 7.88 (s, 1H), 7.64 (s, 1H), 7.44 – 7.37 (m, 2H), 7.17 (dd, *J* = 5.5, 3.8 Hz, 1H), 6.88 (s, 1H), 2.60 (s, 3H), 2.40 (s, 3H), 2.33 (s, 3H). **^{13}C NMR** (151 MHz, $CDCl_3$) δ (ppm) 144.7, 142.3, 141.9, 139.5, 133.0, 133.0, 131.6, 130.5, 126.1, 120.5, 110.8, 104.8, 29.6, 20.6, 20.4 (one signal is missing). **HRMS** (ESI) Calculated for $C_{16}H_{16}IN_2^+$ $[M+H]^+$ *m/z* 363.03527, found *m/z* 363.03596. **IR** (ATR) *ν* (cm^{-1}) 2967, 2901, 1483, 1227, 1024, 879, 884, 783, 732. **M_p** (°C) 171-173.

1-(2-Iodo-3-methylphenyl)-3,5,6-trimethyl-1*H*-benzo[*d*]imidazol-3-ium triflate (4ax)

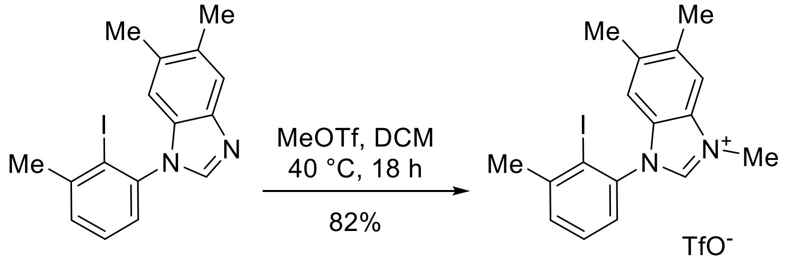

S12 **4ax**

Following a literature procedure,[16] a solution of 1-(2-iodo-3-methylphenyl)-5,6-dimethyl-1*H*-benzo[*d*]imidazole (**S12**, 362 mg, 1.00 mmol) and MeOTf (283 µmol, 2.50 mmol) in dry DCM (5 mL) was stirred at 40 °C for 18 h in a pressure vial. The solvent was removed under reduced pressure, and the residue was suspended in Et_2O (2 mL), filtered, and washed with Et_2O (2 × 2 mL), so that 1-(2-iodo-3-methylphenyl)-3,5,6-trimethyl-1*H*-benzo[*d*]imidazol-3-ium triflate (**4ax**, 434 mg, 824 µmol, 82%) was obtained as a colorless powder.

^{1}H NMR (601 MHz, $CDCl_3$) δ (ppm) 9.44 (s, 1H), 7.63 (s, 1H), 7.51 (dd, *J* = 7.6, 1.8 Hz, 1H), 7.47 (t, *J* = 7.6 Hz, 1H), 7.40 (dd, *J* = 7.4, 2.0 Hz, 1H), 6.99 (s, 1H), 4.26 (s, 3H), 2.57 (s, 3H), 2.46 (s, 3H), 2.36 (s, 3H). **^{13}C NMR** (151 MHz, $CDCl_3$) δ (ppm) 145.3, 141.3, 138.5, 138.2, 135.7, 132.6, 130.3, 130.1, 129.9, 129.8, 126.3, 120.7 (q, *J* = 320.3 Hz), 113.3, 113.1, 102.6, 34.0, 29.3, 20.7. **^{19}F NMR** (565 MHz, $CDCl_3$) δ (ppm) -78.38. **HRMS** (ESI) Calculated for $C_{17}H_{18}IN_2^+$ $[M-OTf]^+$ *m/z* 377.05092, found *m/z* 377.05011. **IR** (ATR) *ν* (cm^{-1}) 2988, 2922, 1563, 1454, 1248, 1154, 1028, 791. **M_p** (°C) 186-187.

1-(2-Iodo-3-methylphenyl)-5,6-dimethyl-3-phenyl-1*H*-benzo[*d*]imidazol-3-ium triflate (4ay)

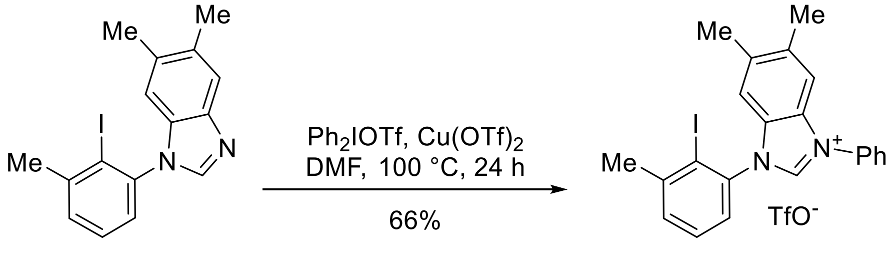

S12 **4ay**

Following a modified literature procedure[17] iodoarene **S12** (797 mg, 2.20 mmol), Ph_2IOTf (1.42 g, 3.30 mmol) and $Cu(OTf)_2$ (39.8 mg, 110 µmol) were suspended in dry DMF (12 mL) under an Ar atmosphere and stirred for 24 h at 100 °C. The solvent was removed under reduced pressure and the residue was purified via column chromatography on silica (DCM

95:5 MeOH) and subsequent suspension of the product fraction in EtOAc (10 mL) to obtain 1-(2-iodo-3-methylphenyl)-5,6-dimethyl-3-phenyl-1*H*-benzo[*d*]imidazol-3-ium triflate (**4ay**, 848 mg, 1.44 mmol, 66%) as a colorless powder.

^{1}H NMR (600 MHz, $CDCl_3$) δ (ppm) 9.62 (s, 1H), 7.87 (d, *J* = 7.2 Hz, 2H), 7.77 (d, *J* = 7.5 Hz, 1H), 7.70 (t, *J* = 7.4 Hz, 2H), 7.65 (t, *J* = 7.4 Hz, 1H), 7.59 – 7.52 (m, 2H), 7.51 (s, 1H), 7.10 (s, 1H), 2.61 (s, 3H), 2.44 (s, 3H), 2.41 (s, 3H). **^{13}C NMR** (151 MHz, $CDCl_3$) δ (ppm) 145.1, 140.8, 139.0, 138.8, 135.5, 132.9, 131.2, 130.8, 130.6, 130.2, 129.8, 127.1, 125.4, 120.7 (q, *J* = 320.4 Hz), 113.7, 113.5, 102.5, 29.4, 20.9, 20.8 (one signal is overlapping). **^{19}F NMR** (565 MHz, $CDCl_3$) δ (ppm) -78.42. **HRMS** (ESI) Calculated for $C_{22}H_{20}IN_2^+$ [M-OTf]$^+$ *m/z* 439.06657, found *m/z* 439.06573. **IR** (ATR) *ν* (cm^{-1}) 3094, 2996, 1541, 1262, 1220, 1142, 1025, 771, 695. **M$_p$** (°C) 217-218.

1-(2-Iodo-3-methylphenyl)-3-mesityl-5,6-dimethyl-1*H*-benzo[*d*]imidazol-3-ium triflate (4az)

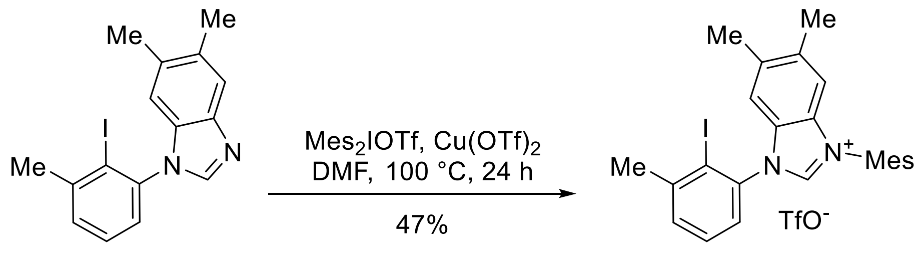

S12 **4az**

Following a modified literature procedure[17], iodoarene **S12** (1.81 g, 5.00 mmol), Mes_2IOTf (3.86 g, 7.50 mmol), and $Cu(OTf)_2$ (90.5 mg, 250 µmol) were suspended in dry DMF (25 mL) under an Ar atmosphere and stirred for 24 h at 100 °C. The solvent was removed under reduced pressure, and the residue was purified via column chromatography on silica (EtOAc 99:1 HOAc) to obtain 1-(2-iodo-3-methylphenyl)-3-mesityl-5,6-dimethyl-1*H*-benzo[*d*]imidazol-3-ium triflate (**4az**, 1.48 g, 2.36 mmol, 47%) as a colorless powder.

^{1}H NMR (600 MHz, $CDCl_3$) δ (ppm) 9.69 (s, 1H), 7.67 (d, *J* = 7.1 Hz, 1H), 7.61 – 7.49 (m, 2H), 7.13 (d, *J* = 5.9 Hz, 2H), 7.10 (s, 1H), 7.06 (s, 1H), 2.61 (s, 3H), 2.47 – 2.35 (m, 9H), 2.13 (s, 3H), 2.10 (s, 3H). **^{13}C NMR** (151 MHz, $CDCl_3$) δ (ppm) 145.1, 142.2, 141.8, 139.0, 136.0, 135.4, 134.8, 132.9, 130.4, 130.1, 130.0 130.0, 128.0, 127.1, 120.6 (q, *J* = 320.7 Hz), 113.8, 113.0, 102.5, 29.3, 21.3, 20.7, 18.1, 17.1. **^{19}F NMR** (565 MHz, $CDCl_3$) δ (ppm) -78.51. **HRMS** (ESI) Calculated for $C_{25}H_{26}IN_2^+$ [M-OTf]$^+$ *m/z* 481.11352, found *m/z* 481.11288. **IR** (ATR) *ν* (cm^{-1}) 3095, 2986, 2922, 1541, 1477, 1253, 1147, 1028, 851. **M$_p$** (°C) 98-99.

1-(2-Iodophenyl)-1*H*-indazole (4bc)

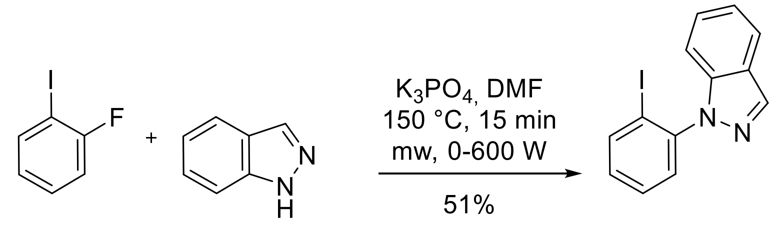

S10a **S13** **4bc**

Following a literature procedure,[11] 2-fluoroiodobenzene (**S10a**, 0.35 mL, 3.00 mmol) and 1*H*-indazole (**S13**, 240 mg, 2.00 mmol) were dissolved in DMF (20 mL). The homogenous solution was equally distributed to 2 MW-vials, and to each was added K_3PO_4 [1.06 g, 5.00 mmol (2.12 g, 10.0 mmol in total)]. The vials were placed in the microwave and were stirred at 150 °C with 0-600 W for 15 min. Afterwards the vials were combined, and H_2O (50 mL) was added. The mixture was extracted with Et_2O (4 x 25 mL) and the combined organic phases were washed with brine (50 mL), dried over Na_2SO_4, and the solvent was removed under reduced pressure. The residue was purified via column chromatography on silica (Cy/EtOAc 30:1) to give 1-(2-iodophenyl)-1*H*-indazole (**4bc**, 0.332 g, 1.02 mmol, 51%) as a yellow solid.

^{1}H NMR ($CDCl_3$, 360 MHz) δ (ppm) 8.26 (d, J = 1.0 Hz, 1H), 8.04 (dd, J = 7.9, 1.4 Hz, 1H), 7.83 (dt, J = 8.1, 1.1 Hz, 1H), 7.49 (ddd, J = 7.9, 7.3, 1.4 Hz, 1H), 7.45 – 7.35 (m, 2H), 7.29 – 7.16 (m, 3H). **^{13}C NMR** ($CDCl_3$, 91 MHz) δ (ppm) 141.8, 140.0, 139.9, 135.1, 130.4, 129.2, 126.8, 124.1, 121.2, 121.0, 120.8, 110.3, 96.5. **HRMS** (ESI) Calculated for $C_{13}H_{10}IN_2^+$ $[M+H]^+$ m/z = 320.98832, found: m/z 320.98842. **IR** (ATR) $\tilde{\nu}$ (cm^{-1}) 3058, 1629, 1579, 1497, 1481, 1412, 1199, 1080, 1020, 981. **Mp** (°C) 54–56. Analytical data is in accordance to those reported in literature.[11]

1-(2-Iodo-3-methylphenyl)-1*H*-indazole (4bd)

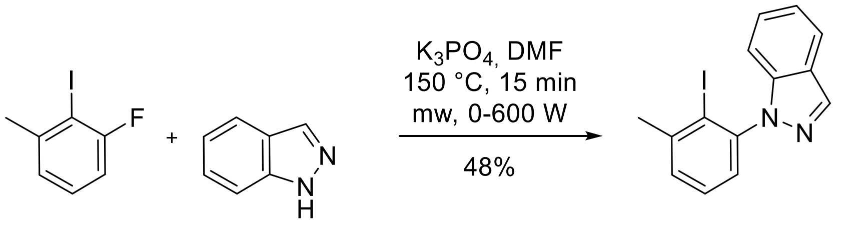

S10b **S13** **4bd**

Following a literature procedure,[18] 1-fluoro-2-iodo-3-methylbenzene (**S10b**, 177 mg, 0.750 mmol) and 1*H*-indazole (**S13**, 59.1 mg, 0.500 mmol) were dissolved in DMF (5 mL) in a PFA-MW-vial. K_3PO_4 (530 mg, 2.50 mmol) was added, and the vial was placed in the microwave and was stirred at 150 °C with 0-300 W for 2 h. Afterward H_2O (10 mL) was added. The mixture was extracted with Et_2O (4 x 10 mL) and the combined organic phases were washed with brine (10 mL), dried over Na_2SO_4, filtered, and the solvent was removed under reduced pressure. The residue was purified via column chromatography on silica gel (cyclohexane/EtOAc 100:0 → 20:1) to give 1-(2-iodo-3-methylphenyl)-1*H*-indazole (**4bd**, 81.0 mg, 0.240 mmol, 48%) as a colorless solid.

^{1}H NMR ($CDCl_3$, 601 MHz) δ (ppm) 8.24 (s, 1H), 7.82 (d, J = 8.1 Hz, 1H), 7.42 – 7.35 (m, 3H), 7.23 (t, J = 7.2 Hz, 2H), 7.17 (d, J = 8.4 Hz, 1H), 2.60 (s, 3H). **^{13}C NMR** ($CDCl_3$, 151 MHz) δ (ppm) 144.2, 142.4, 140.2, 134.9, 130.2, 128.6, 126.8, 126.6, 124.2, 121.2, 121.0, 110.4, 104.4, 29.3. **HRMS** (ESI) Calculated for $C_{14}H_{11}IN_2Na^+$ $[M+Na]^+$ m/z = 356.98592, found: m/z 356.98557. **IR** (ATR) ν (cm^{-1}) 3057, 2976, 1615, 1568, 1615, 1497, 1473, 1360, 1197, 1022. **Mp** (°C): 97–98. Analytical data is in accordance to those reported in literature.[18]

1-(2-Iodophenyl)-2-methyl-1*H*-indazol-2-ium triflate (4be)

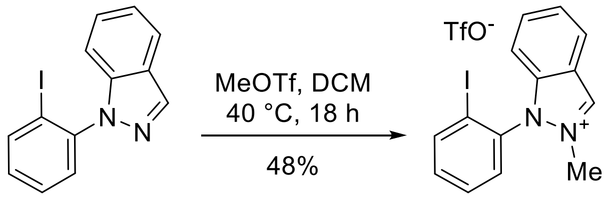

4bc **4be**

Following a literature procedure,[16] a solution of 1-(2-iodophenyl)-1*H*-indazol (**4bc**, 320 mg, 1.00 mmol) and MeOTf (170 µmol, 1.50 mmol) in dry DCM (5 mL) was stirred at 40 °C for 18 h in a pressure vial. The solvent was removed under reduced pressure, and Et_2O (2 mL), was added, which led to the formation of an oil. The Et_2O was removed by decantation, and the procedure was repeated two times with Et_2O (2 × 2 mL), so that 1-(2-iodophenyl)-2-methyl-1*H*-indazol-2-ium triflate (**4be**, 232 mg, 479 µmol, 48%) was obtained as a colorless oil.

^{1}H NMR (600 MHz, CD_3CN) δ (ppm) 8.96 (s, 1H), 8.22 (d, *J* = 8.1 Hz, 1H), 8.17 (d, *J* = 8.5 Hz, 1H), 8.02 (dd, *J* = 8.8, 7.0 Hz, 1H), 7.89 (d, *J* = 8.9 Hz, 1H), 7.84 – 7.74 (m, 2H), 7.64 (t, *J* = 7.7 Hz, 1H), 7.59 (ddd, *J* = 8.6, 5.2, 3.7 Hz, 1H), 3.95 (s, 3H). **^{13}C NMR** (151 MHz, CD_3CN) δ (ppm) 142.0, 141.7, 136.5, 136.1, 135.8, 135.2, 131.5, 131.2, 127.3, 124.4, 120.8, 112.4, 97.9, 35.2. **^{19}F NMR** (565 MHz, CD_3CN) δ (ppm) -79.31. **HRMS** (ESI) Calculated for $C_{14}H_{12}IN_2^+$ [M-TfO]$^+$ *m/z* 335.00397, found *m/z* 335.00338. **IR** (ATR) ν (cm^{-1}) 3085, 1631, 1528, 1251, 1151, 1026, 753.

General procedure for synthesis of substituted 1-(2-iodophenyl)-1*H*-benzo[*d*]imidazoles from *o*-fluoronitrobenzenes (GP2)

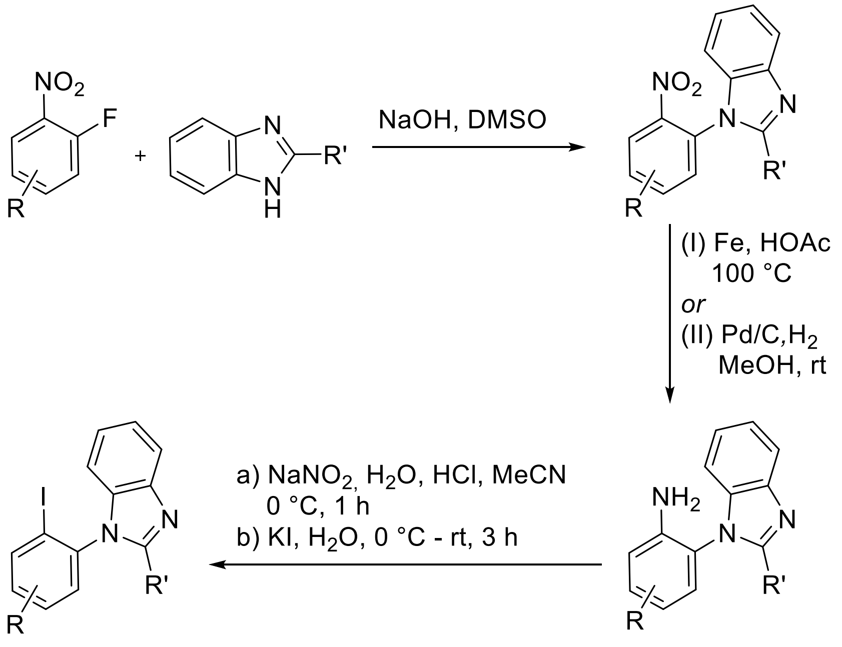

(GP2a) Following a modified literature procedure[19], powdered NaOH (3.00 equiv) was added slowly to a solution of the corresponding *o*-fluoronitrobenzene (1.00 equiv) and the corresponding benzimidazole (1.10 equiv) in DMSO, and the mixture was stirred for the indicated time and temperature. Water (10 mL/mmol) was added and the mixture was extracted with EtOAc (3 × 10 mL/mmol). The combined organic layers were dried over Na_2SO_4,

filtered, and the solvent was removed under reduced pressure. The residue was purified via column chromatography

(GP2bI) A modified literature procedure was used.[20] To a solution of the corresponding nitroarene (1.00 equiv) in HOAc (3 mL/mmol) and EtOH (3 mL/mmol) was added iron powder (3.00 equiv), and the mixture was stirred at 100 °C for the given time. After cooling to room temperature, sat. $NaHCO_3$ solution was added until neutralization, and the mixture was then extracted with EtOAc (3 × 70 mL/mmol). The combined organic layers were washed with brine (70 mL/mmol), dried over Na_2SO_4, filtered, and the solvent was removed under reduced pressure. The crude product was purified as described or used without further purification.

(GP2bII) Following a literature-known procedure,[21] the corresponding nitroarene (1.00 equiv) was dissolved in MeOH in a stainless steel autoclave, and Pd/C (10%, 0.100 equiv Pd) was added. The autoclave was closed, filled with H_2 (6.0 bar), and the reaction mixture was stirred at room temperature for the indicated time. The reaction mixture was filtered through Celite, washed with MeOH and the solvent was removed under reduced pressure to obtain the corresponding aniline, which was either used without further purification or further purified.

(GP2c) A modified literature procedure was used.[22] To a solution of the corresponding aniline (1.00 equiv) in MeCN (3 mL/mmol) and aq. HCl (6 M, 3 mL/mmol) at 0 °C was added a solution of $NaNO_2$ (1.15 equiv) in H_2O (1 mL/mmol) dropwise over 10 min. After 1 h at this temperature, a solution of KI (3.00 equiv) in H_2O (1 mL/mmol) was added dropwise. The solution was stirred for 1 h at 0 °C and 2 h at room temperature before H_2O (40 mL/mmol) was added and the mixture was extracted with EtOAc (3 × 40 mL/mmol). The combined organic phases were washed with sat. $Na_2S_2O_3$ solution (40 mL/mmol) and brine (40 mL/mmol), then dried over Na_2SO_4, filtered, and the solvent was removed under reduced pressure. The residue was purified on column chromatography.

1-(2-Iodo-4-(trifluoromethyl)phenyl)-1*H*-benzo[*d*]imidazole (4ag)

Following **GP2a** 1-fluoro-2-nitro-4-(trifluormethyl)benzene (**S10i**, 707 mg, 3.38 mmol), benzimidazole (836 mg, 7.08 mmol) and NaOH-powder (310 mg, 7.75 mmol) in DMSO (4 mL) gave 1-(2-nitro-4-(trifluoromethyl)phenyl)-1*H*-benzo[*d*]imidazole (**S4ag1**, 1.29 g, 4.20 mmol), which was used without further purification.

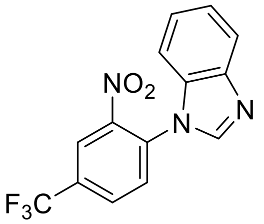

^{1}H NMR (601 MHz, $CDCl_3$) δ (ppm) 8.46 (d, *J* = 2.0 Hz, 1H), 8.12 (dd, *J* = 8.5, 2.3 Hz, 1H), 8.05 (s, 1H), 7.93 (dt, *J* = 8.1, 0.8 Hz, 1H), 7.80 (d, *J* = 8.2 Hz, 1H), 7.41 (ddd, *J* = 8.1, 7.3, 1.2 Hz, 1H), 7.36 (td, *J* = 7.8, 7.3, 1.1 Hz, 1H), 7.19 (dt, *J* = 8.0, 0.9 Hz, 1H). **^{13}C NMR** (151 MHz, $CDCl_3$) δ (ppm) 145.5, 143.6, 141.9, 133.9, 132.6, 132.2, 131.0, 130.4, 124.7, 123.8, 123.7, 121.2, 109.2. **^{19}F NMR** (565 MHz, $CDCl_3$) δ (ppm) -62.87. **HRMS** (ESI) Calculated for $C_{14}H_9F_3N_3O_2^+$ $[M+H]^+$ *m/z* 308.06414, found *m/z* 308.06381. **IR** (ATR) ν (cm^{-1}) 3081, 1609, 1537, 1495, 1455, 1320, 1141, 977, 715, 677. **Mp** (°C) 124.

Following **GP2bII** 1-(2-nitro-4-(trifluoromethyl)phenyl)-1*H*-benzo[*d*]imidazole (**S4ag1**, 1.29 g, 4.20 mmol) and Pd/C (447 mg, 4.20 mmol) and MeOH (40 mL) gave 2-(1*H*-benzo[*d*]imidazol-1-yl)-5-(trifluoromethyl)aniline (**S4ag2**, 1.13 g, 4.09 mmol, 97%) as a colorless solid, which was used without further purification. **HRMS** (ESI) Calculated for $C_{14}H_{11}F_3N_3^+$ $[M+H]^+$ *m/z* 278.08996, found *m/z* 278.08971.

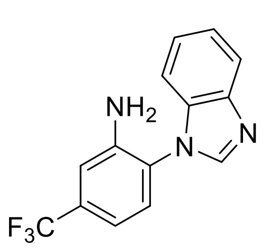

Following **GP2c** 2-(1*H*-benzo[*d*]imidazol-1-yl)-5-(trifluoromethyl)aniline (**S4ag2**, 1.11 g, 4.87 mmol), $NaNO_2$ (317 mg, 4.60 mmol) in water (4.6 mL), HCl (6 M, 12 mL), MeCN (3.0 mL) and KI (1.49 g, 9.00 mmol) in water (4.6 mL) gave 1-(2-iodo-4-(trifluoromethyl)phenyl)-1*H*-benzo[*d*]imidazole (**4ag**, 37.0 mg, 95.3 µmol, 2%) after column chromatography (Cy 2:1 EtOAc) as a colorless solid.

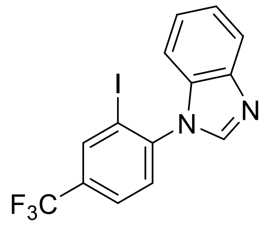

^{1}H NMR (601 MHz, $CDCl_3$) δ (ppm) 8.32 (d, *J* = 1.4 Hz, 1H), 8.28 (s, 1H), 7.94 (d, *J* = 7.8 Hz, 1H), 7.86 – 7.82 (m, 1H), 7.60 (d, *J* = 8.1 Hz, 1H), 7.43 – 7.39 (m, 1H), 7.37 (td, *J* = 7.7, 7.2, 1.1 Hz, 1H), 7.18 – 7.15 (m, 1H). **^{13}C NMR** (151 MHz, $CDCl_3$) δ (ppm) 145.5, 143.6, 141.9, 133.9, 132.6, 132.3 (q, *J* = 35.0 Hz), 131.0, 130.4, 124.7, 123.8, 123.7 (q, *J* = 3.5 Hz), 122.3 (q, *J* = 273.2 Hz), 121.2, 109.2. **^{19}F NMR** (565 MHz, $CDCl_3$) δ (ppm) -77.75. **HRMS** (ESI) Calculated for $C_{14}H_9F_3IN_2^+$ $[M+H]^+$ *m/z* 388.97571, found *m/z* 388.97556. **IR** (ATR) ν (cm^{-1}) 2341, 1678, 1575, 1552,1488, 1408, 1216, 1095, 1027, 704. **Mp** (°C) 232.

1-(2-Iodo-6-methylphenyl)-1*H*-benzo[*d*]imidazole (4al)

Following **GP2a** 2-fluoro-1-methyl-3-nitrobenzene (**S10k**, 1.55 g, 10.0 mmol), benzimidazole (1.30 g, 11.0 mmol) and NaOH powder (610 mg, 15.3 mmol) in DMSO (6 mL) gave 1-(2-methyl-6-nitrophenyl)-1*H*-benzo[*d*]imidazole (**S4al1**, 1.14 g, 4.50 mmol, 45%) after stirring for 5 h at room temperature and 2 h at 40 °C as a colorless solid.

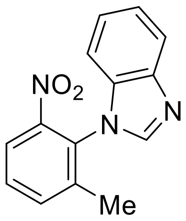

^{1}H NMR (600 MHz, $CDCl_3$) δ (ppm) 9.26 (s, 1H), 8.93 (d, *J* = 8.1 Hz, 1H), 8.74 (d, *J* = 7.7 Hz, 1H), 8.62 (d, *J* = 5.2 Hz, 2H), 8.13 (t, *J* = 7.5 Hz, 1H), 8.10 (t, *J* = 7.5 Hz, 1H), 7.91 (d, *J* = 7.7 Hz, 1H), 4.16 (s, 3H). **^{13}C NMR** (151 MHz, $CDCl_3$) δ (ppm) 152.6, 149.1, 148.1, 144.7, 141.4, 139.2, 135.8, 132.2, 129.0, 128.3, 127.7, 125.2, 115.0, 22.1. **HRMS** (ESI) Calculated for $C_{14}H_{12}N_3O_2^+$ $[M+H]^+$ *m/z* 254.09240, found *m/z* 254.09244. **IR** (ATR) ν (cm^{-1}) 3068, 2922, 1607, 1526, 1492, 1452, 1337, 1305, 1204, 1005, 713. **Mp** (°C) 113.

Following **GP2bI** 1-(2-methyl-6-nitrophenyl)-1*H*-benzo[*d*]imidazole (**S4al1**, 1.02 g, 4.00 mmol) and Fe powder (670 mg, 12.0 mmol) in HOAc (12 mL) and EtOH (12 mL) gave 2-(1*H*-benzo[*d*]imidazol-1-yl)-3-methylaniline (**S4al2**) as a colorless powder, which was used without further purification.

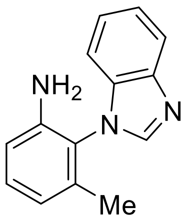

Following **GP2c** 2-(1*H*-benzo[*d*]imidazol-1-yl)-3-methylaniline (**S4al2**, 582 mg, 2.61 mmol), $NaNO_2$ (199 mg, 2.88 mmol) in water (2.9 mL), HCl (6 M, 7.5 mL), MeOH (7.5 mL) and KI (934 mg, 5.63 mmol) in water (2.9 mL) gave 1-(2-iodo-6-methylphenyl)-1*H*-benzo[*d*]imidazole (**4al**, 422 mg, 1.26 mmol, 48%) as a colorless solid after column chromatography (Cy 4:1 EtOAc).

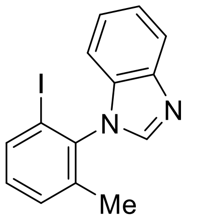

^{1}H NMR (600 MHz, $CDCl_3$) δ (ppm) 7.95 – 7.92 (m, 2H), 7.91 – 7.86 (m, 1H), 7.42 – 7.36 (m, 2H), 7.33 – 7.30 (m, 1H), 7.18 (t, *J* = 7.8 Hz, 1H), 7.05 (d, *J* = 8.1 Hz, 1H), 2.06 (s, 3H). **^{13}C NMR** (151 MHz, $CDCl_3$) δ (ppm) 143.3, 142.4, 138.6, 137.7, 133.4, 131.1, 129.7, 125.6, 123.8, 122.7, 120.6, 118.5, 110.3, 18.7. **HRMS** (ESI) Calculated for $C_{14}H_{12}IN_2^+$ $[M+H]^+$ *m/z* 335.00397, found *m/z* 335.00339. **IR** (ATR) ν (cm^{-1}) 3115, 1479, 1459, 1285, 1221, 1147, 861, 715. **Mp** (°C) 142.

1-(2-Iodophenyl)-2-phenyl-1*H*-benzo[*d*]imidazole (4an)

Following **GP2a** 2-fluoronitrobenzene (**S10l**, 0.76 mL, 7.20 mmol), 2-phenyl-1*H*-benzo[*d*]imidazole (**S2c**, 1.17 g, 6.00 mmol) and NaOH powder (360 mg, 9.00 mmol) in DMSO (6 mL) gave 1-(2-nitrophenyl)-2-phenyl-1*H*-benzo[*d*]imidazole (**S4an1**, 994 mg, 3.15 mmol, 53%) after 30 h reaction time at 100 °C and column chromatography (Cy 4:1 EtOAc) of the crude product.

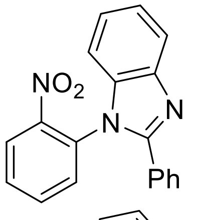

Following **GP2bI** 1-(2-nitrophenyl)-2-phenyl-1*H*-benzo[*d*]imidazole (**S4an1**, 788 mg, 2.50 mmol) and Fe powder (419 mg, 7.50 mmol) in HOAc (7.5 mL) and EtOH (7.5 mL) gave 2-(2-phenyl-1*H*-benzo[*d*]imidazol-1-yl)aniline (**S4an2**, 868 mg), which was used without further purification.

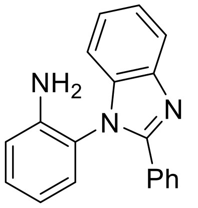

Following **GP2c** crude 2-(2-phenyl-1*H*-benzo[*d*]imidazol-1-yl)aniline (**S4an2**, 868 mg), $NaNO_2$ (198 mg, 2.88 mmol) in water (2.5 mL), HCl (6 M, 7.5 mL), MeCN (7.5 mL) and KI (934 mg, 5.63 mmol) in water (2.5 mL) gave 1-(2-iodophenyl)-2-phenyl-1*H*-benzo[*d*]imidazole (**4an**, 325 mg, 230 µmol, 33% over two steps) as a colorless solid after column chromatography (DCM 10:1 MeOH).

^{1}H NMR (601 MHz, $CDCl_3$) δ (ppm) 8.02 (dd, *J* = 8.0, 1.4 Hz, 1H), 7.91 (dd, *J* = 8.0, 0.9 Hz, 1H), 7.61 (dd, *J* = 7.3, 1.7 Hz, 2H), 7.50 (td, *J* = 7.6, 1.4 Hz, 1H), 7.31-7.38 (m, 3H), 7.33 – 7.25 (m, 3H), 7.23 (td, *J* = 7.7, 1.6 Hz, 1H), 7.00 (dt, *J* = 8.1, 0.9 Hz, 1H). **^{13}C NMR** (151 MHz, $CDCl_3$) δ (ppm) 152.3, 143.1, 140.6, 140.2, 136.8, 130.2, 130.1, 129.9, 129.7, 129.2, 128.5, 123.6, 123.2, 120.1, 111.0, 98.8 (one signal is overlapping). **HRMS** (ESI) Calculated for $C_{19}H_{14}IN_2^+$ $[M+H]^+$ *m/z* 397.01962, found *m/z* 397.01893. **IR** (ATR) *ν* (cm^{-1}) 3056, 1471, 1376, 1261, 1225, 762, 740, 690. **M_p** (°C) 152-154.

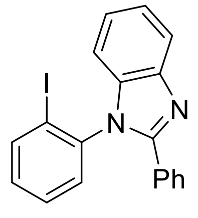

1-(2-Iodophenyl)-2-(trifluoromethyl)-1*H*-benzo[*d*]imidazole (4ao)

Following **GP2a** 2-fluoronitrobenzene (**S10l**, 1.14 mL, 10.8 mmol), 2-(trifluoromethyl)-1*H*-benzo[*d*]imidazole (**S2b**, 1.68 g, 9.00 mmol) and NaOH powder (540 mg, 13.5 mmol) in DMSO (9 mL) gave 1-(2-nitrophenyl)-2-(trifluoromethyl)-1*H*-benzo[*d*]imidazole (**S4ao1**, 1.46 g, 4.77 mmol, 53%) as a yellowish oil after 18 h reaction time at 100 °C and column chromatography (Cy 4:1 EtOAc) of the crude product.

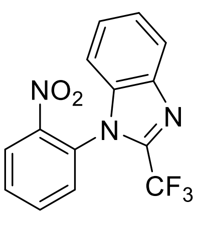

^{1}**H NMR** (601 MHz, $CDCl_3$) δ (ppm) 8.30 (dd, *J* = 8.2, 1.6 Hz, 1H), 7.95 (dt, *J* = 8.1, 1.1 Hz, 1H), 7.89 (td, *J* = 7.7, 1.5 Hz, 1H), 7.82 (ddd, *J* = 8.2, 7.6, 1.4 Hz, 1H), 7.60 (dd, *J* = 7.8, 1.4 Hz, 1H), 7.43 (ddd, *J* = 8.2, 7.2, 1.2 Hz, 1H), 7.38 (ddd, *J* = 8.3, 7.2, 1.2 Hz, 1H), 7.00 (dt, *J* = 8.0, 0.9 Hz, 1H). ^{13}**C NMR** (151 MHz, $CDCl_3$) δ (ppm) 145.9, 140.9, 140.5 (q, *J* = 39.0 Hz), 136.8, 134.7, 131.7, 131.4, 128.1, 126.5, 126.3, 124.5, 121.8, 118.7 (q, *J* = 272.0 Hz), 110.3. **HRMS** (ESI) Calculated for $C_{14}H_9F_3N_3O_3^+$ $[M+H]^+$ *m/z* 308.06414, found *m/z* 308.06382. **IR** (ATR) *ν* (cm^{-1}) 3058, 1607, 1529, 1345, 1210, 1131, 983, 852, 740.

Following **GP2bI** 1-(2-nitrophenyl)-2-(trifluoromethyl)-1*H*-benzo[*d*]imidazole (**S4ao1**, 1.24 g, 4.02 mmol) and Fe powder (670 mg, 12.1 mmol) in HOAc (12 mL) and EtOH (12 mL) gave 2-(2-(trifluoromethyl)-1*H*-benzo[*d*]imidazol-1-yl)aniline (**S4ao2**, 1.05 g, 4.02 mmol, 100%), which was used without further purification.

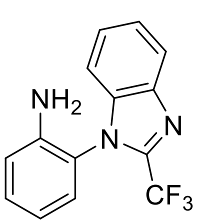

Following **GP2c** crude 2-(2-(trifluoromethyl)-1*H*-benzo[*d*]imidazol-1-yl)aniline (**S4ao2**, 832 mg, 3.00 mmol), $NaNO_2$ (238 mg, 3.45 mmol) in water (3 mL), HCl (6 M, 9 mL), MeCN (9 mL) and KI (1.12 g, 6.75 mmol) in water (3 mL) gave 1-(2-iodophenyl)-2-(trifluoromethyl)-1*H*-benzo[*d*]imidazole (**4ao**, 820 mg, 2.12 mmol, 76%) as a colorless solid after column chromatography (Cy 15:1 EtOAc).

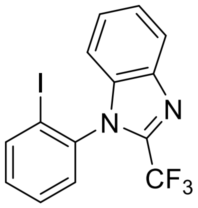

^{1}**H NMR** (600 MHz, $CDCl_3$) δ (ppm) 8.05 (dd, *J* = 8.0, 1.4 Hz, 1H), 7.96 (d, *J* = 7.6 Hz, 1H), 7.56 (td, *J* = 7.7, 1.4 Hz, 1H), 7.48 – 7.36 (m, 3H), 7.30 (td, *J* = 7.7, 1.6 Hz, 1H), 7.00 (dt, *J* = 7.9, 0.9 Hz, 1H). ^{13}**C NMR** (151 MHz, $CDCl_3$) δ (ppm) 140.8, 140.4 (q, *J* = 38.8 Hz), 140.4, 137.5, 136.2, 131.8, 129.7, 129.7, 126.2, 124.3, 121.7, 118.7 (q, *J* = 272.1 Hz), 111.6, 98.1. ^{19}**F NMR** (565 MHz, $CDCl_3$) δ (ppm) -61.47. **HRMS** (ESI) Calculated for $C_{14}H_8F_3IN_2Na^+$ $[M+Na]^+$ *m/z* 410.95765, found *m/z* 410.95696. **IR** (ATR) *ν* (cm^{-1}) 2988, 1528, 1473, 1415, 1261, 1169, 1131, 981, 740. **M$_p$** (°C) 134-135.

1-(2-Iodo-3-(1*H*-pyrazol-1-yl)phenyl)-1*H*-benzo[*d*]imidazole (4ba)

A pressure vial was filled with 1,3-difluoro-2-nitrobenzene (**S10h**, 9.94 g, 75.0 mmol), benzimidazole (**S2f**, 2.95 g, 25.0 mmol), K_3PO_4 (20.1 g, 100 mmol) and DMF (250 mL), sealed and stirred at 150 °C for 5 h.[11] H_2O (200 mL) was added, and the mixture was extracted with Et_2O (4 × 200 mL). The combined organic layers were washed with brine (100 mL), dried over Na_2SO_4, filtered, and the solvent was removed under reduced pressure. The residue was purified via column chromatography on silica (Cy 1:1 EtOAc) to obtain 1-(3-fluoro-2-nitrophenyl)-1*H*-benzo[*d*]imidazole (**S4ba1**, 1.59 g) as black oil, which was used without further purification.

Crude 1-(3-fluoro-2-nitrophenyl)-1*H*-benzo[*d*]imidazole (**S4ba1**, 1.33 g, 517 µmol) and pyrazole (689 mg, 10.1 mmol) were dissolved in DMSO (3.6 mL), and NaOH powder (405 mg, 10.1 mmol) was added. After 10 min stirring, ice water (40 mL) was added, the formed solid was filtered and dried in vacuo. The crude product was purified via column chromatography (Cy 1:1 EtOAc), so that 1-(2-nitrophenyl)-4-(1*H*-pyrazol-1-yl)-1*H*-benzo[*d*]imidazole (**S4ba2**, 878 mg, 2.88 mmol, 56%) was obtained as a colorless solid.

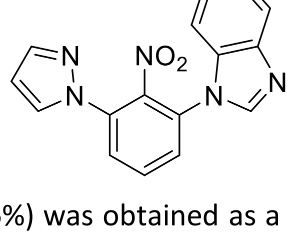

¹H NMR (601 MHz, $CDCl_3$) δ (ppm) 8.08 (s, 1H), 7.88 (dt, *J* = 7.9, 0.8 Hz, 1H), 7.84 (dd, *J* = 2.5, 0.6 Hz, 1H), 7.81 – 7.78 (m, 2H), 7.77 (dt, *J* = 1.9, 0.9 Hz, 1H), 7.53 (dd, *J* = 6.3, 2.9 Hz, 1H), 7.37 (ddd, *J* = 8.1, 7.2, 1.3 Hz, 1H), 7.33 (ddd, *J* = 8.3, 7.1, 1.3 Hz, 1H), 7.26 (d, *J* = 7.8 Hz, 1H), 6.54 (dd, *J* = 2.6, 1.8 Hz, 1H). **¹³C NMR** (151 MHz, $CDCl_3$) δ (ppm) 143.3, 142.8, 142.7, 142.5, 134.9, 133.8, 132.0, 129.8, 129.6, 127.8, 125.7, 124.7, 123.8, 120.8, 110.1, 109.3. **HRMS** (ESI) Calculated for $C_{16}H_{12}N_5O_2^+$ $[M+H]^+$ *m/z* 306.09855, found *m/z* 306.09848. **IR** (ATR) *ν* (cm^{-1}) 1599, 1538, 1495, 1393, 1208, 1042, 809, 739. **M_p** (°C) 174-176.

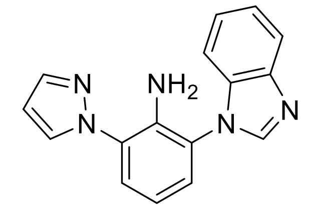

Following **GP2bII** 1-(2-nitrophenyl)-4-(1*H*-pyrazol-1-yl)-1*H*-benzo[*d*]imidazole (**S4ba2**, 611 mg, 2.00 mmol) and Pd/C (213 mg, 200 µmol) and MeOH (40 mL) gave 2-(1*H*-benzo[*d*]imidazol-1-yl)-6-(1*H*-pyrazol-1-yl)aniline (**S4ba3**, 561 mg, 2.00 mmol, 100%) as a colorless solid.

¹H NMR (601 MHz, $CDCl_3$) δ (ppm) 7.94 (s, 1H), 7.81 (dd, *J* = 7.8, 1.3 Hz, 1H), 7.77 (d, *J* = 2.5 Hz, 1H), 7.71 (d, *J* = 1.9 Hz, 1H), 7.32 (dd, *J* = 8.0, 1.5 Hz, 1H), 7.30 – 7.20 (m, 3H), 7.14 (dd, *J* = 7.8, 1.5 Hz, 1H), 6.84 (t, *J* = 7.9 Hz, 1H), 6.44 (t, *J* = 2.2 Hz, 1H), 4.90 (s, 2H). **¹³C NMR** (151 MHz, $CDCl_3$) δ (ppm) 143.4, 143.1, 141.0, 138.2, 133.7, 130.1, 127.4, 127.2, 124.6, 123.7, 122.8, 122.7, 120.5, 117.0, 110.8, 106.9. **HRMS** (ESI) Calculated for $C_{16}H_{14}N_5^+$ $[M+H]^+$ *m/z* 276.12437, found *m/z* 276.12418. **IR** (ATR) *ν* (cm^{-1}) 3410, 3123, 2988, 1630, 1488, 1395, 1223, 1029, 890, 747. **M_p** (°C) 134-136.

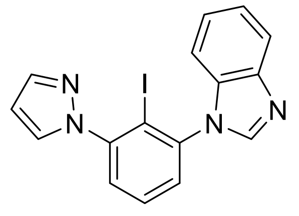

Following **GP2c** 2-(1*H*-benzo[*d*]imidazol-1-yl)-6-(1*H*-pyrazol-1-yl)aniline (**S4ba3**, 138 mg, 500 µmol), $NaNO_2$ (40.0 mg, 570 µmol) in water (0.5 mL), HCl (6 M, 3.0 mL), MeCN (3.0 mL) and KI (249 mg, 1.13 mmol) in water (0.5 mL) gave 1-(2-iodo-3-(1*H*-pyrazol-1-yl)phenyl)-1*H*-benzo[*d*]imidazole (**4ba**, 140 mg, 360 µmol, 72%) after column chromatography (Cy 1:1 EtOAc) as a colorless solid.

¹H NMR (601 MHz, $CDCl_3$) δ (ppm) 8.03 (s, 1H), 7.88 (dt, *J* = 8.0, 1.1 Hz, 1H), 7.77 (d, *J* = 2.2 Hz, 2H), 7.62 (t, *J* = 7.8 Hz, 1H), 7.56 (dd, *J* = 7.9, 1.6 Hz, 1H), 7.47 (dd, *J* = 7.7, 1.6 Hz, 1H), 7.34 (ddd, *J* = 8.1, 7.2, 1.3 Hz, 1H), 7.30 (ddd, *J* = 8.3, 7.1, 1.2 Hz, 1H), 7.17 (dt, *J* = 7.9, 0.9 Hz, 1H), 6.50 (t, *J* = 2.1 Hz, 1H). **¹³C NMR** (151 MHz, $CDCl_3$) δ (ppm) 145.9, 143.3, 142.7, 141.3, 140.6, 134.2, 131.2, 130.1, 129.1, 129.0, 124.0, 123.0, 120.7, 110.6, 107.2, 99.6. **HRMS** (ESI) Calculated for $C_{16}H_{12}IN_4^+$ $[M+H]^+$ *m/z* 387.01012, found *m/z* 387.01004. **IR** (ATR) ν (cm^{-1}) 2988, 2901, 1574, 1486, 1392, 1235, 1037, 749. **Mp** (°C) 133-134.

2-(4-(1*H*-Pyrazol-1-yl)-1*H*-benzo[*d*]imidazol-1-yl)aniline (S4aq1)

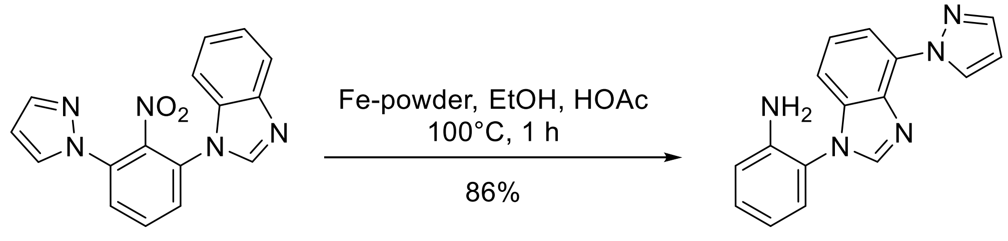

S4ba1 **S4aq1**

Following **GP2bI** 1-(2-nitrophenyl)-4-(1*H*-pyrazol-1-yl)-1*H*-benzo[*d*]imidazole (**S4ba1**, 906 mg, 2.97 mmol) and iron powder (496 mg, 8.90 mmol) were stirred in HOAc (9 mL) and EtOH

(9 mL) for 1 h at 100 °C, so that 2-(4-(1*H*-pyrazol-1-yl)-1*H*-benzo[*d*]imidazol-1-yl)aniline (**S4aq1**, 707 mg, 2.56 mmol, 86%) was isolated after column chromatography (Cy 3:1 EtOAc).

^{1}H NMR (601 MHz, $CDCl_3$) δ (ppm) 9.23 (dd, *J* = 2.5, 0.7 Hz, 1H), 8.05 (s, 1H), 7.97 (dd, *J* = 7.9, 1.0 Hz, 1H), 7.79 (d, *J* = 1.8 Hz, 1H), 7.37 (t, *J* = 8.0 Hz, 1H), 7.33 (ddd, *J* = 8.1, 7.5, 1.5 Hz, 1H), 7.20 (dd, *J* = 7.8, 1.5 Hz, 1H), 7.13 (dd, *J* = 8.1, 1.0 Hz, 1H), 6.92 (dd, *J* = 8.2, 1.3 Hz, 1H), 6.89 (td, *J* = 7.6, 1.3 Hz, 1H), 6.54 (dd, *J* = 2.4, 1.8 Hz, 1H), 3.65 (s, 2H). **^{13}C NMR** (151 MHz, $CDCl_3$) δ (ppm) 143.2, 142.9, 140.8, 135.8, 134.2, 131.8, 131.7, 130.7, 128.4, 124.4, 120.9, 119.0, 116.8, 113.6, 108.6, 107.3. **HRMS** (ESI) Calculated for $C_{16}H_{14}N_5^+$ $[M+H]^+$ *m/z* 276.12437, found *m/z* 276.12439. **IR** (ATR) *v* (cm^{-1}) 3324, 3208, 2988, 2901, 1592, 1503, 1395, 1203, 1044, 893, 743. **Mp** (°C) 150-151.

1-(2-Iodophenyl)-4-(1*H*-pyrazol-1-yl)-1*H*-benzo[*d*]imidazole (4aq)

Following **GP2c** 2-(4-(1*H*-pyrazol-1-yl)-1*H*-benzo[*d*]imidazol-1-yl)aniline (**S4aq1**, 240 mg, 870 µmol), $NaNO_2$ (69.5 mg, 1.00 mmol) in water (30.9 mL), HCl (6 M, 2.6 mL), MeCN (2.6 mL) and KI (433 mg, 2.61 mmol) in water (0.9 mL) gave 1-(2-iodophenyl)-4-(1*H*-pyrazol-1-yl)-1*H*-benzo[*d*]imidazole (**4aq**, 245 mg, 632 µmol, 73%) as a colorless solid after column chromatography (Cy 3:1 EtOAc).

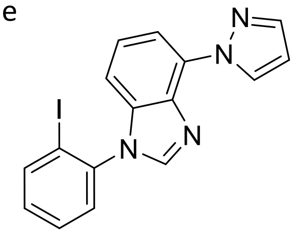

^{1}H NMR (601 MHz, $CDCl_3$) δ (ppm) 9.28 (dd, *J* = 2.5, 0.7 Hz, 1H), 8.07 (dd, *J* = 8.0, 1.4 Hz, 1H), 8.06 (s, 1H), 8.01 (dd, *J* = 7.9, 0.9 Hz, 1H), 7.80 (dd, *J* = 1.8, 0.7 Hz, 1H), 7.56 (td, *J* = 7.6, 1.4 Hz, 1H), 7.44 (dd, *J* = 7.8, 1.6 Hz, 1H), 7.38 (t, *J* = 8.0 Hz, 1H), 7.28 (ddd, *J* = 8.0, 7.5, 1.6 Hz, 1H), 7.02 (dd, *J* = 8.1, 1.0 Hz, 1H), 6.55 (dd, *J* = 2.5, 1.8 Hz, 1H). **^{13}C NMR** (151 MHz, $CDCl_3$) δ (ppm) 143.2, 142.9, 140.8, 135.8, 134.2, 131.8, 131.7, 130.7, 128.4, 124.4, 120.9, 119.0, 116.8, 113.6, 108.6, 107.3. **HRMS** (ESI) Calculated for $C_{16}H_{12}IN_4^+$ $[M+H]^+$ *m/z* 387.01012, found *m/z* 387.00986. **IR** (ATR) v (cm^{-1}) 3158, 3111, 3053, 1595, 1489, 1419, 1080, 893, 745. **Mp** (°C) 111-112.

1-(2-Iodo-3-(1*H*-pyrazol-1-yl)phenyl)-5,6-dimethyl-1*H*-benzo[*d*]imidazole (4bb)

Following modified **GP2a** 2,6-difluoronitrobenzene (**S10h**, 2.39 g, 15.0 mmol) was dissolved in DMSO (75 mL), and a solution of 5,6-dimethyl-1*H*-benzimidazole (**S2d**, 2.19 g, 15.0 mmol) and NaOH powder (600 mg, 15.0 mmol) in DMSO (75 mL) was added dropwise over 20 min. Afterward, NaOH powder (800 mg, 20.0 mmol) and pyrazole (1.36 g, 20.0 mmol) were added, and the reaction mixture was stirred for another 3 h. The solution was poured into ice water (800 mL) and stirred for 10 min. The suspension was filtered, and the filter cake was dried in vacuo. The solid was purified via column chromatography on silica (Cy 60:40 -> 40:60 EtOAc) to obtain 5,6-dimethyl-1-(2-nitro-3-(1*H*-pyrazol-1-yl)phenyl)-1*H*-benzo[*d*]imidazole (**S4bb1**, 2.40 g, 7.19 mmol, 48%) as a yellow solid.

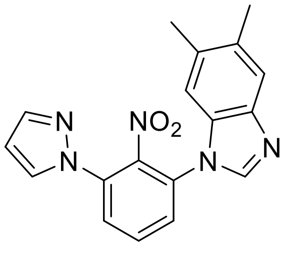

^{1}H NMR (600 MHz, $CDCl_3$) δ (ppm) 7.91 (s, 1H), 7.82 (d, *J* = 2.6 Hz, 1H), 7.80 – 7.74 (m, 3H), 7.61 (s, 1H), 7.50 (t, *J* = 4.6 Hz, 1H), 7.02 (s, 1H), 6.53 (dd, *J* = 2.6, 1.8 Hz, 1H), 2.38 (s, 3H), 2.33 (s, 3H). **^{13}C NMR** (151 MHz, $CDCl_3$) δ (ppm) 143.2, 142.5, 142.0, 141.7, 134.0, 133.8, 133.5, 132.7, 131.9, 130.3, 129.6, 127.9, 125.4, 120.8, 110.1, 109.2, 20.7, 20.4. **HRMS** (ESI) Calculated for $C_{18}H_{16}N_5O_2^+$ $[M+H]^+$ *m/z* 334.12985, found *m/z* 334.12891. **IR** (ATR) *v* (cm^{-1}) 3117, 2907, 1541, 1491, 1396, 1219, 842, 760. **M_p** (°C) 125-126.

Following **GP2bII** 5,6-dimethyl-1-(2-nitro-3-(1*H*-pyrazol-1-yl)phenyl)-1*H*-benzo[*d*]imidazole (**S4bb1**, 2.37 g, 7.10 mmol) and Pd/C (687 mg, 710 µmol) and MeOH (40 mL) gave 2-(5,6-dimethyl-1*H*-benzo[*d*]imidazol-1-yl)-6-(1*H*-pyrazol-1-yl)aniline (**S4bb2**, 1.80 g, 6.05 mmol, 85%) as an off-white solid.

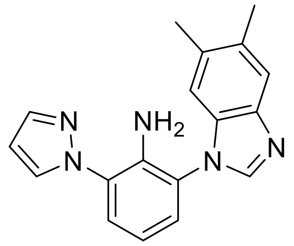

^{1}H NMR (601 MHz, $CDCl_3$) δ (ppm) 8.30 (s, 1H), 7.82 (d, *J* = 2.4 Hz, 1H), 7.77 (d, *J* = 1.8 Hz, 1H), 7.64 (s, 1H), 7.38 (d, *J* = 7.7 Hz, 1H), 7.20 (d, *J* = 7.4 Hz, 1H), 7.13 (s, 1H), 6.90 (t, *J* = 7.4 Hz, 1H), 6.50 (t, *J* = 2.0 Hz, 1H), 4.98 (s, 2H), 2.38 (s, 3H), 2.36 (s, 3H). **^{13}C NMR** (151 MHz, $CDCl_3$) δ (ppm) 141.2, 138.5, 134.5, 133.3, 130.3, 127.6, 127.6, 125.1, 122.4, 119.6, 117.2, 111.5, 107.1, 20.6, 20.4 (three signals are missing due to broad signals). **HRMS** (ESI) Calculated for $C_{18}H_{18}N_5^+$ $[M+H]^+$ *m/z* 304.15433, found *m/z* 304.15509. **IR** (ATR) *v* (cm^{-1}) 3435, 3301, 3101, 2918, 1622, 1588, 1493, 1393, 1225, 939, 772, 739. **M_p** (°C) 174-175.

Following **GP2c** 2-(5,6-dimethyl-1*H*-benzo[*d*]imidazol-1-yl)-6-(1*H*-pyrazol-1-yl)aniline (**S4bb2**, 1.79 g, 5.90 mmol), $NaNO_2$ (476 mg, 6.79 mmol) in water (5.4 mL), HCl (6 M, 32 mL), MeCN (32 mL) and KI (2.69 g, 13.3 mmol) in water (5.4 mL) gave 1-(2-iodo-3-(1*H*-pyrazol-1-yl)phenyl)-5,6-dimethyl-1*H*-benzo[*d*]imidazole (**4bb**, 1.86 g, 4.50 mmol, 76%) as a colorless solid after column chromatography (Cy 1:1 EtOAc).

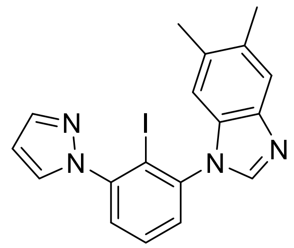

^{1}H NMR (600 MHz, $CDCl_3$) δ (ppm) 7.92 (s, 1H), 7.78 (s, 2H), 7.64 (s, 1H), 7.61 (td, *J* = 7.9, 1.8 Hz, 1H), 7.55 (d, *J* = 8.0 Hz, 1H), 7.46 (dd, *J* = 7.5, 2.0 Hz, 1H), 6.94 (s, 1H), 6.50 (d, *J* = 2.3 Hz, 1H), 2.39 (s, 3H), 2.34 (s, 3H). **^{13}C NMR** (151 MHz, $CDCl_3$) δ (ppm) 145.9, 142.0, 141.9, 141.3, 140.9, 133.4, 132.8, 132.0, 131.3, 130.0, 129.0, 128.9, 120.6, 110.7, 107.2, 99.7, 20.6, 20.3. **HRMS** (ESI) Calculated for $C_{18}H_{16}IN_4^+$ $[M+H]^+$ *m/z* 415.04142, found *m/z* 415.04048. **IR** (ATR) *v* (cm^{-1}) 3097, 2918, 1728, 1576, 1473, 1216, 987, 750, 714. **M_p** (°C) 72-73.

3 Oxidative cyclization reactions

Optimization of reaction conditions

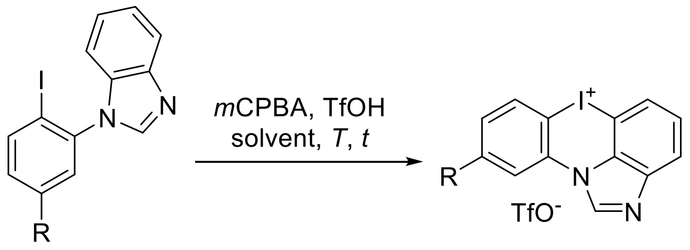

Entry	R	*m*CPBA [equiv]	TfOH [equiv]	*T* [°C]	*t* [d]	Solvent	Yield [%]
1	H	1.1	3.0	50	3	MeCN	0
2	H	1.1	3.0	50	3	DCE	23
3	H	1.1	2.5	50	3	DCE	69
4	H	1.1	2.0	50	3	DCE	19
**5**	**H**	**1.1**	**2.5**	**40**	**3**	**DCM**	**69**
6	H	1.1	2.5	rt	3	DCM	64
7	H	1.5	2.5	40	3	DCM	53
8	Cl	1.1	2.5	40	3	DCM	9[a]
9	Cl	1.1	2.5	80	3	DCM	35[a]
10	Cl	1.3	5.0	50	6	DCE	13[a]
11	Cl	1.3	5.0	65	14	DCE	25[a]
12	Cl	1.3	5.0	65	14	DCE:TFE	46
**13**	**Cl**	**1.3**	**5.0**	**65**	**14**	**DCM**	**52**
14	Cl	2.0	5.0	65	14	DCM	50

[a] incomplete conversion, product not clean.

General procedure for oxidative cyclization of (2-iodophenyl)-1*H*-benzo[*d*]imidazoles and -indazoles 5 (GP3a/b)

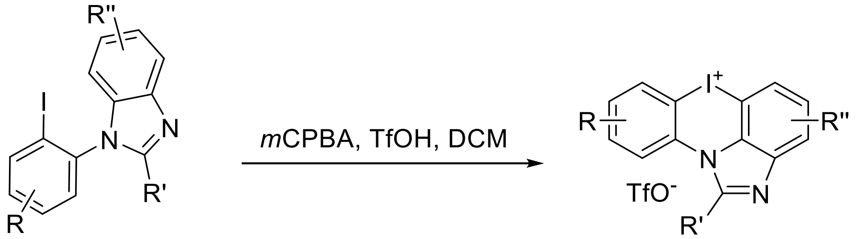

GP3a: To a stirred solution of the corresponding (2-iodophenyl)-1*H*-benzo[*d*]imidazole or -indazole (200 µmol, 1.00 equiv) and *m*CPBA (85%, 44.7 mg, 220 µmol, 1.10 equiv) in DCM (1 mL) was added TfOH (44.2 µL, 500 µmol, 2.50 equiv) and the solution was stirred for 72 h at 40 °C. The solvent was removed under reduced pressure, suspended in Et_2O (1 mL) or other solvents, if necessary, stored at 4 °C for 30 min, filtered, washed with Et_2O (3 × 1 mL), and dried in vacuo.

GP3b: To a stirred solution of the corresponding (2-iodophenyl)-1*H*-benzo[*d*]imidazole or -indazole (200 µmol, 1.00 equiv) and *m*CPBA (85%, 52.8 mg, 260 µmol, 1.30 equiv) in DCM (1 mL) was added TfOH (88.4 µL, 1.00 mmol, 5.00 equiv) and the solution was stirred for 14 d at 65 °C. The solvent was removed under reduced pressure, suspended in Et_2O (1 mL) or other solvents, if necessary, stored at 4 °C for 30 min, filtered, washed with Et_2O (3 × 1 mL), and dried in vacuo.

6*H*-6λ³-Ioda-2,10b-diazaaceanthrylen-6-yl triflate (5aa)

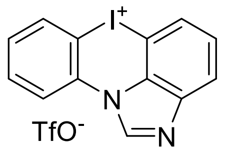

Following **GP3a** 1-(2-iodophenyl)-1*H*-benzo[*d*]imidazole (**4aa**, 64.0 mg, 200 µmol), *m*CPBA (44.9 mg, 220 µmol) and TfOH (44.2 µL, 500 µmol) in DCM (1 mL) gave 6*H*-6λ³-ioda-2,10b-diazaaceanthrylen-6-yl triflate (**5aa**, 64.7 mg, 138 µmol, 69%) as a colorless powder.

Gram-scale reaction

To a solution of 1-(2-iodophenyl)-1*H*-benzo[*d*]imidazole (**4aa**, 1.92 g, 6.00 mmol) and *m*CPBA (1.35 g, 6.60 mmol) in DCM (15 mL) was added TfOH (1.32 mL, 15.0 mmol) dropwise and stirred for 72 h at 50 °C. The solvent was removed by decantation, the residue was suspended in Et_2O (10 mL) and stored for 1 h at 4 °C. The suspension was filtered, washed with Et_2O (3 × 3 mL), and dried in vacuo, so that 6*H*-6λ³-ioda-2,10b-diazaaceanthrylen-6-yl triflate (**5aa**, 2.15 g, 4.58 mmol, 76%) was obtained as a colorless powder. For transformation reactions, the salt was recrystallized from water to obtain yellowish needles.

^{1}H NMR (601 MHz, DMSO-d_6) δ (ppm) 9.44 (s, 1H), 8.33 (dd, J = 8.4, 1.4 Hz, 1H), 8.09 (dd, J = 8.2, 1.4 Hz, 1H), 7.92 (dd, J = 8.0, 0.7 Hz, 1H), 7.83 (dd, J = 8.0, 0.8 Hz, 1H), 7.66 (ddd, J = 8.4, 7.3, 1.4 Hz, 1H), 7.52 (t, J = 8.0 Hz, 1H), 7.45 (ddd, J = 8.4, 7.3, 1.3 Hz, 1H). **^{13}C NMR** (151 MHz, DMSO-d_6) δ (ppm) 144.2, 141.3, 133.8, 133.0, 132.4, 128.9, 127.3, 127.0, 125.1, 122.3, 120.7 (q, J = 322.5 Hz), 119.2, 98.0, 86.0. **^{19}F NMR** (565 MHz, DMSO-d_6) δ (ppm) -77.86 ppm. **HRMS** (ESI) Calculated for $C_{13}H_{10}IN_2^+$ $[M-OTf+2H]^+$ *m/z* 320.98832, found *m/z* 320.98826 (reduction of the iodine in the mass spectrometer). **IR** (ATR) v (cm^{-1}) 3132, 3052, 3029, 2987, 2950, 2771, 2733, 2694, 1607, 155, 1509, 1467, 1435, 1329, 1278, 1217, 1157, 1022, 976, 905, 852, 779, 761, 732. **M_P** (°C) 293 (decom.).

7-Methyl-6*H*-6Λ³-ioda-2,10b-diazaaceanthrylen-6-ium triflate (5ab)

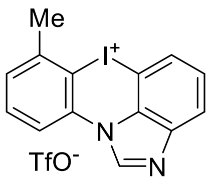

Following larger scaled **GP3a** 1-(2-iodo-3-methylphenyl)-1*H*-benzo[*d*]imidazole (**4ab**, 66.8 mg, 200 µmol), *m*CPBA (61.2 mg, 300 µmol) and TfOH (44.2 µL, 500 µmol) in DCM (1 mL) gave 7-methyl-6*H*-6Λ³-ioda-2,10b-diazaaceanthrylen-6-ium triflate (**5ab**, 18.0 mg, 37.3 µmol, 19%) as a colorless solid after 45 h reaction time.

^{1}H NMR (601 MHz, DMSO-d_6) δ (ppm) 9.46 (s, 1H), 8.13 (d, J = 8.1 Hz, 1H), 7.99 (d, J = 8.1 Hz, 1H), 7.87 (d, J = 8.0 Hz, 1H), 7.58 (td, J = 7.9, 2.3 Hz, 2H), 7.43 (d, J = 7.5 Hz, 1H), 2.69 (s, 3H). **^{13}C NMR** (151 MHz, DMSO-d_6) δ (ppm) 144.0, 142.0, 141.1, 133.2, 132.3, 130.1, 127.8, 127.5, 125.4, 122.6, 120.7 (q, J = 322.2 Hz), 116.7, 102.5, 85.0, 25.0. **^{19}F NMR** (565 MHz, DMSO-d_6) δ (ppm) -77.87. **HRMS** (ESI) Calculated for $C_{14}H_{12}IN_2^+$ [M-

OTf+2H]+ m/z 335.00397, found m/z 335.00384 (reduction of the iodine in the mass spectrometer). **IR** (ATR) v (cm^{-1}) 3112, 3035, 1690, 194, 1542, 1226, 1022, 808. **Mp** (°C) 223.

7-Fluoro-6*H*-6λ^3-ioda-2,10b-diazaaceanthrylen-6-ium triflate (5ac)

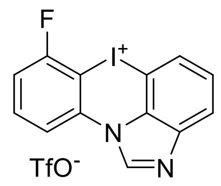

Following **GP3b** 1-(3-fluoro-2-iodophenyl)-1*H*-benzo[*d*]imidazole (**4ac**, 67.6 mg, 200 µmol), *m*CPBA (52.8 mg, 260 µmol) and TfOH (88.4 µL, 1.00 mol) in DCM (1 mL) gave 7-fluoro-6*H*-6λ^3-ioda-2,10b-diazaaceanthrylen-6-ium triflate (**5ac**, 53.1 mg, 109 mmol, 55%) as a colorless powder after 14 d reaction time.

^{1}H NMR (601 MHz, CD_3OD) δ (ppm) 9.43 (s, 1H), 8.10 (dt, J = 8.4, 0.9 Hz, 1H), 7.98 (d, J = 8.2 Hz, 1H), 7.89 (dd, J = 8.0, 0.7 Hz, 1H), 7.78 (td, J = 8.4, 6.6 Hz, 1H), 7.65 (t, J = 8.1 Hz, 1H), 7.43 (td, J = 8.3, 1.1 Hz, 1H). **^{13}C NMR** (151 MHz, CD_3OD) δ (ppm) 161.5 (d, J = 246.3 Hz), 144.9, 142.7, 136.3 (d, J = 9.4 Hz), 135.3 (d, J = 5.0 Hz), 129.6, 128.6, 126.9, 123.9, 121.8 (q, J = 318.2 Hz), 116.1 (d, J = 2.4 Hz), 115.9 (d, J = 21.9 Hz), 86.2 (d, J = 30.4 Hz), 84.7. **^{19}F NMR** (565 MHz, CD_3OD) δ (ppm) -80.13, -92.99 (dd, J = 8.2, 6.7 Hz). **HRMS** (ESI) Calculated for $C_{13}H_7FIN_2^+$ [M-TfO]+ m/z 336.96325, found m/z 336.96272. **IR** (ATR) v (cm^{-1}) 3122, 2988, 2901, 1551, 1474, 1432, 1223, 1162, 1021, 775. **Mp** (°C) 268-269 (decom.).

8-Methyl-6*H*-6λ^3-ioda-2,10b-diazaaceanthrylen-6-ium triflate (5af)

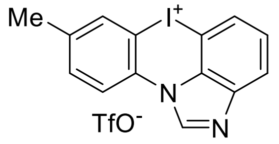

Following **GP3a** 1-(2-iodo-4-methylphenyl)-1*H*-benzo[*d*]imidazole (**4af**, 66.8 mg, 200 µmol), *m*CPBA (61.2 mg, 300 µmol) and TfOH (44.2 µL, 500 µmol) in DCM (1 mL) gave 8-methyl-6*H*-6λ^3-ioda-2,10b-diazaaceanthrylen-6-ium triflate (**5af**, 58.0 mg, 120 µmol, 60%) as a colorless powder after 72 h reaction time.

^{1}H NMR (601 MHz, DMSO-d_6) δ (ppm) 9.39 (s, 1H), 8.22 (d, J = 8.4 Hz, 1H), 7.93 (d, J = 8.1 Hz, 1H), 7.91 (s, 1H), 7.81 (d, J = 8.0 Hz, 1H), 7.51 (t, J = 8.1 Hz, 1H), 7.48 (d, J = 8.1 Hz, 1H), 2.37 (s, 3H). **^{13}C NMR** (151 MHz, DMSO-d_6) δ (ppm) 144.1, 141.2, 138.7, 133.5, 133.4, 130.1, 127.1, 126.9, 125.1, 122.3, 120.7 (q, J = 322.3 Hz), 118.8, 97.8, 86.0, 20.1. **^{19}F NMR** (565 MHz, DMSO-d_6) δ (ppm) -77.87. **HRMS** (ESI) Calculated for $C_{14}H_{12}IN_2^+$ [M-OTf+2H]+ m/z 335.00397, found m/z 335.00375 (reduction of the iodine in the mass spectrometer). **IR** (ATR) v (cm^{-1}) 3113, 3063, 3022, 1545, 1274, 1219, 1156, 1023, 810, 785, 736. **Mp** (°C) 288-289 °C (decom.).

8-(Trifluoromethyl)-6*H*-6λ^3-ioda-2,10b-diazaaceanthrylen-6-ium triflate (5ag)

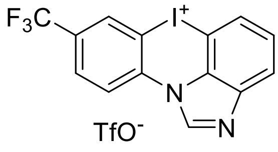

Following a modified procedure **GP3b** 1-(2-iodo-4-(trifluoromethyl)phenyl)-1*H*-benzo[*d*]imidazole (**4ag**, 35.1 mg, 90.4 µmol), *m*CPBA (24.0 mg, 118 µmol) and TfOH (39.9 µL, 452 µmol) in DCM (1 mL) gave 8-(trifluoromethyl)-6*H*-6λ^3-ioda-

2,10b-diazaaceanthrylen-6-ium triflate (**5ag**, 18.7 mg, 34.9 µmol, 39%) as a colorless powder after 14 d reaction time at 40 °C.

^{1}H NMR (601 MHz, CD_3OD) δ (ppm) 9.52 (s, 1H), 8.49 – 8.40 (m, 2H), 8.04 (d, *J* = 8.3 Hz, 1H), 7.96 (d, *J* = 8.0 Hz, 1H), 7.90 (d, *J* = 8.0 Hz, 1H), 7.67 (t, *J* = 8.1 Hz, 1H). **^{13}C NMR** (151 MHz, CD_3OD) δ (ppm) 143.0, 141.3, 135.4, 130.7, 130.2, 128.2, 127.2, 125.6, 122.1, 121.4 (q, *J* = 243.3 Hz), 119.7, 119.3, 96.5, 84.4 (the triflate carbon is not visible). **^{19}F NMR** (565 MHz, CD_3OD) δ (ppm) -64.42 (3F), -80.13 (3F). **HRMS** (ESI) Calculated for $C_{14}H_9F_3IN_2^+$ [M+2H-OTf]$^+$ *m/z* 388.97571, found *m/z* 388.97488 (reduction of the iodine in the mass spectrometer). **IR** (ATR) ν (cm^{-1}) 1278, 1224, 1170, 1134, 1090, 1066, 1027. **Mp** (°C) 285 (Decom.).

9-Chloro-6*H*-6λ^3-ioda-2,10b-diazaaceanthrylen-6-ium triflate (5ah)

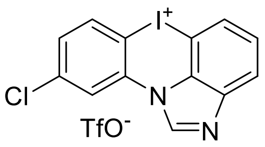

Following modified **GP3b** 1-(5-chloro-2-iodophenyl)-1*H*-benzo[*d*]imidazole (**4ah**, 70.7 mg, 200 µmol), *m*CPBA (55.9 mg, 324 µmol) and TfOH (88.4 µL, 1.00 mmol) in DCM (1 mL) were stirred at 65 °C for 14 d, so that 9-chloro-6*H*-6λ^3-ioda-2,10b-diazaaceanthrylen-6-ium triflate (**5ah**, 51.8 mg, 103 µmol, 52%) as a colorless powder.

^{1}H NMR (601 MHz, DMSO-d_6) δ (ppm) 9.45 (s, 1H), 8.51 (s, 1H), 8.09 (d, *J* = 8.7 Hz, 1H), 7.92 (d, *J* = 7.9 Hz, 1H), 7.82 (d, *J* = 7.9 Hz, 1H), 7.61 – 7.49 (m, 2H). **^{13}C NMR** (151 MHz, CD_3OD) δ (ppm) 144.6, 142.0, 138.1, 135.3, 134.1, 128.8, 127.7, 125.7, 122.2, 121.7 121.1 (q, *J* = 322.4 Hz), 119.2, 96.8, 86.6. **^{19}F NMR** (565 MHz, DMSO-d_6) δ (ppm) -77.75. **HRMS** (ESI) Calculated for $C_{13}H_9ClIN_2$ [M+2H-OTf]$^+$ *m/z* 354.94935, found *m/z* 354.94873 (reduction of the iodine in the mass spectrometer). **IR** (ATR) ν (cm^{-1}) 3130, 3063, 1584, 1494, 1254, 1223, 1164, 1027, 994, 775, 758, 729. **Mp** (°C) 319 (decom.).

9-Bromo-6*H*-6λ^3-ioda-2,10b-diazaaceanthrylen-6-ium triflate (5ai)

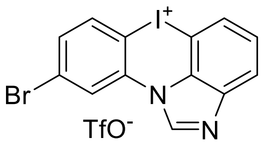

Following modified **GP3a** 1-(5-bromo-2-iodophenyl)-1*H*-benzo[*d*]imidazole (**4ai**, 79.8 mg, 200 µmol), *m*CPBA (55.9 mg, 220 µmol) and TfOH (44.2 µL, 500 µmol) in DCM (2 mL) were stirred at 80 °C for 6 d in a pressure vial and purified via column chromatography on silica (DCM -> DCM 10:1 MeOH) to obtain 9-bromo-6*H*-6λ^3-ioda-2,10b-diazaaceanthrylen-6-ium triflate (**5ai**, 53.6 mg, 98.0 µmol, 49%) as a colorless powder.

^{1}H NMR (601 MHz, CD_3OD) δ (ppm) 9.70 (s, 1H), 8.50 (d, *J* = 2.1 Hz, 1H), 7.94 (dd, *J* = 8.1, 0.7 Hz, 1H), 7.91 (d, *J* = 8.7 Hz, 1H), 7.81 (dd, *J* = 8.2, 0.7 Hz, 1H), 7.64 (dd, *J* = 8.7, 2.1 Hz, 1H), 7.61 (t, *J* = 8.1 Hz, 1H). **^{13}C NMR** (151 MHz, CD_3OD) δ (ppm) 142.4, 141.4, 135.9, 134.1, 133.9, 130.1, 128.6, 128.0, 127.7, 124.1, 122.2, 121.7 (q, *J* = 318.6 Hz), 96.0, 86.3. **^{19}F NMR** (565 MHz, CD_3OD) δ (ppm) -80.03. **HRMS** (ESI) Calculated for $C_{13}H_7BrIN_2^+$ [M-OTf]$^+$ *m/z* 396.88318, found *m/z* 396.88230. **IR** (ATR) ν (cm^{-1}) 3119, 2988, 2901, 1574, 1548, 1478, 1217, 1161, 1020, 780, 732. **Mp** (°C) 302-304 (decom.).

9-Cyano-6*H*-6λ³-ioda-2,10b-diazaaceanthrylen-6-ium triflate (5aj)

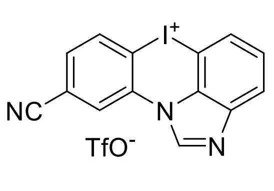

Following **GP3a** 3-(1*H*-benzo[*d*]imidazol-1-yl)-4-iodobenzonitrile (**4aj**, 69.0 mg, 200 µmol), *m*CPBA (44.9 mg, 220 µmol) and TfOH (44.2 µL, 500 µmol) in DCM (1 mL) gave 9-cyano-6*H*-6λ³-ioda-2,10b-diazaaceanthrylen-6-ium triflate (**5aj**, 45.4 mg, 92.1 µmol, 46%) as colourless powder after 72 h reaction time and suspending in Et_2O 1:1 DCM.

^{1}H NMR (601 MHz, DMSO-d_6) δ (ppm) 9.40 (s, 1H), 8.82 (s, 1H), 8.28 – 8.23 (m, 1H), 7.95 – 7.87 (m, 2H), 7.82 (d, *J* = 8.0 Hz, 1H), 7.53 (t, *J* = 8.0 Hz, 1H). **^{13}C NMR** (151 MHz, DMSO-d_6) δ (ppm) 144.6, 141.9, 135.2, 133.9, 131.7, 127.9, 127.6, 125.7, 122.9, 122.4, 117.3, 115.9, 104.9, 87.0 (triflate carbon not visible) **^{19}F NMR** (565 MHz, DMSO-d_6) δ (ppm) -77.74. **HRMS** (ESI) Calculated for $C_{14}H_9IN_3$ [M+2H-OTf]$^+$ *m/z* 345.98357, found *m/z* 345.98292 (reduction of the iodine in the mass spectrometer). **IR** (ATR) ν (cm^{-1}) 3125, 2237, 1558, 1496, 1478, 1240, 1156, 1024, 943, 793, 739. **Mp** (°C) 205 (decom.)

9-Nitro-6*H*-6λ³-ioda-2,10b-diazaaceanthrylen-6-ium triflate (5ak)

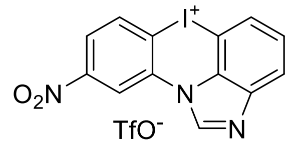

Following **GP3b** 1-(2-iodo-5-nitrophenyl)-1*H*-benzo[*d*]imidazole (**4ak**, 73.1 mg, 200 µmol), *m*CPBA (53.1 mg, 250 µmol) and TfOH (120 µL, 1.36 mmol) in DCM (1 mL) gave 9-nitro-6*H*-6λ³-ioda-2,10b-diazaaceanthrylen-6-ium triflate (**5ak**, 70.5 mg, 137 µmol, 69%) as a colorless powder after 14 d reaction time.

^{1}H NMR (601 MHz, CD_3OD) δ (ppm) 9.79 (s, 1H), 9.11 (s, 1H), 8.37 (d, *J* = 8.9 Hz, 1H), 8.32 (dd, *J* = 8.9, 1.9 Hz, 1H), 8.00 (d, *J* = 8.1 Hz, 1H), 7.92 (d, *J* = 8.1 Hz, 1H), 7.70 (t, *J* = 8.1 Hz, 1H). **^{13}C NMR** (151 MHz, CD_3OD) δ (ppm) 151.2, 142.0, 141.6, 134.7, 133.4, 128.5, 127.1, 125.8, 122.7, 121.8, 121.4 (q, *J* = 199.1 Hz), 113.9, 102.7, 84.7. **^{19}F NMR** (565 MHz, CD_3OD) δ (ppm) -80.12. **HRMS** (ESI) Calculated for $C_{13}H_9IN_3O_2^+$ [M+2H-OTf]$^+$ *m/z* 365.97340, found *m/z* 365.97289 (reduction of the iodine in the mass spectrometer). **IR** (ATR) ν (cm^{-1}) 3129, 1541, 1464, 1358, 1286, 1220, 1176, 1025, 739. **Mp** (°C) 299 (decom.).

10-Methyl-6*H*-6λ³-ioda-2,10b-diazaaceanthrylen-6-ium triflate (5al)

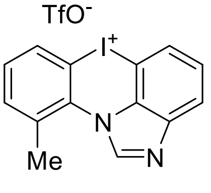

Following **GP3b** 1-(2-iodo-6-methylphenyl)-1*H*-benzo[*d*]imidazole (**4al**, 66.7 mg, 200 µmol), *m*CPBA (53.1 mg, 260 µmol) and TfOH (88.2 µL, 1.00 mmol) in DCM (1 mL) gave 10-methyl-6*H*-6λ³-ioda-2,10b-diazaaceanthrylen-6-ium triflate (**5al**, 72.6 mg, 151 µmol, 75%) as a colorless powder after 7 d reaction time.

^{1}H NMR (600 MHz, CD_3OD) δ (ppm) 9.72 (s, 1H), 8.20 – 8.00 (m, 2H), 7.96 (d, *J* = 8.1 Hz, 1H), 7.75 (t, *J* = 8.1 Hz, 1H), 7.71 (d, *J* = 7.7 Hz, 1H), 7.47 (t, *J* = 7.8 Hz, 1H), 2.84 (s, 3H). **^{13}C NMR** (151 MHz, CD_3OD) δ (ppm) 145.1, 139.7, 137.7, 134.4, 133.9, 132.0, 131.4, 131.3, 131.0, 128.3, 121.7 (q, *J* = 318.4 Hz), 120.7, 100.3, 87.2, 24.5. **^{19}F NMR** (565 MHz, CD_3OD) δ (ppm) -80.09. **HRMS** (ESI) Calculated for $C_{14}H_{10}IN_2^+$ [M-OTf]$^+$ *m/z* 332.98832, found *m/z* 332.98825. **IR** (ATR) ν (cm^{-1}) 3167, 1541, 1426, 1273, 1218, 1155, 1117, 1020, 771, 702. **Mp** (°C) 142.

1-Bromo-6*H*-6λ^3-ioda-2,10b-diazaaceanthrylen-6-ium triflate (5am)

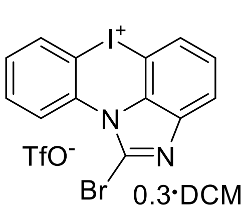

Following **GP3a** 2-bromo-1-(2-iodophenyl)-1*H*-benzo[*d*]imidazole (**4am** 79.8 mg, 200 µmol), *m*CPBA (44.9 mg, 210 µmol) and TfOH (44.2 µL, 500 µmol) in DCM (1 mL) gave 1-bromo-6*H*-6λ^3-ioda-2,10b-diazaaceanthrylen-6-ium triflate (**5am**, 97.1 mg, 170 µmol, 85%) as a colorless solid with 0.3 equiv of DCM after filtration of cold reaction suspension, further washing with DCM (2 × 1 mL), and drying in vacuo.

^{1}H NMR (601 MHz, CD_3CN) δ (ppm) 8.77 (dd, *J* = 8.1, 1.4 Hz, 1H), 8.15 (td, *J* = 7.8, 1.4 Hz, 1H), 8.03 (td, *J* = 7.9, 1.6 Hz, 1H), 8.00 (dt, *J* = 8.4, 0.9 Hz, 1H), 7.96 (dd, *J* = 7.9, 1.5 Hz, 1H), 7.76 (ddd, *J* = 8.4, 7.4, 1.0 Hz, 1H), 7.40 (dt, *J* = 8.5, 0.9 Hz, 1H), 5.45 (s, 0.6H, DCM). **^{13}C NMR** (151 MHz, CD_3CN) δ (ppm) 141.1, 137.9, 136.2, 135.0, 134.0, 132.6, 132.5, 132.2, 129.3, 129.1, 122.8, 121.3 (q, *J* = 319.0 Hz), 115.6, 114.3, 55.3 (DCM). **^{19}F NMR** (565 MHz, CD_3CN) δ (ppm) -79.22. **HRMS** (ESI) Calculated for $C_{13}H_7BrIN_2^+$ [M-TfO]$^+$ *m/z* 396.88138, found *m/z* 396.88276. **IR** (ATR) ν (cm^{-1}) 3070, 1715, 1490, 1221, 1169, 1055, 1023, 750. **Mp** (°C) 85-86 (decom.)

1-Phenyl-6*H*-6λ^3-ioda-2,10b-diazaaceanthrylen-6-ium triflate (5an)

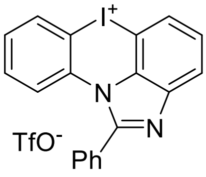

Following **GP3a** 1-(2-iodophenyl)-2-phenyl-1*H*-benzo[*d*]imidazole (**4an** 79.2 mg, 200 µmol), *m*CPBA (44.9 mg, 210 µmol) and TfOH (44.2 µL, 500 µmol) in DCM (1 mL) gave 1-phenyl-6*H*-6λ^3-ioda-2,10b-diazaaceanthrylen-6-ium triflate (**5an**, 59.8 mg, 110 µmol, 55%) as a colorless solid after suspending in Et_2O (1 mL) and a few drops of EtOAc.

^{1}H NMR (600 MHz, DMSO-d_6) δ (ppm) 8.21 – 8.08 (m, 1H), 8.02 – 7.92 (m, 3H), 7.90 (d, *J* = 8.0 Hz, 1H), 7.70 – 7.59 (m, 4H), 7.40 (tq, *J* = 7.3, 3.7, 2.2 Hz, 2H), 7.11 – 7.04 (m, 1H). **^{13}C NMR** (151 MHz, DMSO-d_6) δ (ppm) 154.6, 142.6, 135.6, 134.9, 134.1, 132.2, 131.4, 130.1, 129.4, 128.7, 128.0, 126.0, 123.0, 121.8, 121.8, 120.2 (q, *J* = 322.2 Hz), 100.9, 88.2. **^{19}F NMR** (565 MHz, DMSO-d_6) δ (ppm) -77.73. **HRMS** (ESI) Calculated for $C_{19}H_{12}IN_2^+$ [M-TfO]$^+$ *m/z* 395.00397, found *m/z* 395.00369. **IR** (ATR) ν (cm^{-1}) 2988, 1901, 1475, 1221, 1155, 1023, 759, 693. **Mp** (°C) 256-257 (decom.).

1-(Trifluoromethyl)-6*H*-6λ^3-ioda-2,10b-diazaaceanthrylen-6-ium triflate (5ao)

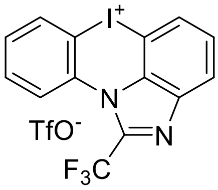

Following **GP3b** 2-(trifluoromethyl)-1-(2-iodphenyl)-1*H*-benzo[*d*]imidazole (**4ao**, 77.6 mg, 200 µmol), *m*CPBA (62.8 mg, 260 µmol) and TfOH (88.4 µL, 1.00 mol) were stirred in DCM (1 mL). The solvent was removed under reduced pressure, the residue was suspended in Et_2O (1 mL), filtered, washed with additional Et_2O (3 × 1 mL), and dried in vacuo, so that 1-(trifluoromethyl)-6*H*-6λ^3-ioda-2,10b-diazaaceanthrylen-6-ium triflate (**5ao**, 53.9 mg, 101 µmol, 50%) was obtained as a gray solid.

^{1}H NMR (601 MHz, DMSO-d_6) δ (ppm) 8.30 (dd, *J* = 8.2, 1.5 Hz, 1H), 8.18 (d, *J* = 8.1 Hz, 1H), 8.16 (d, *J* = 7.9 Hz, 1H), 7.90 (t, *J* = 7.4 Hz, 1H), 7.82 (t, *J* = 8.0 Hz, 1H), 7.77 (d, *J* = 8.3 Hz, 1H), 7.70 (t, *J* = 7.7 Hz, 1H). **^{13}C NMR** (151 MHz, DMSO-d_6) δ 140.7, 140.5 (q, *J* = 39.7 Hz), 135.8, 133.4, 133.3, 132.7, 130.0, 128.9, 128.7, 123.3, 122.9 (q, *J* = 5.1 Hz), 120.7 (q, *J* = 322.4 Hz),

118.7 (q, *J* = 272.2 Hz), 101.0, 88.7. **^{19}F NMR** (565 MHz, DMSO-d_6) δ (ppm) -57.84 (s, 3F), -77.75 (s, 3F). **HRMS** (ESI) Calculated for $C_{14}H_7F_3IN_2^+$ [M-TfO]$^+$ *m/z* 386.96006, found *m/z* 386.95891. **IR** (ATR) ν (cm^{-1}) 3073, 1528, 1474, 1424, 1381, 1238, 1142, 1023, 754. **Mp** (°C) 229-232 (decom.).

1-Methyl-6*H*-6λ^3-ioda-2,10b-diazaaceanthrylen-6-ium triflate (5ap)

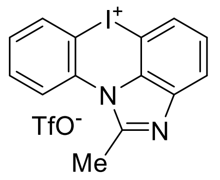

Following **GP3a** 2-methyl-1-(2-iodophenyl)-1*H*-benzo[*d*]imidazole (**4ap**, 66.8 mg, 200 µmol), *m*CPBA (61.2 mg, 300 µmol) and TfOH (44.2 µL, 500 µmol) in DCM (1 mL) gave 1-methyl-6*H*-6λ^3-ioda-2,10b-diazaaceanthrylen-6-ium triflate (**5ap**, 58.0 mg, 120 µmol, 60%) as a colorless powder after 72 h reaction time.

^{1}H NMR (601 MHz, DMSO-d_6) *δ* (ppm) 8.15 (d, *J* = 8.1 Hz, 1H), 8.08 (d, *J* = 8.4 Hz, 1H), 7.81 (d, *J* = 8.0 Hz, 1H), 7.76 (d, *J* = 7.9 Hz, 1H), 7.71 (t, *J* = 7.8 Hz, 1H), 7.52 (q, *J* = 8.0 Hz, 2H), 3.03 (s, 3H). **^{13}C NMR** (151 MHz, DMSO-d_6) *δ* (ppm) 153.4, 141.9, 135.4, 133.8, 133.2, 131.9, 128.8, 127.3, 124.7, 121.3, 120.7, 120.7 (q, *J* =322.5 Hz), 99.7, 86.2, 18.6. **^{19}F NMR** (565 MHz, DMSO-d_6) *δ* (ppm) -77.8. **HRMS** (ESI) Calculated for $C_{14}H_{12}IN_2^+$ [M-TfO+2H]$^+$ *m/z* 335.00397, found *m/z* 335.00377 (reduction of the iodine in the mass spectrometer). **IR** (ATR) *ν* (cm^{-1}) 2988, 1473, 1389, 1321, 1011, 743. **Mp** (°C) 223-234 (decom.).

3-(1*H*-Pyrazol-1-yl)-6*H*-6λ^3-ioda-2,10b-diazaaceanthrylen-6-ium triflate (5aq)

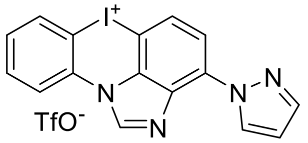

Following slightly modified **GP3a** 1-(2-iodophenyl)-4-(1*H*-pyrazol-1-yl)-1*H*-benzo[*d*]imidazole (**4aq**, 77.2 mg, 200 µmol) *m*CPBA (44.9 mg, 210 µmol) and TfOH (44.2 µL, 500 µmol) in MeCN (1 mL) gave 3-(1*H*-pyrazol-1-yl)-2,10b-diazaaceanthrylen-6-ium triflate (**5aq**, 69.8 mg, 131 µmol, 65%) as a colorless solid after suspending MeCN (1 mL).

^{1}H NMR (600 MHz, DMSO-d_6) δ (ppm) 9.58 (s, 1H), 9.24 (d, *J* = 2.6 Hz, 1H), 8.39 (d, *J* = 8.2 Hz, 1H), 8.15 (d, *J* = 8.8 Hz, 1H), 8.12 (d, *J* = 8.2 Hz, 1H), 8.03 (d, *J* = 8.8 Hz, 1H), 7.91 (d, *J* = 1.7 Hz, 1H), 7.70 (t, *J* = 7.8 Hz, 1H), 7.50 (t, *J* = 7.7 Hz, 1H), 6.69 (t, *J* = 2.2 Hz, 1H). **^{13}C NMR** (151 MHz, DMSO-d_6) δ (ppm) 141.7, 141.5, 133.8, 133.5, 133.0, 132.3, 132.1, 131.6, 129.3, 128.9, 126.1, 120.7 (d, *J* = 322.0 Hz), 119.6, 116.3, 108.5, 97.9, 81.4. **^{19}F NMR** (565 MHz, DMSO-d_6) δ (ppm) -77.75. **HRMS** (ESI) Calculated for $C_{16}H_{10}IN_4^+$ [M-TfO]$^+$ *m/z* 384.99447, found *m/z* 384.99412. **IR** (ATR) ν (cm^{-1}) 3120, 1988, 2901, 1612, 1532, 1504, 1459, 1402, 1360, 1223, 1203, 1021, 765. **Mp** (°C) 280-282 (decom.).

4,5-Dimethyl-6*H*-6λ^3-ioda-2,10b-diazaaceanthrylen-6-ium triflate (5ar)

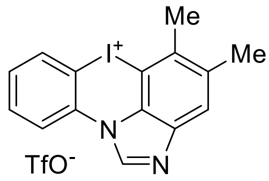

Following **GP3a** 1-(2-iodophenyl)-5,6-dimethyl-1*H*-benzo[*d*]imidazole (**4ar**, 69.6 mg, 200 µmol), *m*CPBA (44.9 mg, 210 µmol) and TfOH (44.2 µL, 500 µmol) in DCM (1 mL) gave 4,5-dimethyl-6*H*-6λ^3-ioda-2,10b-diazaaceanthrylen-6-ium triflate (**5ar**, 84.5 mg, 170 µmol, 85%) as a colorless solid.

^{1}**H NMR** (600 MHz, DMSO-d_6) δ (ppm) 9.34 (s, 1H), 8.21 (d, *J* = 8.2 Hz, 1H), 8.16 (d, *J* = 8.3 Hz, 1H), 7.68 (t, *J* = 7.7 Hz, 1H), 7.64 (s, 1H), 7.47 (t, *J* = 7.7 Hz, 1H), 2.46 (s, 3H), 2.41 (s, 3H). ^{13}**C NMR** (151 MHz, DMSO-d_6) δ (ppm) 141.7, 141.3, 136.9, 134.8, 133.3, 133.0, 132.5, 129.3, 126.8, 122.3, 120.7 (q, *J* = 322.3 Hz). 119.7, 96.7, 88.8, 21.9, 20.7. ^{19}**F NMR** (565 MHz, DMSO-d_6) δ (ppm) -77.74. **HRMS** (ESI) Calculated for $C_{15}H_{12}IN_2^+$ [M-TfO]$^+$ *m/z* 347.00397, found *m/z* 347.00356. **IR** (ATR) ν (cm^{-1}) 3128, 2988, 1544, 1440, 1286, 1221, 1144, 1024, 764. **Mp** (°C) 273 – 274 (decom.).

9-Chloro-4,5-dimethyl-6*H*-6λ^3-ioda-2,10b-diazaaceanthrylen-6-ium triflate (5at)

Following **GP3a** 1-(5-chloro-2-iodophenyl)-5,6-dimethyl-1*H*-benzo[*d*]imidazole (**4at**, 76.3 mg, 200 µmol), *m*CPBA (44.9 mg, 210 µmol) and TfOH (44.2 µL, 500 µmol) in DCM (1 mL) gave 9-chloro-4,5-dimethyl-6*H*-6λ^3-ioda-2,10b-diazaaceanthrylen-6-ium triflate (**5at**, 51.7 mg, 97.4 µmol, 49%) as a colorless solid 72 h reaction time.

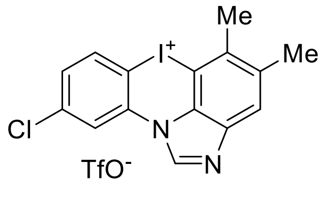

^{1}**H NMR** (601 MHz, CD_3OD) δ (ppm) 9.61 (s, 1H), 8.43 (d, *J* = 2.3 Hz, 1H), 8.15 (d, *J* = 8.9 Hz, 1H), 7.74 (s, 1H), 7.64 (dd, *J* = 8.9, 2.3 Hz, 1H), 2.59 (s, 3H), 2.56 (s, 3H). ^{13}**C NMR** (151 MHz, CD_3OD) δ (ppm) 140.7, 139.9, 139.5, 137.9, 135.1, 135.0, 132.5, 130.0, 125.8, 120.6, 120.4, 92.8, 87.8, 21.1, 19.9. ^{19}**F NMR** (565 MHz, CD_3OD) δ (ppm) -80.11. **HRMS** (ESI) Calculated for $C_{15}H_{13}ClIN_2^+$ [M-OTf+2H]$^+$ *m/z* 382.98065, found *m/z* 382.98019 (reduction of the iodine in the mass spectrometer). **IR** (ATR) ν (cm^{-1}) 2608, 1602, 1579, 1547, 1445, 1285, 1216, 1157, 1023, 859, 826. **Mp** (°C) 280 (decom.).

9-Bromo-4,5-dimethyl-6*H*-6λ^3-ioda-2,10b-diazaaceanthrylen-6-ium triflate (5au)

Following **GP3a** 1-(5-bromo-2-iodophenyl)-5,6-dimethyl-1*H*-benzo[*d*]imidazole (**4au**, 85.2 mg, 200 µmol), *m*CPBA (44.9 mg, 210 µmol) and TfOH (44.2 µL, 500 µmol) in DCM (1 mL) gave 9-bromo-4,5-dimethyl-6*H*-6λ^3-ioda-2,10b-diazaaceanthrylen-6-ium triflate (**5au**, 106 mg, 184 µmol, 92%) as a colorless solid after 72 h reaction time.

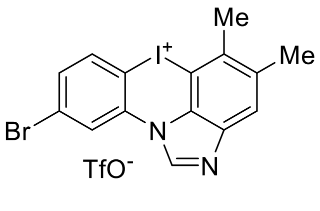

^{1}**H NMR** (601 MHz, CD_3OD) δ (ppm) 9.91 (s, 1H), 8.59 (d, *J* = 2.0 Hz, 1H), 8.10 (d, *J* = 8.8 Hz, 1H), 7.81 (dd, *J* = 8.8, 2.0 Hz, 1H), 7.77 (s, 1H), 2.61 (s, 3H), 2.57 (s, 3H). ^{13}**C NMR** (151 MHz, CD_3OD) δ (ppm) 140.6, 140.0, 136.5, 135.6, 135.2, 133.4, 127.7, 125.5, 123.4, 121.4, 120.0 (q, *J* = 104.9 Hz), 119.3, 93.8, 88.1, 21.1, 19.9. ^{19}**F NMR** (565 MHz, CD_3OD) δ (ppm) -80.08. **HRMS** (ESI) Calculated for $C_{15}H_{13}BrIN_2^+$ [M-OTf+2H]$^+$ *m/z* 426.93013, found *m/z* 426.92953 (reduction of the iodine in the mass spectrometer). **IR** (ATR) ν (cm^{-1}) 1547, 1286, 1235, 1189, 1025, 825. **Mp** (°C) 285.

2-Methyl-6*H*-6λ^3-ioda-2,10b-diazaaceanthrylen-2,6-diium bistriflate (5av)

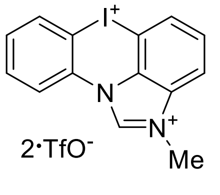

To a solution of 1-(2-iodophenyl)-3-methyl-1*H*-benzo[*d*]imidazolium triflate (**4av**, 200 µmol, 96.8 mg) and *m*CPBA (220 µmol, 44.9 mg) in DCM (1 mL) was added TfOH (44.2 µL, 500 µmol) dropwise and the

mixture was stirred for 1 h at room temperature and afterward for 72 h at 40 °C. The solvent was removed under reduced pressure, and the residue was suspended in Et_2O (1 mL) and a few drops of EtOAc. The suspension was stored at 4 °C for 1 h, filtered and washed with EtOAc 1:1 Et_2O (2 × 0.5 mL) to give 2-methyl-6*H*-6λ³-ioda-2,10b-diazaaceanthrylen-2,6-diium bistriflate (**5av**, 59.2 mg, 93.6 µmol, 47%) as a colorless powder.

^{1}H NMR (601 MHz, DMSO-d_6) *δ* (ppm) 10.91 (s, 1H), 8.34 (t, *J* = 8.0 Hz, 2H), 8.26 (d, *J* = 8.2 Hz, 1H), 8.23 (d, *J* = 8.3 Hz, 1H), 7.94 (t, *J* = 8.2 Hz, 1H), 7.87 (t, *J* = 7.8 Hz, 1H), 7.69 (t, *J* = 7.7 Hz, 1H), 4.22 (s, 3H). **^{13}C NMR** (151 MHz, DMSO-d_6) *δ* (ppm) 142.0, 134.1, 133.4, 132.9, 131.5, 130.2, 129.5, 128.9, 125.2, 120.5, 120.4 (q, *J* = 322.4 Hz), 116.7, 99.1, 89.7, 34.7. **^{19}F NMR** (565 MHz, DMSO-d_6) *δ* (ppm) -77.8. **HRMS** (ESI) Calculated for $C_{14}H_{12}IN_2^+$ $[M\text{-}2TfO+H]^+$ *m/z* 335.00397, found *m/z* 335.00375 (reduction in the mass spectrometer). **IR** (ATR) *ν* (cm^{-1}) 3157, 3089, 1567, 1462, 1240, 1157, 1024, 757. **Mp** (°C) 179 (decom.).

2-Phenyl-6*H*-6λ³-ioda-2,10b-diazaaceanthrylen-2,6-diium bistriflate (5aw)

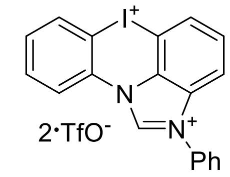

Following **GP3a** 1-(2-iodophenyl)-3-phenyl-1*H*-benzo[*d*]imidazol-3-ium triflate (109 mg, 200 µmol), *m*CPBA (44.9 mg, 210 µmol) and TfOH (44.2 µL, 500 µmol) in DCM (1 mL) gave 2-phenyl-6*H*-6λ³-ioda-2,10b-diazaaceanthrylen-2,6-diium bistriflate (**4aw**, 49.8 mg, 71.7 µmol, 36%) as a colorless solid after 72 h reaction time.

^{1}H NMR (601 MHz, CD_3OD) δ (ppm) 11.09 (s, 1H), 8.51 (d, *J* = 8.2 Hz, 1H), 8.36 (d, *J* = 7.7 Hz, 1H), 8.24 (d, *J* = 8.1 Hz, 1H), 8.01 – 7.90 (m, 4H), 7.88 – 7.79 (m, 4H), 7.71 (t, *J* = 7.7 Hz, 1H). **^{19}F NMR** (565 MHz, CD_3OD) δ (ppm) -80.09. **^{13}C NMR** (151 MHz, CD_3OD) δ (ppm) 142.6, 135.2, 135.1, 134.9, 133.9, 133.4, 133.0, 132.5, 131.9, 131.0, 130.6, 127.1, 126.6, 122.6, 121.7 (q, *J* = 318.5 Hz). 118.2, 98.0, 89.3. **HRMS** (ESI) Calculated for $C_{19}H_{13}IN_2^{2+}$ $[M\text{-}2{\cdot}OTf]^{2+}$ *m/z* 198.00562, found *m/z* 198.00647. **IR** (ATR) *ν* (cm^{-1}) 3125, 3061, 3028, 1533, 1483, 1386, 1272, 1235, 1157, 1018, 758, 693. **Mp** (°C) 265-267 (decom.).

2,4,5,7-Tetramethyl-6*H*-6λ³-ioda-2,10b-diazaaceanthrylen-2,6-diium bistriflate (5ax)

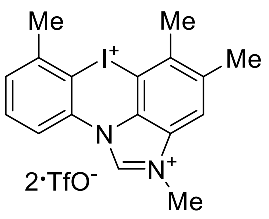

Following **GP3a** 1-(2-iodo-3-methylphenyl)-3,5,6-trimethyl-1*H*-benzo[*d*]imidazol-3-ium triflate (**4ax**, 421 mg, 800 µmol), *m*CPBA (180 mg, 880 µmol) and TfOH (177 µL, 2.00 mmol) in DCM (4 mL) gave 2,4,5,7-tetramethyl-6*H*-6λ³-ioda-2,10b-diazaaceanthrylen-2,6-diium bistriflate (**5ax**, 365 mg, 541 µmol, 68%) as a colorless solid after 72 h reaction time and suspension in EtOAc.

^{1}H NMR (601 MHz, CD_3OD) δ (ppm) 10.64 (s, 1H), 8.15 (dd, *J* = 8.2, 1.4 Hz, 1H), 8.06 (s, 1H), 7.78 (t, *J* = 7.9 Hz, 1H), 7.72 (d, *J* = 7.6 Hz, 1H), 4.28 (s, 3H), 2.82 (s, 3H), 2.68 (s, 3H), 2.66 (s, 3H). **^{13}C NMR** (151 MHz, CD_3OD) δ (ppm) 143.7, 143.3, 142.8, 139.1, 134.7, 134.3, 133.5, 130.9, 125.7, 121.7 (q, *J* = 318.7 Hz), 119.9, 117.9, 102.4, 90.1, 35.3, 25.9, 23.1, 21.6. **^{19}F NMR** (565 MHz, CD_3OD) δ (ppm) -80.11. **HRMS** (ESI) Calculated for $C_{17}H_{18}IN_2^+$ $[M\text{-}2{\cdot}OTf+H]^+$ *m/z* 377.05092, found *m/z* 377.05171 (reduction in the mass spectrometer). **IR** (ATR) ν (cm^{-1}) 3139, 3053, 1566, 1457, 1242, 1224, 1150, 1025, 781. **Mp** (°C) 260 – 261 (decom.)

4,5,7-Trimethyl-2-phenyl-6*H*-6λ^3-ioda-2,10b-diazaaceanthrylen-2,6-diium bistriflate (5ay)

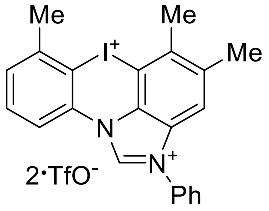

Following **GP3a** 1-(2-iodo-3-methylphenyl)-5,6-dimethyl-3-phenyl-1*H*-benzo[*d*]imidazol-3-ium triflate (**4ay**, 765 mg, 1.30 mmol), *m*CPBA (321 mg, 1.43 mmol) and TfOH (287 µL, 3.25 mmol) in DCM (6 mL) gave 4,5,7-trimethyl-2-phenyl-6*H*-6λ^3-ioda-2,10b-diazaaceanthrylen-2,6-diium bistriflate (**5ay**, 930 mg, 1.26 mmol, 97%) as a colorless solid after 72 h reaction time.

^{1}H NMR (601 MHz, DMSO-*d*$_6$) δ (ppm) 11.23 (s, 1H), 8.26 (dd, *J* = 8.3, 1.4 Hz, 1H), 7.99 (s, 1H), 7.95 (d, *J* = 7.4 Hz, 2H), 7.89 – 7.79 (m, 4H), 7.78 (d, *J* = 7.0 Hz, 1H), 2.83 (s, 3H), 2.64 (s, 3H), 2.57 (s, 3H). **^{13}C NMR** (151 MHz, DMSO-*d*$_6$) δ (ppm) 142.6.0 142.3, 142.2, 138.5, 133.1, 132.9, 132.6, 131.4, 131.0, 130.6, 130.3, 126.5, 125.2, 120.7 (q, *J* = 322.3 Hz), 119.7, 116.3, 102.7, 91.4, 25.4, 22.9, 20.9. **^{19}F NMR** (565 MHz, DMSO-*d*$_6$) δ -77.75. **HRMS** (ESI) Calculated for $C_{22}H_{20}IN_2^+$ [M-2•OTf+H]$^+$ *m/z* 439.06657, found *m/z* 439.06527 (reduction in the mass spectrometer). **IR** (ATR) *v* (cm^{-1}) 3112, 3084, 1549, 1491, 1160, 1025, 768, 699. **M$_p$** (°C) 251-252 (decom.).

2-Mesityl-4,5,7-trimethyl-6*H*-6λ^3-ioda-2,10b-diazaaceanthrylen-2,6-diium bistriflate (5az)

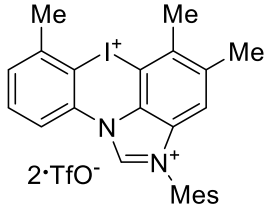

Following **GP3a** 1-(2-iodo-3-methylphenyl)-3-mesityl-5,6-dimethyl-1*H*-benzo[*d*]imidazol-3-ium triflate (**4az**, 126 mg, 200 µmol), *m*CPBA (44.9 mg, 220 µmol) and TfOH (44.2 µL, 500 µmol) in DCM (1 mL) gave 2-mesityl-4,5,7-trimethyl-6*H*-6λ^3-ioda-2,10b-diazaaceanthrylen-2,6-diium bistriflate (**5az**, 104 mg, 133 µmol, 66%) as a colorless solid after 72 h reaction time.

^{1}H NMR (601 MHz, DMSO-*d*$_6$) δ (ppm) 11.14 (s, 1H), 8.24 (d, *J* = 7.5 Hz, 1H), 7.84 (t, *J* = 7.8 Hz, 1H), 7.78 (d, *J* = 7.5 Hz, 1H), 7.61 (s, 1H), 7.31 (s, 2H), 2.85 (s, 3H), 2.64 (s, 3H), 2.53 (s, 3H), 2.42 (s, 3H), 2.10 (s, 6H). **^{13}C NMR** (151 MHz, DMSO-*d*$_6$) δ (ppm) 142.6, 142.3, 142.2, 141.6, 138.5, 135.4, 133.1, 132.9, 130.8, 130.4, 129.9, 127.9, 125.6, 120.7 (q, *J* = 322.5 Hz), 119.5, 115.8, 102.2, 91.3, 25.5, 22.9, 20.8, 20.7, 17.2. **^{19}F NMR** (565 MHz, DMSO-*d*$_6$) δ (ppm) -77.74. **HRMS** (ESI) Calculated for $C_{25}H_{26}IN_2O^+$ [M-2·OTf+OH]$^+$ *m/z* 497.10844, found *m/z* 497.10787. **IR** (ATR) *v* (cm^{-1}) 3099, 2988, 1542, 1474, 1246, 1152, 1024, 786. **M$_p$** (°C) 198-199 (decom.).

7-(1*H*-Pyrazol-1-yl)-6*H*-6λ^3-ioda-2,10b-diazaaceanthrylen-6-ium triflate (5ba)

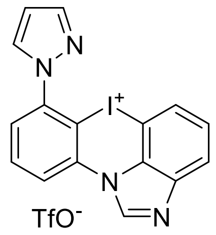

Following **GP3a** 1-(2-iodo-3-(1*H*-pyrazol-1-yl)phenyl)-1*H*-benzo[*d*]imidazole (**4ba**, 77.4 mg, 200 µmol), *m*CPBA (44.9 mg, 210 µmol) and TfOH (44.2 µL, 500 µmol) in DCM (1 mL) gave 7-(1*H*-pyrazol-1-yl)-6*H*-6λ^3-ioda-2,10b-diazaaceanthrylen-6-ium triflate (**5ba**, 94.2 mg, 176 µmol, 88%) as a colorless powder.

^{1}H NMR (600 MHz, CD_3OD) δ (ppm) 9.71 (s, 1H), 8.76 (d, *J* = 2.4 Hz, 1H), 8.26 (dd, *J* = 8.4, 1.3 Hz, 1H), 8.12 (d, *J* = 1.8 Hz, 1H), 8.03 (dd, *J* = 8.2, 1.2 Hz, 1H), 7.93 (dd, *J* = 7.9, 0.8 Hz, 1H), 7.91 – 7.80 (m, 2H), 7.63 (t, *J* = 8.0 Hz, 1H), 6.88 (dd, *J* = 2.7, 2.0 Hz, 1H). **^{13}C**

NMR (151 MHz, CD_3OD) δ (ppm) ^{13}C NMR (151 MHz, MeOD) δ 142.6, 141.1, 139.4, 136.4, 136.0, 135.3, 131.4, 129.7, 126.7, 123.2, 122.0, 121.8 (d, *J* = 318.6 Hz), 121.2, 119.5, 112.2, 93.8, 90.5. **^{19}F NMR** (565 MHz, CD_3OD) δ (ppm) -80.12. **HRMS** (ESI) Calculated for $C_{16}H_{10}IN_4^+$ [M-OTf]$^+$ *m/z* 384.99447, found *m/z* 384.99404. **IR** (ATR) *ν* (cm^{-1}) 3144, 1539, 1488, 1390, 1281, 1228, 1157, 1020, 784. **M$_p$** (°C) 292-293 (decom.).

4,5-Dimethyl-7-(1*H*-pyrazol-1-yl)-6*H*-6Λ^3-ioda-2,10b-diazaaceanthrylen-6-ium triflate (5bb)

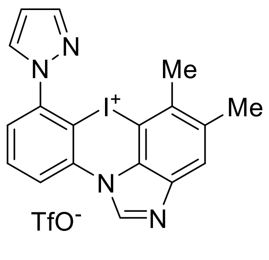

Following **GP3a** 1-(2-iodo-3-(1*H*-pyrazol-1-yl)phenyl)-5,6-dimethyl-1*H*-benzo[*d*]imidazole (**4bb**, 1.45 g, 3.50 mmol), *m*CPBA (786 mg, 3.85 mmol) and TfOH (774 µL, 8.75 mmol) in DCM (18 mL) gave 4,5-dimethyl-7-(1*H*-pyrazol-1-yl)-6*H*-6Λ^3-ioda-2,10b-diazaaceanthrylen-6-ium triflate (**5bb**, 999 mg, 1.77 mmol, 50%) as a colorless solid.

^{1}H NMR (601 MHz, DMSO-*d*$_6$) δ (ppm) 9.35 (s, 1H), 9.01 (d, *J* = 2.6 Hz, 1H), 8.26 (dd, *J* = 8.4, 1.3 Hz, 1H), 8.12 (d, *J* = 2.0 Hz, 1H), 8.04 (dd, *J* = 8.2, 1.2 Hz, 1H), 7.85 (t, *J* = 8.2 Hz, 1H), 7.62 (s, 1H), 6.95 (t, *J* = 2.32 Hz, 1H), 2.44 (s, 3H), 2.40 (s, 3H). **^{13}C NMR** (151 MHz, DMSO-*d*$_6$) δ (ppm) 141.9, 140.9, 140.1, 136.8, 136.1, 134.3, 133.8, 131.6, 130.6, 126.4, 121.9, 120.7 (q, *J* = 322.3 Hz), 119.0, 117.7, 111.1, 93.9, 88.2, 20.3, 20.3. **^{19}F NMR** (565 MHz, DMSO-*d*$_6$) δ (ppm) -77.75. **HRMS** (ESI) Calculated for $C_{18}H_{14}IN_4^+$ [M-OTf]$^+$ *m/z* 413.02577, found *m/z* 413.02443. **IR** (ATR) *ν* (cm^{-1}) 3572, 3438, 3108, 3083, 1585, 1492, 1246, 1160, 1030, 809, 779. **M$_p$** (°C) 301-303 (decom.).

6*H*-6Λ^3-Ioda-1,10b-diazaaceanthrylen-6-ium triflate (5bc)

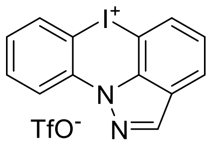

To a solution of 1-(2-iodophenyl)-1*H*-indazole (**4bc**, 64.0 mg, 200 µmol) and *m*CPBA (44.9 mg, 220 µmol) in DCM (1 mL) was added TfOH (44.2 µL, 500 µmol) dropwise and the solution was stirred for 72 h at 40 °C. The solvent was removed under reduced pressure, the residue suspended in Et_2O (1 mL) and stored for 1 h at 4 °C. After filtration and washing with Et_2O (1 mL) 6*H*-6Λ^3-ioda-1,10*b*-diazaaceanthrylen-6-ium triflate (**5bc**, 22.9 mg, 49.0 µmol, 24%) was obtained as a brown solid.

^{1}H NMR (600 MHz, DMSO-*d*$_6$) *δ* (ppm) 8.70 (s, 1H), 8.29 (dd, *J* = 8.3, 1.6 Hz, 1H), 8.10 (d, *J* = 7.7 Hz, 1H), 8.05 (dd, *J* = 8.2, 1.4 Hz, 1H), 7.95 (d, *J* = 7.9 Hz, 1H), 7.64 (td, *J* = 8.0, 7.4, 1.4 Hz, 1H), 7.47 (t, *J* = 7.9 Hz, 1H), 7.39 (ddd, *J* = 7.32, 7.23, 1,58 Hz, 1H). **^{13}C NMR** (151 MHz, DMSO-*d*$_6$) *δ* (ppm) 138.4, 134.7, 133.3, 133.2, 133.1, 129.7, 128.1, 126.5, 126.2, 124.5, 118.6, 95.8, 87.1 (the signal of the triflate carbon is missing). **^{19}F NMR** (565 MHz, DMSO-*d*$_6$) *δ* (ppm) -77.8. **HRMS** (ESI) Calculated for $C_{13}H_{10}IN_2^+$ [M-OTf+2H]$^+$ *m/z* 320.98832, found *m/z* 320.98835 (reduction of the iodine in the mass spectrometer). **IR** (ATR) *ν* (cm^{-1}) 3097, 1482, 1413, 1274, 1219, 1159, 1022, 750, 726. **Mp** (°C) 210-211 (decom.).

7-Methyl-6*H*-6λ^3-ioda-1,10b-diazaaceanthrylen-6-ium triflate (5bd)

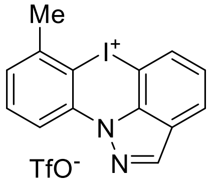

To a solution of 1-(2-iodo-3-methylphenyl)-1*H*-indazole (**4bd**, 64.0 mg, 200 µmol) and *m*CPBA (44.9 mg, 220 µmol) in DCM (1.5 mL) was added TfOH (44.2 µL, 500 µmol) dropwise and the solution was stirred for 72 h at 40 °C. The solvent was removed under reduced pressure, the residue suspended in Et_2O (1 mL) and stored for 1 h at 4 °C. After filtration and washing with Et_2O (1 mL) 7-methyl-6*H*-6λ^3-ioda-1,10b-diazaaceanthrylen-6-ium triflate (**5bd**, 42.9 mg, 890 µmol, 44%) was obtained as an off-white powder.

^{1}H NMR (600 MHz, DMSO-d_6) δ (ppm) 8.76 (s, 1H), 8.17 (d, *J* = 7.8 Hz, 1H), 8.09 (d, *J* = 6.8 Hz, 1H), 8.01 (d, *J* = 7.9 Hz, 1H), 7.56 (t, *J* = 7.8 Hz, 1H), 7.54 (t, *J* = 7.9 Hz, 1H), 7.38 (d, *J* = 7.5 Hz, 1H), 2.68 (s, 3H). **^{13}C NMR** (151 MHz, DMSO-d_6) δ (ppm) 140.5, 139.3, 135.6, 134.1, 132.4, 130.1, 129.3, 127.1, 126.2, 124.8, 120.7 (q, *J* = 322.5 Hz), 116.2, 99.8, 85.9, 24.7. **^{19}F NMR** (565 MHz, DMSO-d_6) δ (ppm) -77.8. **HRMS** (ESI) Calculated for $C_{14}H_{12}IN_2^+$ [M-OTf+2H]$^+$ *m/z* 335.00397, found *m/z* 335.00387 (reduction of the iodine in the mass spectrometer). **IR** (ATR) *v* (cm^{-1}) 3079, 1498, 1474, 1280, 1219, 1019, 783, 729. **Mp** (°C) 204-205 (decom.).

1-Methyl-6*H*-6λ^3-ioda-1,10b-diazaaceanthrylen-1,6-diium bistriflate (5be)

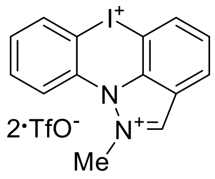

Following **GP3a** 1-(2-iodophenyl)-2-methyl-1*H*-indazol-2-ium triflate (**4be**, 96.8 mg, 200 µmol) *m*CPBA (44.9 mg, 220 µmol) and TfOH (44.2 µL, 500 µmol) in DCM (1 mL) gave 1-methyl-6*H*-6λ^3-ioda-1,10b-diazaaceanthrylen-1,6-diium bistriflate (**5be**, 37.8 mg, 59.8 µmol, 30%) as a colorless solid after suspending in Et_2O (1 mL) and a few drops of DCM.

^{1}H NMR (601 MHz, CD_3CN) δ (ppm) 9.57 (s, 1H), 8.60 (d, *J* = 7.8 Hz, 1H), 8.38 (d, *J* = 8.2 Hz, 1H), 8.32 (dd, *J* = 8.2, 1.4 Hz, 1H), 7.99 – 7.81 (m, 2H), 7.78 (dd, *J* = 8.3, 1.4 Hz, 1H), 7.66 (ddd, *J* = 8.5, 7.4, 1.4 Hz, 1H), 4.70 (s, 3H). **^{13}C NMR** (151 MHz, CD_3CN) δ (ppm) 148.5, 143.8, 139.8, 137.5, 135.0, 132.7, 132.1, 131.7, 128.5, 125.0, 122.9, 122.8, 121.8 (q, *J* = 319.8 Hz), 99.4, 90.0, 44.3. **^{19}F NMR** (565 MHz, CD_3CN) δ (ppm) -79.24. **HRMS** (ESI) Calculated for $C_{14}H_{12}IN_2^+$ [M-2•TfO+H]$^+$ *m/z* 335.00397, found *m/z* 335.00363 (reduction in the mass spectrometer). **IR** (ATR) v (cm^{-1}) 3064, 1988, 2901, 1626, 1461, 1222, 1154, 1024, 788, 770. **Mp** (°C) 286-288 (decom.).

4 Reactions of iodonium salt 5aa

2-(7-Iodo-1*H*-benzo[*d*]imidazol-1-yl)phenylacetate (6a) and 1-(2-iodophenyl)-1*H*-benzo[*d*]imidazol-7-yl acetate (6b)

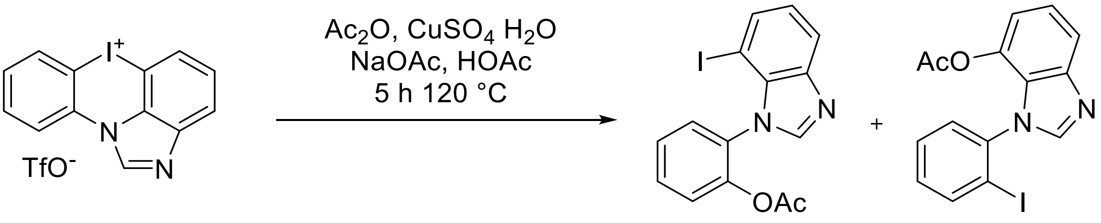

5aa **6a** (54%) **6b** (25%)

Using a modified literature procedure,[23] 6*H*-6λ^3-ioda-2,10b-diazaaceanthrylen-6-ium triflate (**5aa**, 70.2 mg, 150 µmol), NaOAc (32.8 mg, 400 mµol) and $CuSO_4{\cdot}5H_2O$ (3.8 mg, 15.0 µmol) were stirred in AcOH (1 mL) and Ac_2O (200 µL, 2.12 mmol) under an Ar atmosphere for 5 h at 120 °C. Toluene (1 mL) was added, and the solvent was removed under reduced pressure. The residue was dissolved in H_2O (10 mL) and DCM, and the layers were separated. The aqueous layer was extracted with DCM (2 × 10 mL), the combined organic layers were dried over Na_2SO_4, filtered, and the solvent was removed under reduced pressure. The residue was purified via column chromatography on silica (Cy : EtOAc 7:1 → 4:1), so that 2-(7-iodo-1*H*-benzo[*d*]imidazol-1-yl)phenylacetate (**6a**, 30.7 mg, 80.9 µmol, 54%) as a colorless, slowly crystallizing solid and 1-(2-iodophenyl)-1*H*-benzo[*d*]imidazol-7-ylacetate (**6b**, 14.1 mg, 37.3 µmol, 25%, more polar isomer) as a colorless solid were obtained.

2-(7-Iodo-1*H*-benzo[*d*]imidazol-1-yl)phenyl acetate (6a)

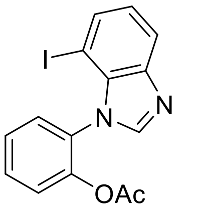

^{1}H NMR (601 MHz, $CDCl_3$) *δ* (ppm) 7.89 (s, 1H), 7.84 (dd, *J* = 8.1, 1.0 Hz, 1H), 7.72 (dd, *J* = 7.6, 1.0 Hz, 1H), 7.60 (ddd, *J* = 8.2, 7.6, 1.7 Hz, 1H), 7.50 (dd, *J* = 7.8, 1.7 Hz, 1H), 7.42 (td, *J* = 7.7, 1.4 Hz, 1H), 7.32 (dd, *J* = 8.2, 1.4 Hz, 1H), 7.04 (t, *J* = 7.8 Hz, 1H), 1.82 (s, 3H). **^{13}C NMR** (151 MHz, $CDCl_3$) *δ* (ppm) 168.6, 148.5, 145.4, 143.8, 135.3, 134.5, 131.4, 131.0, 128.0, 126.5, 124.4, 123.4, 120.9, 72.1, 20.3. **HRMS** (ESI) Calculated for $C_{15}H_{12}IN_2O_2^+$ $[M+H]^+$ *m/z* 378.99380, found *m/z* 378.99384. **IR** (ATR) *v* (cm^{-1}) 2915, 1748, 1504, 1410, 1189, 904, 777, 733. **M$_P$** (°C) 101.

1-(2-Iodophenyl)-1*H*-benzo[*d*]imidazol-7-yl acetate (6b)

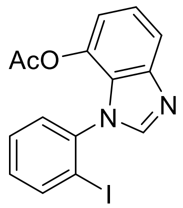

^{1}H NMR (601 MHz, $CDCl_3$) *δ* (ppm) 8.03 (dd, *J* = 8.0, 1.4 Hz, 1H), 7.88 (s, 1H), 7.78 (d, *J* = 8.1 Hz, 1H), 7.51 (td, *J* = 7.6, 1.4 Hz, 1H), 7.42 (dd, *J* = 7.8, 1.6 Hz, 1H), 7.32 (t, *J* = 8.0 Hz, 1H), 7.25 (td, *J* = 7.7, 1.6 Hz, 1H), 7.01 (d, *J* = 7.8 Hz, 1H), 1.58 (s, 3H). **^{13}C NMR** (151 MHz, $CDCl_3$) *δ* (ppm) 168.9, 145.8, 143.4, 139.8, 139.4, 136.1, 131.0, 129.5, 129.1, 123.1, 118.7, 117.4, 98.8, 60.5, 19.9. **HRMS** (ESI) Calculated for $C_{15}H_{12}IN_2O_2^+$ $[M+H]^+$ *m/z* 378.99380, found *m/z* 378.99352. **IR** (ATR) *v* (cm^{-1}) 3123, 1754, 1489, 1373, 1204, 1042, 772, 742. **M$_P$** (°C) 213.

Imidazo[4,5,1-*kl*]phenothiazine (7a)

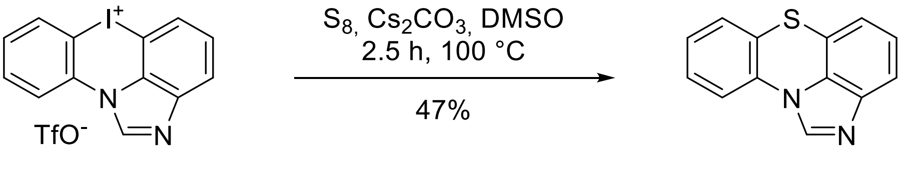

5aa **7a**

Following a literature procedure[24] 6*H*-6λ³-ioda-2,10b-diazaaceanthrylen-6-ium triflate (**5aa**, 70.2 mg, 150 µmol), S_8 (192 mg, 750 µmol) and Cs_2CO_3 (196 mg, 600 µmol) in dry DMSO (1.5 mL) were stirred under a N_2 atmosphere for 2.5 h at 100 °C. H_2O (10 mL) was added, and the mixture was extracted with EtOAc (4 × 10 mL). The combined organic layers were dried over Na_2SO_4, filtered, and the solvent was removed under reduced pressure. The residue was purified via column chromatography on silica (PE 3:1 EtOAc), so that imidazo[4,5,1-*kl*]phenothiazine (**7a**, 15.7 mg, 70.5 µmol, 47%) was obtained as a yellowish solid.

^{1}H NMR (600 MHz, CDCl3) *δ* (ppm) 8.26 (s, 1H), 7.31 (dd, *J* = 8.1, 1.2 Hz, 1H), 7.31 (dd, *J* = 8.2, 0.7 Hz, 1H), 7.10 (ddd, *J* = 8.1, 7.2, 1.6 Hz, 1H), 7.08 – 7.04 (m, 2H), 7.02 (ddd, *J* = 8.1, 7.2, 1.3 Hz, 1H), 6.74 (d, *J* = 7.2 Hz, 1H). **^{13}C NMR** (151 MHz, $CDCl_3$) *δ* (ppm) 142.7, 135.9, 131.6, 128.6, 127.7, 127.5, 126.8, 125.3, 122.8, 116.9, 116.9, 115.3 (one signal is missing). **HRMS** (ESI) Calculated for $C_{13}H_9N_2S^+$ $[M+H]^+$ *m/z* 225.04810, found *m/z* 225.04806. **IR** (ATR) *v* (cm^{-1}) 3072, 1486, 1432, 1280, 1194, 1040, 731. **M$_P$** (°C) 166-167.

Imidazo[4,5,1-*kl*]phenoselenazine (7b)

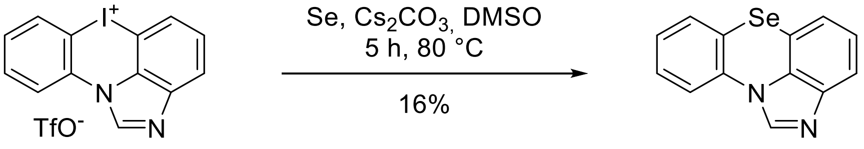

5aa **7b**

Using a modified literature procedure,[24] 6*H*-6λ³-ioda-2,10b-diazaaceanthrylen-6-ium triflate (**5aa**, 70.2 mg, 150 µmol), Se (47.4 mg, 600 µmol) and Cs_2CO_3 (196 mg, 600 µmol) in dry DMSO (1.5 mL) were stirred for 5 h at 80 °C under N_2 atmosphere. H_2O (10 mL) was added, and the mixture was extracted with EtOAc (4 × 10 mL). The combined organic layers were dried over Na_2SO_4, filtered, and the solvent was removed under reduced pressure. The residue was purified via column chromatography on silica (PE 3:1 EtOAc), so that imidazo[4,5,1-*kl*]phenoselenazine (**7b**, 6.4 mg, 23.6 µmol, 16%) was obtained as a yellowish solid.

^{1}H NMR (600 MHz, $CDCl_3$) *δ* (ppm) 8.39 (s, 1H), 7.41 (d, *J* = 8.5 Hz, 1H), 7.39 (d, *J* = 8.1 Hz, 1H), 7.21 (dd, *J* = 7.8, 1.4 Hz, 1H), 7.17 (td, *J* = 7.8, 1.5 Hz, 1H), 7.11 (t, *J* = 7.8 Hz, 1H), 7.04 (t, *J* = 7.6 Hz, 1H), 6.94 (d, *J* = 7.4 Hz, 1H). **^{13}C NMR** (151 MHz, $CDCl_3$) *δ* (ppm) 142.8, 136.8, 132.9, 130.1, 129.2, 128.2, 127.0, 125.6, 120.1, 117.7, 117.7, 115.7, 110.9. **^{77}Se NMR** (115 MHz, $CDCl_3$) δ (ppm) 287.78. **HRMS** (ESI) Calculated for $C_{13}H_9N_2{}^{80}Se^+$ $[M+H]^+$ *m/z* 272.99255, found *m/z* 272.99250. **IR** (ATR) *v* (cm^{-1}) 3075, 2922, 2852, 1494, 1284, 1190, 1030, 800, 733. **M$_P$** (°C) 148-149.

Imidazo[4,5,1-*kl*]phenotellurazine (7c)

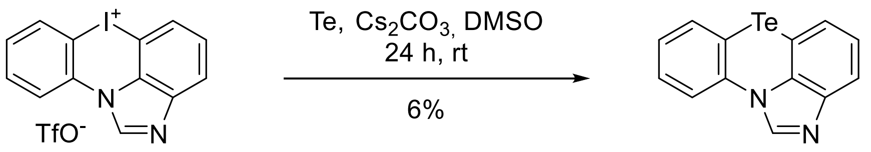

5aa **7c**

Using a modified literature procedure,[24] 6*H*-6λ^3-ioda-2,10b-diazaaceanthrylen-6-ium triflate (**5aa**, 70.2 mg, 150 µmol), Te (76.5 mg, 600 µmol) and Cs_2CO_3 (196 mg, 600 µmol) in dry DMSO (1.5 mL) were stirred for 24 h at room temperature under Ar atmosphere. H_2O (10 mL) was added, and the mixture was extracted with EtOAc (4 × 10 mL). The combined organic layers were dried over Na_2SO_4, filtered, and the solvent was removed under reduced pressure. The residue was purified via column chromatography on silica (Cy 60:40 EtOAc), so that imidazo[4,5,1-*kl*]phenotellurazine (**7c**, 3.0 mg, 9.38 µmol, 6%) was obtained as a yellowish solid.

^{1}H NMR (600 MHz, $CDCl_3$) δ (ppm) 8.51 (s, 1H), 7.50 (d, *J* = 8.3 Hz, 1H), 7.43 (dd, *J* = 7.5, 1.6 Hz, 1H), 7.34 (dd, *J* = 7.6, 1.5 Hz, 1H), 7.22 (t, *J* = 7.7 Hz, 1H), 7.20 – 7.09 (m, 2H), 7.03 (t, *J* = 7.4 Hz, 1H). **^{13}C NMR** (151 MHz, $CDCl_3$) δ (ppm) 142.6, 138.0, 135.8, 135.7, 131.5, 129.3, 127.1, 126.4, 126.0, 118.9, 116.4, 100.6, 91.8. **HRMS** (ESI) Calculated for $C_{13}H_9N_2{}^{130}Te^+$ [M+H]$^+$ *m/z* 322.98229, found *m/z* 322.98163. **IR** (ATR) *ν* (cm^{-1}) 3100, 3076, 1583, 1562, 1489, 1414, 1286, 1247, 1185, 774, 735. **M$_P$** (°C) 168-169.

6-Phenyl-6*H*-imidazo[4,5,1-*de*]phenazine (8)

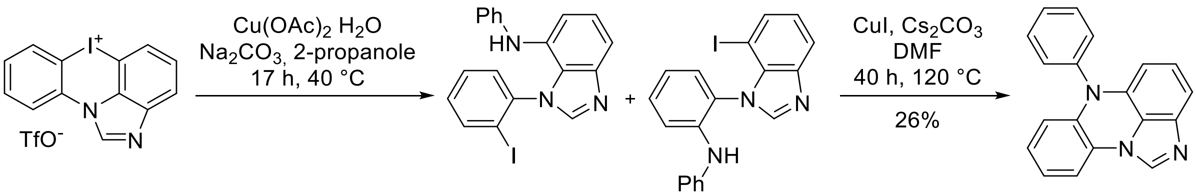

5aa **8**

Using a modified literature procedure,[25] 6*H*-6λ^3-ioda-2,10b-diazaaceanthrylen-6-ium triflate (**5aa**, 70.2 mg, 150 µmol), $Cu(OAc)_2 \cdot H_2O$ (6.0 mg, 30.0 µmol) and Na_2CO_3 (47.7 mg, 450 µmol) were stirred in 2-propanole (3 mL) under N_2 atmosphere for 17 h at 40 °C. The solvent was removed under reduced pressure, and the residue was dissolved in EtOAc (10 mL), sat. aq. NH_4Cl solution (2 mL) and H_2O (8 mL). The layers were separated, and the aqueous layer was extracted with EtOAc (2 × 10 mL). The combined organic layers were dried over Na_2SO_4, filtered, and the solvent was removed under reduced pressure. The residue was filtered through a plug of silica (Cy 1:1 EtOAc), so that a crude mixture of 1-(2-iodophenyl)-*N*-phenyl-1*H*-benzo[*d*]imidazol-7-amine and 2-(7-iodo-1*H*-benzo[*d*]imidazol-1-yl)-*N*-phenylaniline was obtained, which was used without further purification.

Using a modified literature procedure,[26] the crude mixture of 1-(2-iodophenyl)-*N*-phenyl-1*H*-benzo[*d*]imidazol-7-amine and 2-(7-iodo-1*H*-benzo[*d*]imidazol-1-yl)-*N*-

phenylaniline, CuI (5.7 mg, 30.0 µmol) and Cs_2CO_3 (97.7 mg, 300 µmol) was stirred in DMF (1 mL) for 40 h at 120 °C. The mixture was filtered through Celite, washed with EtOAc (20 mL), and the product was purified via column chromatography on silica (Cy 4:1 EtOAc), so that 6-phenyl-6*H*-imidazo[4,5,1-*de*]phenazine (**8**, 10.9 mg, 38.5 µmol, 26%) was obtained as pale orange solid.

^{1}H NMR (600 MHz, $CDCl_3$) δ (ppm) 8.12 (s, 1H), 7.63 (t, *J* = 7.8 Hz, 2H), 7.51 (t, *J* = 7.5 Hz, 1H), 7.38 (d, *J* = 7.0 Hz, 2H), 7.23 (dd, *J* = 7.5, 1.8 Hz, 1H), 6.95 (d, *J* = 8.2 Hz, 1H), 6.87 – 6.71 (m, 3H), 6.20 (dd, *J* = 8.0, 1.6 Hz, 1H), 5.56 (d, *J* = 7.6 Hz, 1H). **^{13}C NMR** (151 MHz, $CDCl_3$) δ (ppm) 142.7, 138.4, 136.9, 134.1, 132.6, 131.4, 130.4, 129.0, 127.0, 125.3, 125.0, 121.4, 115.9, 114.6, 111.2, 101.6 (one signal is missing due to overlapping). **HRMS** (ESI) Calculated for $C_{19}H_{14}N_3^+$ $[M+H]^+$ *m/z* 284.11822, found *m/z* 284.11811. **IR** (ATR) *v* (cm^{-1}) 3053, 1660, 1582, 1495, 1350, 1318, 1267, 1158, 823, 732, 695. **M$_P$** (°C) 167.

6-Tosyl-6*H*-imidazo[4,5,1-*de*]phenazine (9)

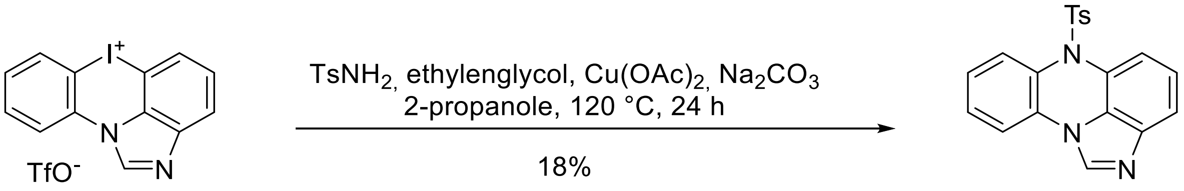

5aa **9**

Using a modified literature procedure,[27] 6*H*-6λ^3-ioda-2,10b-diazaaceanthrylen-6-ium triflate (**5aa**, 70.2 mg, 150 µmol), $TsNH_2$ (103 mg, 600 µmol), Na_2CO_3 (47.7 mg, 450 µmol) and $Cu(OAc)_2{\cdot}H_2O$ (5.4 mg, 30.0 µmol) were stirred in ethylene glycol (0.26 mL) and 2-propanol (2.3 mL) for 24 h at 100 °C in a pressure vial. EtOAc (10 mL) and brine (5 mL) were added, and the layers were separated. The aqueous layer was extracted with EtOAc (2 × 5 mL), and the combined organic layers were dried over Na_2SO_4, filtered and the solvent was removed under reduced pressure. The residue was purified via column chromatography on silica (Cy 1:1 EtOAc), so that 6-tosyl-6*H*-imidazo[4,5,1-*de*]phenazine (**9**, 9.8 mg, 27.1 µmol, 18%) was obtained as a colorless solid.

^{1}H NMR (600 MHz, $CDCl_3$) δ (ppm) 8.12 (s, 1H), 7.99 (dd, *J* = 7.6, 1.9 Hz, 1H), 7.64 (d, *J* = 7.8 Hz, 1H), 7.58 (d, *J* = 8.1 Hz, 1H), 7.39 (t, *J* = 7.9 Hz, 1H), 7.35 (td, *J* = 7.3, 1.8 Hz, 2H), 7.28 (d, *J* = 2.3 Hz, 1H), 7.04 – 6.72 (m, 4H), 2.26 (s, 3H). **^{13}C NMR** (151 MHz, $CDCl_3$) δ (ppm) 144.8, 135.7, 132.4, 130.7, 130.2, 129.2, 128.6, 127.9, 127.5, 126.6, 126.4, 125.2, 124.4, 119.7, 118.1, 114.4, 21.6 (one signal is missing). **HRMS** (ESI) Calculated for $C_{20}H_{16}N_3O_2S^+$ $[M+H]^+$ *m/z* 362.09577, found *m/z* 362.09628. **IR** (ATR) *v* (cm^{-1}) 2988, 2901, 1494, 1077, 1057. **M$_p$** (°C) 184-185.

6*H*-6λ^3-Ioda-2,10b-diazaaceanthrylen-6-ium iodide (10a)

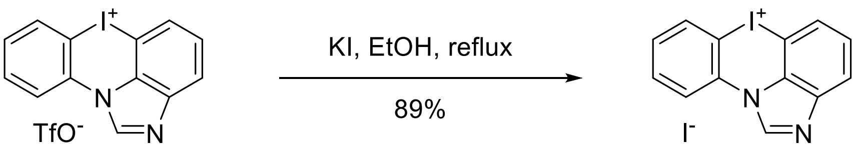

5aa **10a**

Using a literature procedure,[28] to a solution of 6*H*-6λ^3-ioda-2,10b-diazaaceanthrylen-6-ium triflate (**5aa**, 70.2 mg, 150 µmol) in boiling EtOH (4 mL) was added a solution of KI (49.8 mg, 300 µmol) in H_2O (0.5 mL) and the build suspension was stored for 1 h at 4 °C. The precipitate was filtered, washed with H_2O (5 mL), and dried in vacuo, so that 6*H*-6λ^3-ioda-2,10b-diazaaceanthrylen-6-ium iodide (**10a**, 59.4 mg, 130 µmol, 89%) was obtained as a yellowish solid.

^{1}H NMR (600 MHz, DMSO-d_6) δ (ppm) 9.41 (s, 1H), 8.34 – 8.31 (m, 1H), 8.27 (d, *J* = 8.3 Hz, 1H), 8.23 (d, *J* = 8.1 Hz, 1H), 7.80 (d, *J* = 8.0 Hz, 1H), 7.62 (tdd, *J* = 7.3, 1.5, 0.8 Hz, 1H), 7.49 (t, *J* = 8.0 Hz, 1H), 7.40 (ddd, *J* = 8.0, 7.3, 0.6 Hz, 1H). **^{13}C NMR** (151 MHz, DMSO-d_6) δ (ppm) 144.1, 141.3, 134.2, 132.6, 132.3, 128.6, 127.4, 126.8, 125.6, 122.0, 118.9, 100.4, 86.1. **HRMS** (ESI) Calculated for $C_{13}H_{10}IN_2^+$ [M-I+2H]$^+$ *m/z* 320.98832, found *m/z* 320.98828. **IR** (ATR) ν (cm^{-1}) 3068, 3015, 2825, 1753, 1578, 1497, 1249, 1188, 970, 784, 763. **M$_P$** (°C) 170 (decom.).

6*H*-6λ^3-Ioda-2,10b-diazaaceanthrylen-6-ium bromide (10b)

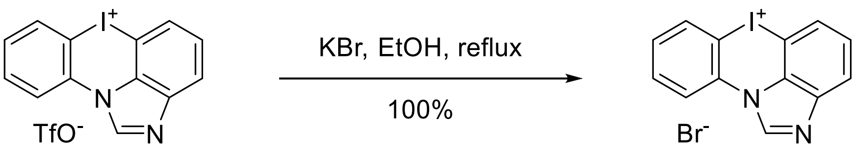

5aa **10b**

Using a literature procedure,[28] to a solution of 6*H*-6λ^3-ioda-2,10b-diazaaceanthrylen-6-ium triflate (**5a**, 46.8 mg, 100 µmol) in boiling EtOH (2.5 mL) was added a solution of KBr (23.8 mg, 200 µmol) in H_2O (0.5 mL) and the formed suspension was stored 1 h at 4 °C. The precipitate was filtered, washed with H_2O (5 mL) and dried in vacuo, so that 6*H*-6λ^3-ioda-2,10b-diazaaceanthrylen-6-ium bromide (**10b**, 39.8 mg, 100 µmol, 100%) was obtained as a colorless solid.

^{1}H NMR (600 MHz, DMSO-d_6) δ (ppm) 9.39 (s, 1H), 8.54 (d, *J* = 8.0 Hz, 1H), 8.29 (dd, *J* = 8.1, 1.4 Hz, 1H), 8.26 (dd, *J* = 8.3, 1.4 Hz, 1H), 7.79 (d, *J* = 7.9 Hz, 1H), 7.59 (ddd, *J* = 8.4, 7.3, 1.5 Hz, 1H), 7.48 (t, *J* = 8.0 Hz, 1H), 7.37 (ddd, *J* = 8.4, 7.3, 1.4 Hz, 1H). **^{13}C NMR** (151 MHz, DMSO-d_6) δ (ppm) 144.1, 141.1, 134.1, 132.4, 132.3, 128.4, 127.6, 126.6, 125.6, 121.9, 118.7, 103.0, 87.1. **HRMS** (ESI) Calculated for $C_{13}H_{10}IN_2^+$ [M-Br+2H]$^+$ *m/z* 320.98832, found *m/z* 320.98821. **IR** (ATR) ν (cm^{-1}) 3440, 3095, 3024, 1497, 1478, 1251, 749, 731. **M$_P$** (°C) 217.

7-Iodo-1-(2-iodphenyl)-1*H*-benzo[*d*]imidazole (11)

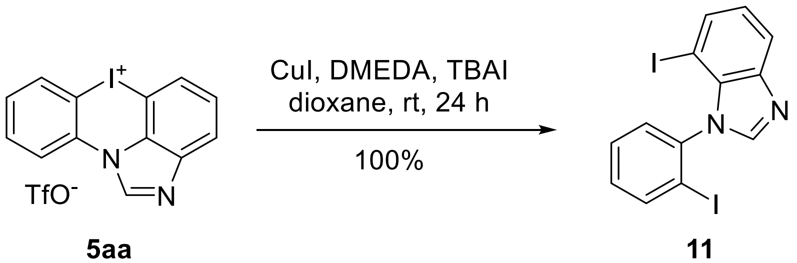

Using a slightly modified literature procedure,[29] 6*H*-6λ^3-ioda-2,10b-diazaaceanthrylen-6-ium triflate (**5aa**, 70.2 mg, 150 µmol), CuI (5.7 mg, 30.0 µmol), TBAI (111 mg, 300 µmol) and *N,N*-dimethylethylenediamine (3.24 µL, 30.0 µmol) were stirred at room temperature in dry dioxane under an Ar atmosphere for 24 h. H_2O (20 mL) was added, and the mixture was extracted with Et_2O (4 × 20 mL). The combined organic layers were dried over Na_2SO_4, filtered, and the solvent was removed under reduced pressure. The residue was filtered through a plug of silica (Cy -> Cy 1:1 EtOAc), so that 7-iodo-1-(2-iodophenyl)-1*H*-benzo[*d*]imidazole (**11**, 67.4 mg, 150 µmol, 100%) was obtained as a colorless solid.

^{1}H NMR (600 MHz, $CDCl_3$) δ (ppm) 8.10 (dd, *J* = 8.0, 1.4 Hz, 1H), 7.84 (d, *J* = 8.1 Hz, 1H), 7.61 (td, *J* = 7.6, 1.4 Hz, 1H), 7.40 (dd, *J* = 7.8, 1.6 Hz, 1H), 7.35 (td, *J* = 7.7, 1.6 Hz, 1H), 7.31 (t, *J* = 7.9 Hz, 1H), 7.24 (t, *J* = 7.8 Hz, 1H), 6.99 (d, *J* = 8.1 Hz, 1H). **^{13}C NMR** (151 MHz, $CDCl_3$) *δ* (ppm) 144.5, 143.9, 139.4, 138.1, 135.1, 134.1, 131.8, 131.3, 129.0, 124.6, 121.1, 101.7, 72.4. **HRMS** (ESI) Calculated for $C_{13}H_9N_2I_2^+$ $[M+H]^+$ *m/z* 446.88497, found *m/z* 446.88448. **IR** (ATR) *v* (cm^{-1}) 3080, 2921, 2852, 1779, 1559, 1491, 1410, 1296, 1258, 1223, 1065, 1023, 902, 803, 767, 736, 709. **M$_P$** (°C) 120.

5 Post-functionalizations of iodonium salts under preservation of the hypervalent iodine

2,4,5-Trimethyl-7-(1*H*-pyrazol-1-yl)-6*H*-6λ^3-ioda-2,10b-diazaaceanthrylen-2,6-diium bistriflate (12)

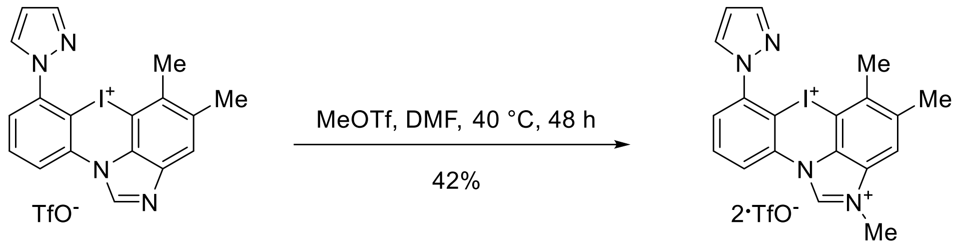

To a solution of iodonium salt **5bb** (999 mg, 1.77 mmol) in dry DMF under an Ar atmosphere was added MeOTf (805 µL, 7.11 mmol) and the reaction mixture was stirred at 40 °C for 48 h. The solvent was removed under reduced pressure and the residue was purified via column chromatography on silica (DCM 90:10 -> 50:50 MeOH) to obtain 2,4,5-trimethyl-7-(1*H*-pyrazol-1-yl)-6*H*-6λ^3-ioda-2,10b-diazaaceanthrylen-2,6-diium bistriflate (**12**, 541 mg, 745 µmol, 42%) as a colorless solid.

^{1}H NMR (600 MHz, DMSO-*d*$_6$) δ (ppm) 10.84 (s, 1H), 9.12 (d, *J* = 2.7 Hz, 1H), 8.34 (d, *J* = 7.2 Hz, 1H), 8.29 (d, *J* = 8.5 Hz, 1H), 8.20 (d, *J* = 2.0 Hz, 1H), 8.10 (t, *J* = 8.2 Hz, 1H), 8.05 (s, 1H), 7.01 (t, *J* = 2.4 Hz, 1H), 4.18 (s, 3H), 2.58 (s, 3H), 2.54 (s, 3H). **^{13}C NMR** (151 MHz, DMSO-*d*$_6$) δ (ppm) 141.8, 140.3, 140.1, 136.6, 136.1, 134.2, 131.6, 130.8, 130.8, 124.5, 121.2, 120.7 (q, *J* = 322.4 Hz), 119.1, 115.7, 111.4, 97.8, 89.7, 34.5, 20.8, 20.6. **HRMS** (ESI) Calculated for $C_{19}H_{18}IN_4O^+$ [M-2·OTf+OH]$^+$ *m/z* 445.05199, found *m/z* 445.05026. **IR** (ATR) ν (cm^{-1}) 3136, 3044, 1565, 1462, 1395, 1225, 1149, 1025, 799, 735. **Mp** (°C) 313-315 (decom.)

General procedure for the oxidative substitution of protected dicationic iodonium salts with oxygen in C1-position (GP4)

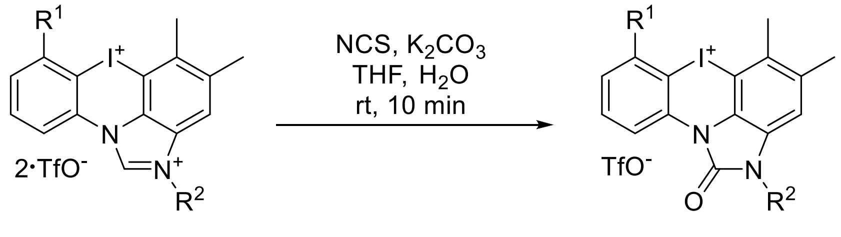

Following a modified literature procedure[30] to a solution of dicationic iodonium salt **5ax–az**, **12** (50.0 µmol) in THF (1 mL) was added NCS (13.2 mg, 100 µmol) and a solution of K_2CO_3 (13.8 mg, 100 µmol) in H_2O (0.12 mL) and the reaction mixture was stirred at room temperature for 10 min. The solvent was removed under reduced pressure, and the residue was purified via column chromatography on silica (DCM 98:2 MeOH), so that the 1-oxo-iodonium salt **13** as a yellowish solid.

Following **GP4** reaction of **12** (36.3 mg, 50.0 µmol) gave **13a** (14.5 mg, 24.5 µmol, 48%).

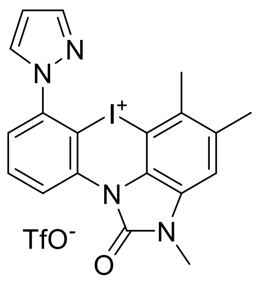

^{1}H NMR (601 MHz, DMSO-*d*$_6$) δ (ppm) 9.00 (dd, *J* = 8.5, 1.4 Hz, 1H), 8.93 (d, *J* = 2.6 Hz, 1H), 8.12 (d, *J* = 1.9 Hz, 1H), 7.95 (dd, *J* = 8.0, 1.4 Hz, 1H), 7.83 (t, *J* = 8.2 Hz, 1H), 7.28 (s, 1H), 6.93 (t, *J* = 2.3 Hz, 1H), 3.40 (s, 3H), 2.36 (s, 3H), 2.35 (s, 3H). **^{13}C NMR** (151 MHz, DMSO-*d*$_6$) δ (ppm) 152.0, 140.6, 137.0, 135.5, 135.3, 133.4, 130.9, 128.1, 127.5, 123.5, 118.9, 117.4, 111.7, 110.9, 93.0, 87.3, 27.8, 20.2, 19.9 (the carbon atom of the triflate could not be measured). **^{19}F NMR** (565 MHz, DMSO-*d*$_6$) δ (ppm) -77.75. **HRMS** (ESI) Calculated for $C_{19}H_{16}IN_4O^+$ [M-OTf]$^+$ *m/z* 443.03634, found *m/z* 443.03602. **IR** (ATR) *v* (cm^{-1}) 3112, 2919, 1748, 1588, 1460, 1245, 1153, 1026, 790. **M$_p$** (°C) 200-202 (decom.).

Following **GP4** reaction of **5ax** (33.7 mg, 50.0 µmol) gave **13b** (8.9 mg, 16.5 µmol, 33%).

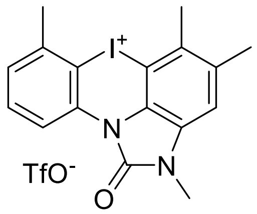

^{1}H NMR (600 MHz, CD_3OD) δ (ppm) 8.86 (d, *J* = 8.4 Hz, 1H), 7.59 (t, *J* = 7.9 Hz, 1H), 7.50 (d, *J* = 7.4 Hz, 1H), 7.28 (s, 1H), 3.51 (s, 3H), 2.76 (s, 3H), 2.51 (s, 6H). **^{13}C NMR** (151 MHz, CD_3OD) δ (ppm) 154.3, 142.1, 138.7, 136.3, 133.8, 130.9, 130.5, 129.3, 125.5, 117.7, 113.4, 100.0, 88.7, 28.2, 26.3, 21.6, 21.2 (the carbon atom of the triflate could not be measured). **^{19}F NMR** (565 MHz, CD_3OD) δ (ppm) -80.11. **HRMS** (ESI) Calculated for $C_{17}H_{16}IN_2O^+$ [M-OTf]$^+$ *m/z* 391.03019, found *m/z* 391.02954. **IR** (ATR) *v* (cm^{-1}) 2921, 2852, 1730, 1470, 1379, 1273, 1222, 1157, 1022, 790, 731. **M$_p$** (°C) 209-211 (decom.).

Following **GP4** reaction of **5ay** (36.8 mg, 50.0 µmol) gave **13c** (5.4 mg, 8.96 µmmol, 18%).

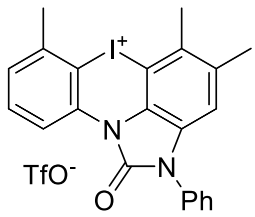

^{1}H NMR (601 MHz, $CDCl_3$) δ (ppm) 9.05 (dd, *J* = 8.5, 1.7 Hz, 1H), 7.62 (t, *J* = 7.8 Hz, 2H), 7.54 (t, *J* = 7.5 Hz, 1H), 7.51 (t, *J* = 7.8 Hz, 3H), 7.35 (dd, *J* = 7.6, 1.8 Hz, 1H), 6.94 (s, 1H), 2.78 (s, 3H), 2.57 (s, 3H), 2.39 (s, 3H). **^{13}C NMR** (151 MHz, $CDCl_3$) δ (ppm) 151.1, 140.5, 137.8, 133.3, 133.3, 133.0, 130.3, 130.3, 129.6, 129.4, 128.7, 126.3, 122.1, 120.6 (q, *J* = 319.6 Hz), 117.7, 113.2, 97.6, 86.6, 27.0, 22.3, 21.5. **^{19}F NMR** (565 MHz, $CDCl_3$) δ (ppm) -78.26. **HRMS** (ESI) Calculated for $C_{22}H_{18}IN_2O^+$ [M-OTf]$^+$ *m/z* 453.04584, found *m/z* 453.04515. **IR** (ATR) *v* (cm^{-1}) 2962, 2924, 2855, 1739, 1466, 1256, 1152, 1025, 794. **M$_p$** (°C) 223-225 (decom.).

Following **GP4** reaction of **5az** (38.9 mg, 50.0 µmol) gave **13d** (8.3 mg, 12.9 µmol, 26%).

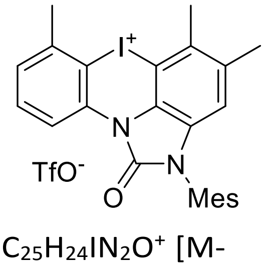

^{1}H NMR (600 MHz, DMSO-*d*$_6$) δ (ppm) 8.60 (d, *J* = 7.7 Hz, 1H), 7.64 (dd, *J* = 8.4, 7.4 Hz, 1H), 7.54 (d, *J* = 6.8 Hz, 1H), 7.15 (s, 2H), 6.74 (s, 1H), 2.75 (s, 3H), 2.44 (s, 3H), 2.35 (s, 3H), 2.34 (s, 3H), 2.02 (s, 6H). **^{13}C NMR** (151 MHz, DMSO-*d*$_6$) δ (ppm) 151.2, 141.5, 139.4, 137.3, 136.3, 135.6, 132.5, 129.8, 129.5, 129.3, 127.8, 127.5, 125.5, 120.7 (q, *J* = 322.3 Hz), 115.2, 111.7, 101.4, 91.1, 25.7, 21.4, 20.7, 20.4, 17.3. **^{19}F NMR** (565 MHz, DMSO-*d*$_6$) δ (ppm) -77.74. **HRMS** (ESI) Calculated for $C_{25}H_{24}IN_2O^+$ [M-OTf]$^+$ *m/z* 495.09279, found *m/z* 495.09237. **IR** (ATR) *v* (cm^{-1}) 2925, 1724, 1463, 1372, 1239, 1150, 1025, 996, 788. **M$_p$** (°C) 183-185 (decom.).

2,4,5-Trimethyl-7-(1*H*-pyrazol-1-yl)-1-(tosylimino)-1,2-dihydro-6*H*-6λ^3-ioda-2,10b-diazaaceanthrylen-6-yl triflate (14a)

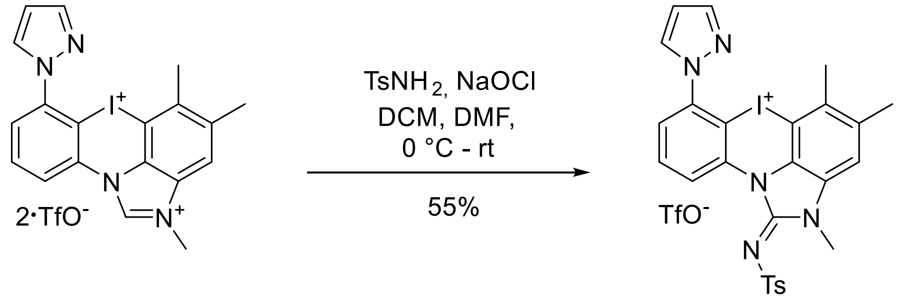

12 **14a**

Following a modified literature procedure,[30] $TsNH_2$ (51.4 mg, 300 µmol) and $NaOCl·5H_2O$ (39.4 mg, 300 mmol) were stirred in DCM (1 mL) for 0.5 h at room temperature. Then iodonium salt **12** (21.8 mg, 30.0 µmol) and DMF (1 mL) were added at 0 °C, and the reaction mixture was stirred for 15 min at room temperature. The solvent was removed, and the residue was purified via column chromatography on silica (DCM 98:2 MeOH) to obtain **14a** (12.2 mg, 16.4 µmol, 55%) as a yellowish solid.

^{1}H NMR (601 MHz, $CDCl_3$) δ (ppm) 8.36 (dd, *J* = 8.5, 1.2 Hz, 1H), 8.12 (d, *J* = 2.7 Hz, 1H), 8.10 (d, *J* = 2.0 Hz, 1H), 7.61 (dd, *J* = 8.0, 1.3 Hz, 1H), 7.57 (d, *J* = 8.3 Hz, 2H), 7.46 (t, *J* = 8.2 Hz, 1H), 7.26 (d, *J* = 7.9 Hz, 2H), 7.14 (s, 1H), 6.62 (t, *J* = 2.3 Hz, 1H), 3.84 (s, 3H), 2.48 (s, 3H), 2.44 (s, 3H), 2.42 (s, 3H). **^{13}C NMR** (151 MHz, $CDCl_3$) δ (ppm) 147.3, 143.2, 141.9, 140.7, 138.3, 138.1, 136.3, 134.0, 132.5, 130.3, 129.7, 128.6, 127.8, 125.9, 120.7 (q, *J* = 320.0 Hz), 120.0, 119.3, 113.2, 111.6, 95.6, 89.3, 32.7, 21.6, 21.4, 20.9. **^{19}F NMR** (565 MHz, $CDCl_3$) δ (ppm) -78.17. **HRMS** (ESI) Calculated for $C_{26}H_{23}IN_5O_2S^+$ [M-OTf]$^+$ *m/z* 596.06117, found *m/z* 596.06057. **IR** (ATR) *v* (cm^{-1}) 3120, 2923, 1581, 1477, 1381, 1247, 1142, 1085, 1027, 877, 686. **M$_p$** (°C) 210-112 (decom.).

2,4,5,7-Tetramethyl-1-(tosylimino)-1,2-dihydro-6*H*-6λ^3-ioda-2,10b-diazaaceanthrylen-6-yl triflate (14b)

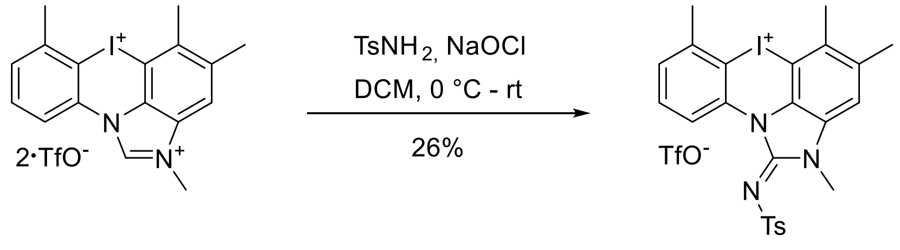

5ax **14b**

Following a modified literature procedure,[30] the corresponding *N*-tosylamide (17.1 mg, 100 µmol) and $NaOCl·5H_2O$ (13.2 mg, 100 mmol) were stirred in DCM (1 mL) for 0.5 h at room temperature. Then 2,4,5,7-tetramethyl-6*H*-6λ^3-ioda-2,10b-diazaaceanthrylen-2,6-diium bistriflate (**5ax**, 33.7 mg, 50.0 µmol) was added at 0 °C, and the reaction was stirred for 15 min at room temperature. The solvent was removed, and the residue purified via column chromatography on silica (DCM 98:2

MeOH) to obtain 2,4,5,7-tetramethyl-1-(tosylimino)-1,2-dihydro-6*H*-6λ^3-ioda-2,10b-diazaaceanthrylen-6-yl triflate (**14b**, 8.9 mg, 12.8 µmol, 26%) as a yellowish solid.

^{1}H NMR (601 MHz, CD_3OD) δ (ppm) 8.23 (dd, *J* = 5.8, 4.0 Hz, 1H), 7.77 (d, *J* = 8.2 Hz, 2H), 7.60 – 7.48 (m, 3H), 7.34 (d, *J* = 8.0 Hz, 2H), 3.63 (s, 3H), 2.79 (s, 3H), 2.55 (s, 3H), 2.53 (s, 3H), 2.42 (s, 3H). **^{13}C NMR** (151 MHz, CD_3OD) δ (ppm) 149.8, 144.2, 143.1, 142.8, 140.3, 138.5, 133.8, 132.7, 131.9, 131.4, 130.6, 130.5, 127.0, 121.8 (q, *J* = 318.3 Hz), 120.1, 115.0, 105.2, 93.7, 31.0, 26.3, 21.8, 21.4, 21.3. **^{19}F NMR** (565 MHz, CD_3OD) δ (ppm) -78.28. **HRMS** (ESI) Calculated for $C_{24}H_{23}IN_3O_2S^+$ $[M\text{-}OTf]^+$ *m/z* 544.05502, found *m/z* 544.05414. **IR** (ATR) *ν* (cm^{-1}) 3051, 2918, 1580, 1275, 1241, 1146, 1088, 1032, 886, 693. **M$_p$** (°C) 247-249 (decom.).

1-((*tert*-Butoxycarbonyl)imino)-2,4,5-trimethyl-7-(1*H*-pyrazol-1-yl)-1,2-dihydro-6*H*-6λ^3-ioda-2,10b-diazaaceanthrylen-6-yl triflate (15)

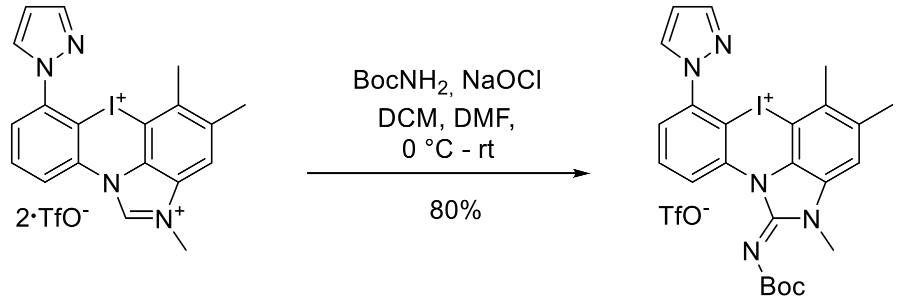

12 **15**

Following a modified literature procedure,[30] $BocNH_2$ (53.1 mg, 300 µmol) and $NaOCl\cdot5H_2O$ (39.4 mg, 300 mmol) were stirred in DCM (1 mL) for 0.5 h at room temperature. Then iodonium salt **12** (21.8 mg, 30.0 µmol) and DMF (1 mL) were added at 0 °C, and the reaction mixture was stirred for 1 h at room temperature. The solvent was removed, and the residue purified via column chromatography on silica (DCM 98:2 MeOH) to obtain **15** (16.6 mg, 24.0 µmol, 80%) as a yellowish solid.

^{1}H NMR (601 MHz, $CDCl_3$) δ (ppm) 8.29 (d, *J* = 2.7 Hz, 1H), 8.10 (d, *J* = 1.9 Hz, 1H), 7.98 (t, *J* = 4.8 Hz, 1H), 7.64 (d, *J* = 5.4 Hz, 2H), 7.05 (s, 1H), 6.69 (t, *J* = 2.3 Hz, 1H), 3.58 (s, 3H), 2.45 (s, 3H), 2.38 (s, 3H), 1.40 (s, 9H). **^{13}C NMR** (151 MHz, $CDCl_3$) δ (ppm) 157.9, 150.8, 141.9, 138.5, 137.8, 136.8, 133.6, 131.0, 130.4, 128.9, 128.9, 120.7 (q, *J* = 320.2 Hz), 118.3, 118.1, 112.4, 111.5, 95.8, 89.9, 80.3, 30.3, 28.3, 21.3, 20.9. **^{19}F NMR** (565 MHz, $CDCl_3$) δ -78.14. **HRMS** (ESI) Calculated for $C_{24}H_{25}IN_5O_2^+$ $[M\text{-}OTf]^+$ *m/z* 542.10475, found *m/z* 542.10449. **IR** (ATR) *ν* (cm^{-1}) 3116, 2915, 1584, 1243, 1147, 1026, 786, 732. **M$_p$** (°C) 151-152 (decom.).

1-Imino-2,4,5-trimethyl-7-(1*H*-pyrazol-1-yl)-1,2-dihydro-6*H*-6λ³-ioda-2,10b-diazaaceanthrylen-6-ium triflate·HOTf (16)

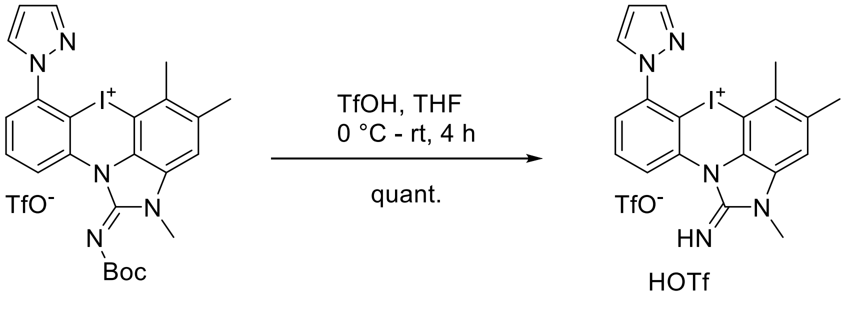

15 **16**

To a suspension of the iodonium salt **15** (10.3 mg, 15.0 µmol) in THF (1 mL) was added TfOH (6.62 mL, 75.0 µmol) at 0 °C and the reaction mixture was stirred at room temperature for 4 h. The solvent was removed under reduced pressure and purified via column chromatography on silica (DCM 10:1 -> 1:1 MeOH) to obtain 1-imino-2,4,5-trimethyl-7-(1*H*-pyrazol-1-yl)-1,2-dihydro-6*H*-6λ³-ioda-2,10b-diazaaceanthrylen-6-ium triflate·HOTf (**16**, 11.1 mg, 15.0 µmol, 100%) as a colorless solid.

^{1}H NMR (601 MHz, CD_3OD) δ (ppm) 8.75 (d, *J* = 2.7 Hz, 1H), 8.25 (dd, *J* = 8.4, 1.2 Hz, 1H), 8.13 (d, *J* = 2.0 Hz, 1H), 8.05 (dd, *J* = 8.1, 1.2 Hz, 1H), 7.94 (t, *J* = 8.2 Hz, 1H), 7.58 (s, 1H), 6.90 (dd, *J* = 2.7, 2.0 Hz, 1H), 3.80 (s, 3H), 2.51 (s, 3H), 2.47 (s, 3H). **^{13}C NMR** (151 MHz, CD_3OD) δ (ppm) 153.1, 142.3, 140.4, 140.1, 135.6, 135.1, 133.4, 131.6, 131.5, 129.8, 121.8 (q, *J* = 318.6 Hz), 121.3, 120.2, 114.8, 112.5, 97.0, 91.9, 30.8, 21.0, 20.6. **^{19}F NMR** (565 MHz, CD_3OD) δ (ppm) -80.10. **HRMS** (ESI) Calculated for $C_{19}H_{17}IN_5^+$ [M-HOTf-OTf]$^+$ *m/z* 442.05232, found *m/z* 442.05153. **IR** (ATR) *ν* (cm^{-1}) 3145, 2922, 1590, 1144, 1011, 987, 786, 699. **M_p** (°C) 206 (decom.).

6 References

1. W. L. Armarego, C. L. Chai. *Purification of Laboratory Chemicals-Six Edition;* Elsevier Inc.: London, 2009.
2. Sheldrick, G. M. *J. Appl. Crystallogr.* **2009** (42), 339.
3. Sheldrick, G. M. S. *Acta Cryst.* **2015**, 3.
4. Sheldrick, G. M. S. *Acta Cryst.* **2008**, 112.
5. Obermayer, D.; Damm, M.; Kappe, M. C. *Chem. Eur. J.* **2013,** *19* (47), 15827–15830.
6. Navarrete-Vázquez, G.; Rojano-Vilchis, M. d. M.; Yépez-Mulia, L.; Meléndez, V.; Gerena, L.; Hernández-Campos, A.; Castillo, R.; Hernández-Luis, F. *Eur. J. Med. Chem.* **2006,** *41* (1), 135–141.
7. Qiu, D.; Wei, H.; Zhou, L.; Zeng, Q. *Appl. Organomet. Chem.* **2014,** *28* (2), 109–112.
8. Ravi, O.; Shaikh, A.; Upare, A.; Singarapu, K. K.; Bathula, S. R. *J. Org. Chem.* **2017,** *82* (8), 4422–4428.
9. Khaksar, S.; Heydari, A.; Tajbakhsh, M.; Vahdat, S. M. *J. Fluor. Chem.* **2010,** *131* (12), 1377–1381.
10. Wu, H.; Dai, W.; Saravanamurugan, S.; Li, H.; Yang, S. *Green Chem.* **2020,** *22* (17), 5822–5832.
11. Boelke, A.; Lork, E.; Nachtsheim, B. J. *Chem. Eur. J.* **2018,** *24* (70), 18653–18657.
12. Diness, F.; Fairlie, D. P. *Angew. Chem. Int. Ed.* **2012,** *51* (32), 8012–8016.

13. Maddock, L. C. H.; Nixon, T.; Kennedy, A. R.; Probert, M. R.; Clegg, W.; Hevia, E. *Angew. Chem. Int. Ed.* **2018,** *57* (1), 187–191.
14. Martin, A. D.; Siamaki, A. R.; Belecki, K.; Gupton, B. F. *J. Org. Chem.* **2015,** *80* (3), 1915–1919.
15. Youn, S. W.; Lee, E. M. *Org. Lett.* **2016,** *18* (21), 5728–5731.
16. Wonner, P.; Vogel, L.; Düser, M.; Gomes, L.; Kniep, F.; Mallick, B.; Werz, D. B.; Huber, S. M. *Angew. Chem. Int. Ed.* **2017,** *56* (39), 12009–12012.
17. Engl, P. S.; Senn, R.; Otth, E.; Togni, A. *Organometallics* **2015,** *34* (7), 1384–1395.
18. Boelke, A.; Nachtsheim, B. J. *Adv. Synth. Catal.* **2020,** *362* (1), 184–191.
19. Fan, Y.-S.; Jiang, Y.-J.; An, D.; Di Sha; Antilla, J. C.; Zhang, S. *Org. Lett.* **2014,** *16* (23), 6112–6115.
20. Feuerstein, W.; Holzer, C.; Gui, X.; Neumeier, L.; Klopper, W.; Breher, F. *Chem. Eur. J.* **2020,** *26* (71), 17156–17164.
21. Ren, P.; Vechorkin, O.; Csok, Z.; Salihu, I.; Scopelliti, R.; Hu, X. *Dalton Trans.* **2011,** *40* (35), 8906–8911.
22. Roth, R.; Schmidt, G.; Prud'homme, A.; Abele, S. *Org. Process Res. Dev.* **2019,** *23* (2), 234–243.
23. Hu, Z.; Tang, Y.; Yu, B. *J. Am. Chem. Soc.* **2019,** *141* (12), 4806–4810.
24. Wang, M.; Fan, Q.; Jiang, X. *Org. Lett.* **2016,** *18* (21), 5756–5759.
25. Zhu, D.; Liu, Q.; Luo, B.; Chen, M.; Pi, R.; Huang, P.; Wen, S. *Adv. Synth. Catal.* **2013,** *355* (11-12), 2172–2178.
26. Zhu, L.; Guo, P.; Li, G.; Lan, J.; Xie, R.; You, J. *J. Org. Chem.* **2007,** *72* (22), 8535–8538.
27. Zhu, D.; Chen, M.; Li, M.; Luo, B.; Zhao, Y.; Huang, P.; Xue, F.; Rapposelli, S.; Pi, R.; Wen, S. *Eur. J. Med. Chem.* **2013,** *68*, 81–88.
28. Boelke, A.; Kuczmera, T. J.; Caspers, L. D.; Lork, E.; Nachtsheim, B. J. *Org. Lett.* **2020,** *22* (18), 7261–7266.
29. Wu, B.; Yoshikai, N. *Angew. Chem. Int. Ed.* **2015,** *54* (30), 8736–8739.
30. Lima, H. M.; Lovely, C. J. *Org. Lett.* **2011,** *13* (21), 5736–5739.

7 NMR spectra

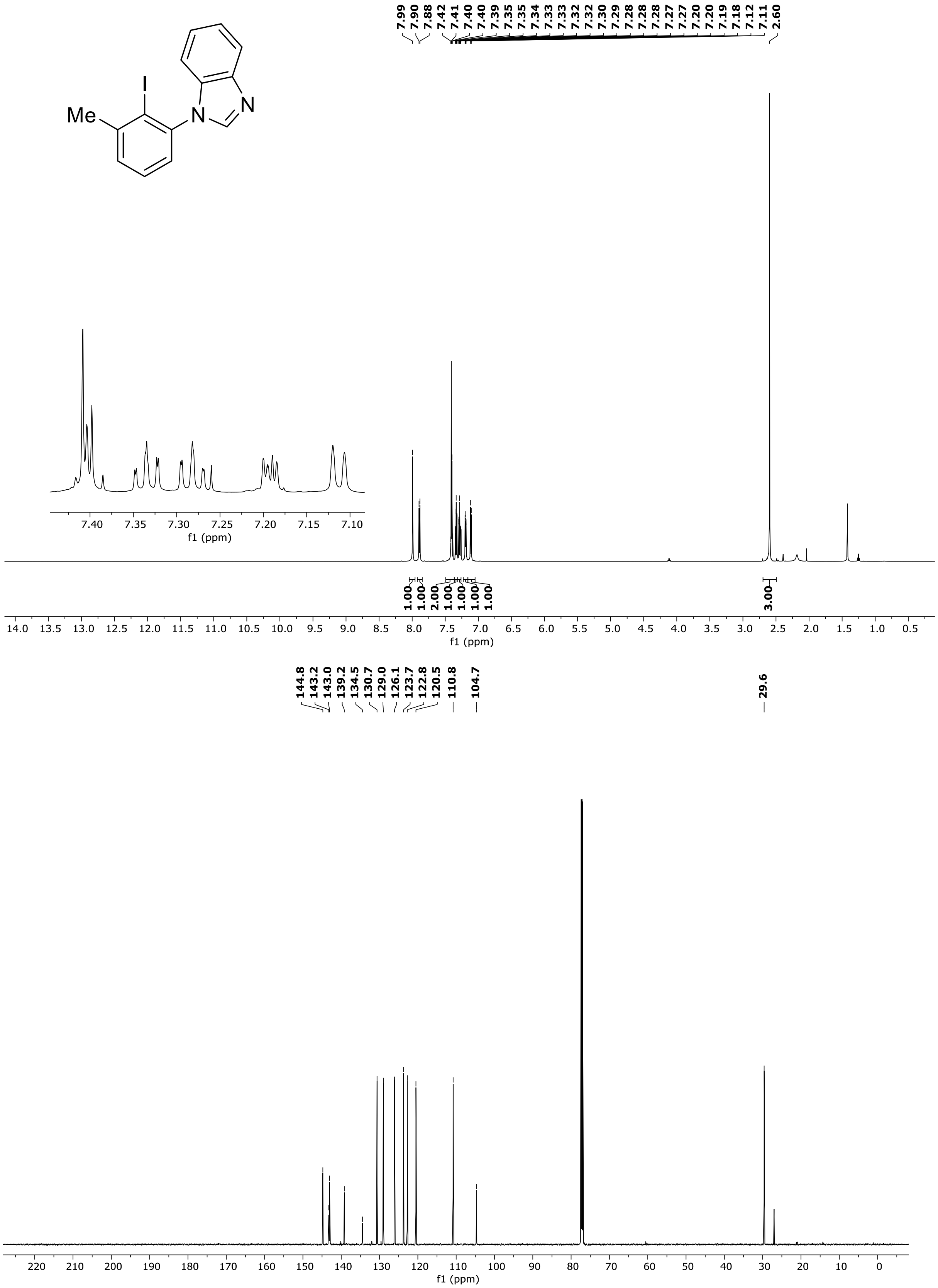

Figure S1: ^{1}H and ^{13}C NMR spectra of 1-(2-iodo-3-methylphenyl)-1*H*-benzo[*d*]imidazole (**4ab**) in $CDCl_3$.

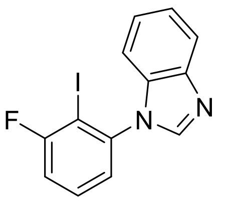
F
I
N
N

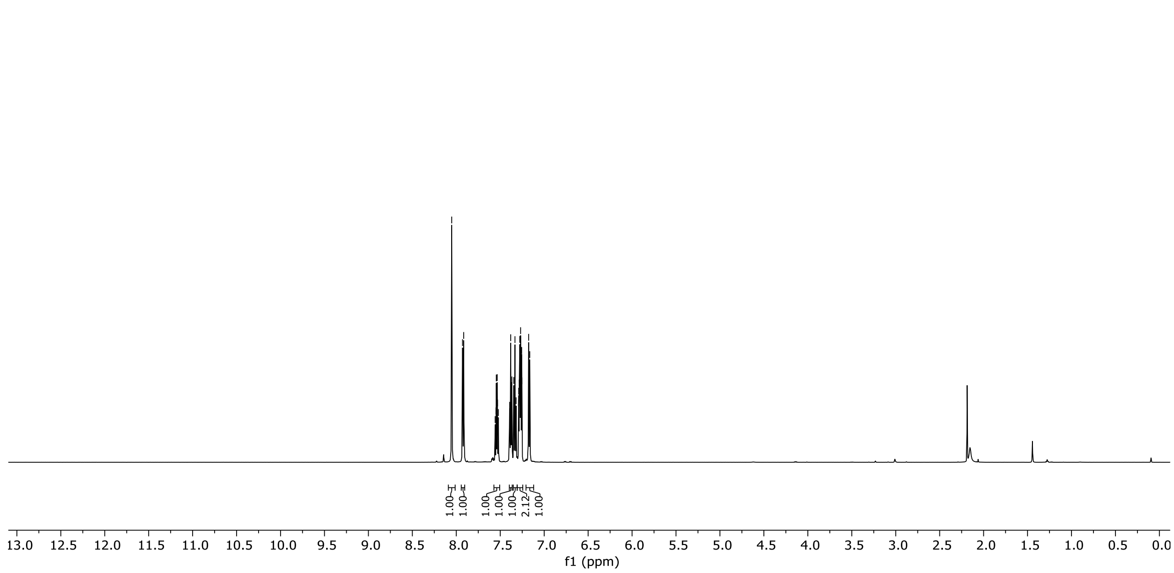
8.05
7.93
7.92
7.56
7.55
7.54
7.53
7.53
7.52
7.39
7.38
7.37
7.35
7.33
7.32
7.29
7.29
7.28
7.28
7.27
7.26
7.26
7.25
7.18
7.16
1.00
1.00
1.00
1.00
1.00
2.12
1.00
f1 (ppm)

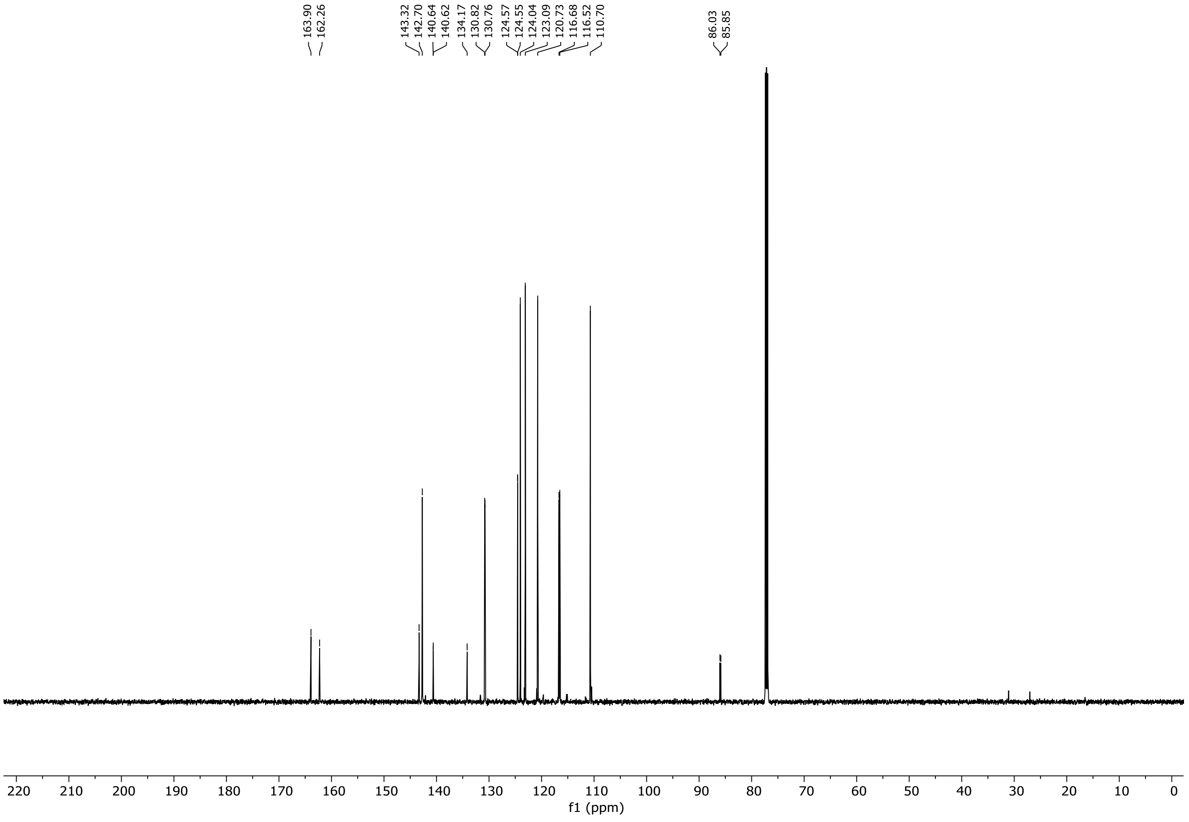
163.90
162.26
143.32
142.70
140.64
140.62
134.17
130.82
130.76
124.57
124.55
124.04
123.09
120.73
116.68
116.52
110.70
86.03
85.85
f1 (ppm)

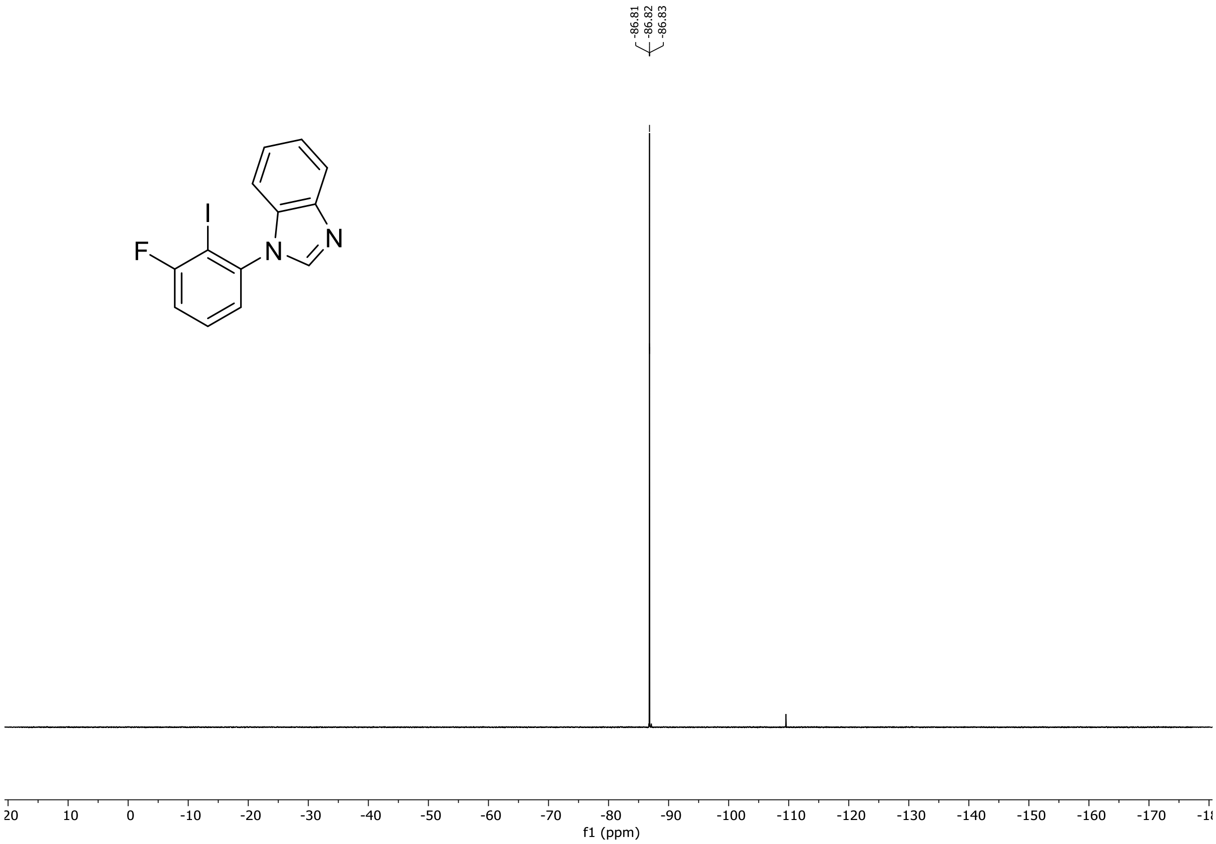

Figure S2: ^{1}H, ^{13}C and ^{19}F NMR spectra of 1-(2-iodo-3-methylphenyl)-1*H*-benzo[*d*]imidazole (**4ac**) in $CDCl_3$.

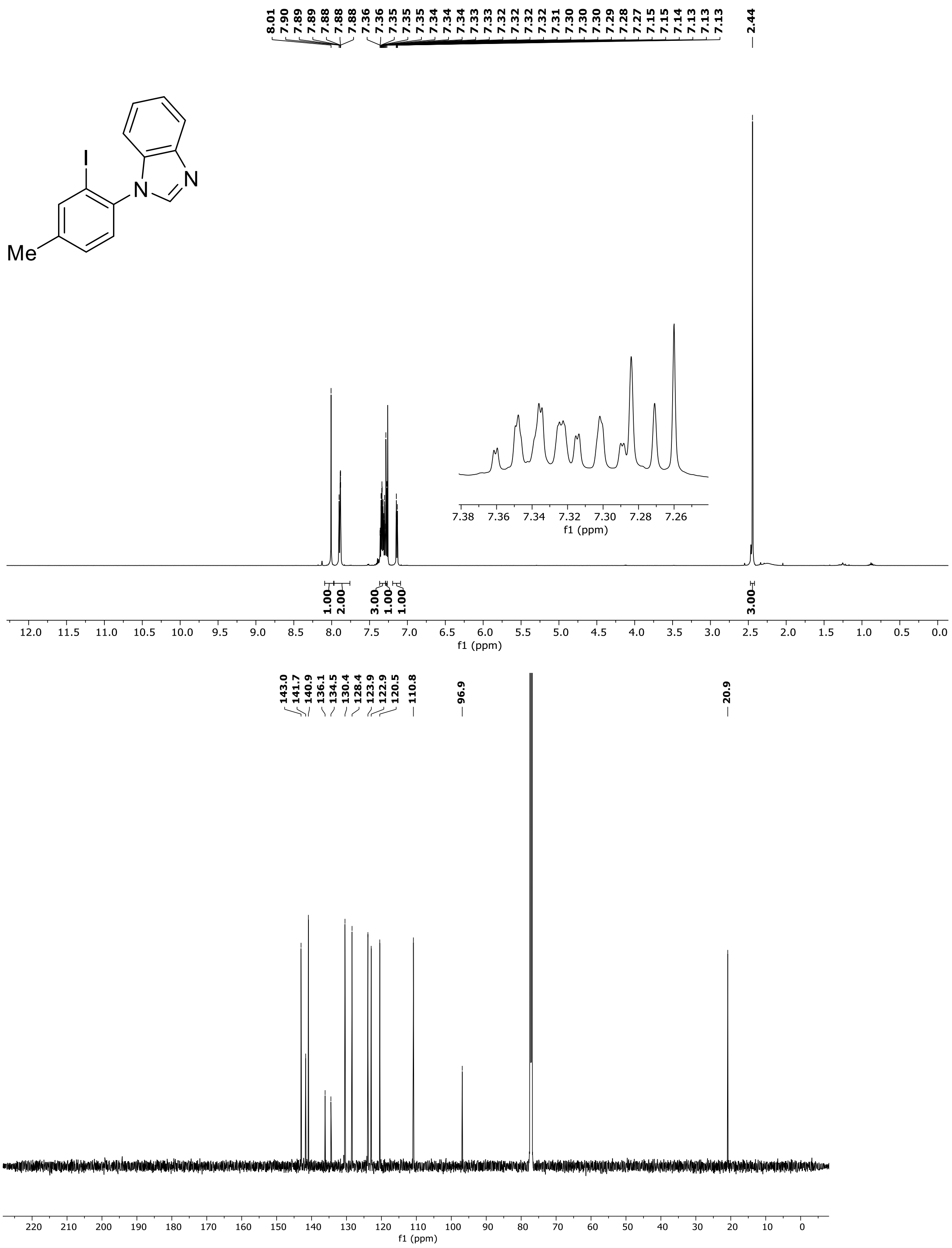

Figure S3: ^{1}H and ^{13}C NMR spectra of 1-(2-iodo-4-methylphenyl)-1*H*-benzo[*d*]imidazole (**4af**) in $CDCl_3$.

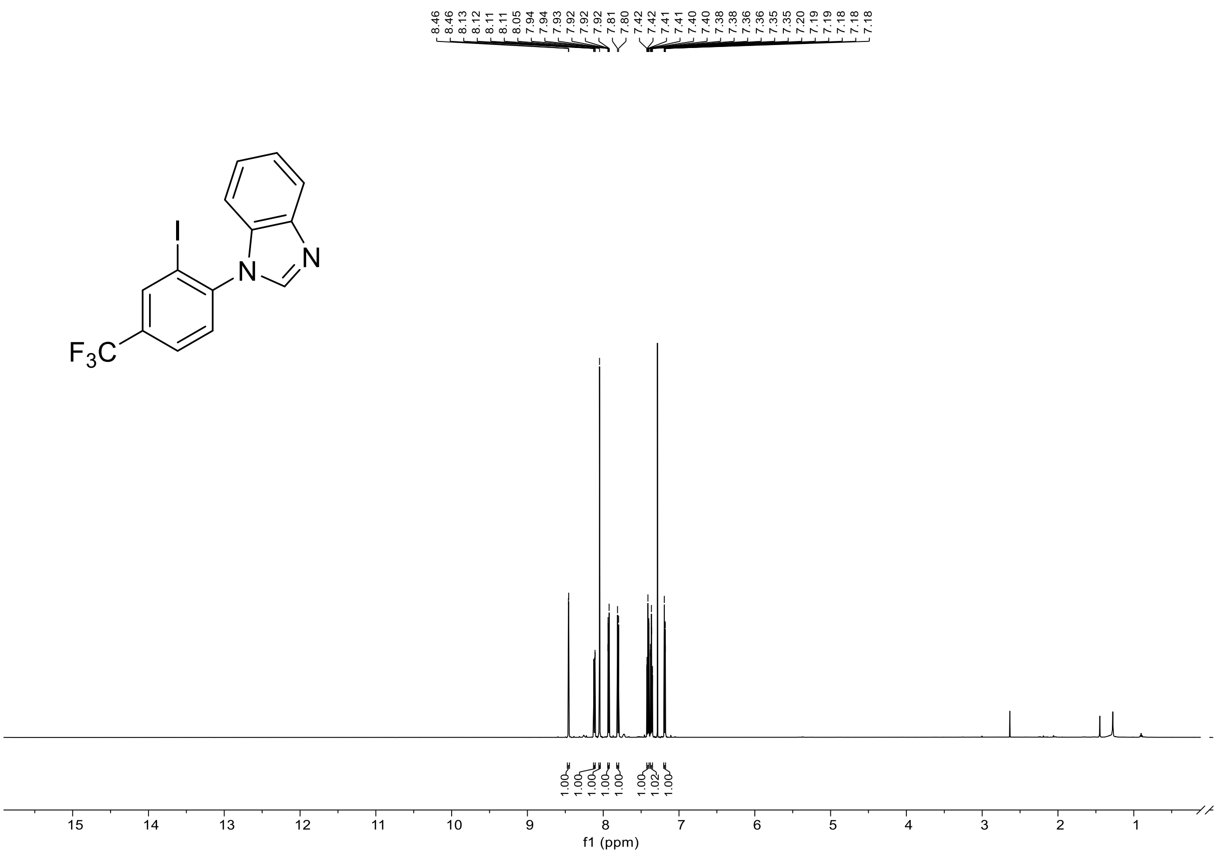

I
N
N
F_3C
8.46
8.46
8.13
8.12
8.11
8.11
8.05
7.94
7.94
7.93
7.92
7.92
7.92
7.81
7.80
7.42
7.42
7.41
7.41
7.40
7.40
7.38
7.38
7.36
7.36
7.35
7.35
7.20
7.19
7.19
7.18
7.18
7.18
1.00
1.00
1.00
1.00
1.00
1.00
1.02
1.00
15 14 13 12 11 10 9 8 7 6 5 4 3 2 1
f1 (ppm)

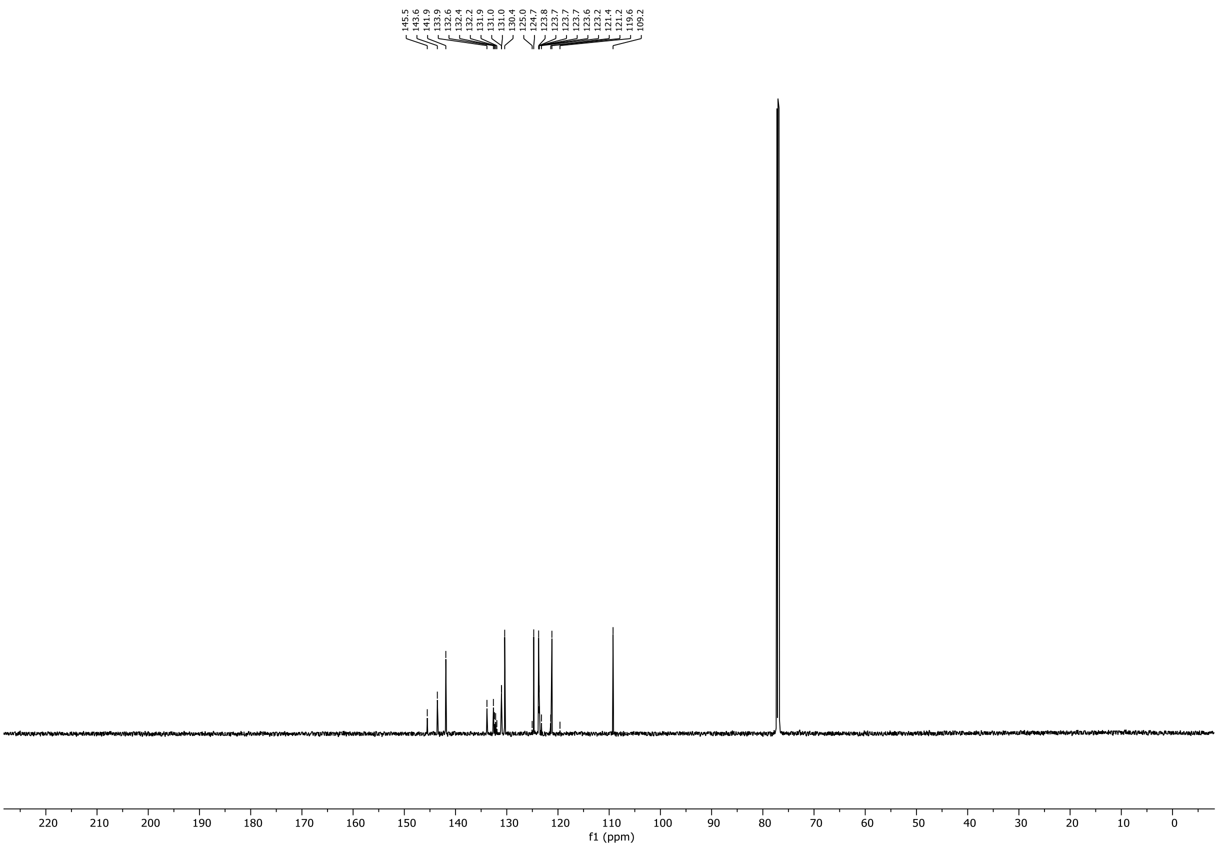

145.5
143.6
141.9
133.9
132.6
132.4
132.2
131.9
131.0
130.4
125.0
124.7
123.8
123.7
123.7
123.7
123.6
123.2
121.4
121.2
119.6
109.2
220 210 200 190 180 170 160 150 140 130 120 110 100 90 80 70 60 50 40 30 20 10 0
f1 (ppm)

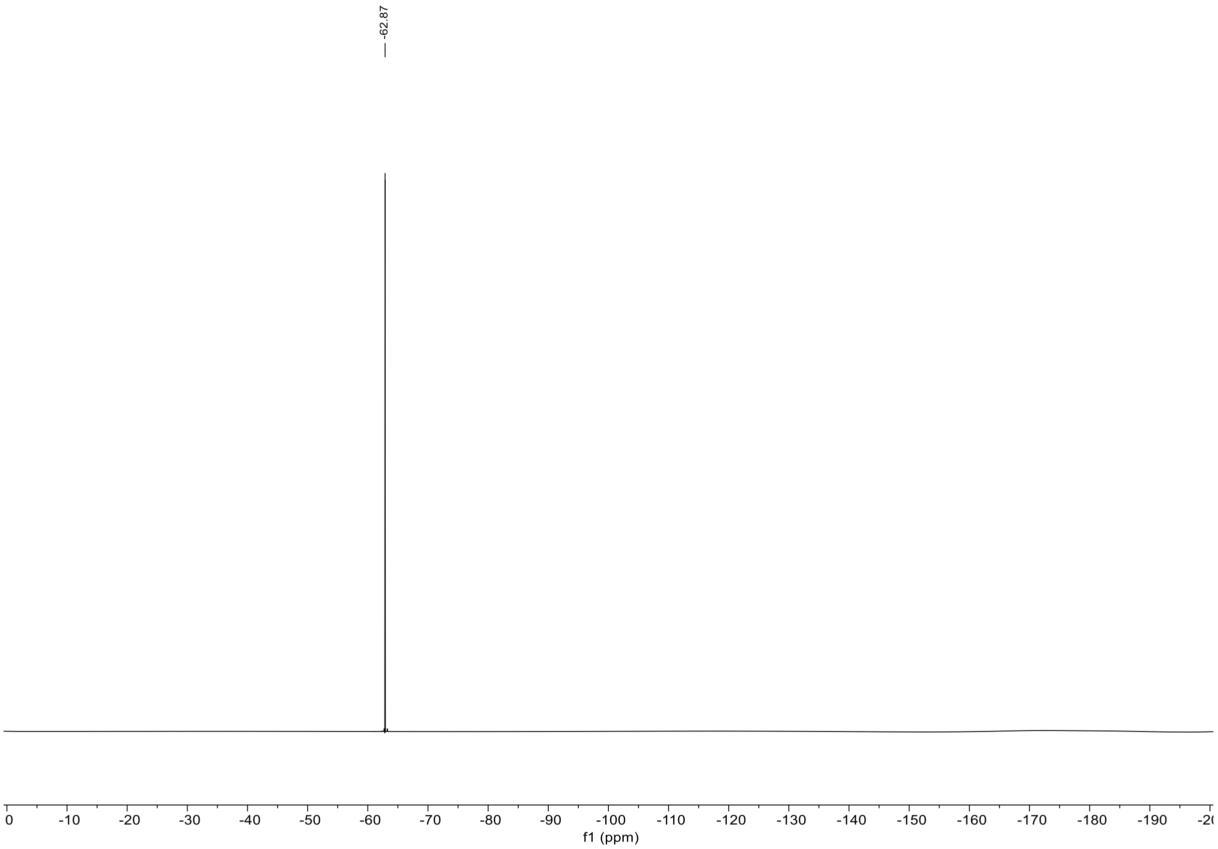

Figure S4: ^{1}H, ^{13}C and ^{19}F NMR spectra of 1-(2-iodo-4-(trifluoromethyl)phenyl)-1*H*-benzo[*d*]imidazole (**4ag**) in $CDCl_3$.

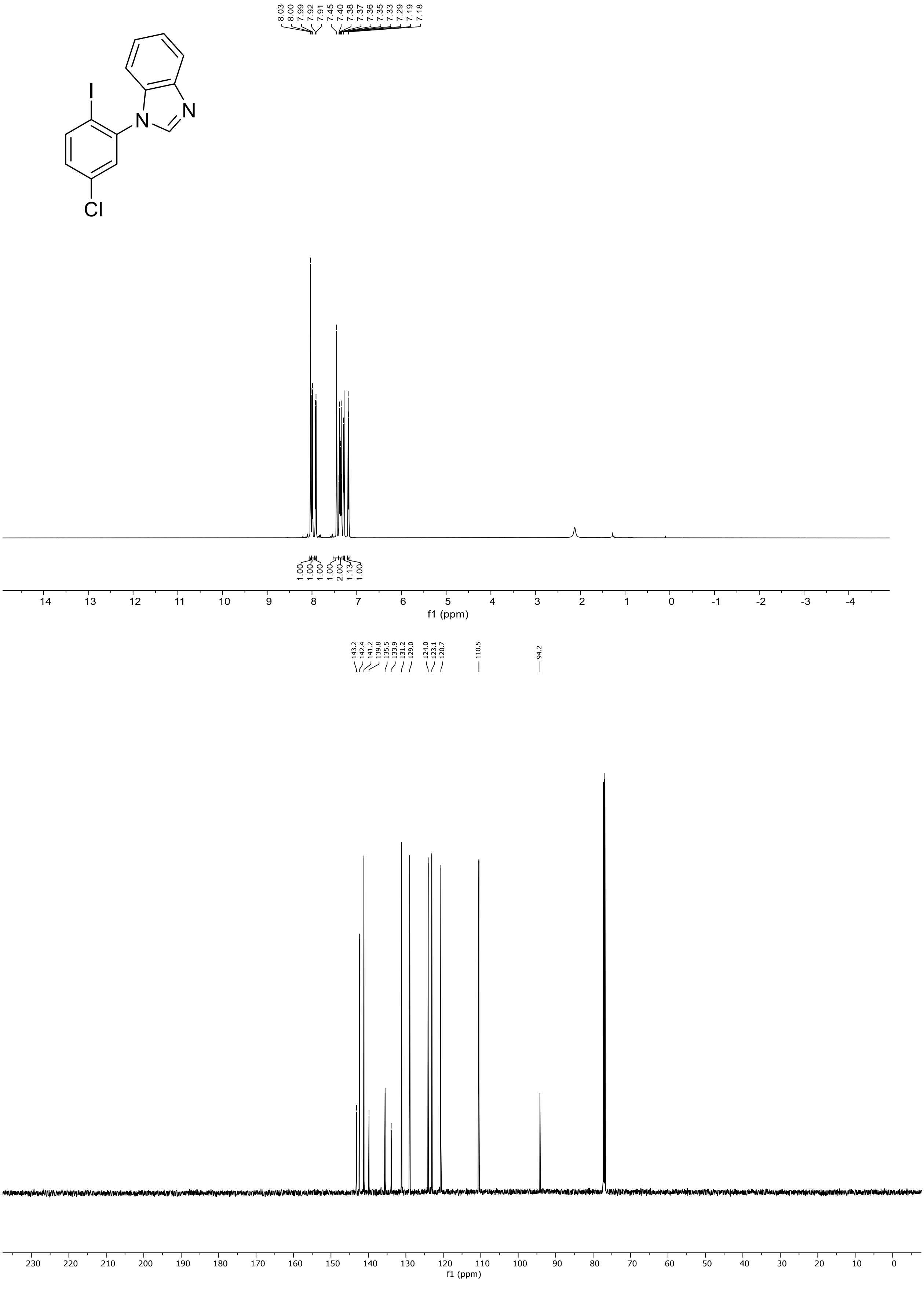

Figure S5: ^{1}H and ^{13}C NMR spectra of 1-(5-chloro-2-iodophenyl)-1*H*-benzo[*d*]imidazole (**4ah**) in $CDCl_3$.

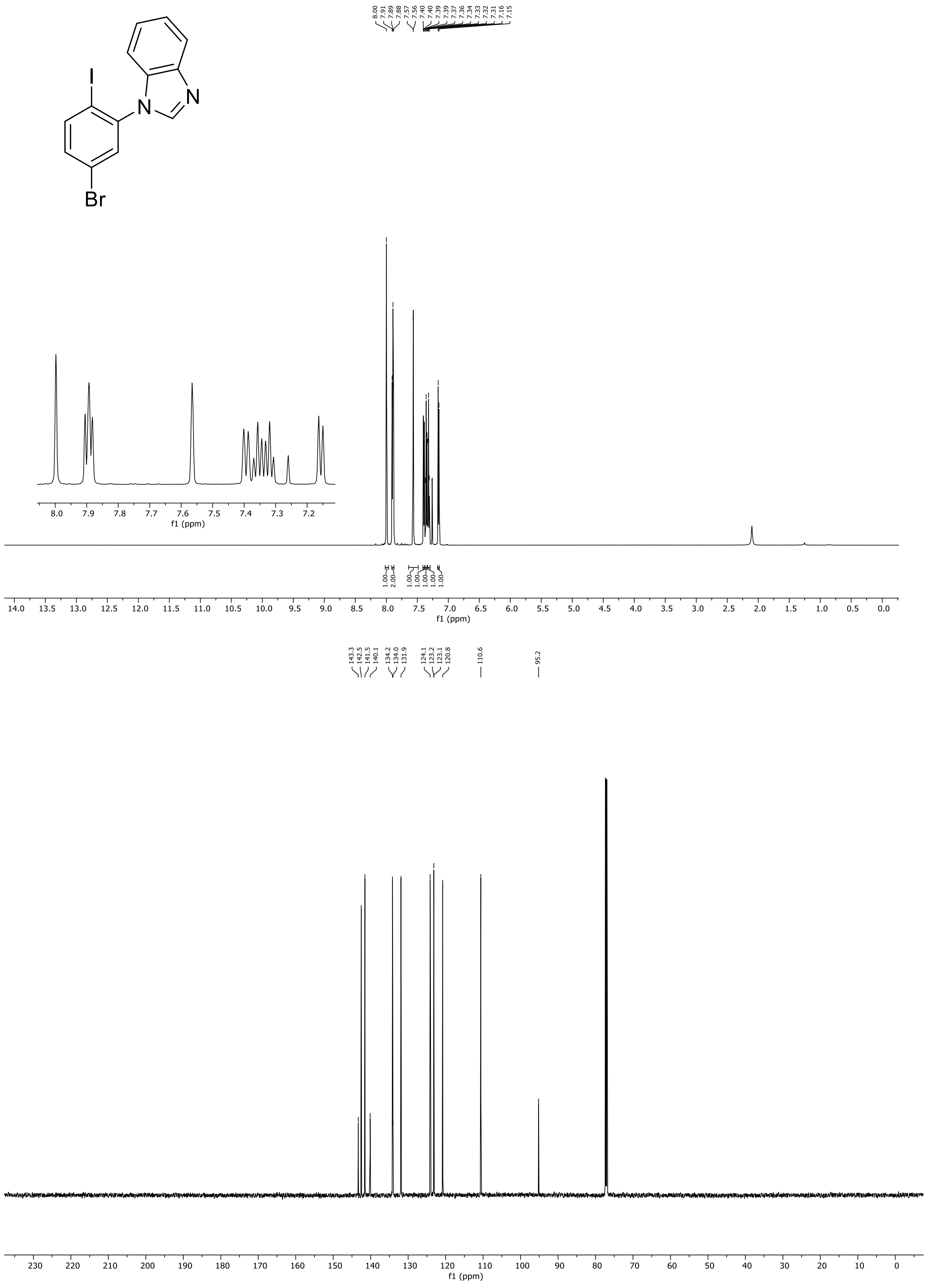

Figure S6: ^{1}H and ^{13}C NMR spectra of 1-(5-bromo-2-iodophenyl)-1*H*-benzo[*d*]imidazole (**4ai**) in $CDCl_3$.

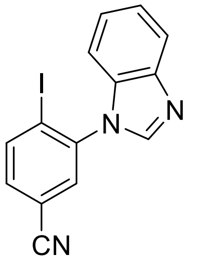

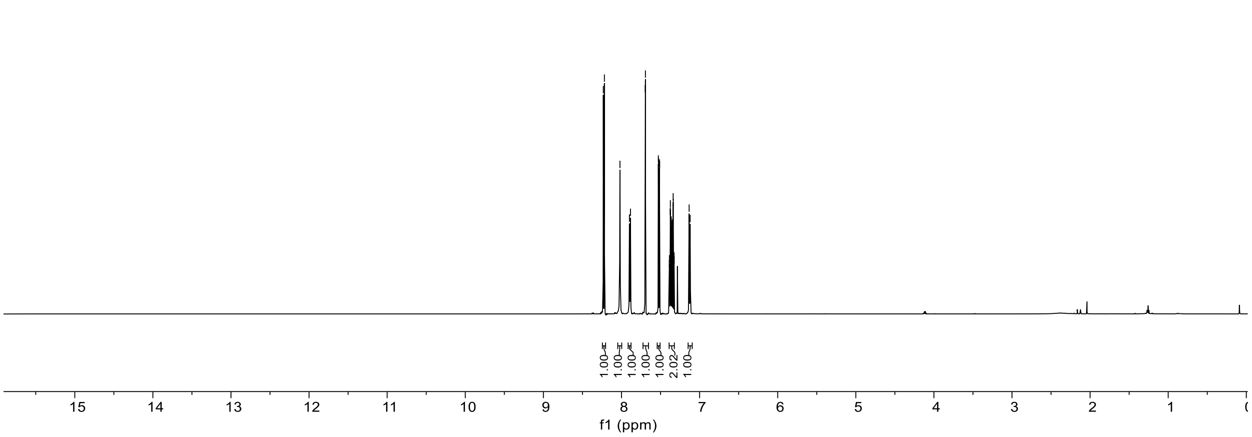

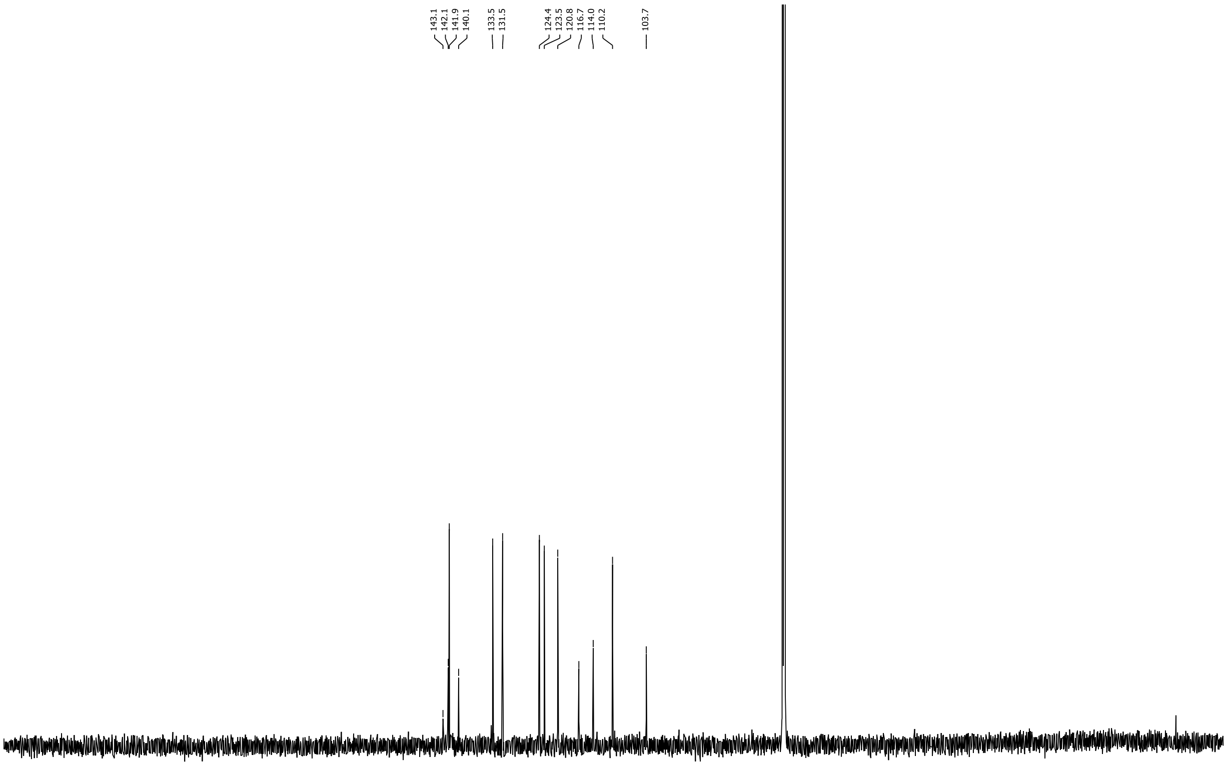

Figure S7: ^{1}H and ^{13}C NMR spectra of 3-(1*H*-benzo[*d*]imidazol-1-yl)-4-iodobenzonitrile (**4aj**) in $CDCl_3$.

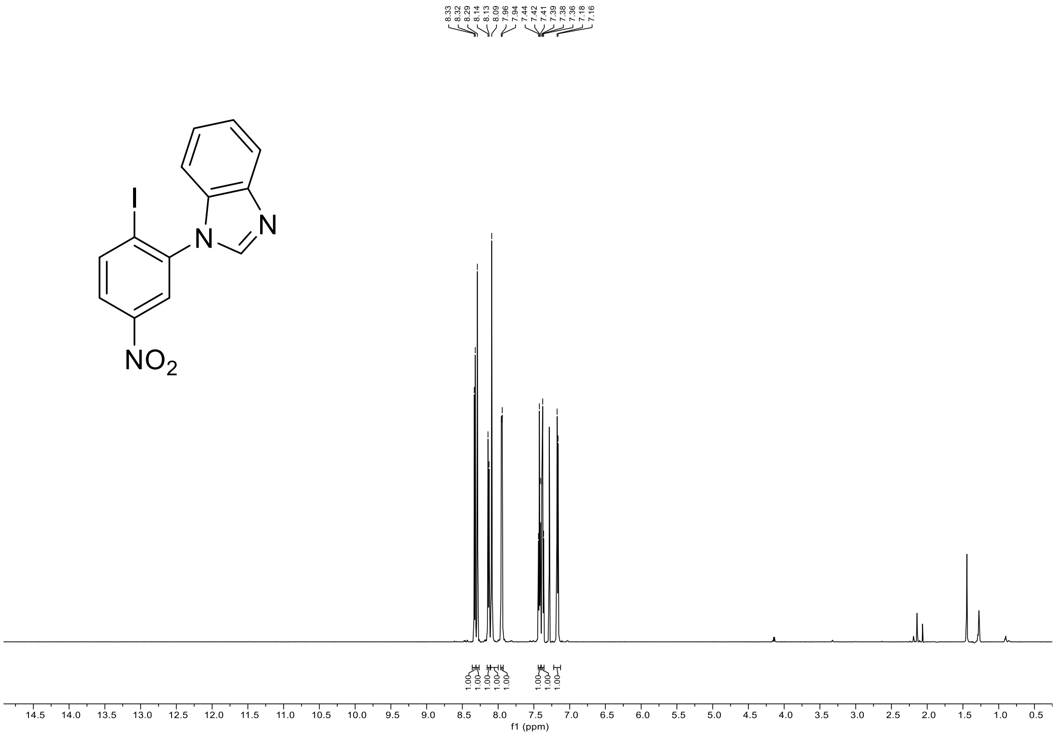

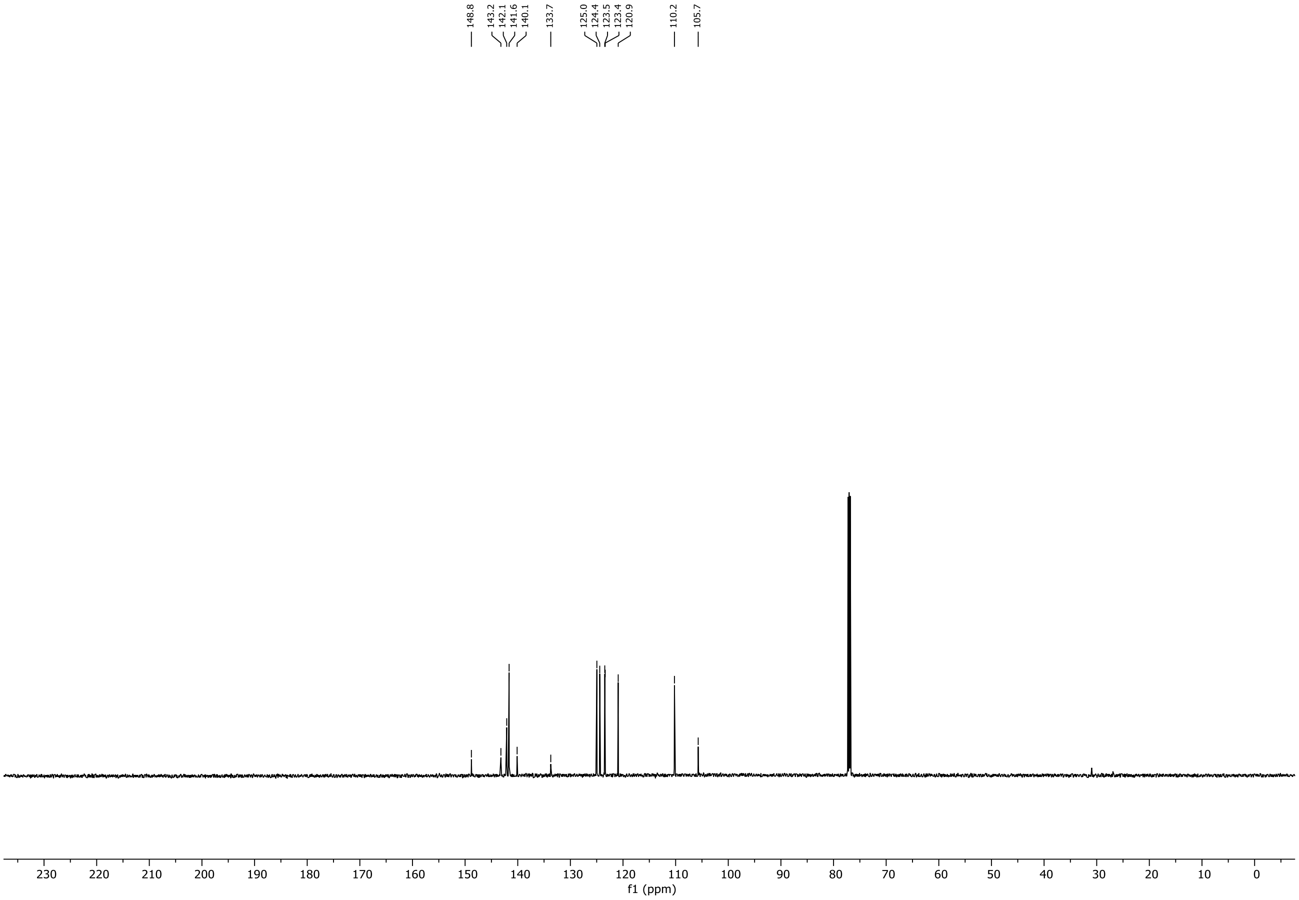

Figure S8: ^{1}H and ^{13}C NMR spectra of 1-(2-iodo-5-nitrophenyl)-1*H*-benzo[*d*]imidazole (**4ak**) in $CDCl_3$.

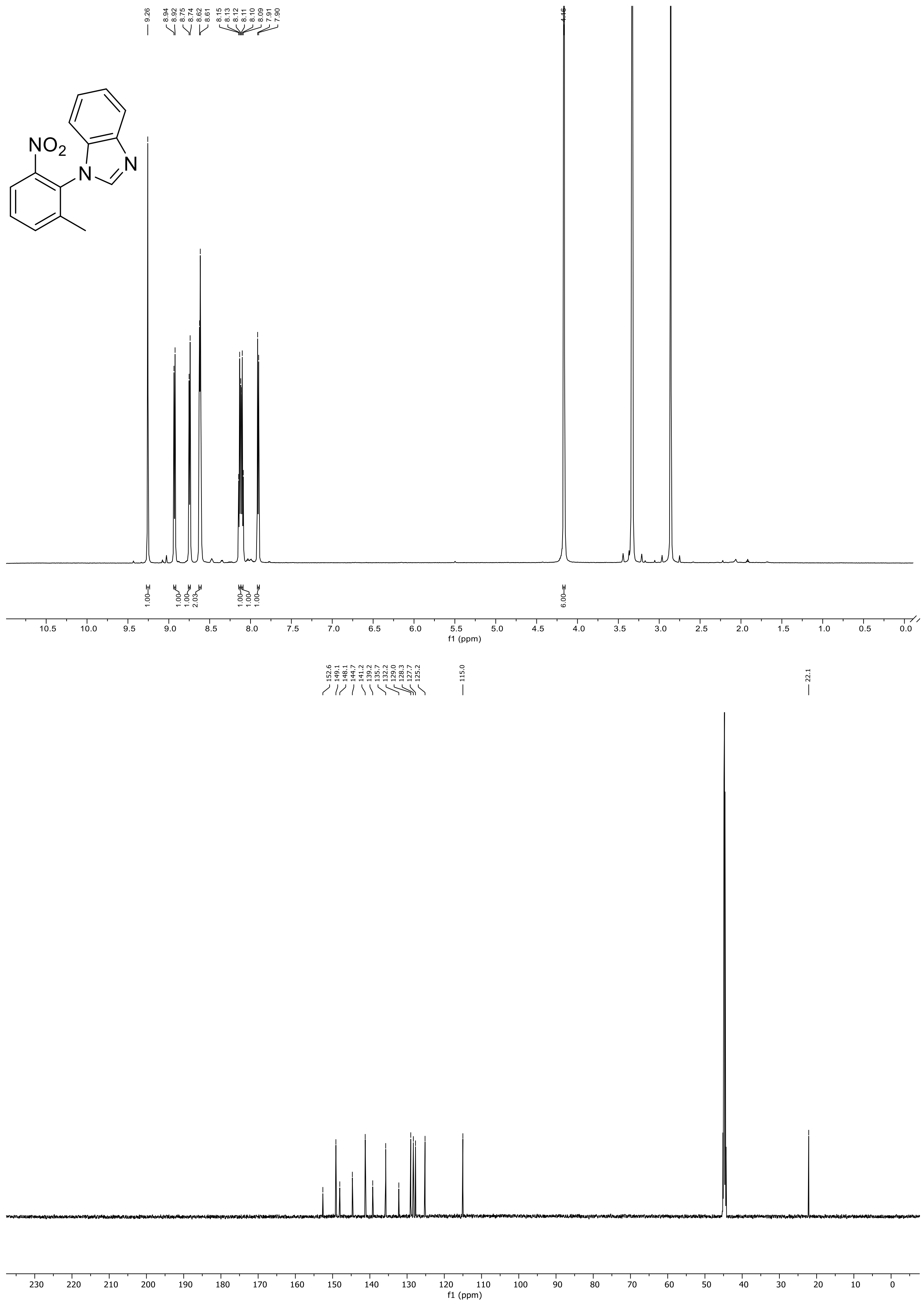

Figure S9: ^{1}H and ^{13}C NMR spectra of 1-(2-methyl-6-nitrophenyl)-1*H*-benzo[*d*]imidazole (**S4al1**) in $CDCl_3$.

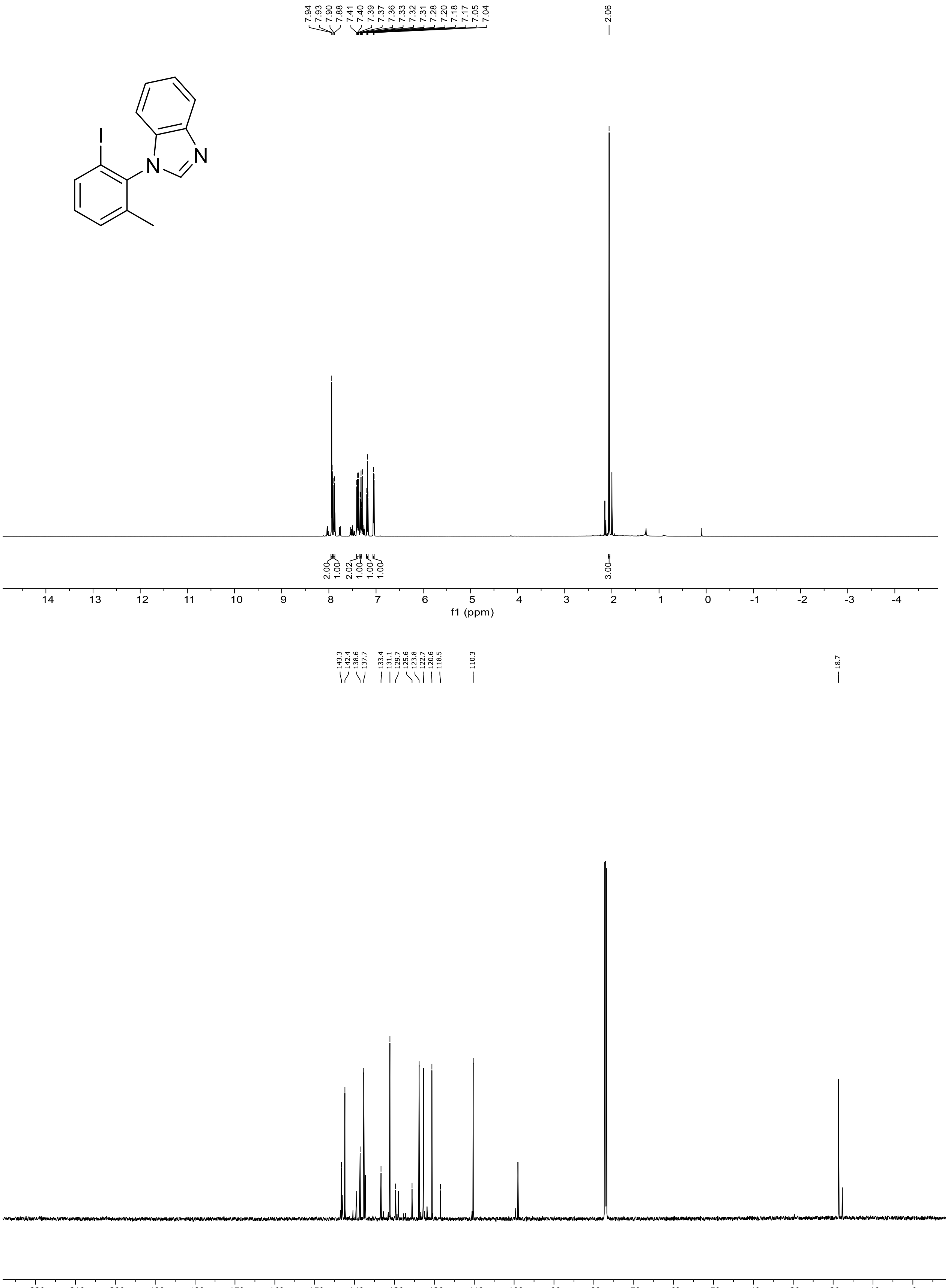

Figure S10: ^{1}H and ^{13}C NMR spectra of 1-(2-iodo-6-methylphenyl)-1*H*-benzo[*d*]imidazole (**4al**) in $CDCl_3$ (two rotamers).

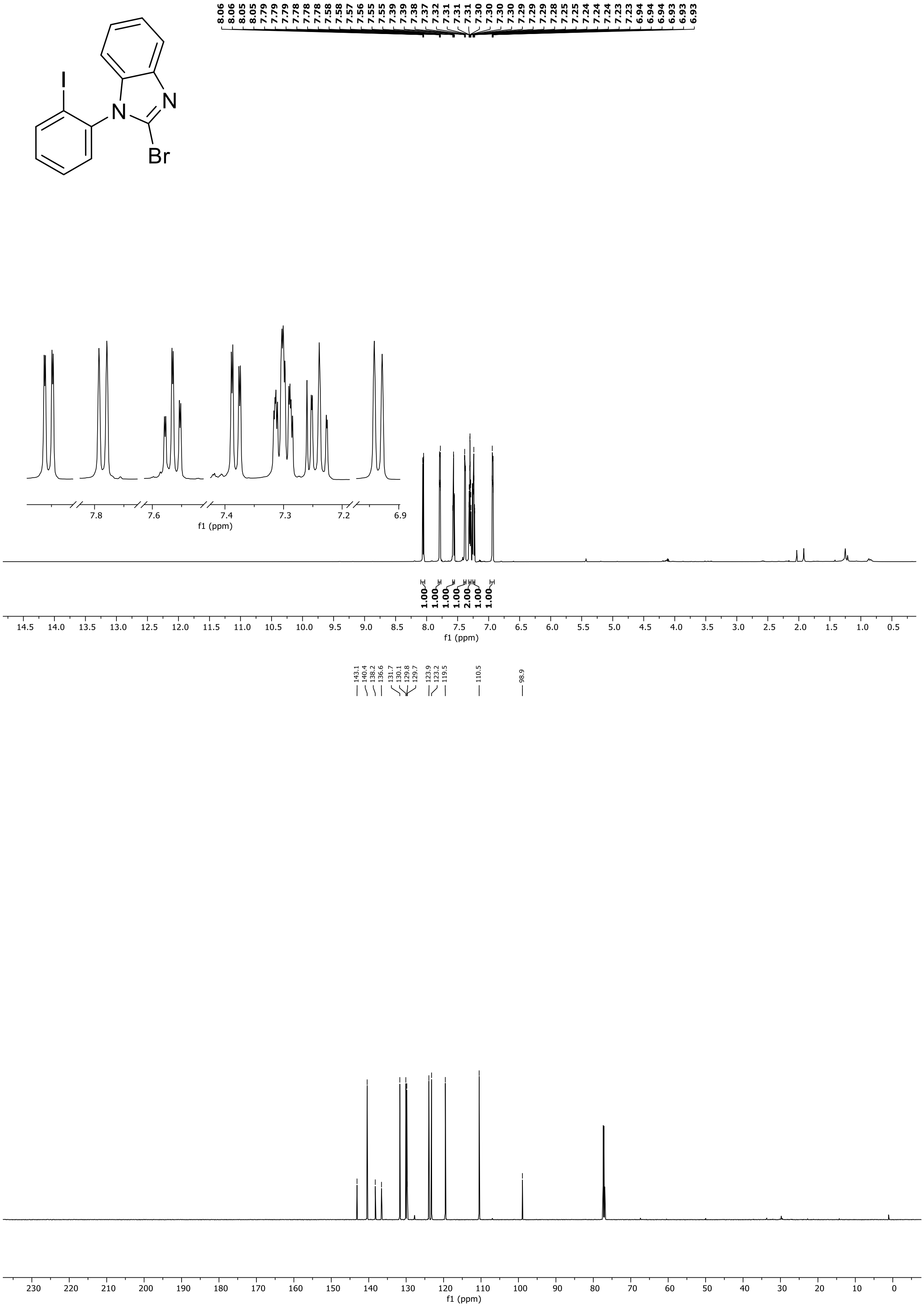

Figure S11: ^{1}H and ^{13}C NMR spectra of 2-bromo-1-(2-iodophenyl)-1*H*-benzo[*d*]imidazole (**4am**) in $CDCl_3$.

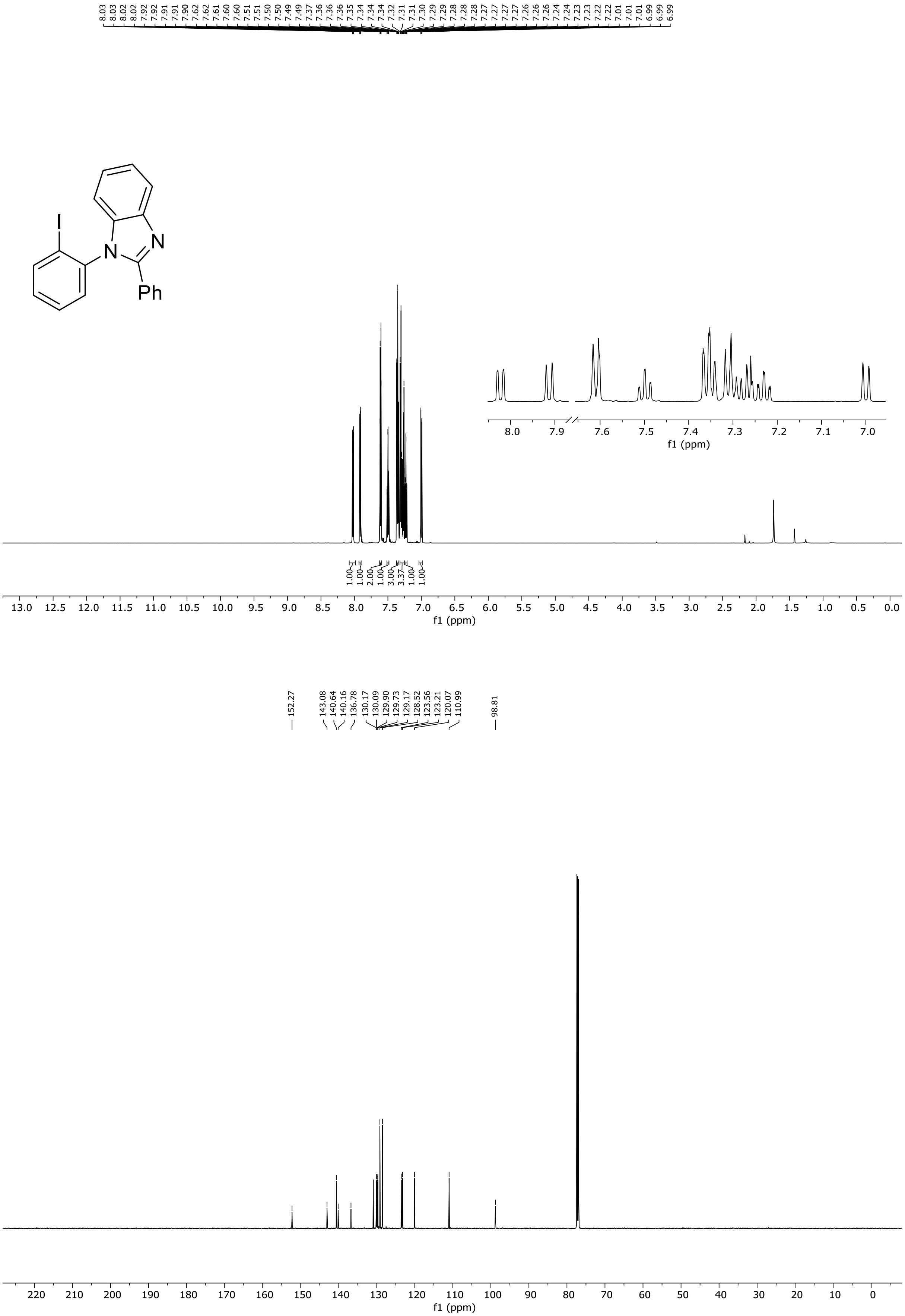

Figure S12: ^{1}H and ^{13}C NMR spectra of 1-(2-iodophenyl)-2-phenyl-1*H*-benzo[*d*]imidazole (**4an**) in $CDCl_3$.

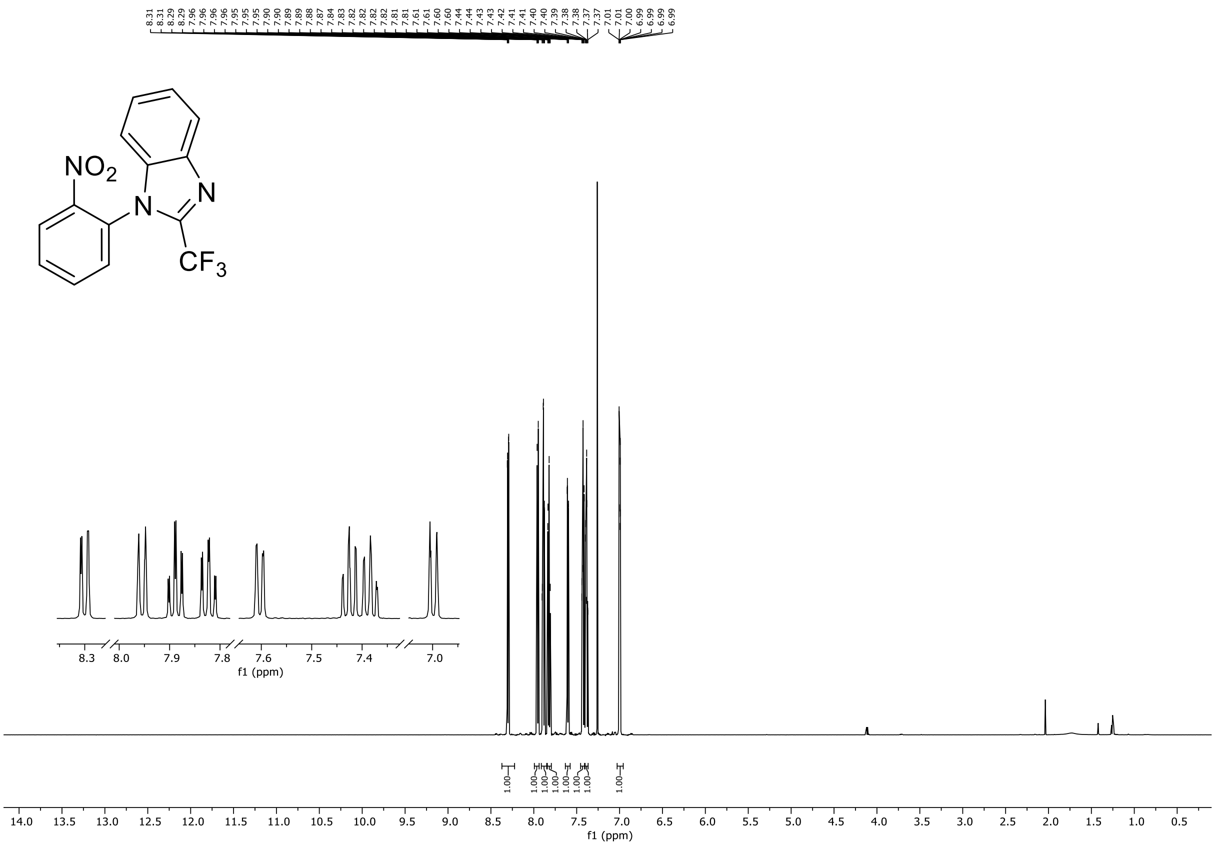

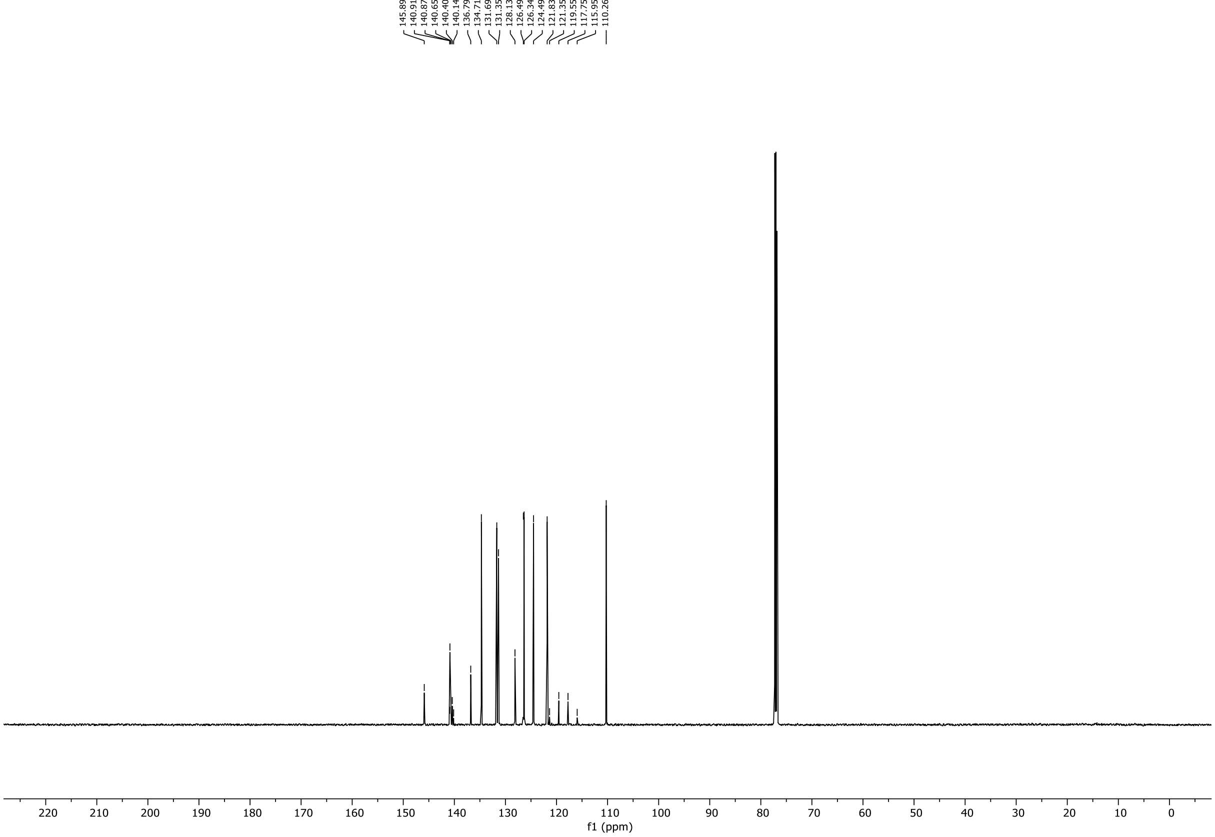

Figure S13: ^{1}H and ^{13}C NMR spectra of 1-(2-nitrophenyl)-2-(trifluoromethyl)-1*H*-benzo[*d*]imidazole (**S4ao1**) in $CDCl_3$.

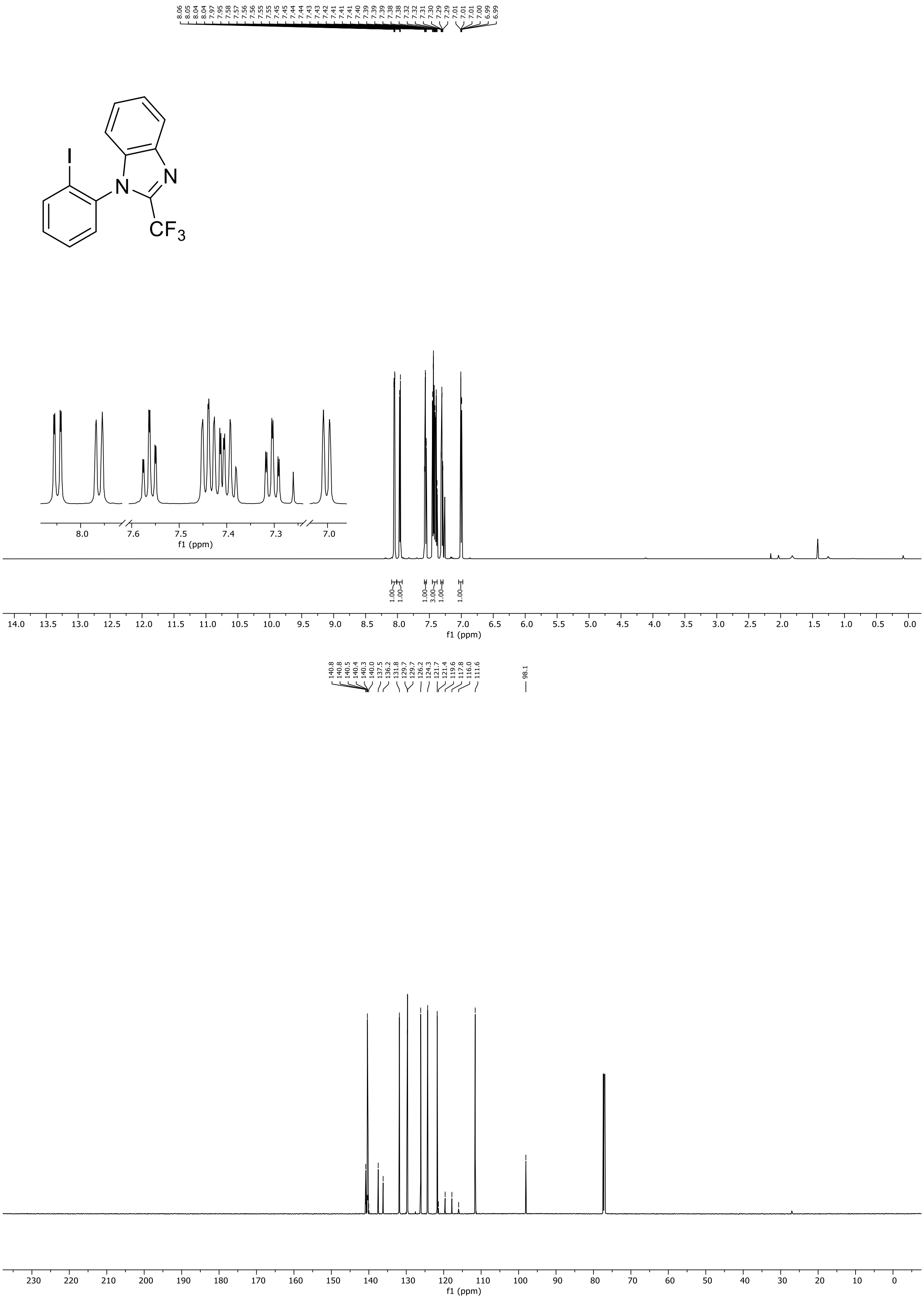

Figure S14: ^{1}H and ^{13}C NMR spectra of 1-(2-iodophenyl)-2-(trifluoromethyl)-1*H*-benzo[*d*]imidazole (**4ao**) in $CDCl_3$.

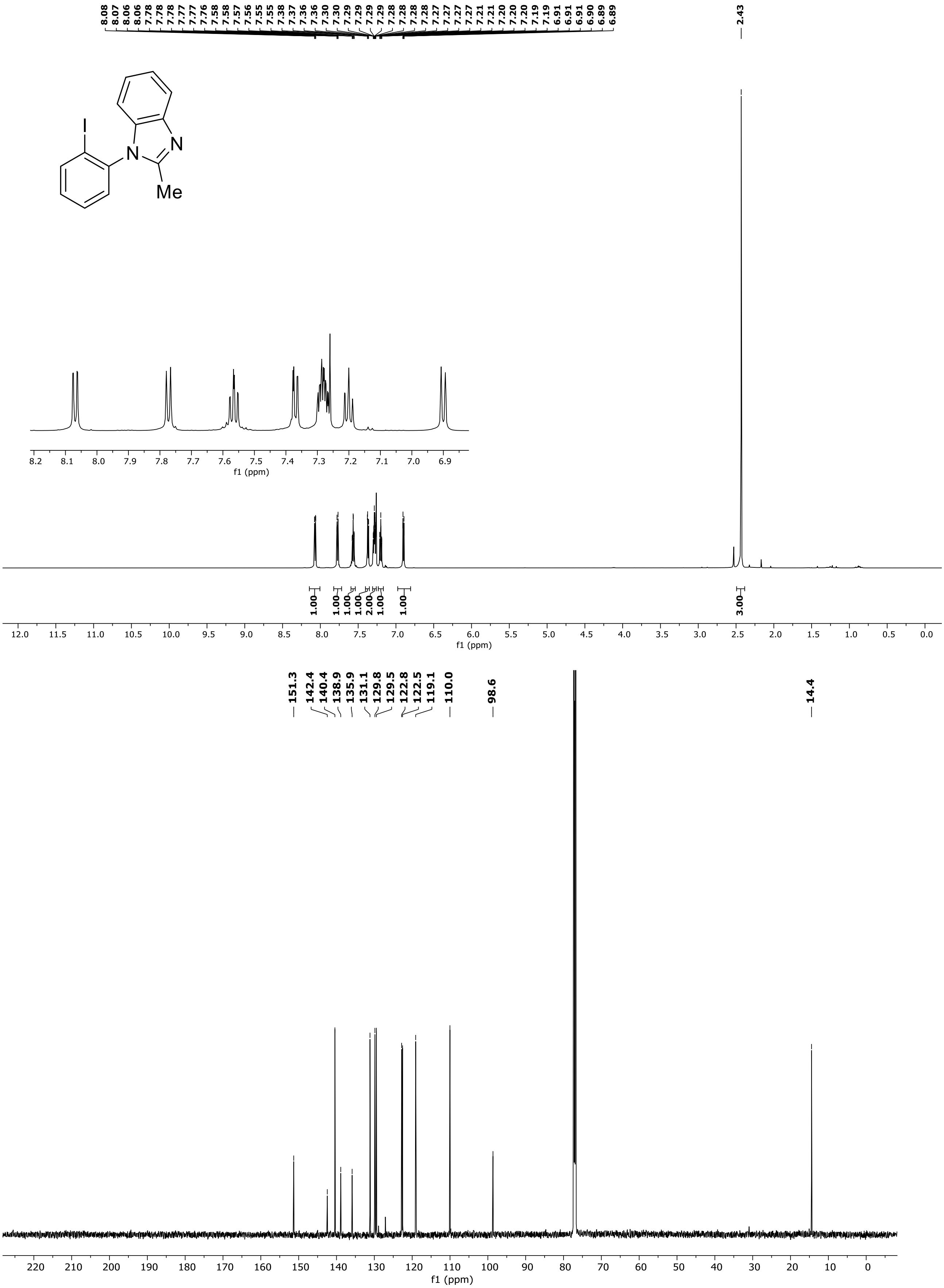

Figure S15: ^{1}H and ^{13}C NMR spectra of 1-(2-iodophenyl)-2-methyl-1*H*-benzo[*d*]imidazole (**4ap**) in $CDCl_3$.

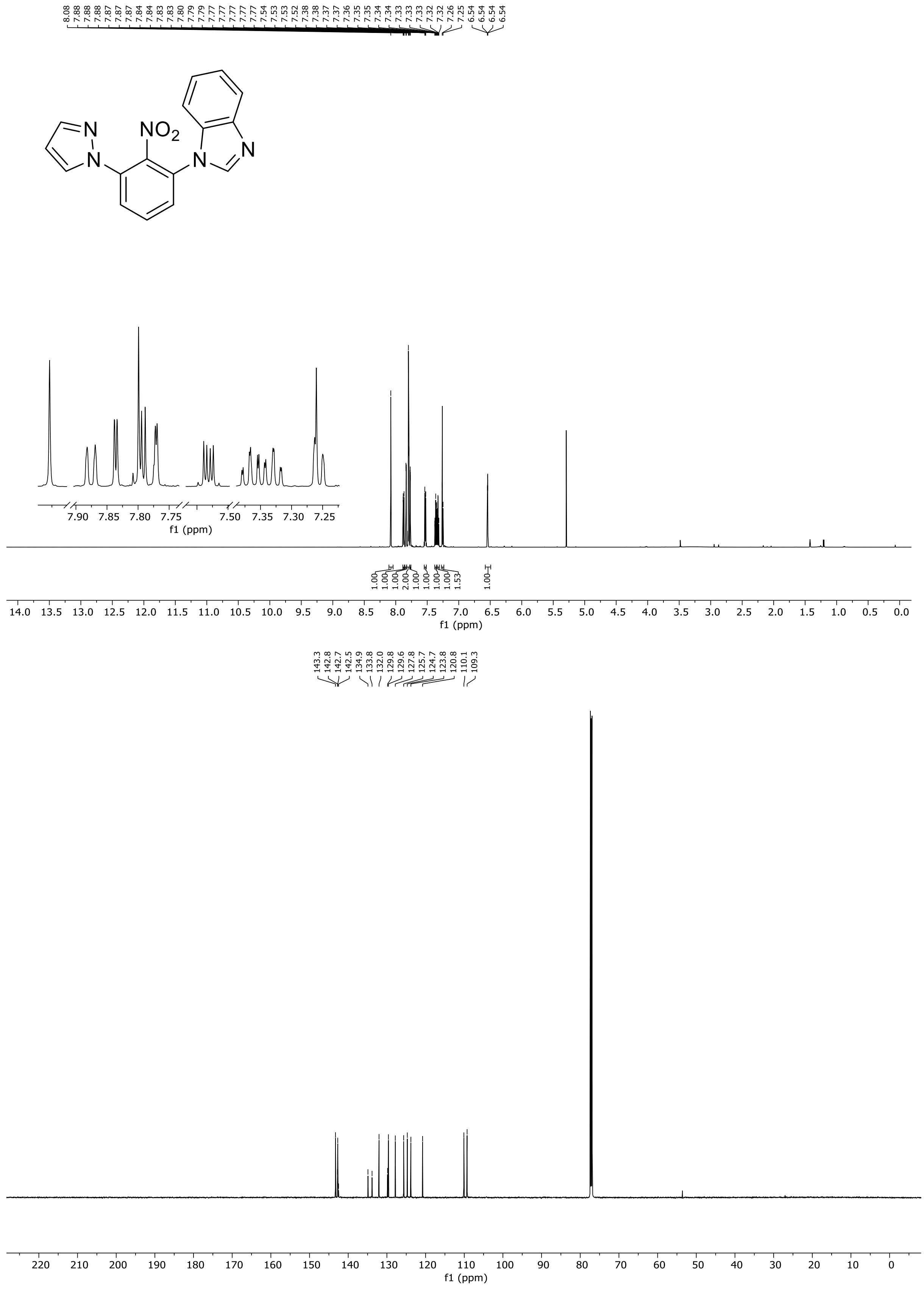

Figure S16: ^{1}H and ^{13}C NMR spectra of 1-(2-nitro-3-(1*H*-pyrazol-1-yl)phenyl)-1*H*-benzo[*d*]imidazole (**S4ba1**) in $CDCl_3$.

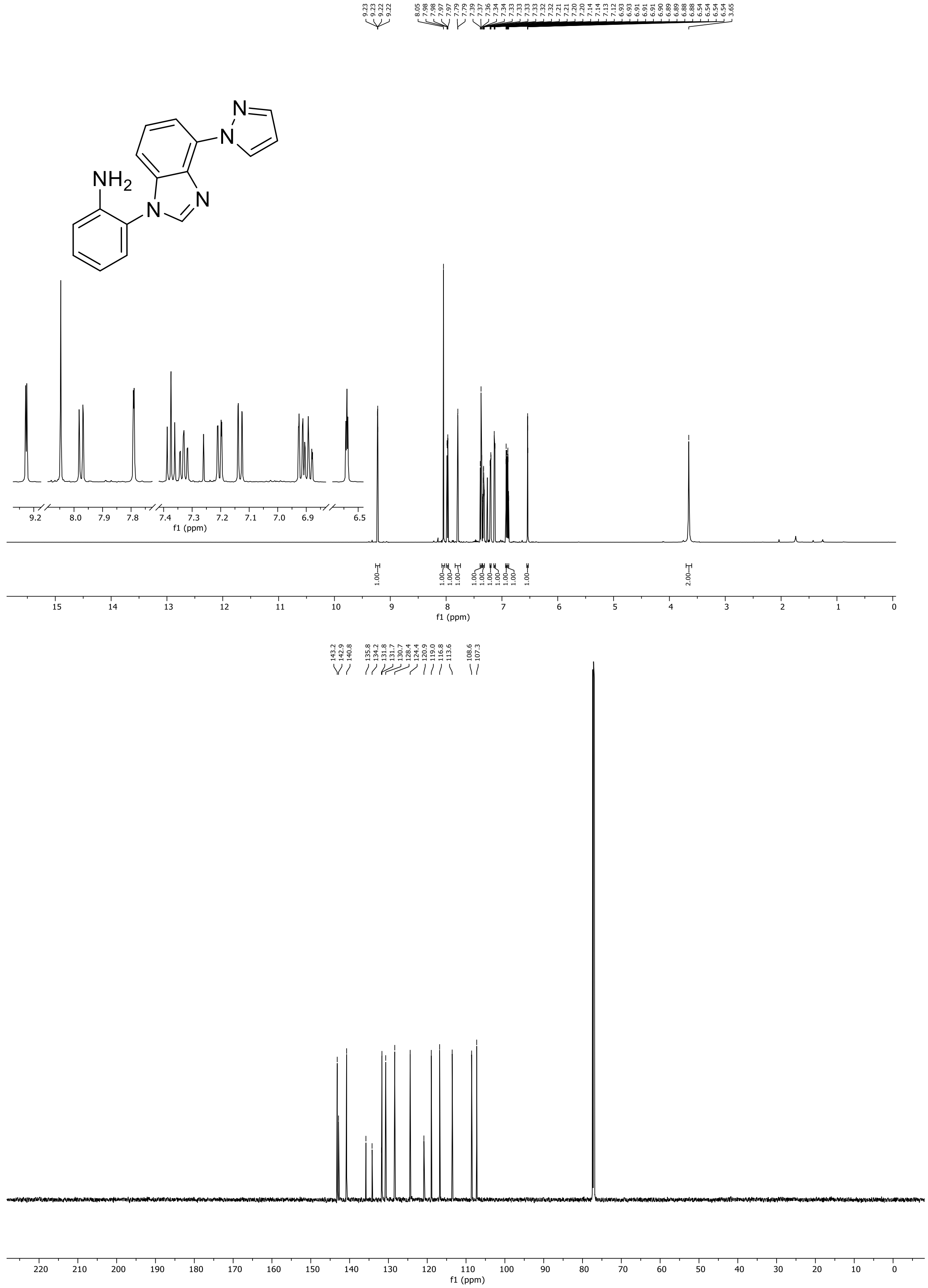

Figure S17: ^{1}H and ^{13}C NMR spectra of 2-(4-(1*H*-pyrazol-1-yl)-1*H*-benzo[*d*]imidazol-1-yl)aniline (**S4aq1**) in $CDCl_3$.

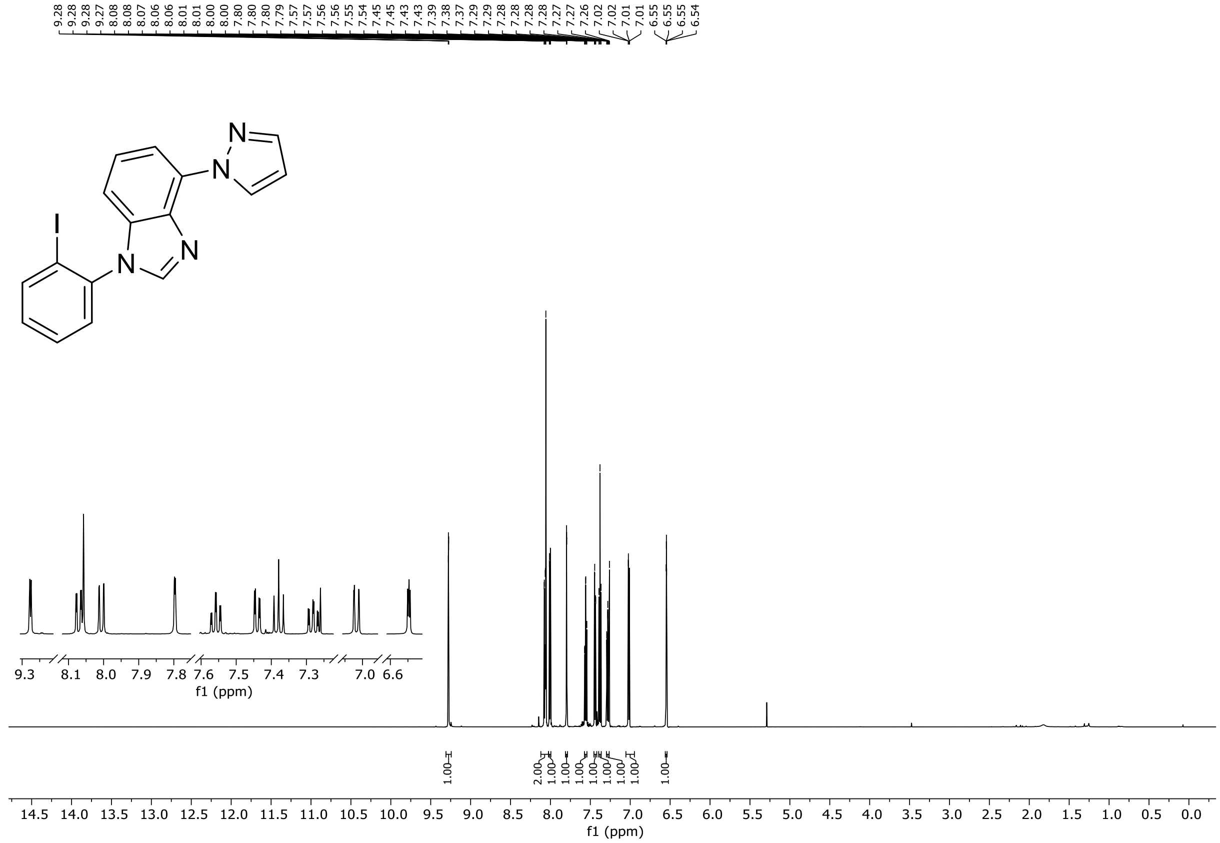

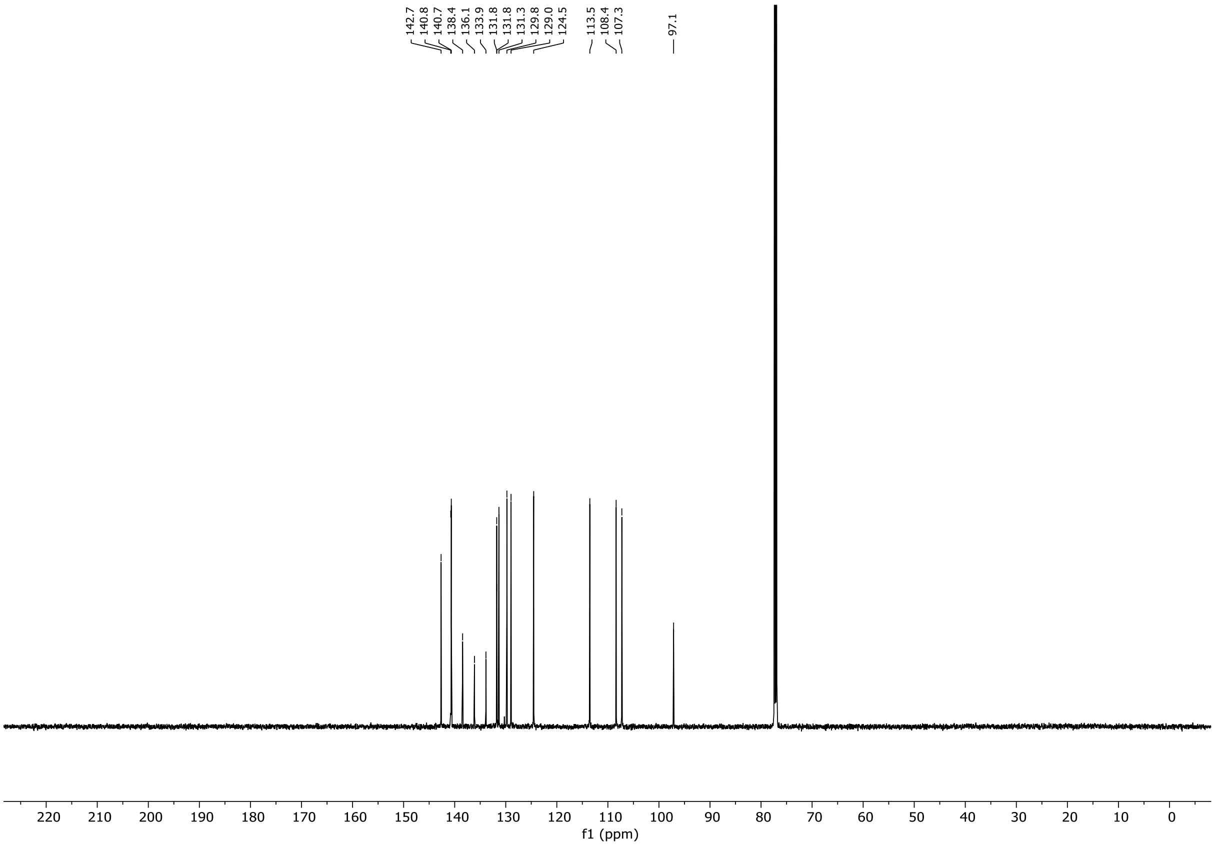

Figure S18: ^{1}H and ^{13}C NMR spectra of 1-(2-iodophenyl)-4-(1*H*-pyrazol-1-yl)-1*H*-benzo[*d*]imidazole (**4aq**) in $CDCl_3$.

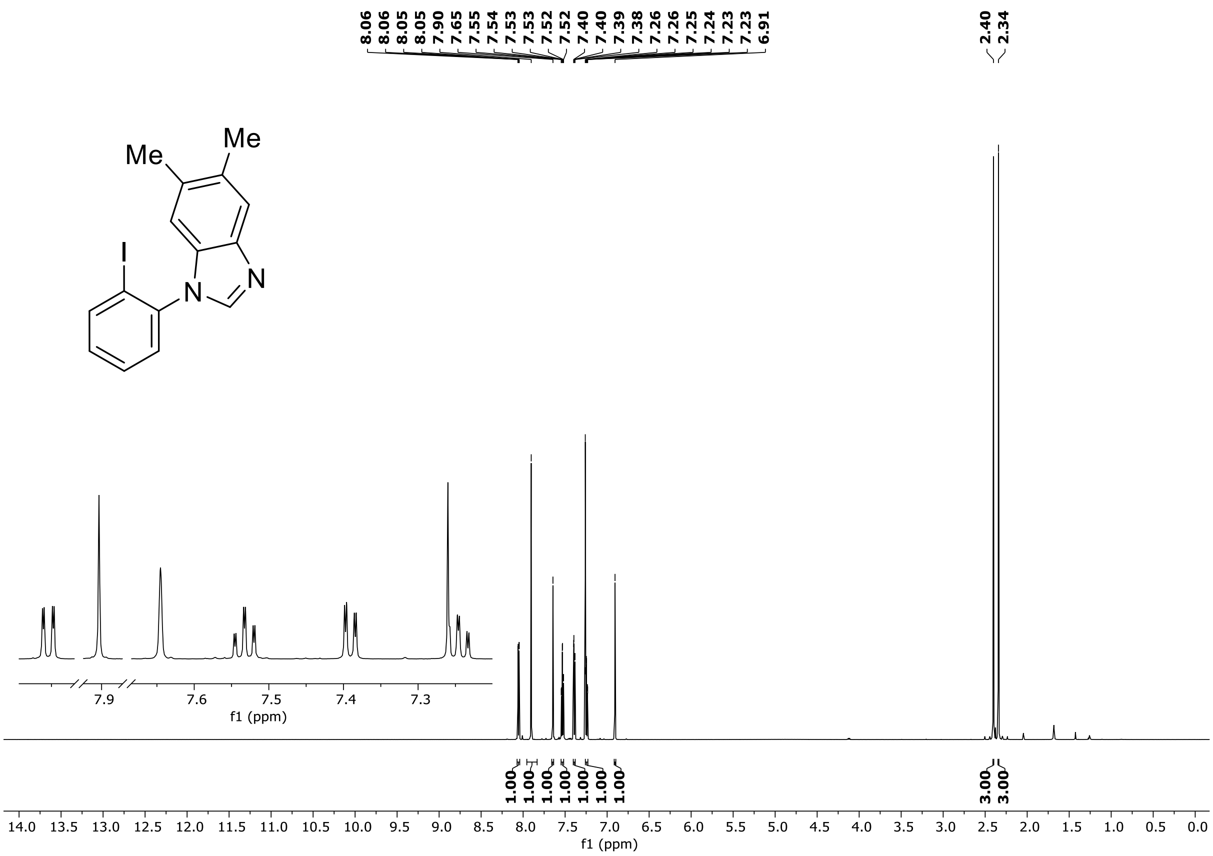

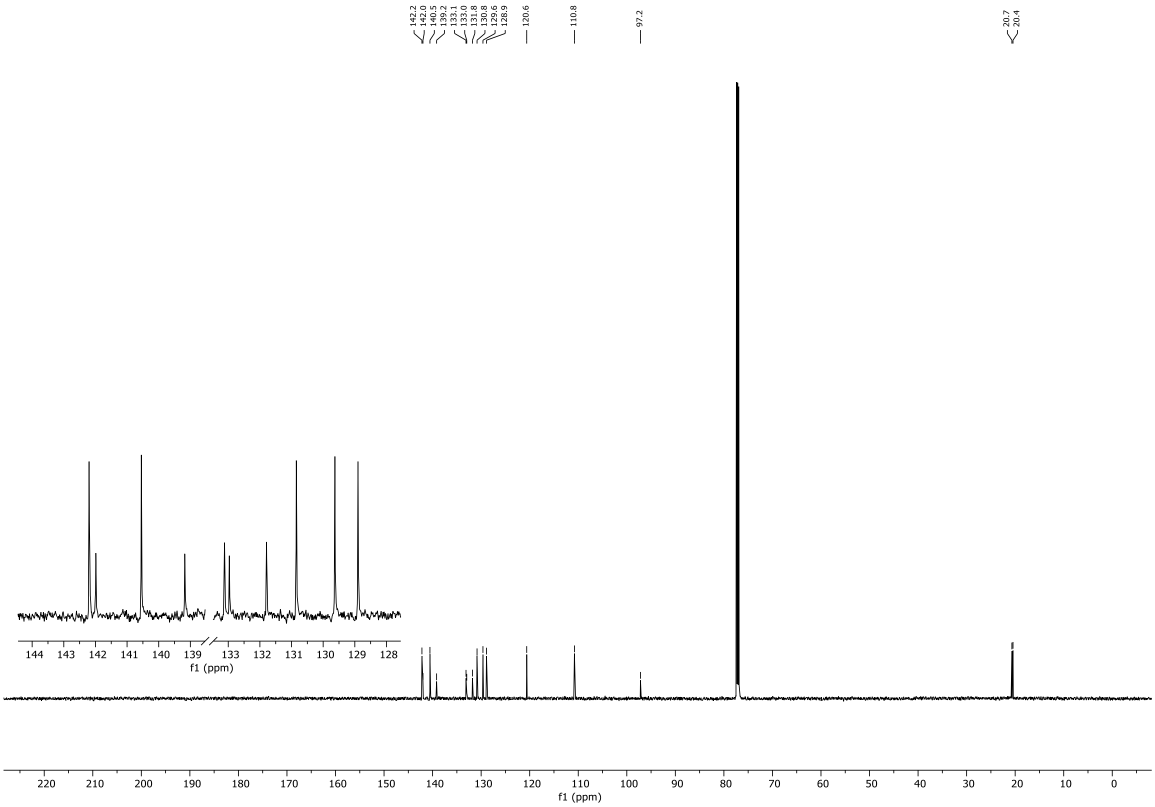

Figure S19: ^{1}H and ^{13}C NMR spectra of 1-(2-iodophenyl)-5,6-dimethyl-1*H*-benzo[*d*]imidazole (**4ar**) in $CDCl_3$.

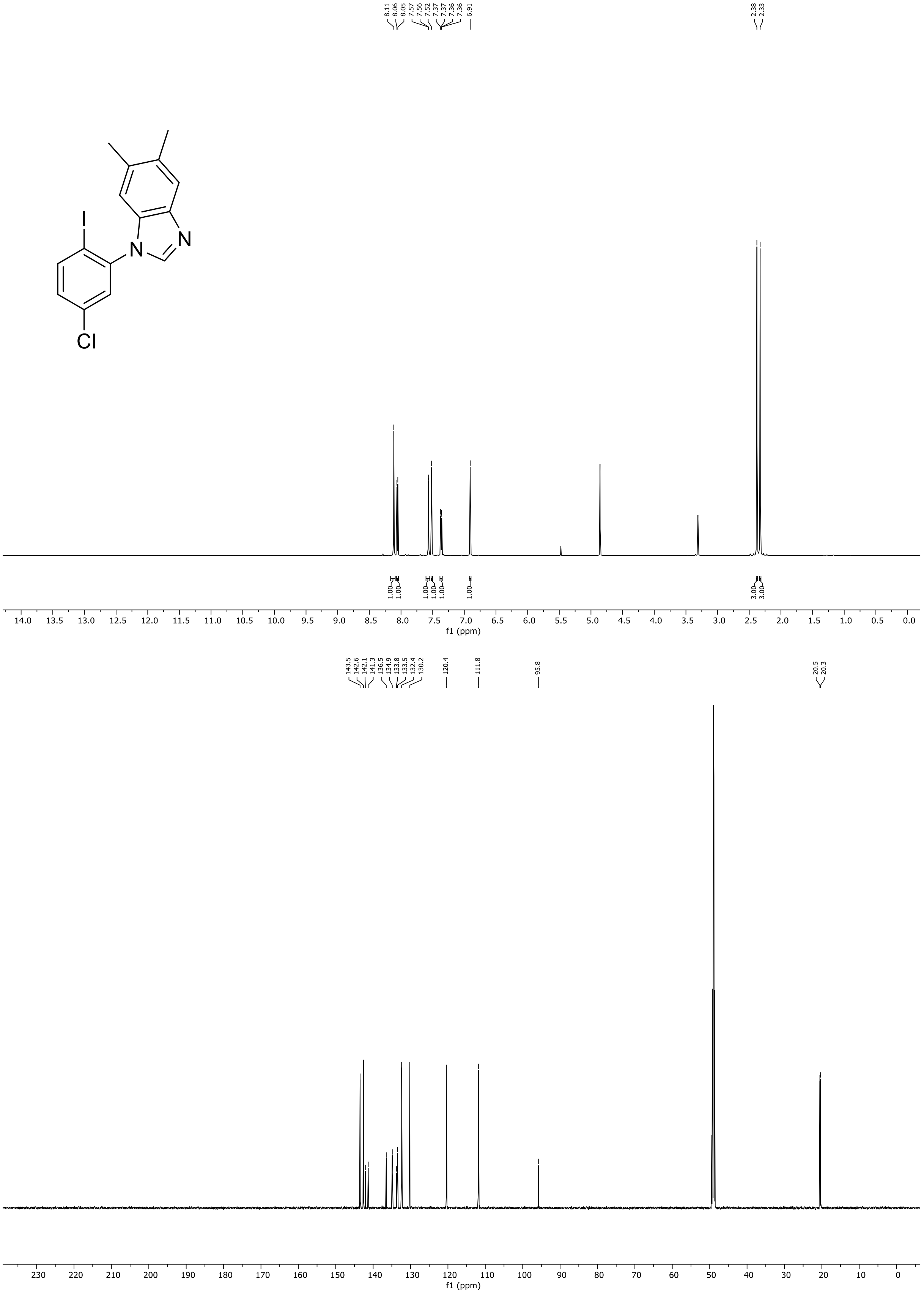

Figure S20: ^{1}H and ^{13}C NMR spectra of 1-(5-chloro-2-iodophenyl)-5,6-dimethyl-1*H*-benzo[*d*]imidazole (**4at**) in $CDCl_3$.

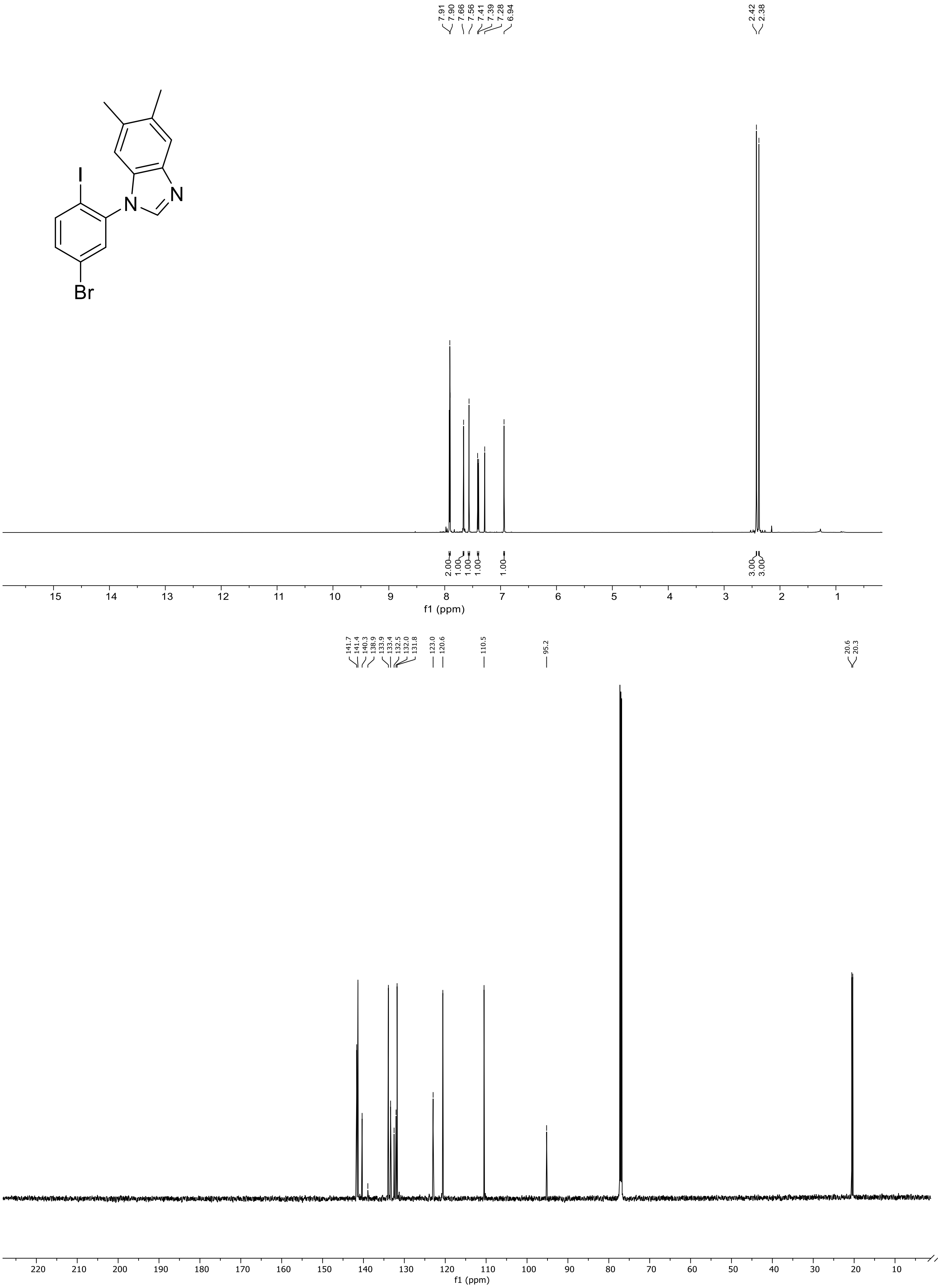

Figure S21: ^{1}H and ^{13}C NMR spectra of 1-(5-bromo-2-iodophenyl)-5,6-dimethyl-1*H*-benzo[*d*]imidazole (**4au**) in $CDCl_3$.

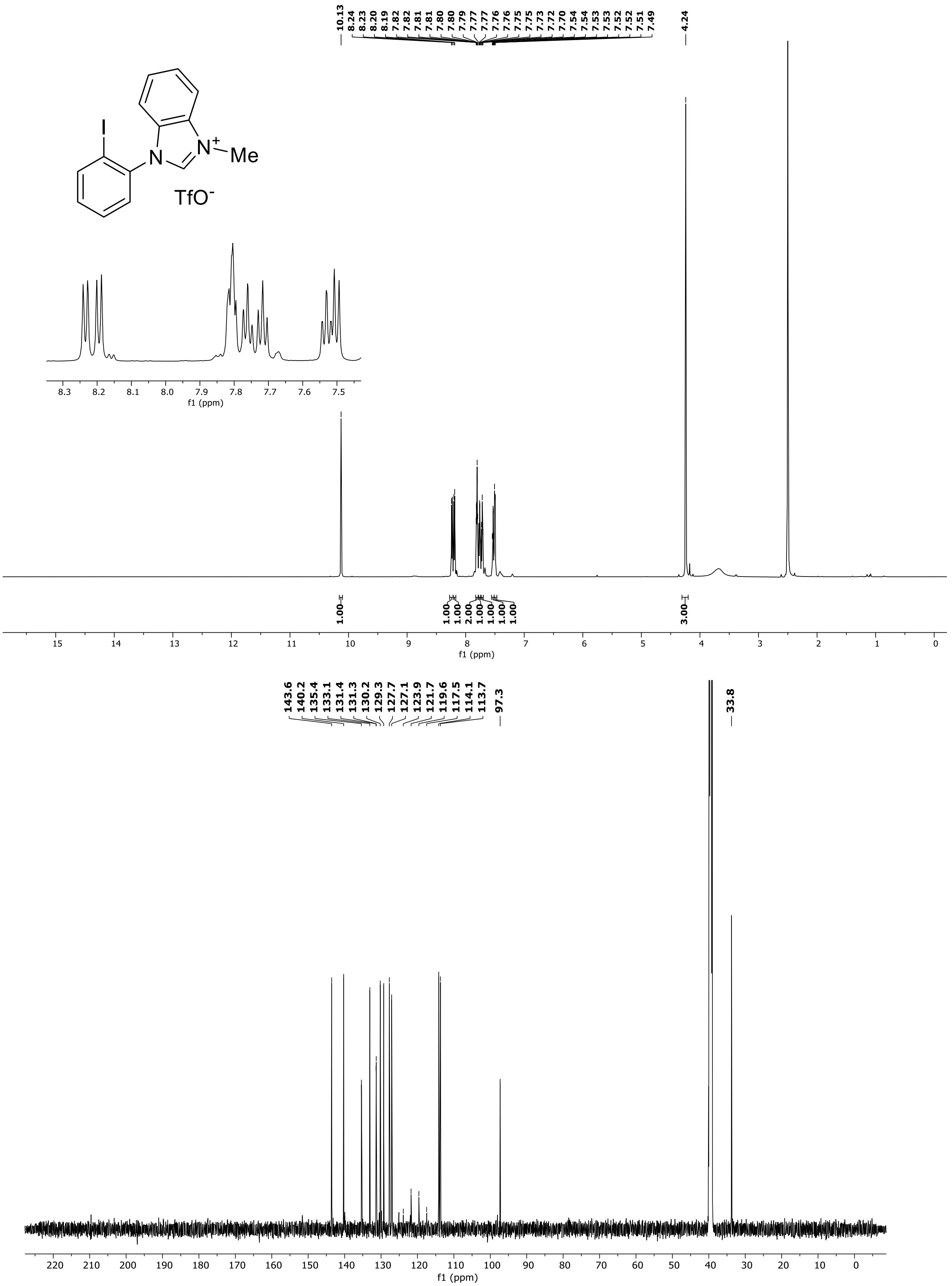

Figure S22: ^{1}H and ^{13}C NMR spectra of 1-(2-iodophenyl)-3-methyl-1*H*-benzo[*d*]imidazol-3-ium triflate (**4av**) in $CDCl_3$.

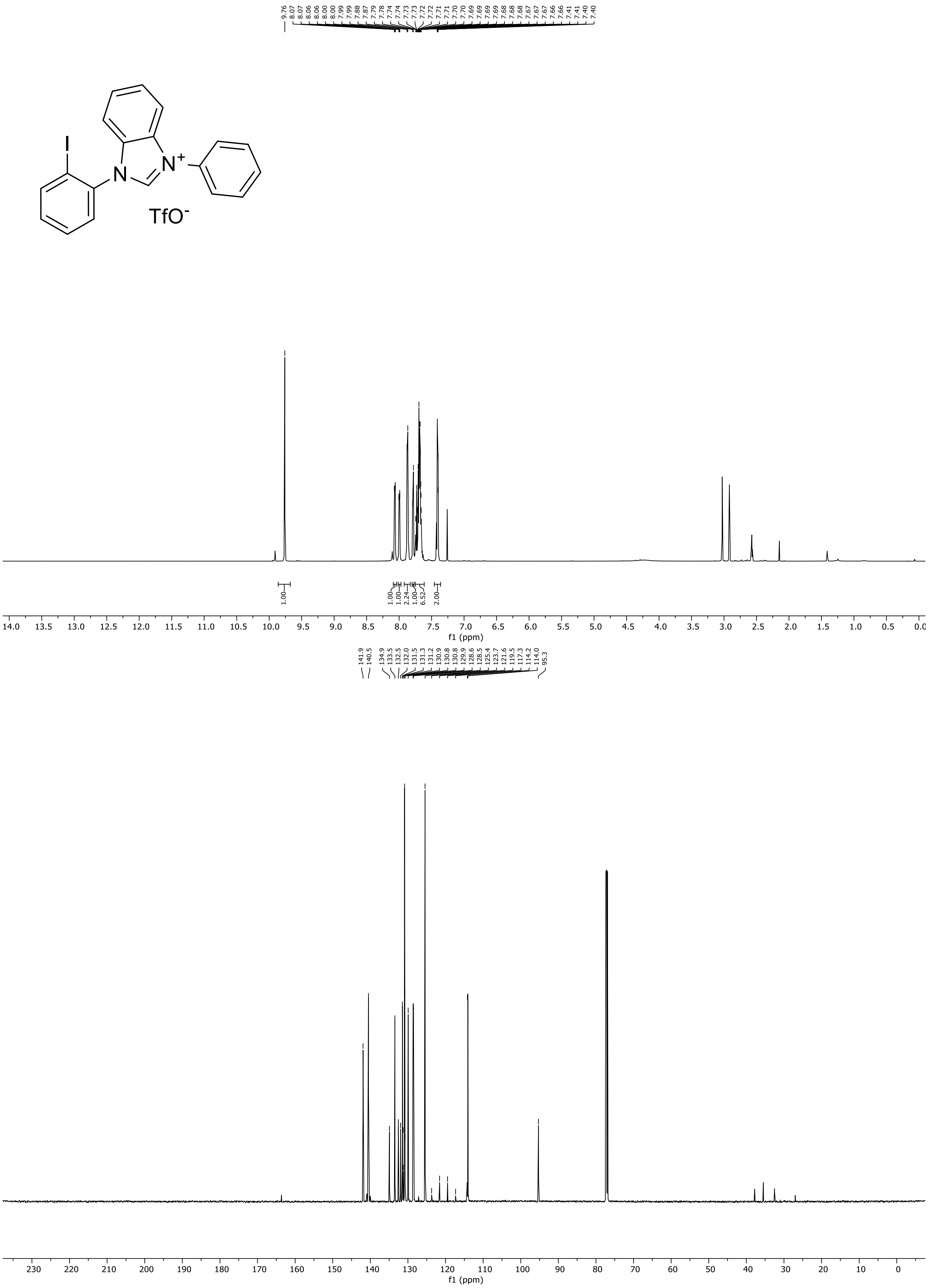

Figure S23: ^{1}H and ^{13}C NMR spectra of 1-(2-iodophenyl)-3-phenyl-1*H*-benzo[*d*]imidazol-3-ium triflate (**4aw**) in $CDCl_3$.

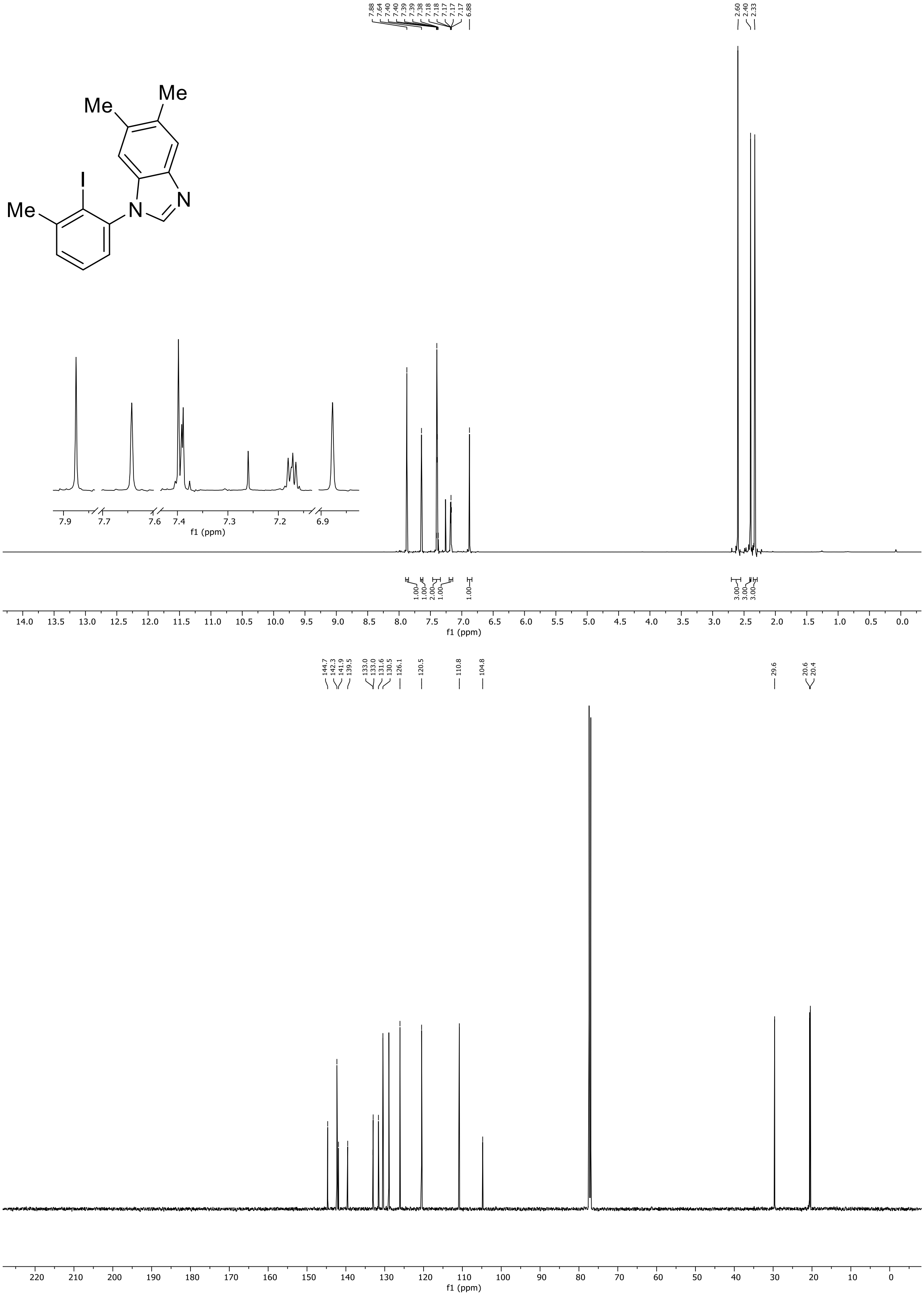

Figure S24: ^{1}H and ^{13}C NMR spectra of 1-(2-iodo-3-methylphenyl)-5,6-dimethyl-1*H*-benzo[*d*]imidazole (**S12**) in $CDCl_3$.

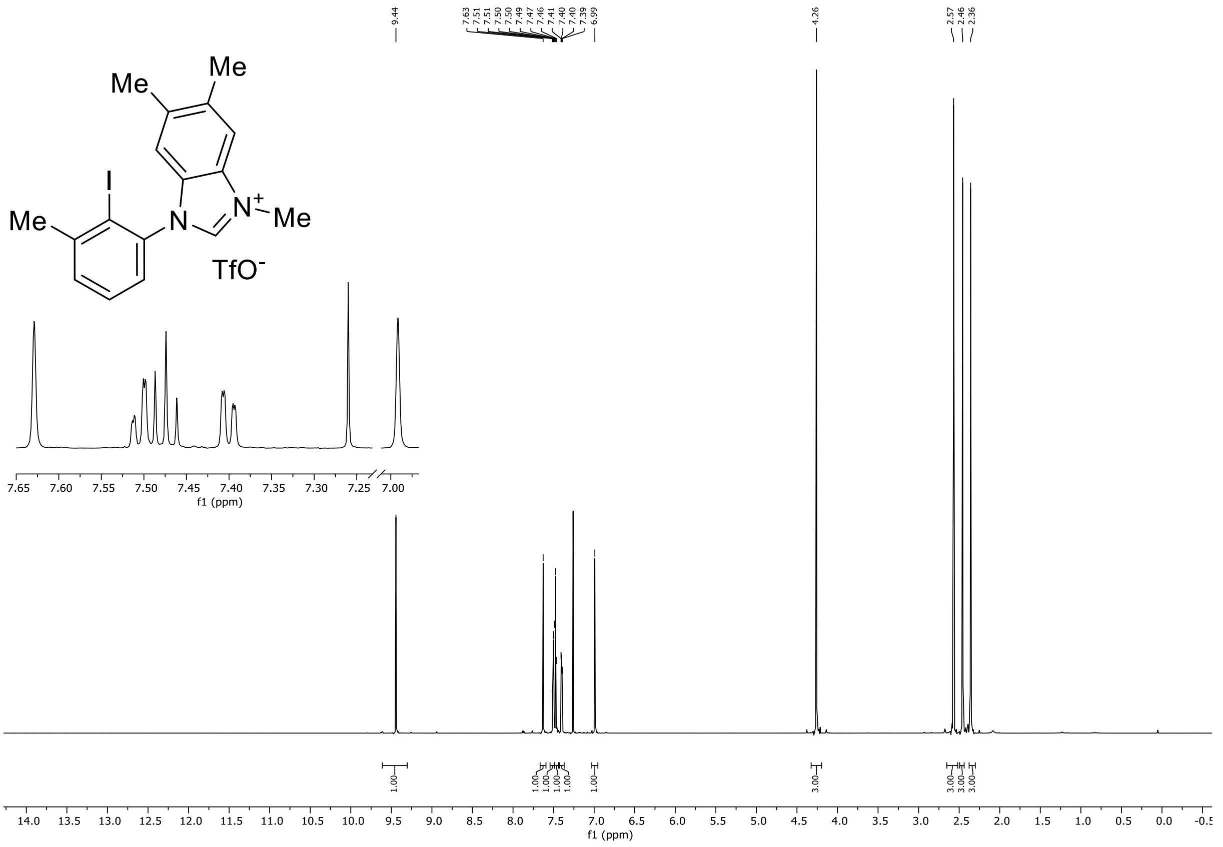

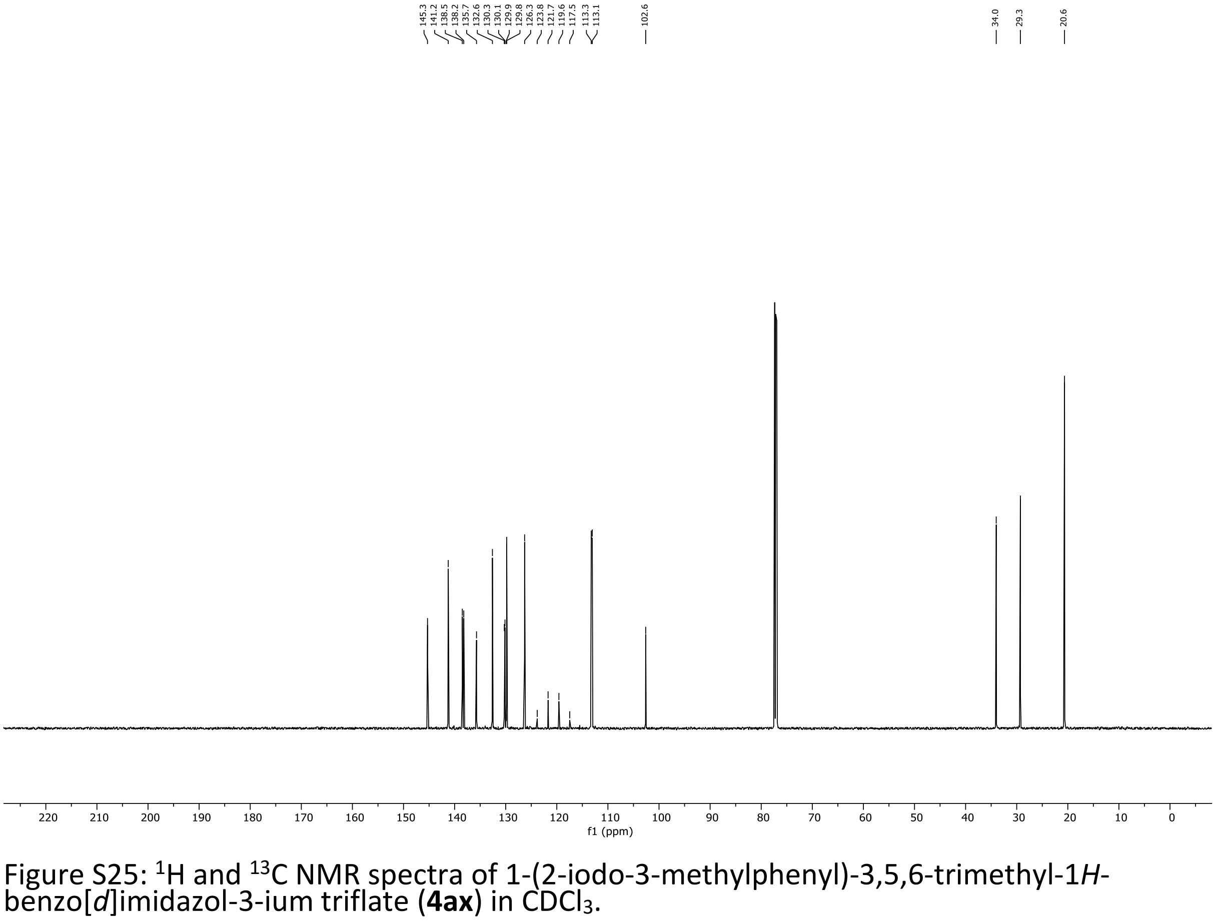

Figure S25: ^{1}H and ^{13}C NMR spectra of 1-(2-iodo-3-methylphenyl)-3,5,6-trimethyl-1*H*-benzo[*d*]imidazol-3-ium triflate (**4ax**) in $CDCl_3$.

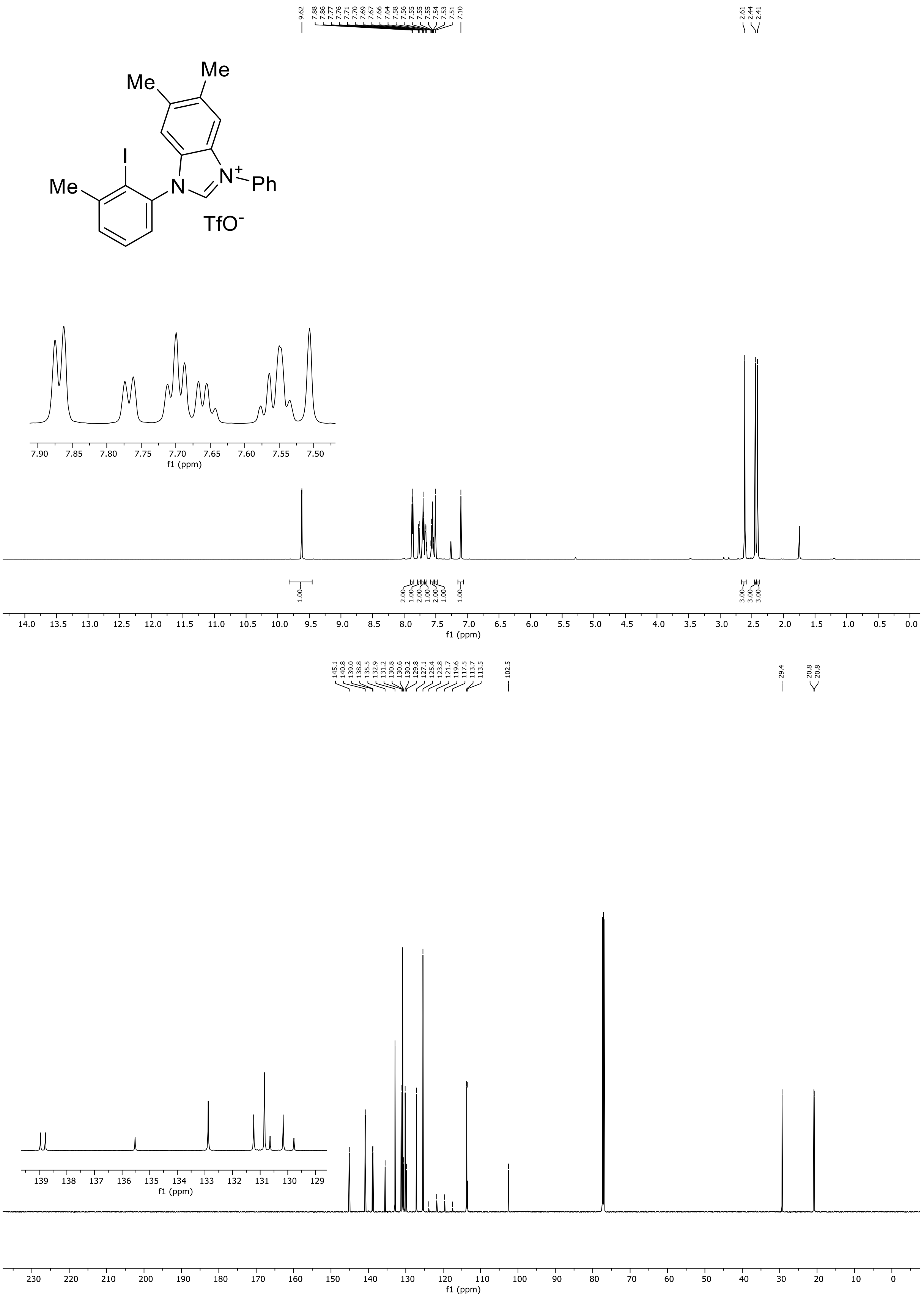

Figure S26: ^{1}H and ^{13}C NMR spectra of 1-(2-iodo-3-methylphenyl)-5,6-dimethyl-3-phenyl-1*H*-benzo[*d*]imidazol-3-ium triflate (**4ay**) in $CDCl_3$.

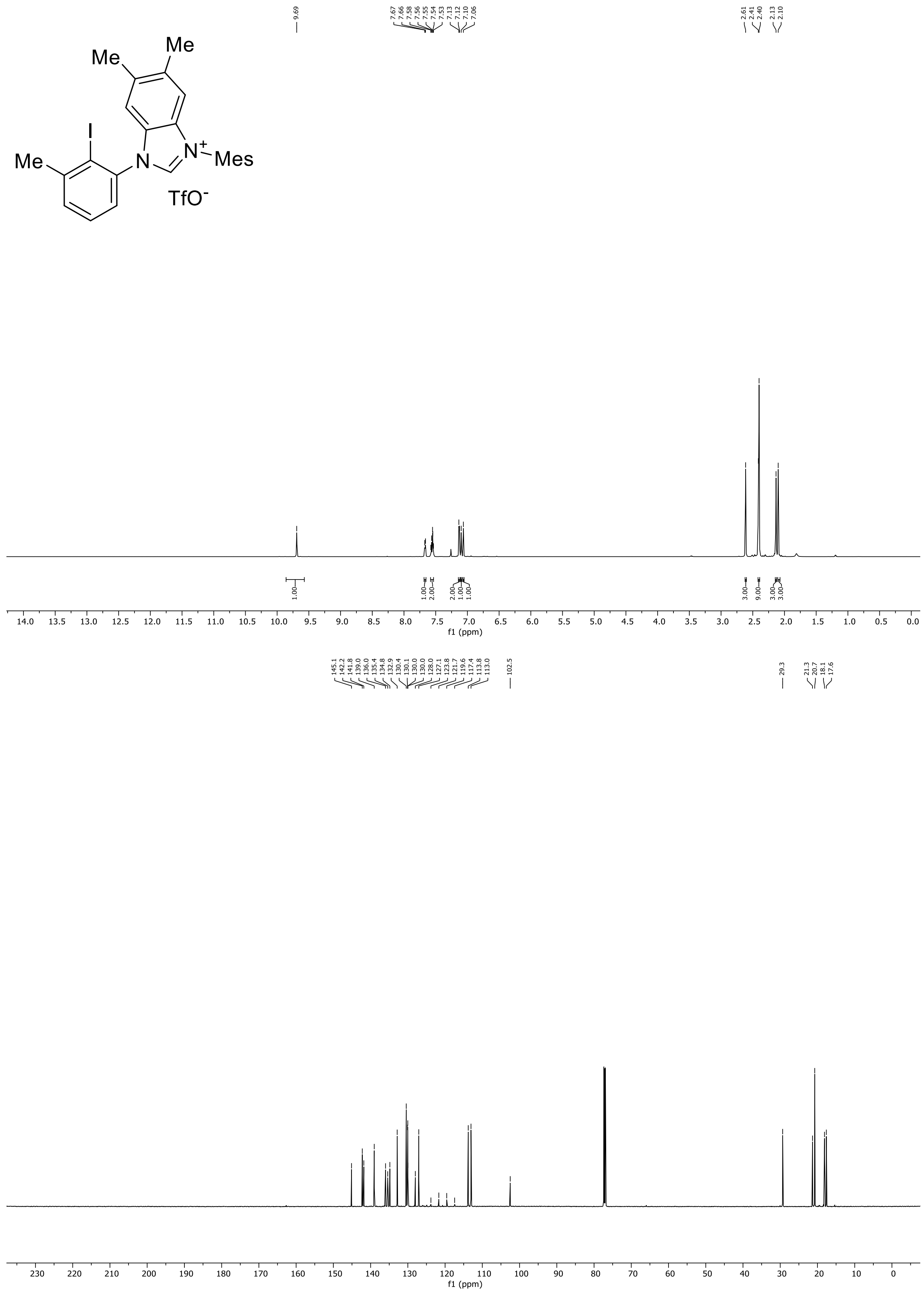

Figure S27: ^{1}H and ^{13}C NMR spectra of 1-(2-iodo-3-methylphenyl)-5,6-dimethyl-3-mesyityl-1*H*-benzo[*d*]imidazol-3-ium triflate (**4az**) in $CDCl_3$.

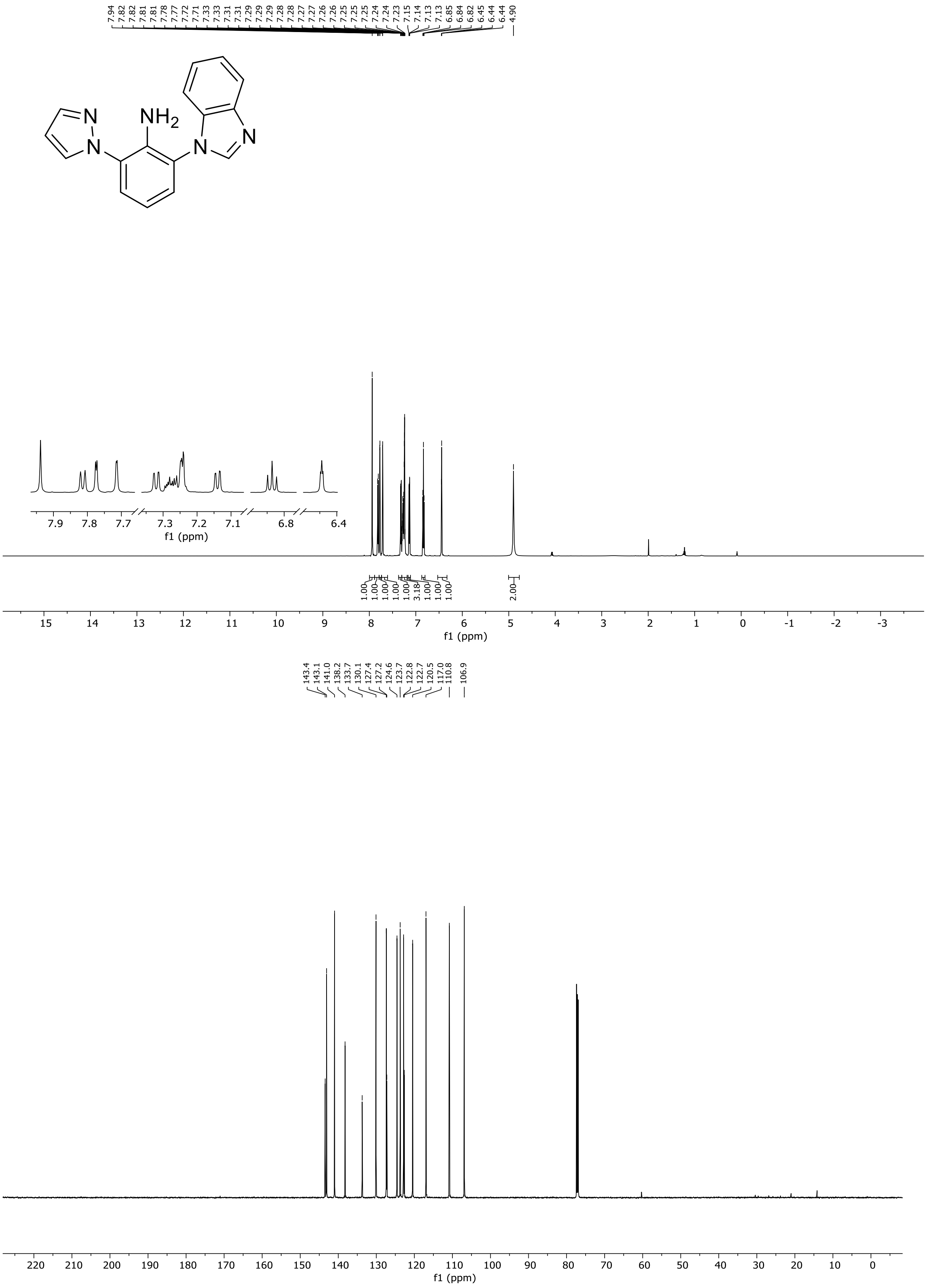

Figure S28: ^{1}H and ^{13}C NMR spectra of 2-(1*H*-benzo[*d*]imidazol-1-yl)-6-(1*H*-pyrazol-1-yl)aniline (**S4ba3**) in $CDCl_3$.

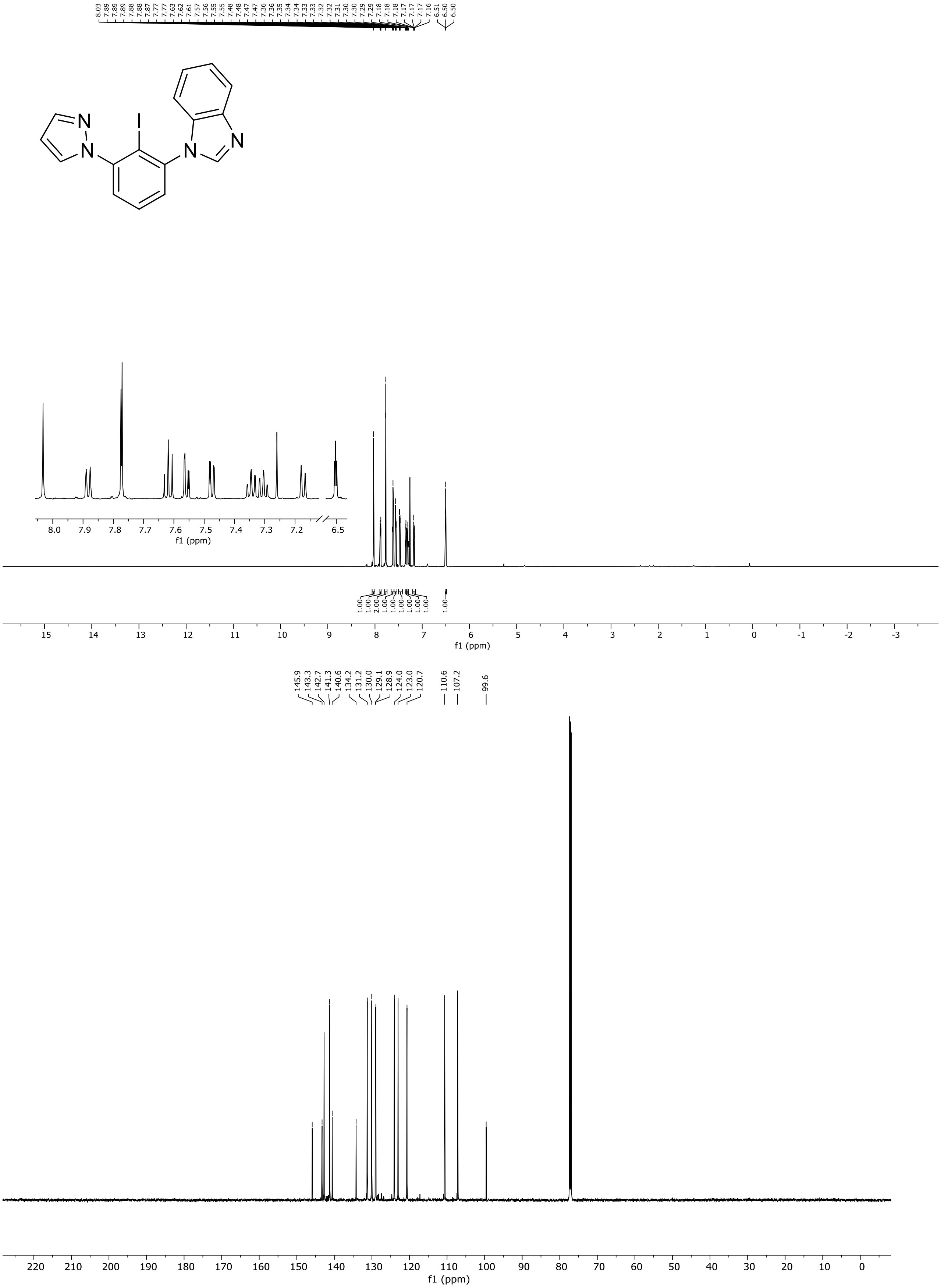

Figure S29: ^{1}H and ^{13}C NMR spectra of 1-(2-iodo-3-(1*H*-pyrazol-1-yl)phenyl)-1*H*-benzo[*d*]imidazole (**4ba**) in $CDCl_3$.

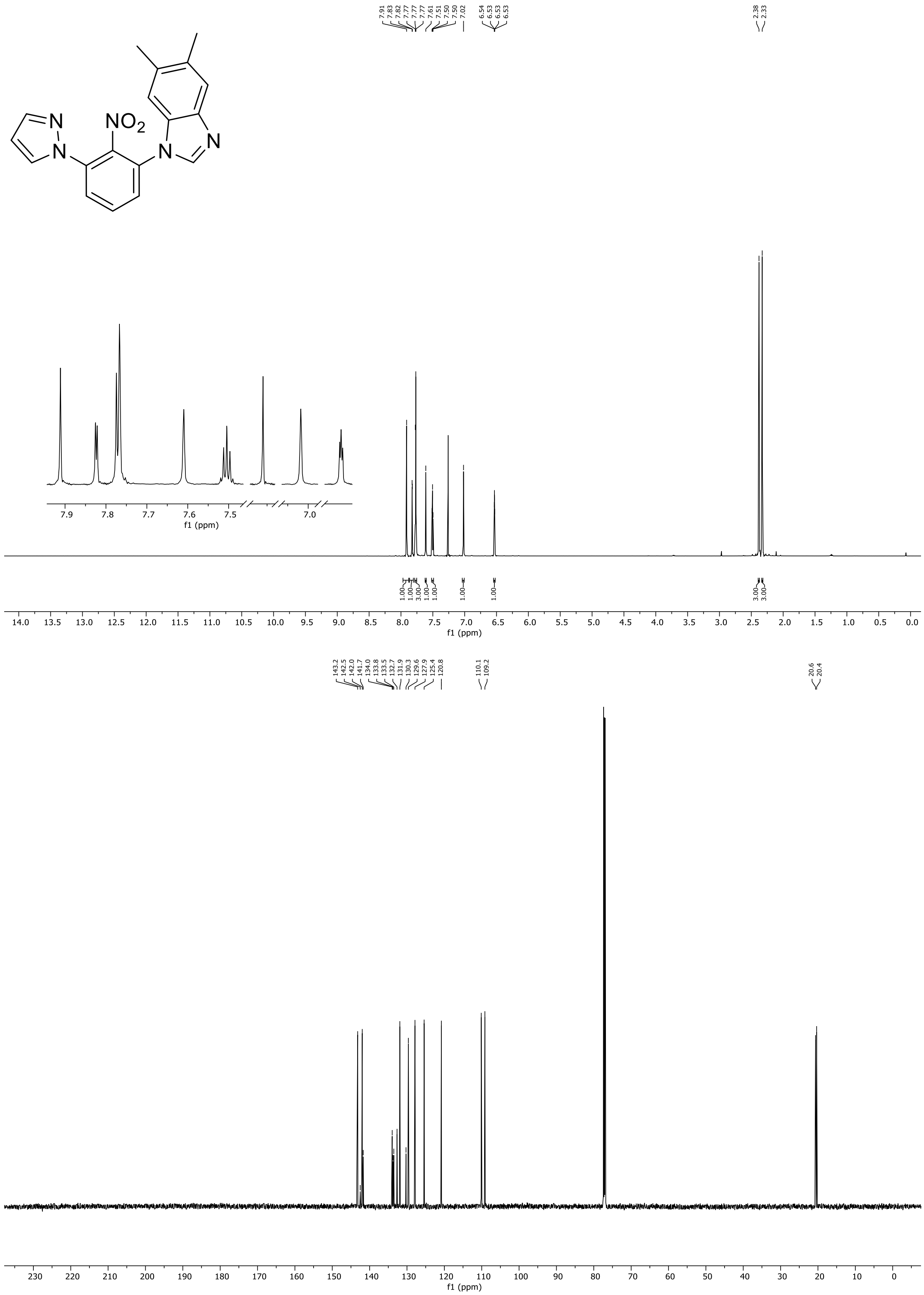

Figure S30: ^{1}H and ^{13}C NMR spectra of 5,6-dimethyl-1-(2-nitro-3-(1*H*-pyrazol-1-yl)phenyl)-1*H*-benzo[*d*]imidazole (**S4bb1**) in $CDCl_3$.

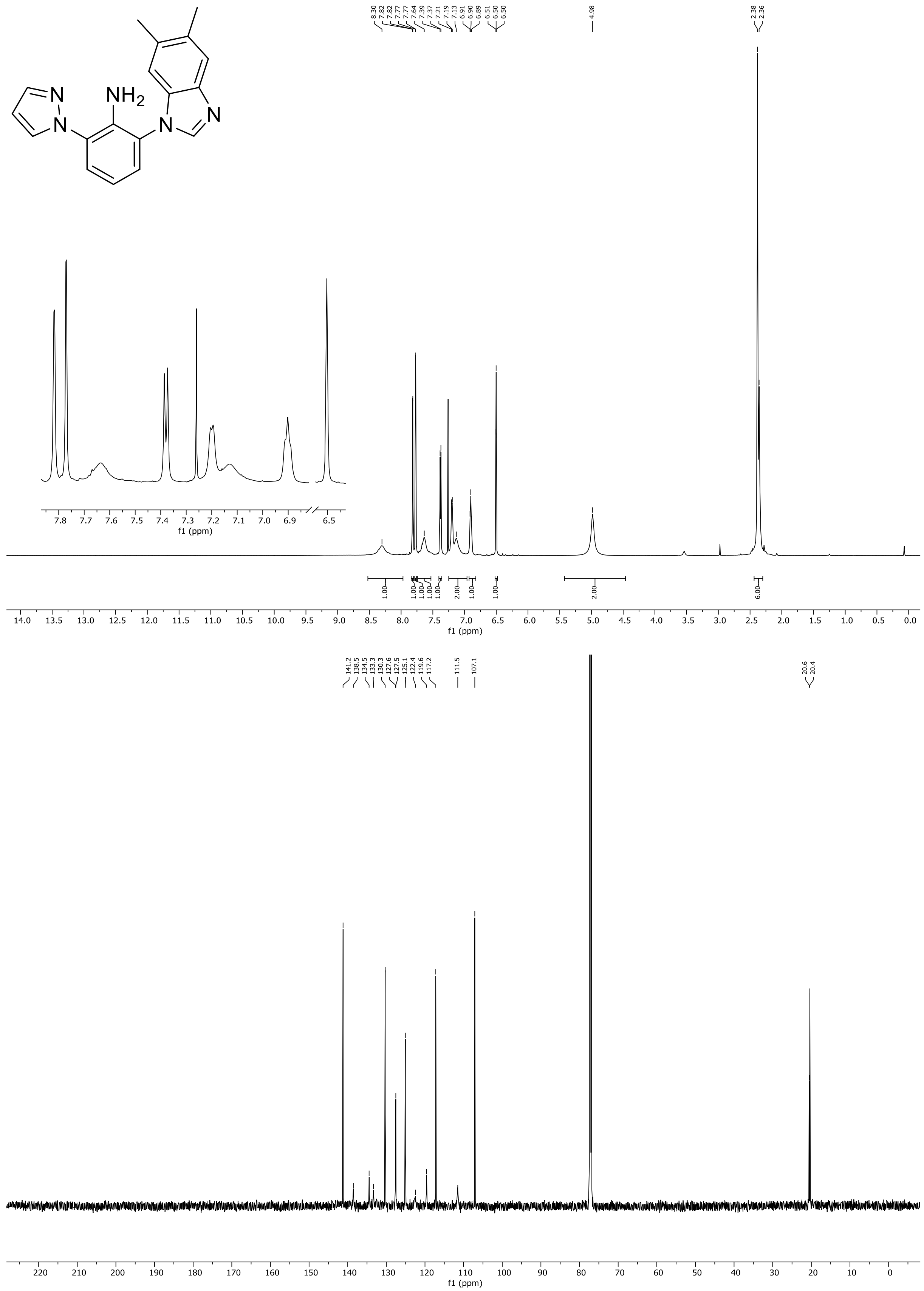

Figure S31: ^{1}H and ^{13}C NMR spectra of 2-(5,6-dimethyl-1*H*-benzo[*d*]imidazol-1-yl)-6-(1*H*-pyrazol-1-yl)aniline (**S4bb2**) in $CDCl_3$.

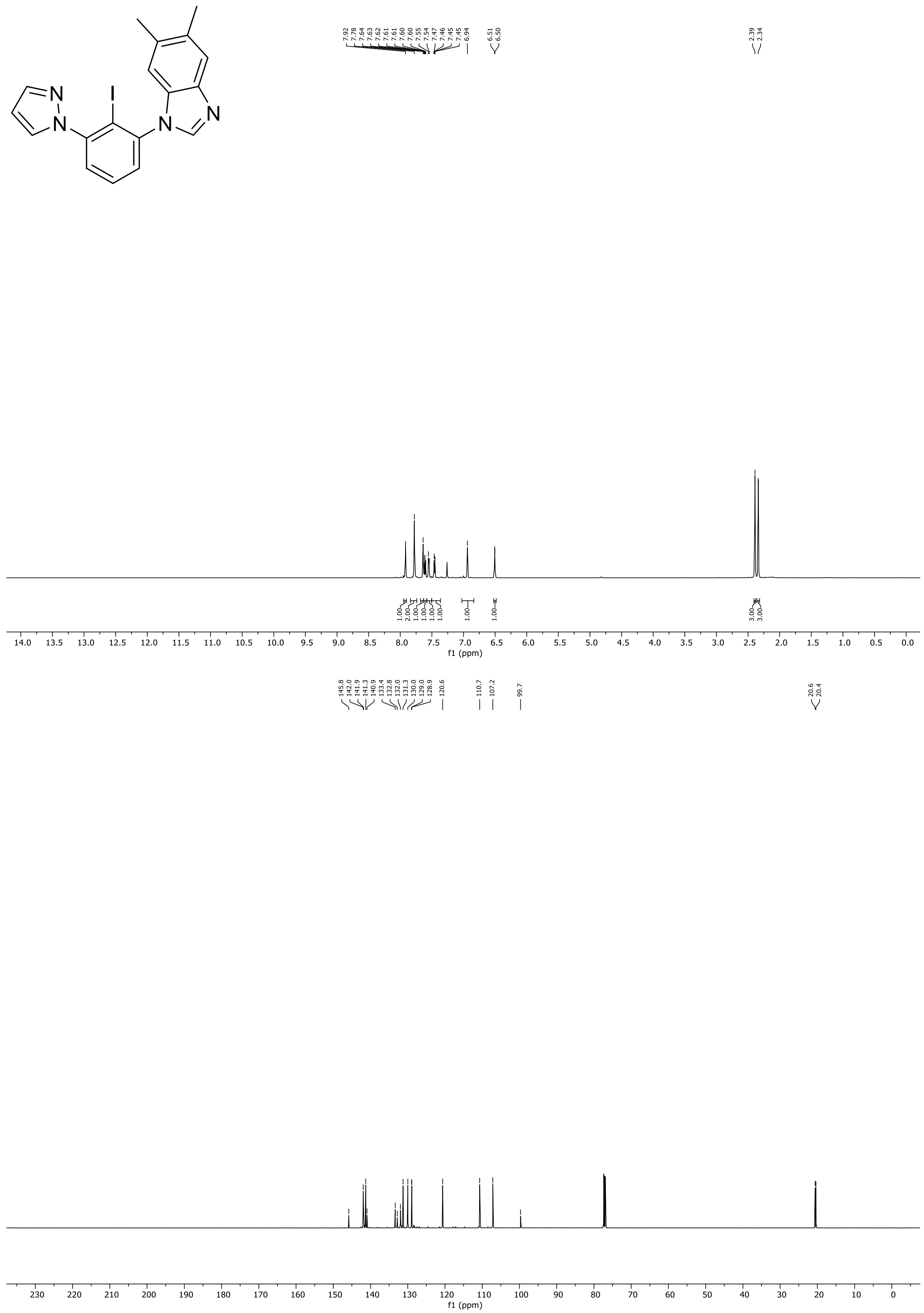

Figure S32: ^{1}H and ^{13}C NMR spectra of 5,6-dimethyl-1-(2-iodo-3-(1*H*-pyrazol-1-yl)phenyl)-1*H*-benzo[*d*]imidazole (**4bb**) in $CDCl_3$.

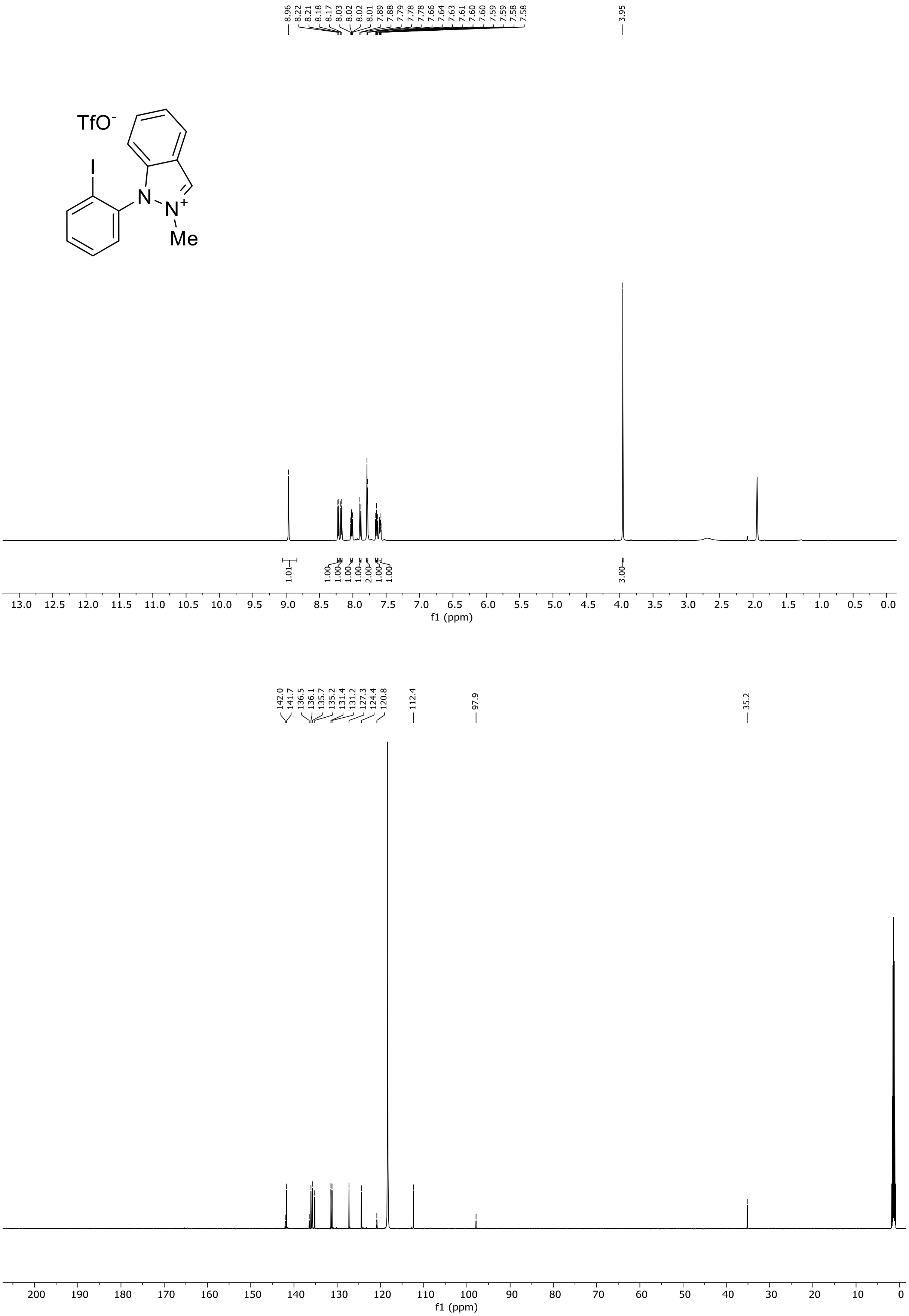

Figure S33: ^{1}H and ^{13}C NMR spectra of 1-(2-iodophenyl)-2-methyl-1*H*-indazol-2-ium triflate (**4be**) in MeCN-d_3.

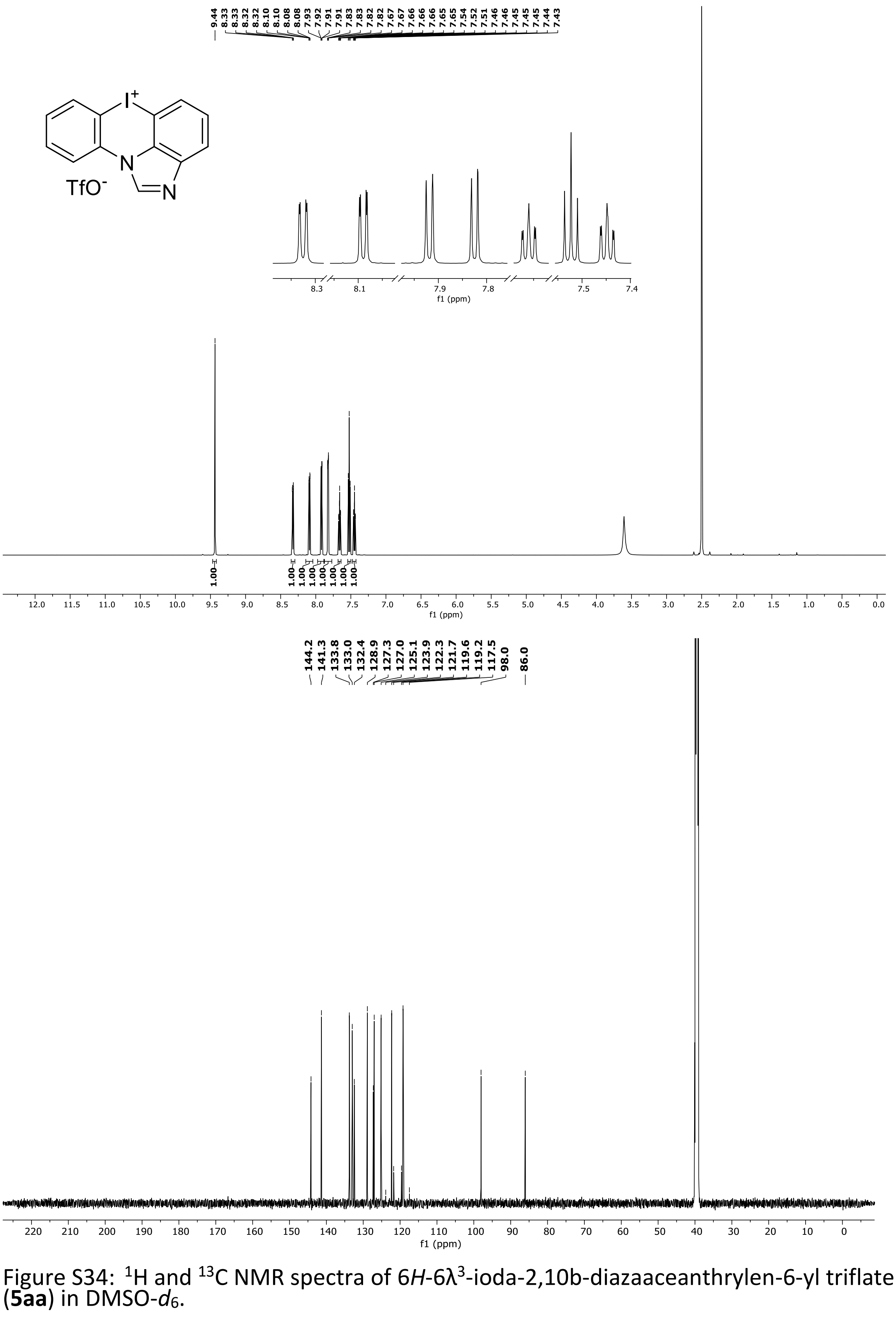

Figure S34: ^{1}H and ^{13}C NMR spectra of 6*H*-6λ^{3}-ioda-2,10b-diazaaceanthrylen-6-yl triflate (**5aa**) in DMSO-d_6.

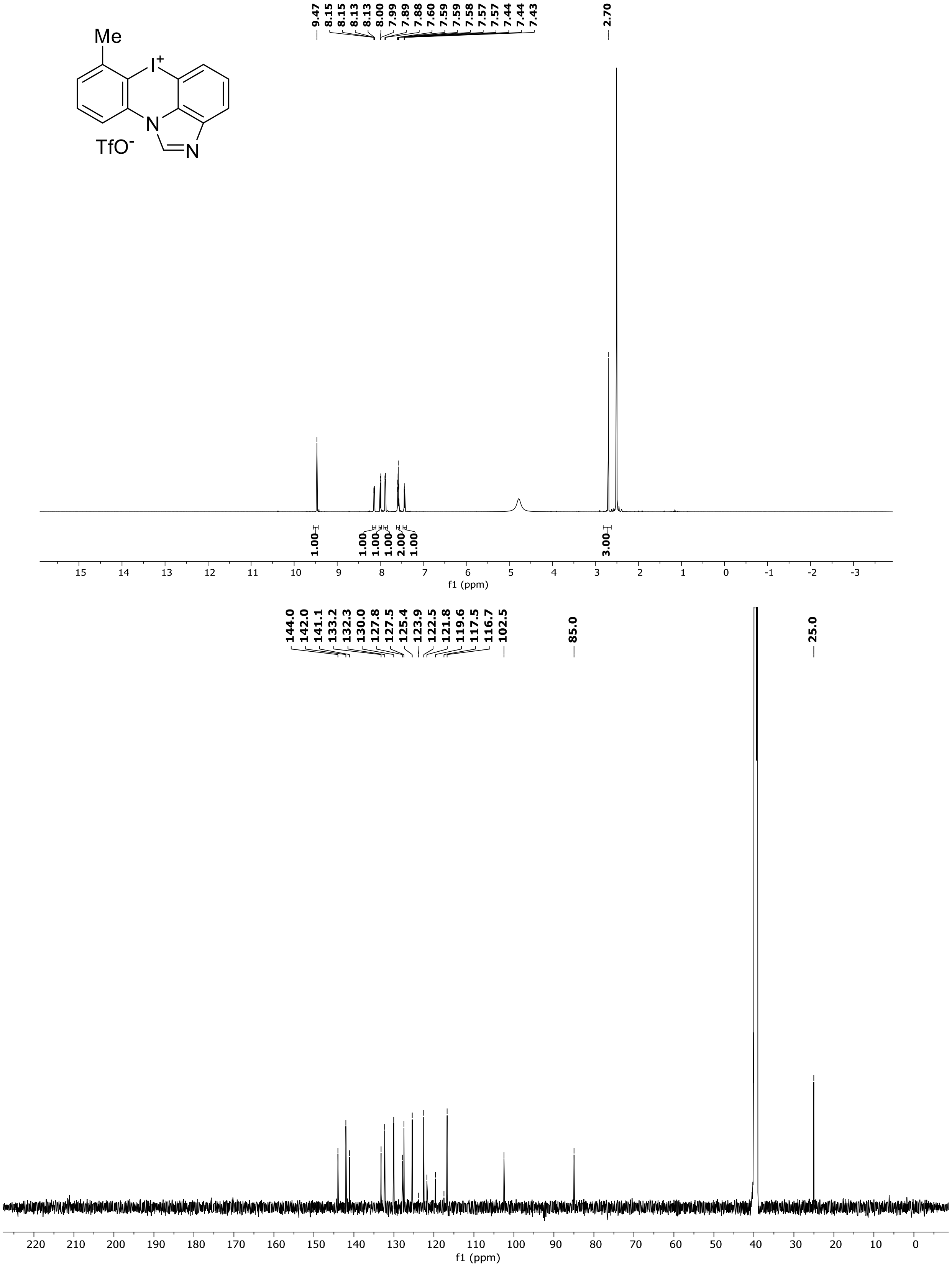

Figure S35: ^{1}H and ^{13}C NMR spectra of 7-methyl-6*H*-6λ^{3}-ioda-2,10b-diazaaceanthrylen-6-yl triflate (**5ab**) in DMSO-d_6.

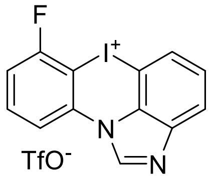
F
I+
N
N
TfO-

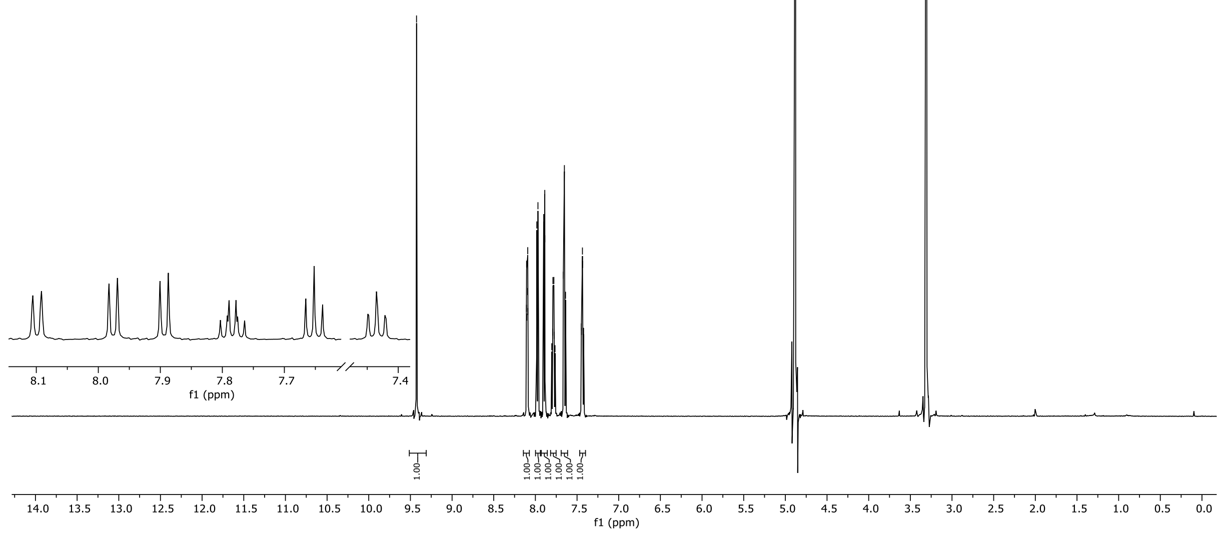
f1 (ppm)
1.00

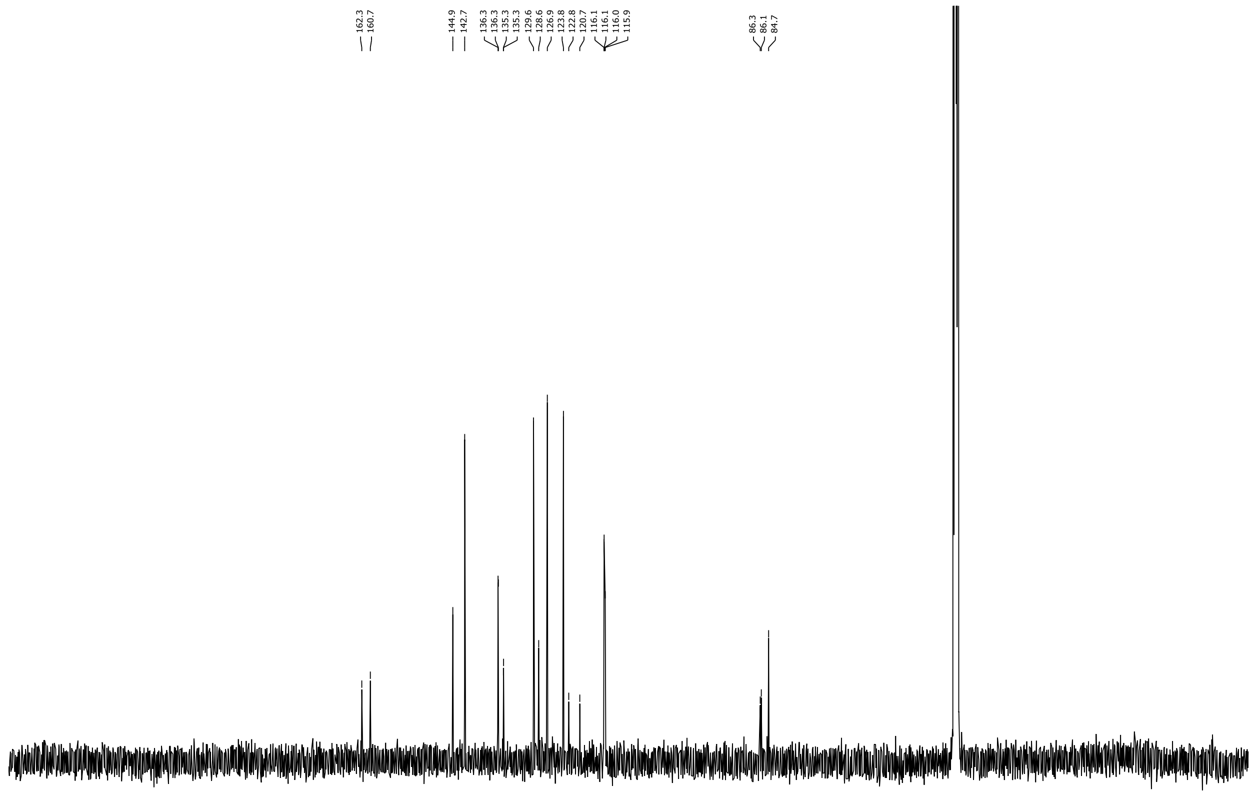
162.3
160.7
144.9
142.7
136.3
136.3
135.3
135.3
129.6
128.6
126.9
123.8
122.8
120.7
116.1
116.0
115.9
86.3
86.1
84.7
f1 (ppm)

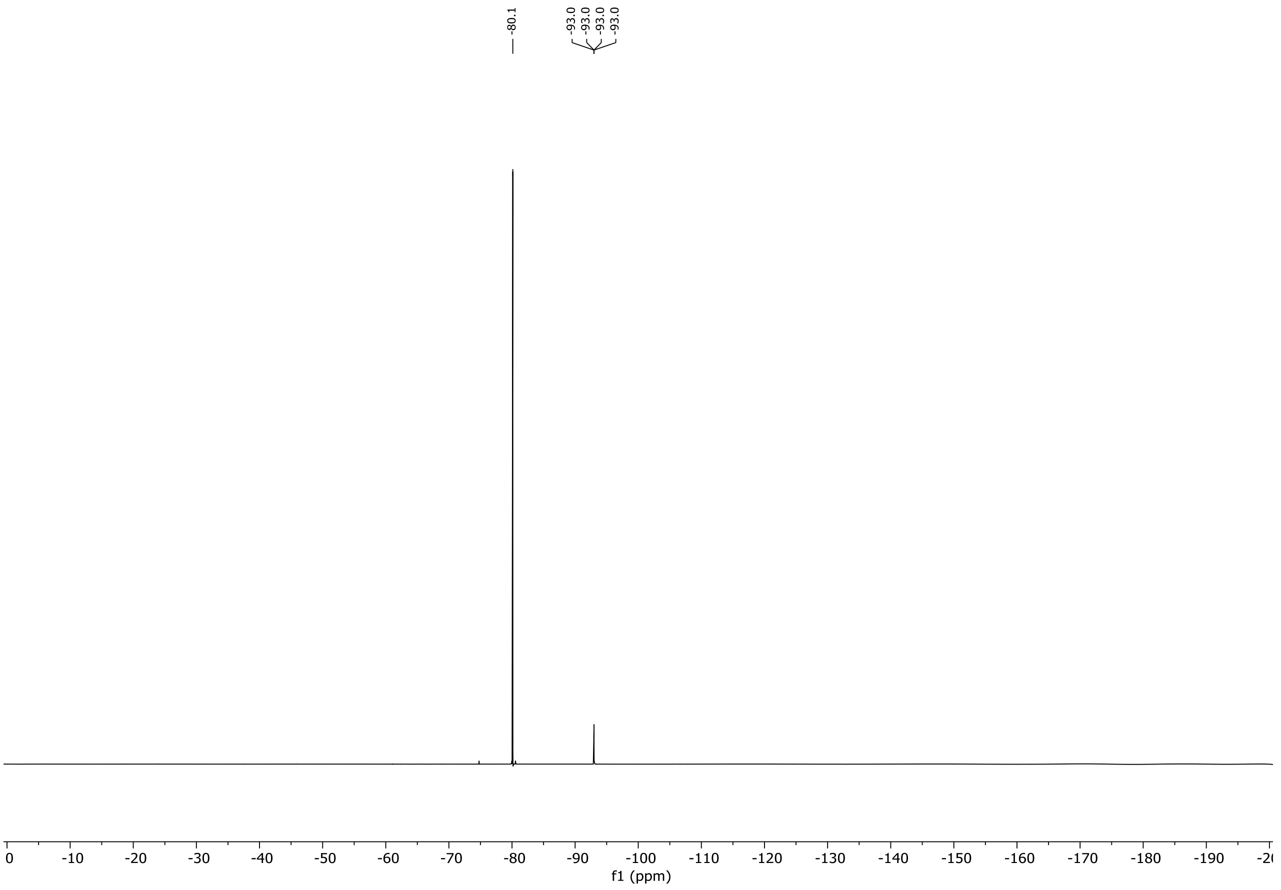

Figure S36: ^{1}H, ^{13}C and ^{19}F NMR spectra of 7-fluoro-6*H*-6λ^{3}-ioda-2,10b-diazaaceanthrylen-6-yl triflate (**5ac**) in CD_3OD.

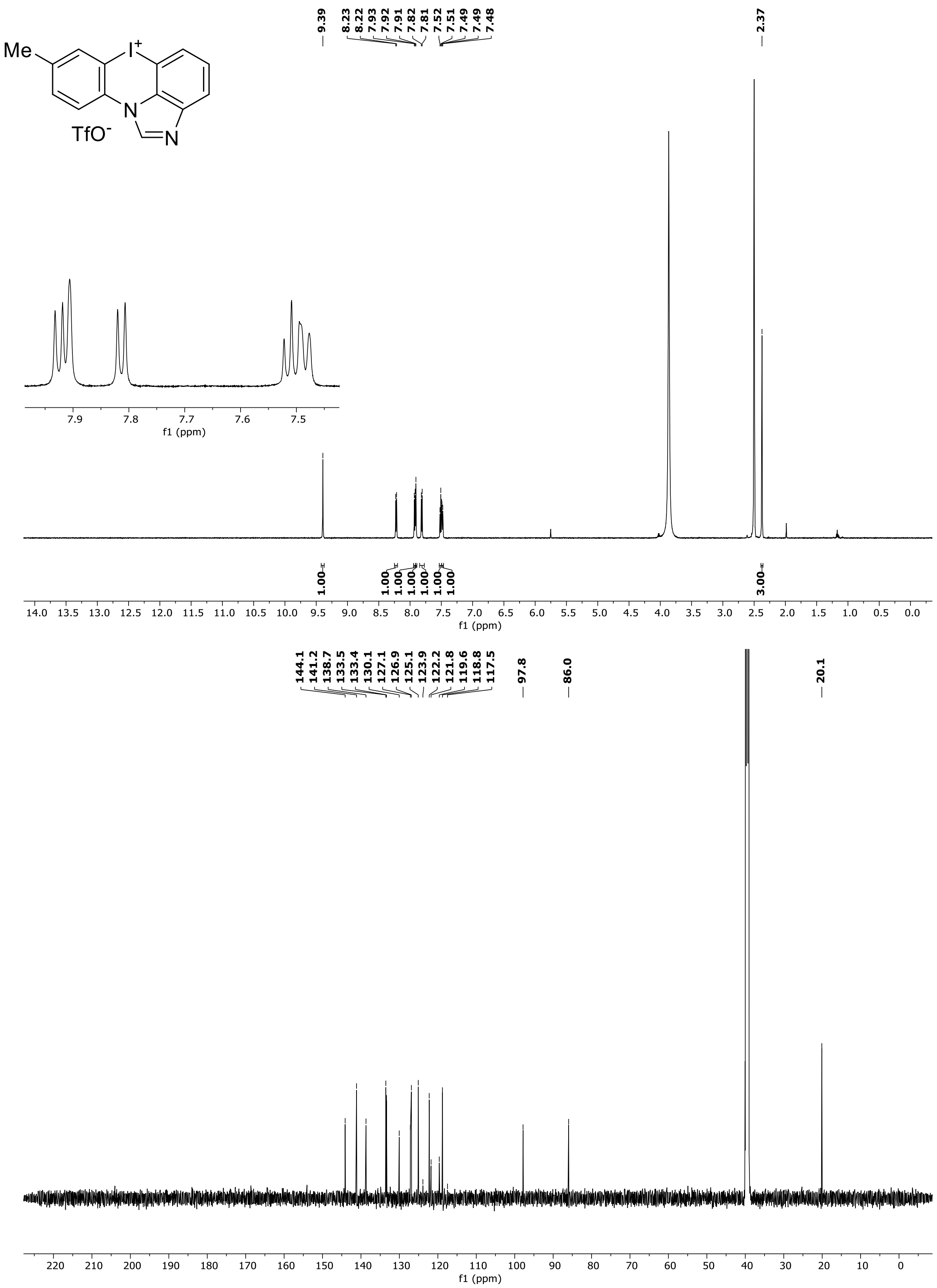

Figure S37: ^{1}H and ^{13}C NMR spectra of 8-methyl-6*H*-6λ^{3}-ioda-2,10b-diazaaceanthrylen-6-yl triflate (**5af**) in DMSO-d_6.

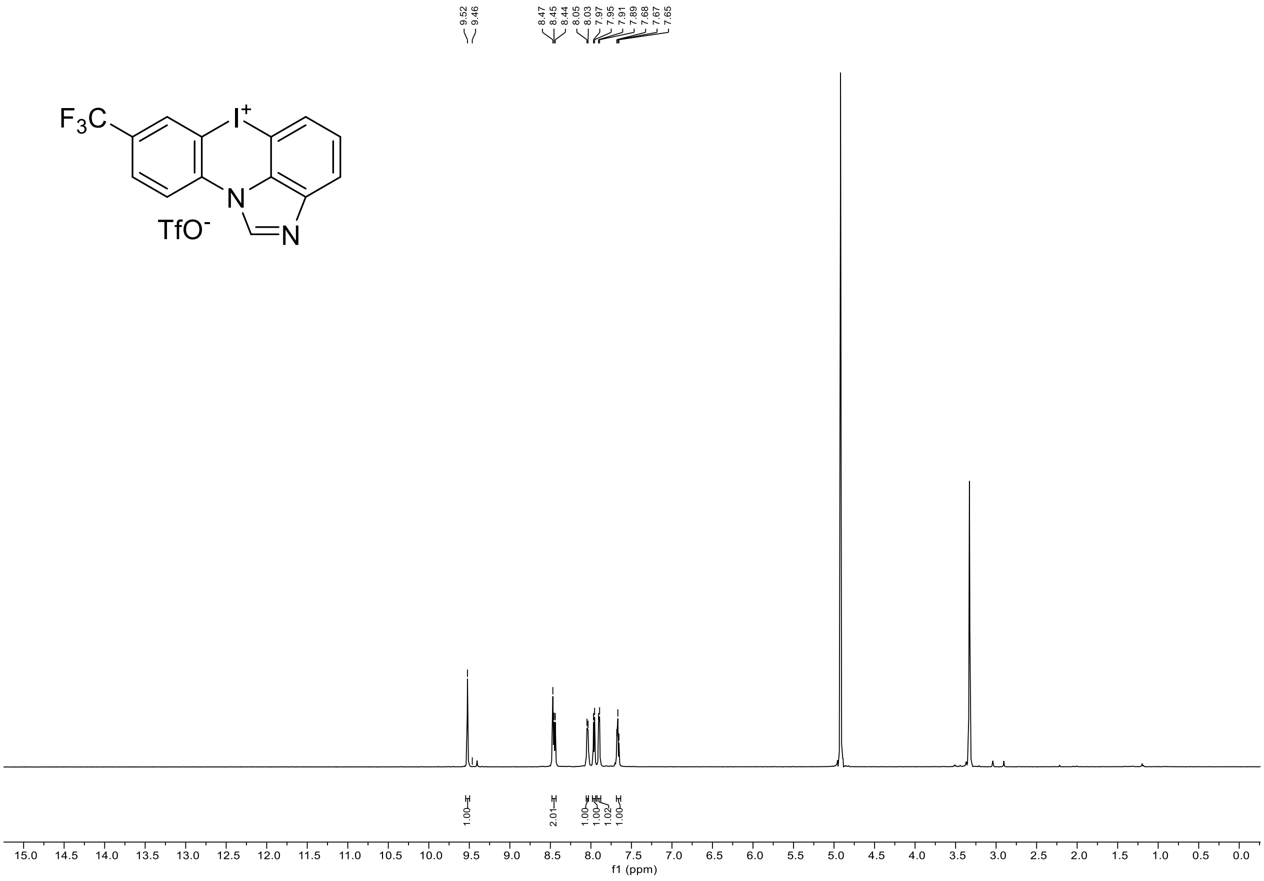
F3C
I+
N
TfO-
N
9.52
9.46
8.47
8.45
8.44
8.05
8.03
7.97
7.95
7.91
7.89
7.68
7.67
7.65
1.00
2.01
1.00
1.00
1.02
1.00
f1 (ppm)

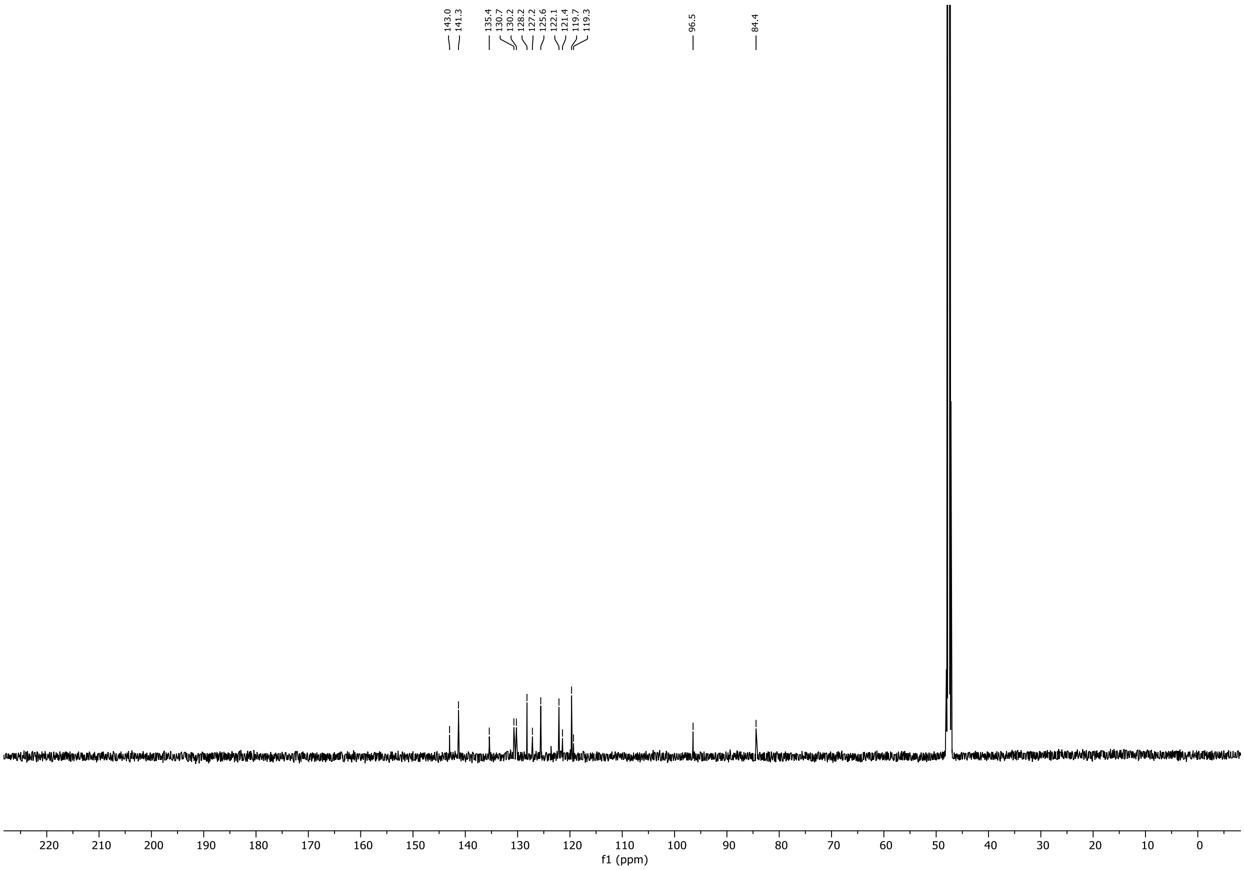
143.0
141.3
135.4
130.7
130.2
128.2
127.2
125.6
122.1
121.4
119.7
119.3
96.5
84.4
f1 (ppm)

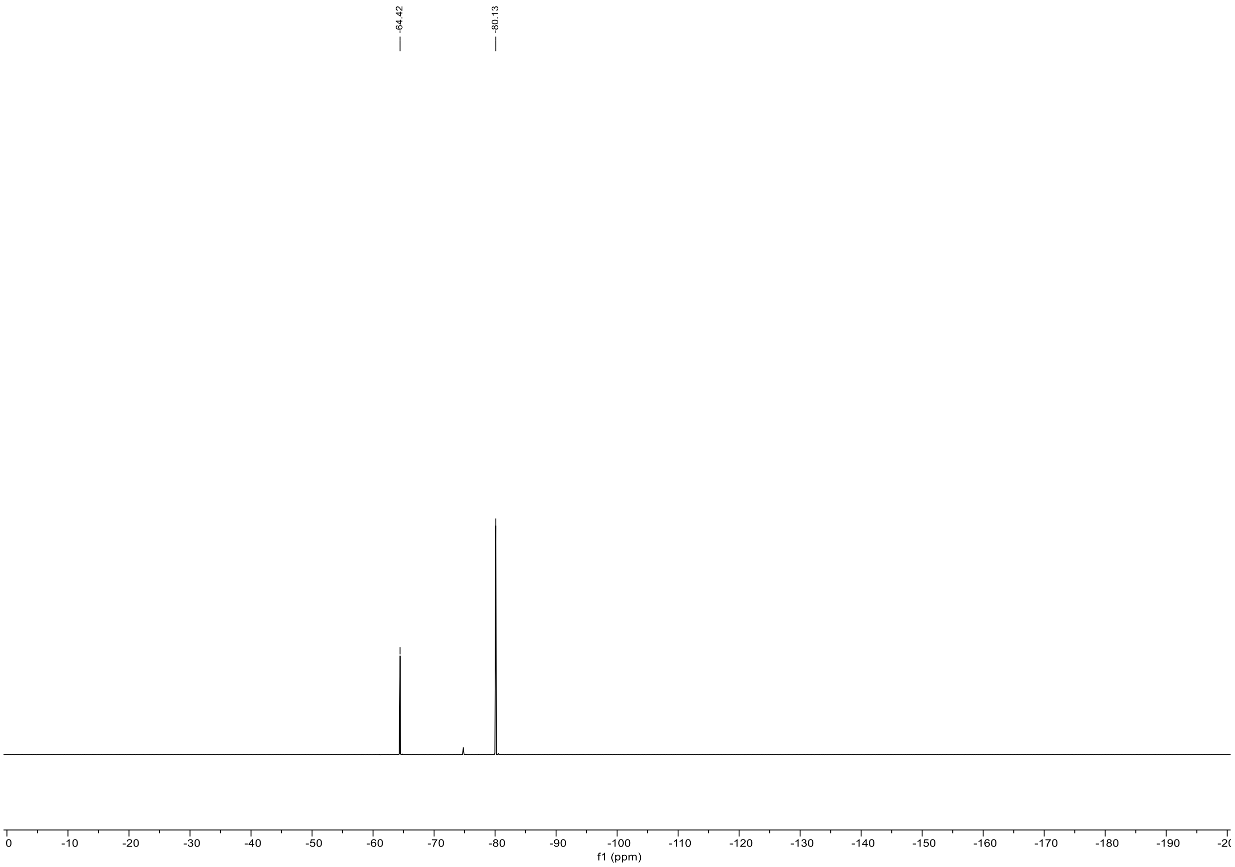

Figure S38: ^{1}H, ^{13}C and ^{19}F NMR spectra of 8-(trifluoromethyl)-6*H*-6λ^{3}-ioda-2,10b-diazaaceanthrylen-6-yl triflate (**5ag**) in CD_3OD.

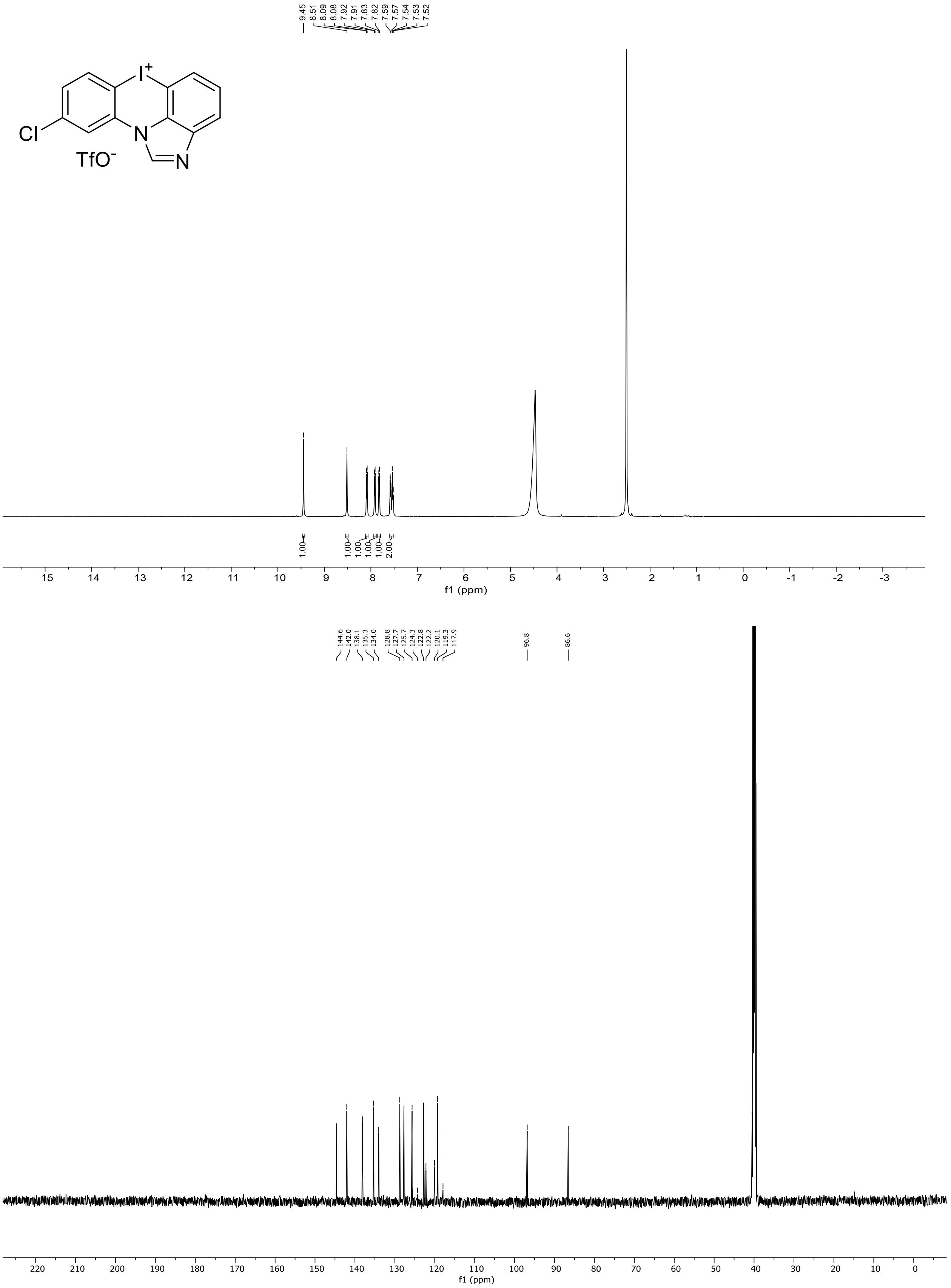

Figure S39: ^{1}H and ^{13}C NMR spectra of 9-chloro-6*H*-6λ^{3}-ioda-2,10b-diazaaceanthrylen-6-yl triflate (**5ah**) in DMSO-d_6.

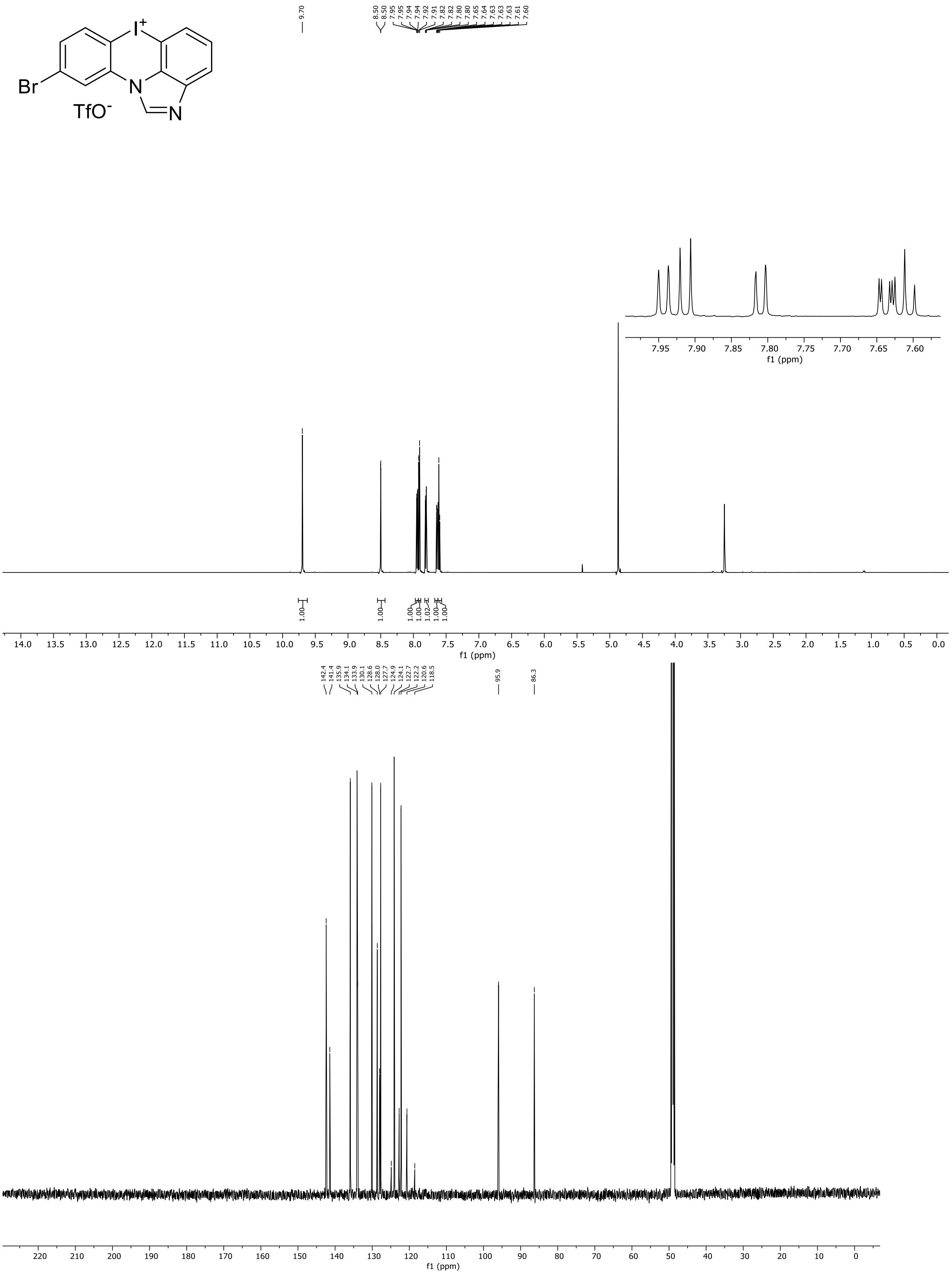

Figure S40: ^{1}H and ^{13}C NMR spectra of 9-bromo-6*H*-6λ^{3}-ioda-2,10b-diazaaceanthrylen-6-yl triflate (**5ai**) in DMSO-d_6.

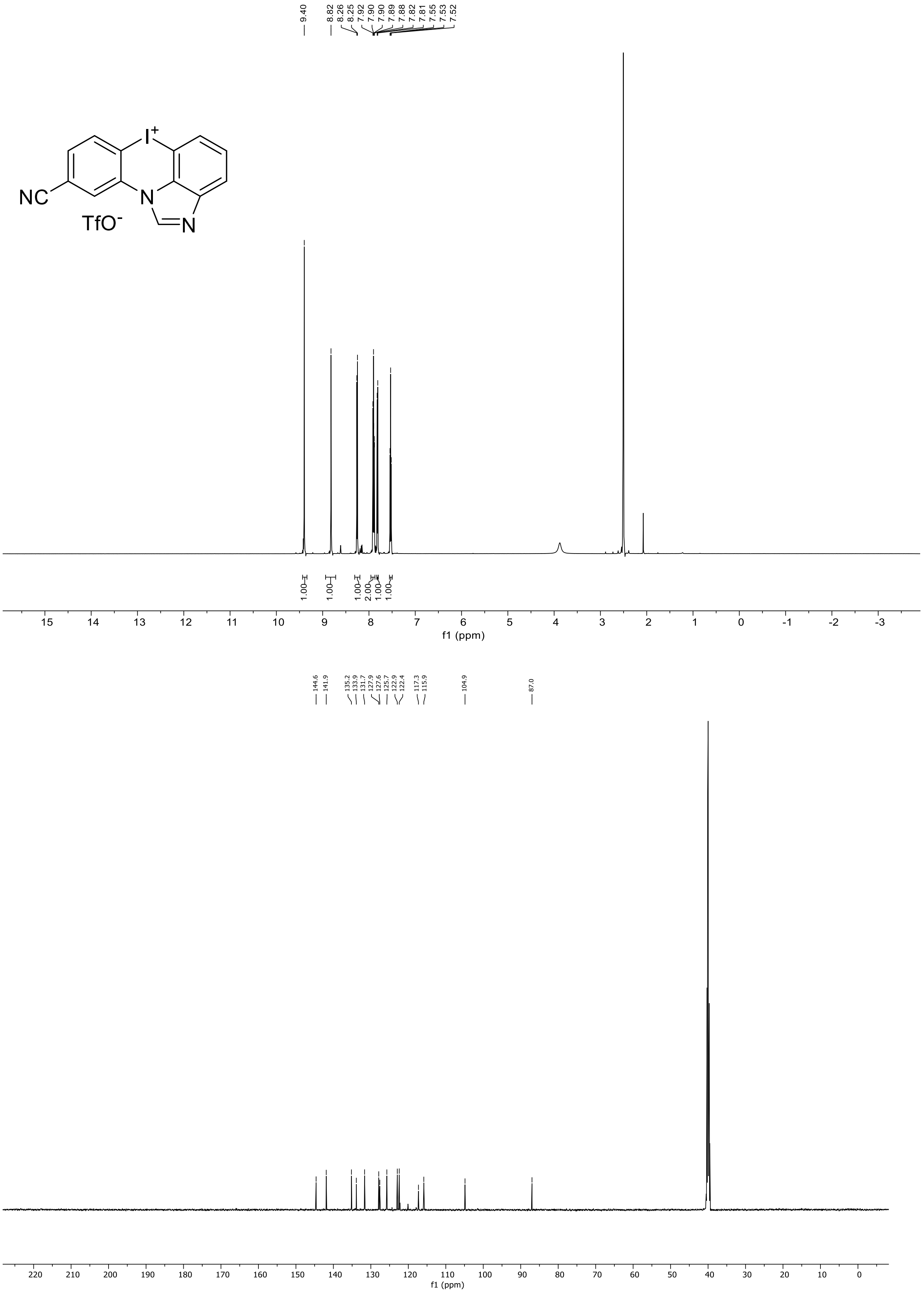

Figure S41: ^{1}H and ^{13}C NMR spectra of 9-cyano-6*H*-6λ^{3}-ioda-2,10b-diazaaceanthrylen-6-yl triflate (**5aj**) in DMSO-d_6.

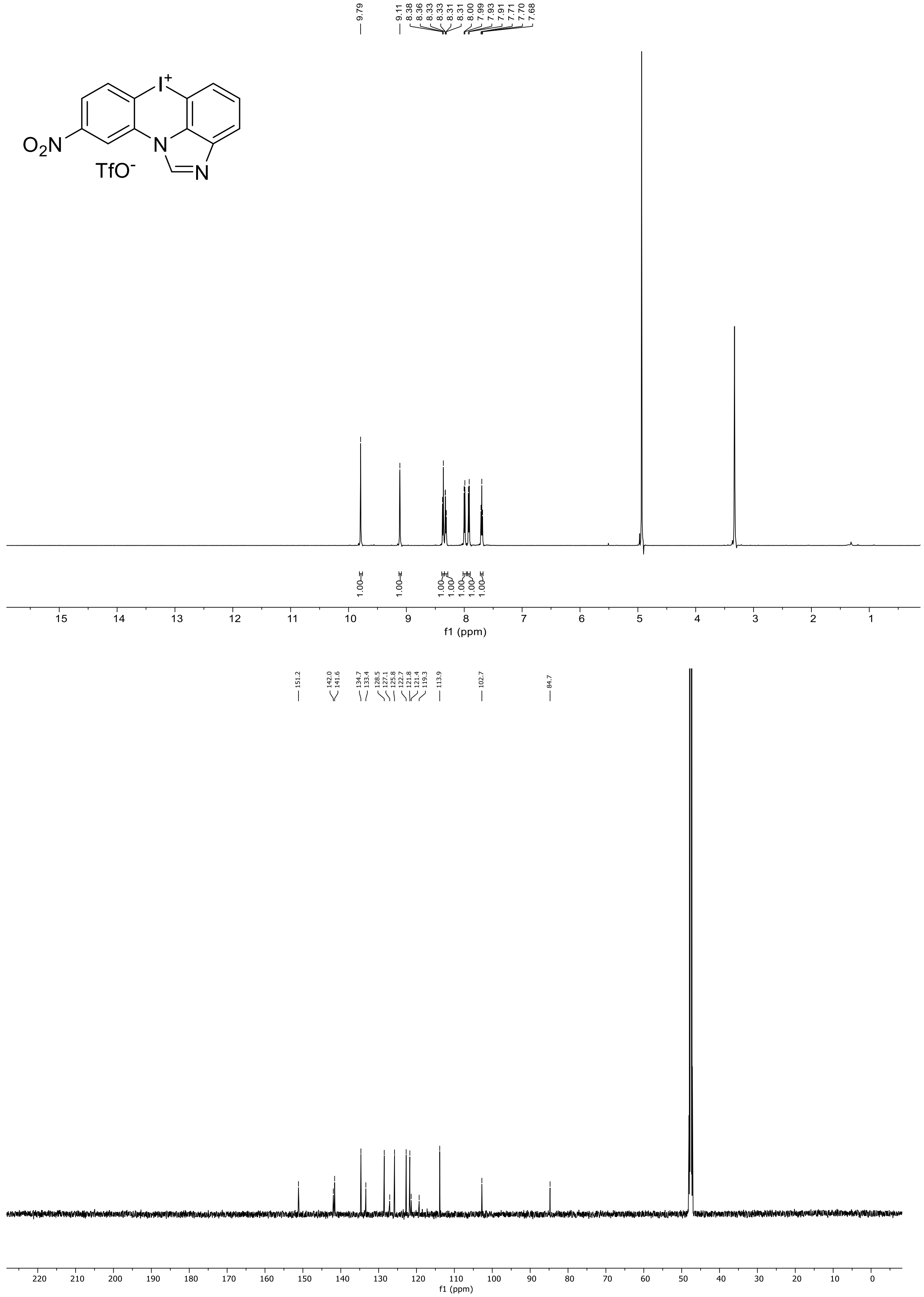

Figure S42: ^{1}H and ^{13}C NMR spectra of 9-nitro-6*H*-6λ^{3}-ioda-2,10b-diazaaceanthrylen-6-yl triflate (**5ak**) in DMSO-*d*$_{6}$.

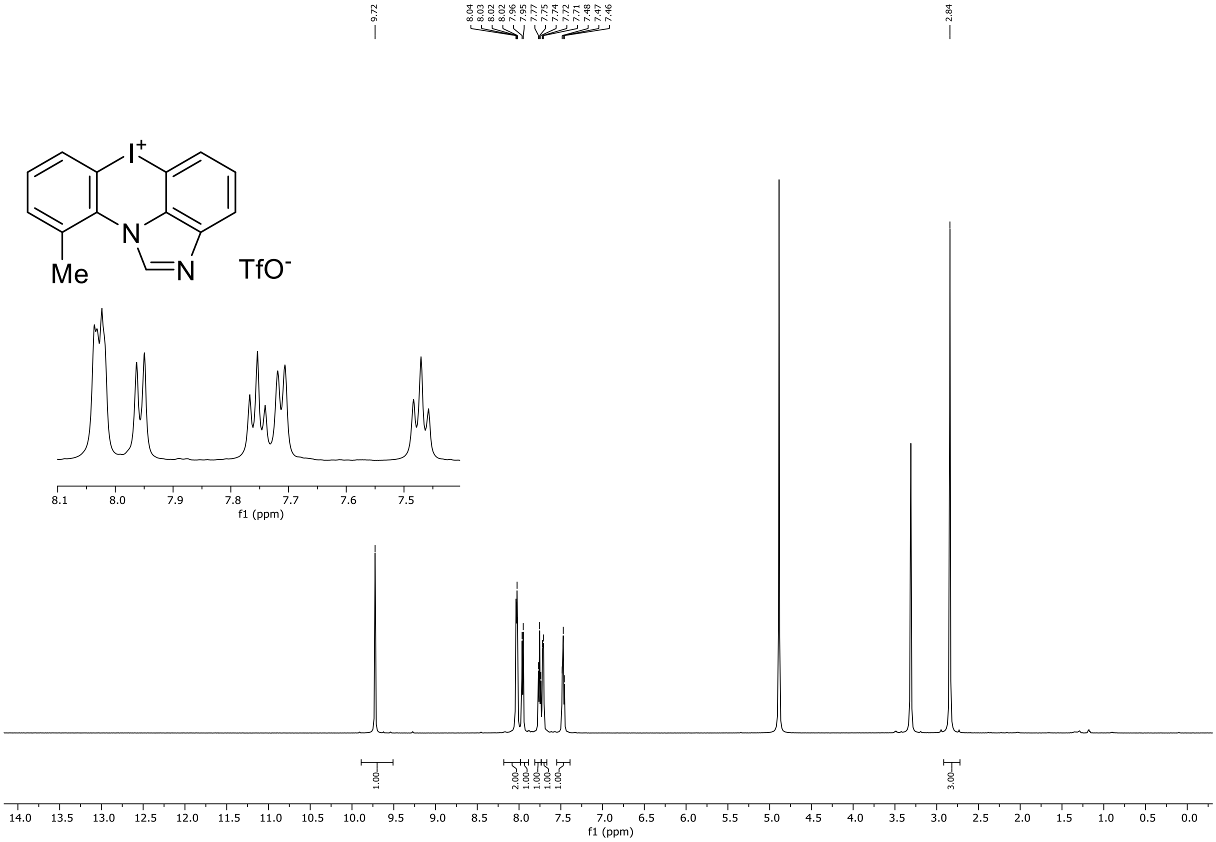

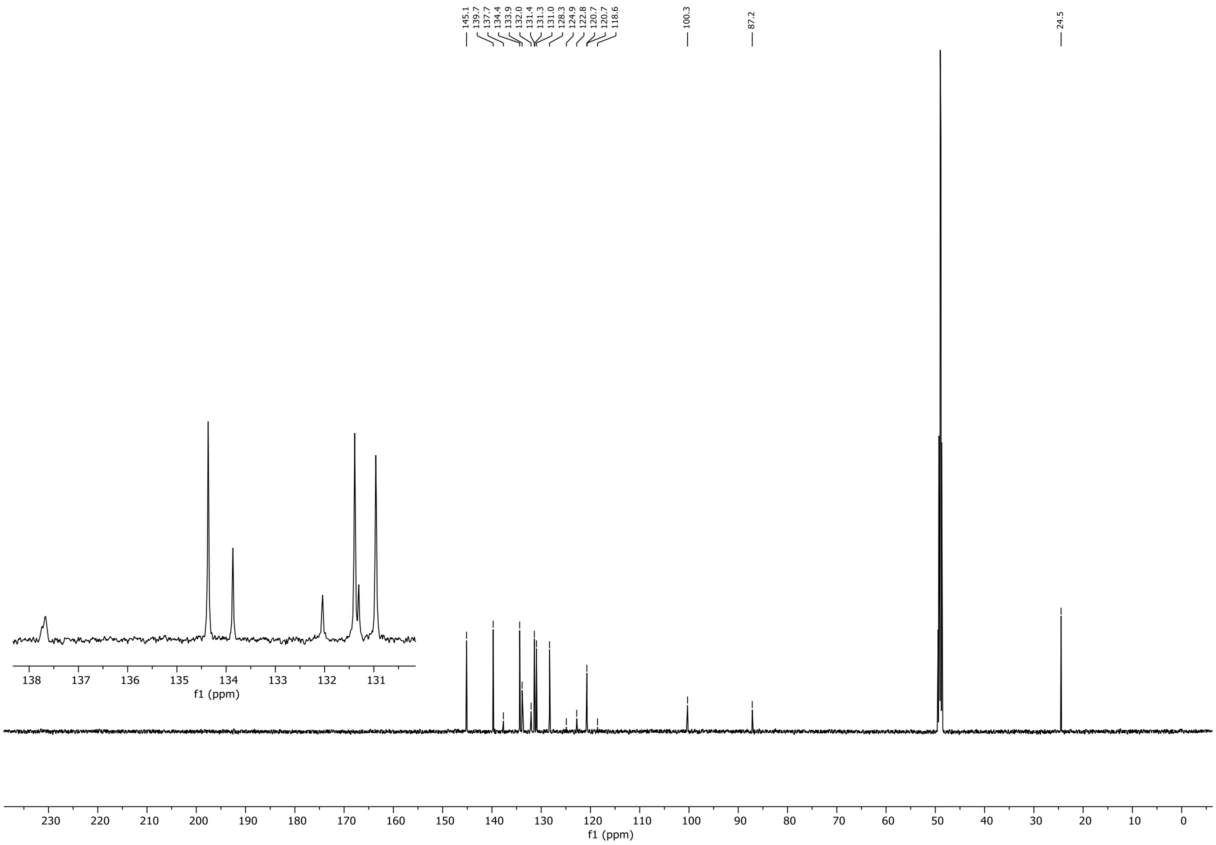

Figure S43: ^{1}H and ^{13}C NMR spectra of 10-methyl-6*H*-6λ^{3}-ioda-2,10b-diazaaceanthrylen-6-yl triflate (**5al**) in DMSO-d_6.

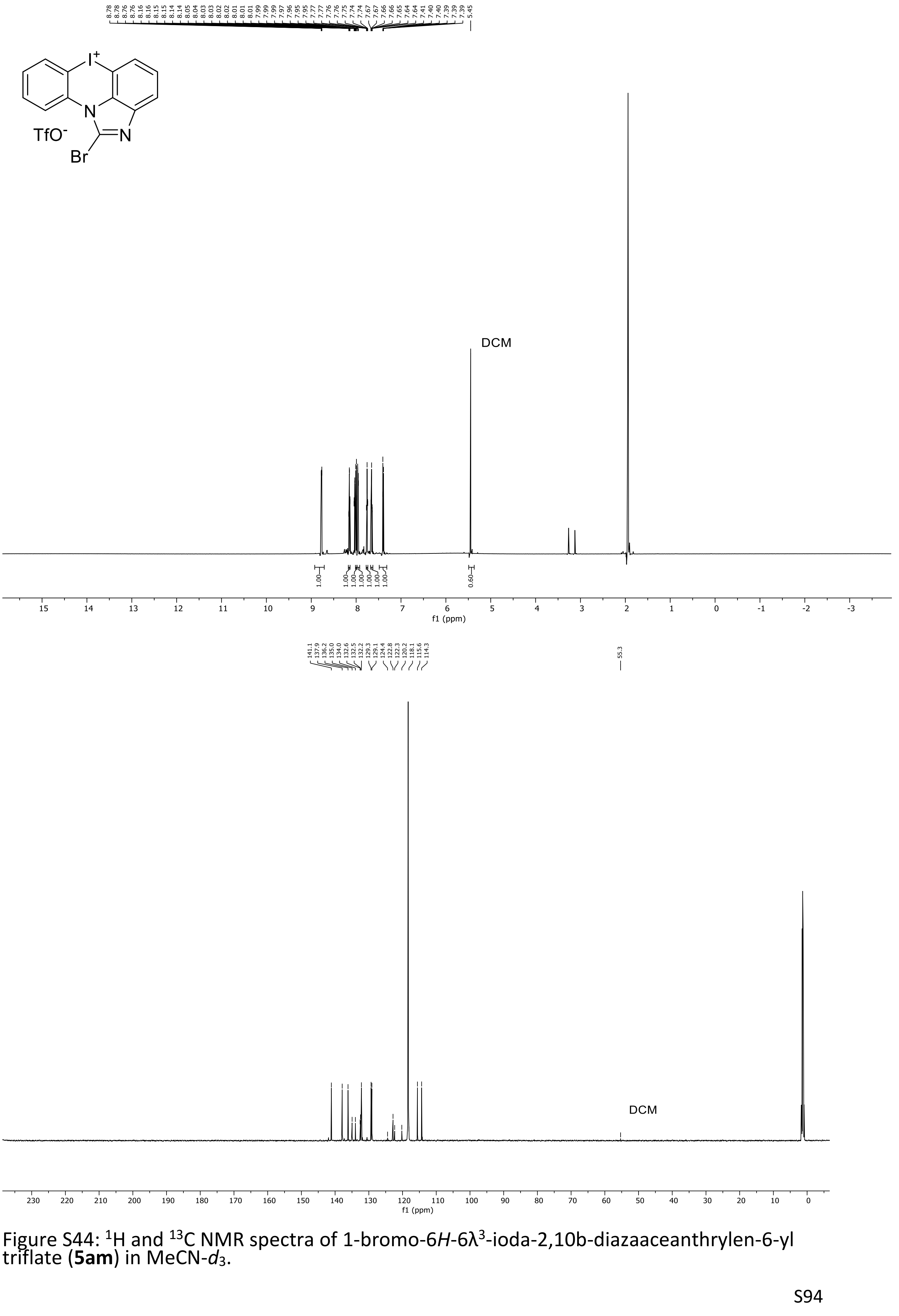

Figure S44: ^{1}H and ^{13}C NMR spectra of 1-bromo-6*H*-6λ^{3}-ioda-2,10b-diazaaceanthrylen-6-yl triflate (**5am**) in MeCN-d_3.

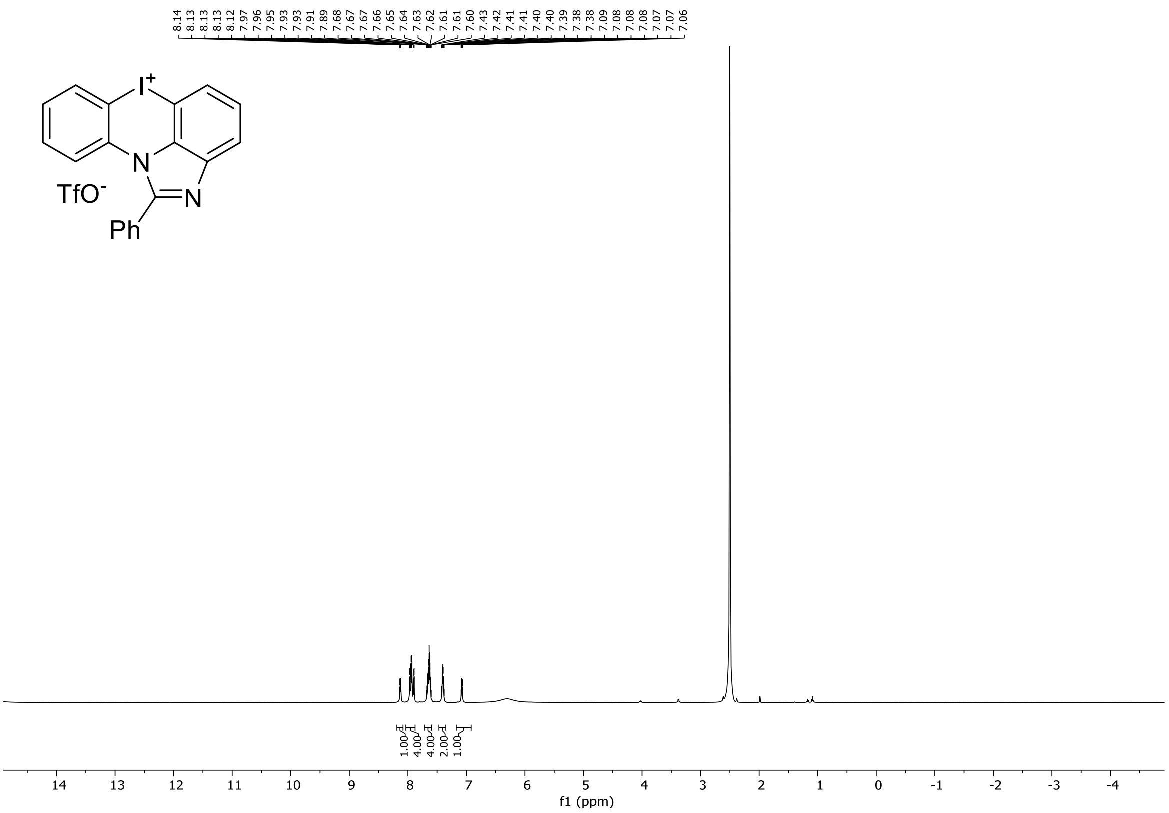

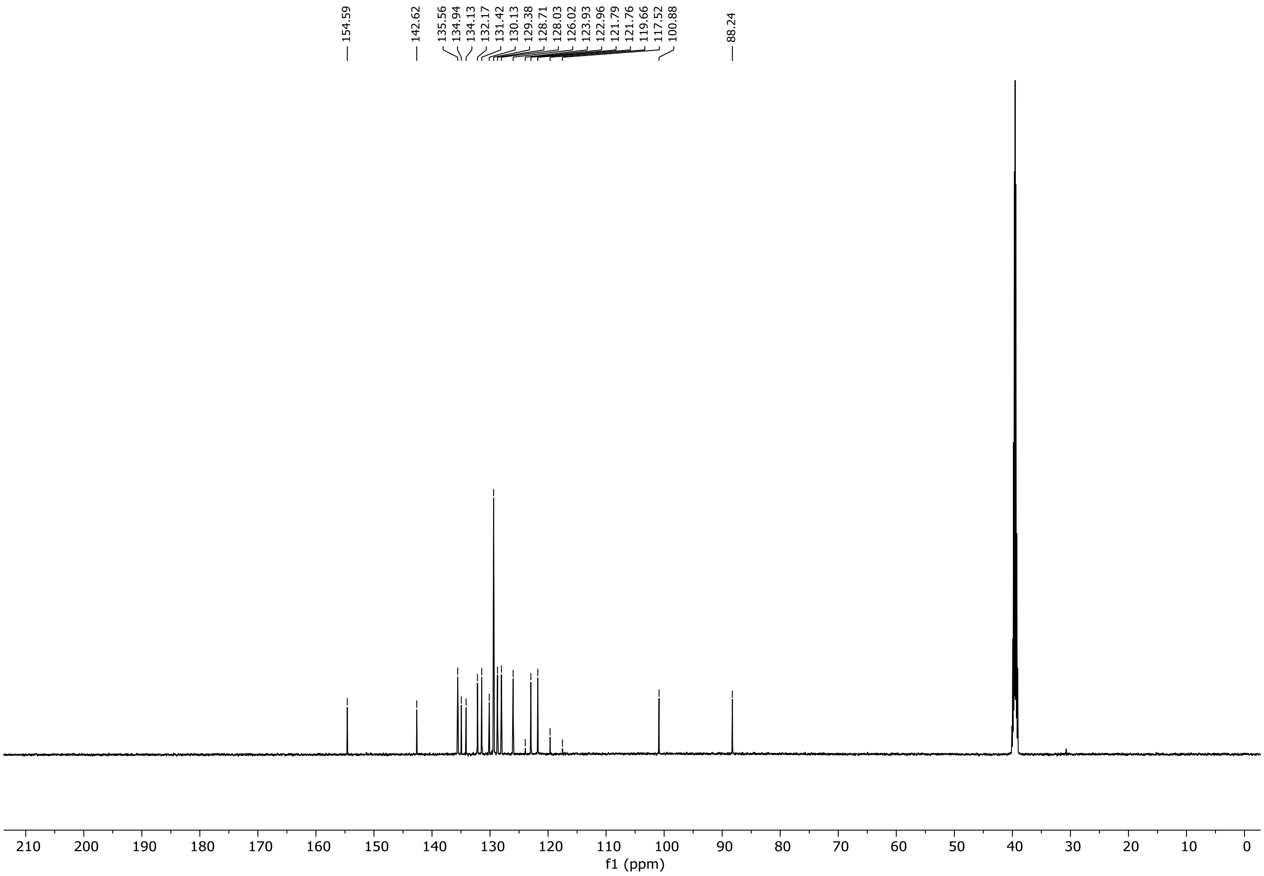

Figure S45: ^{1}H and ^{13}C NMR spectra of 1-phenyl-6*H*-6λ^{3}-ioda-2,10b-diazaaceanthrylen-6-yl triflate (**5an**) in DMSO-d_6.

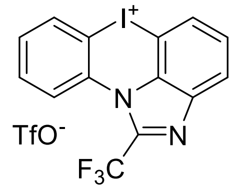
I+
N
N
TfO-
F3C

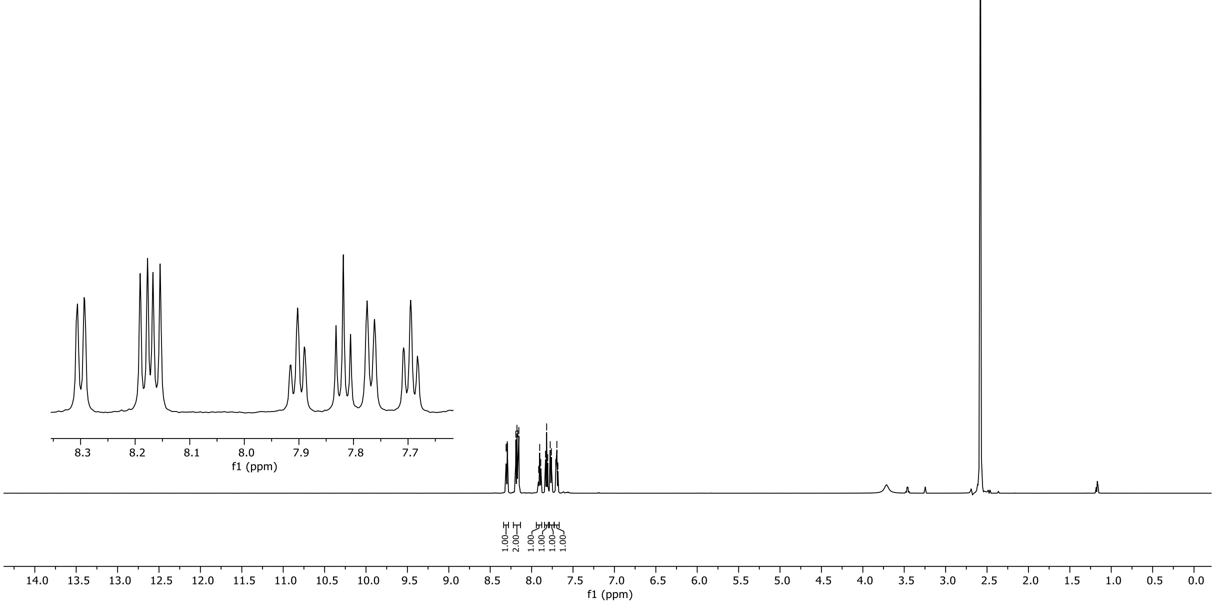
8.31
8.31
8.29
8.29
8.19
8.18
8.17
8.15
7.91
7.90
7.89
7.83
7.82
7.81
7.78
7.76
7.71
7.70
7.68
f1 (ppm)
f1 (ppm)

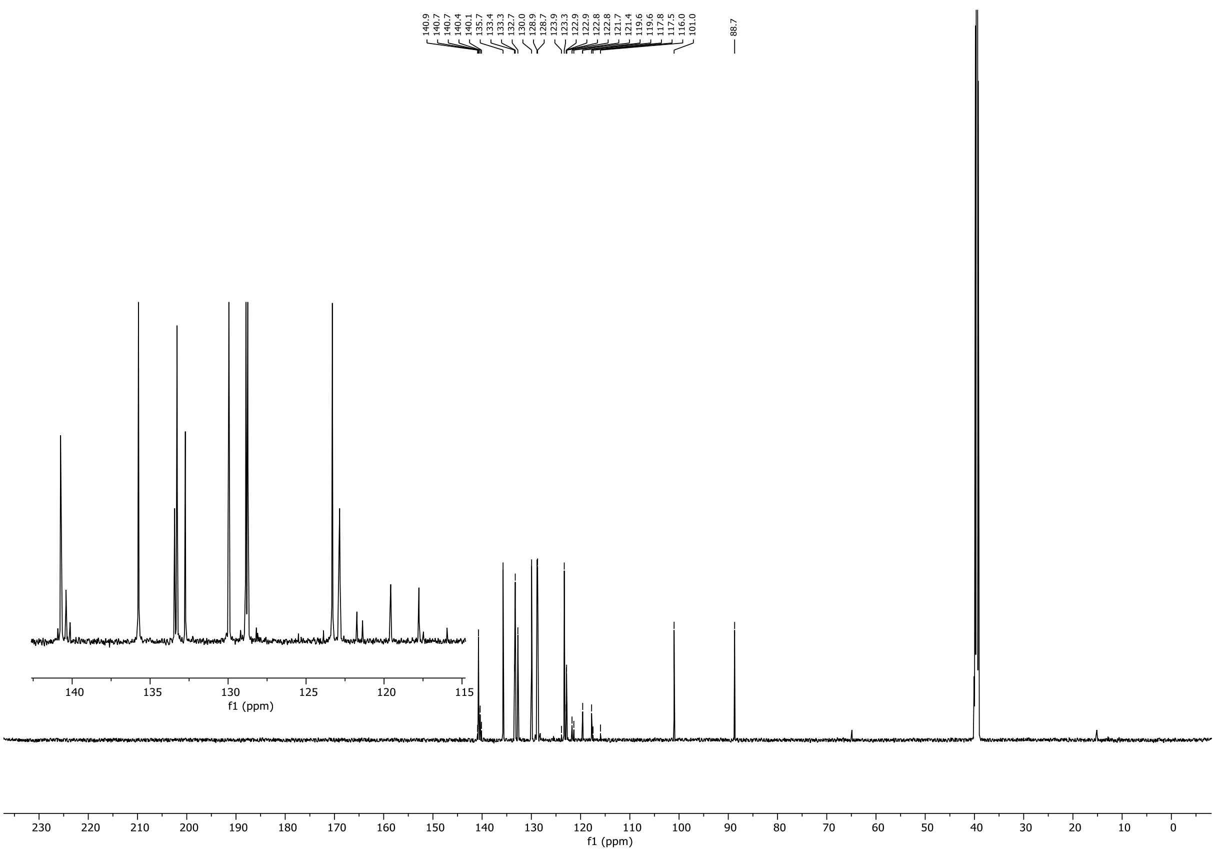
140.9
140.7
140.7
140.4
140.1
135.7
133.4
133.3
132.7
130.0
128.9
128.7
123.9
123.3
122.9
122.9
122.8
122.8
121.7
121.4
119.6
119.6
117.8
117.5
116.0
101.0
88.7
f1 (ppm)
f1 (ppm)

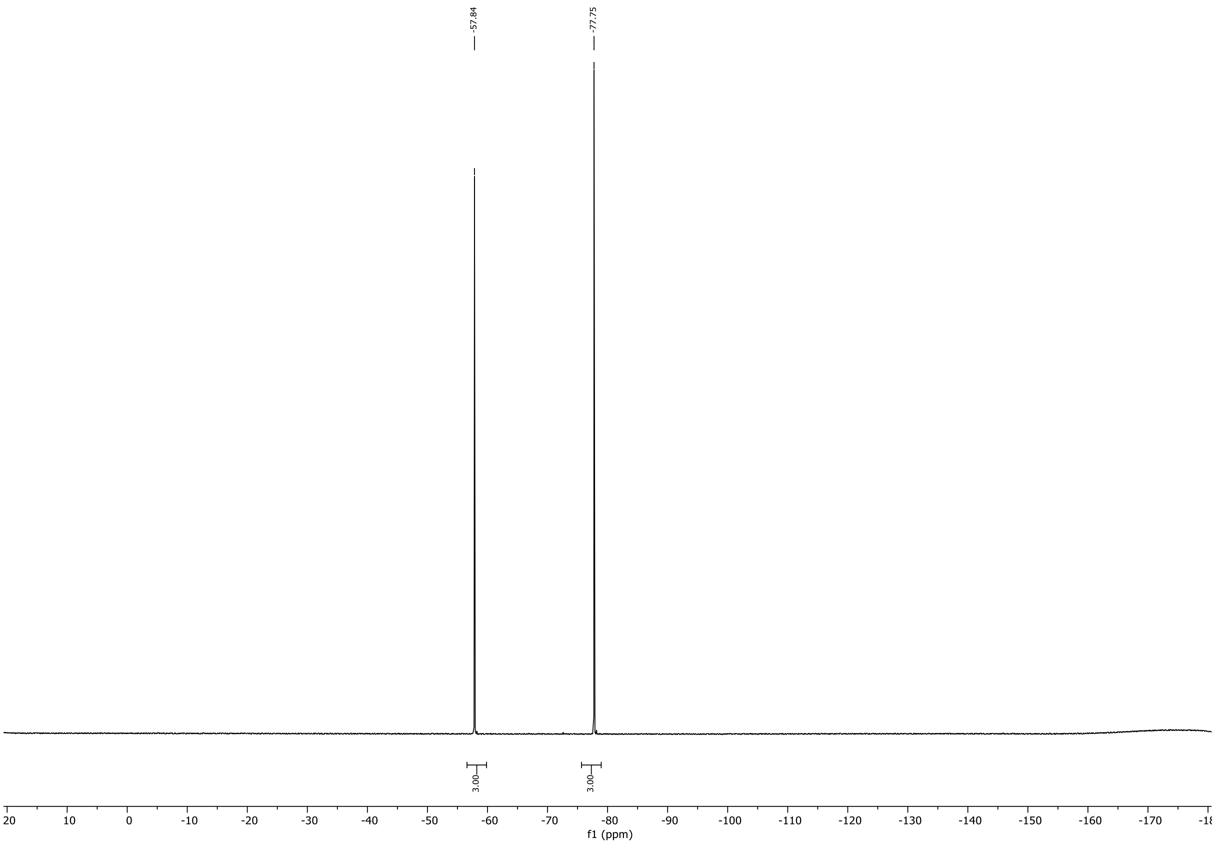

Figure S46: ^{1}H and ^{13}C NMR spectra of 1-(trifluoromethyl)-6*H*-6λ^{3}-ioda-2,10b-diazaaceanthrylen-6-yl triflate (**5ao**) in DMSO-d_6.

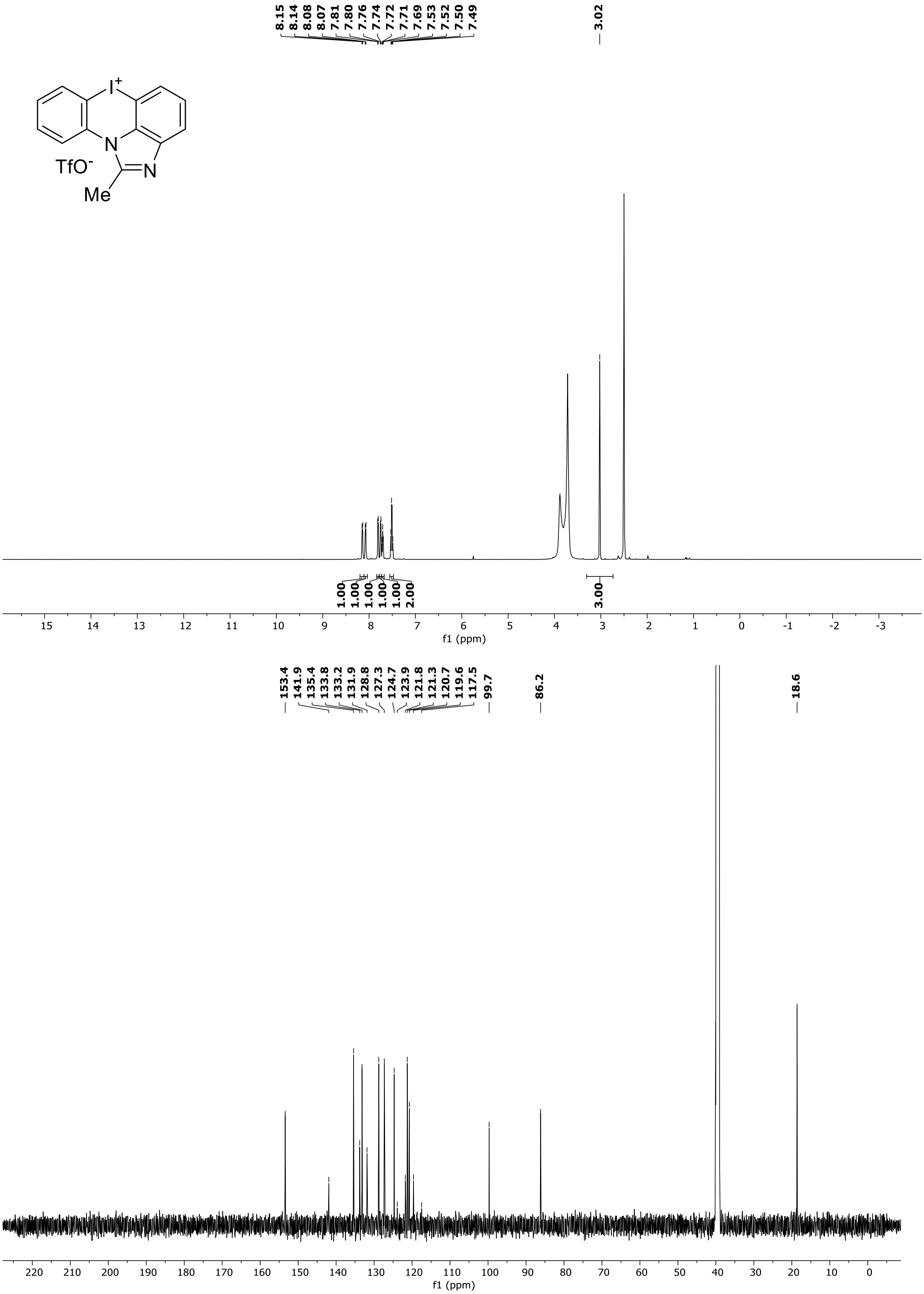

Figure S47: ^{1}H and ^{13}C NMR spectra of 1-methy-6*H*-6λ^{3}-ioda-2,10b-diazaaceanthrylen-6-yl triflate (**5ap**) in DMSO-d_6.

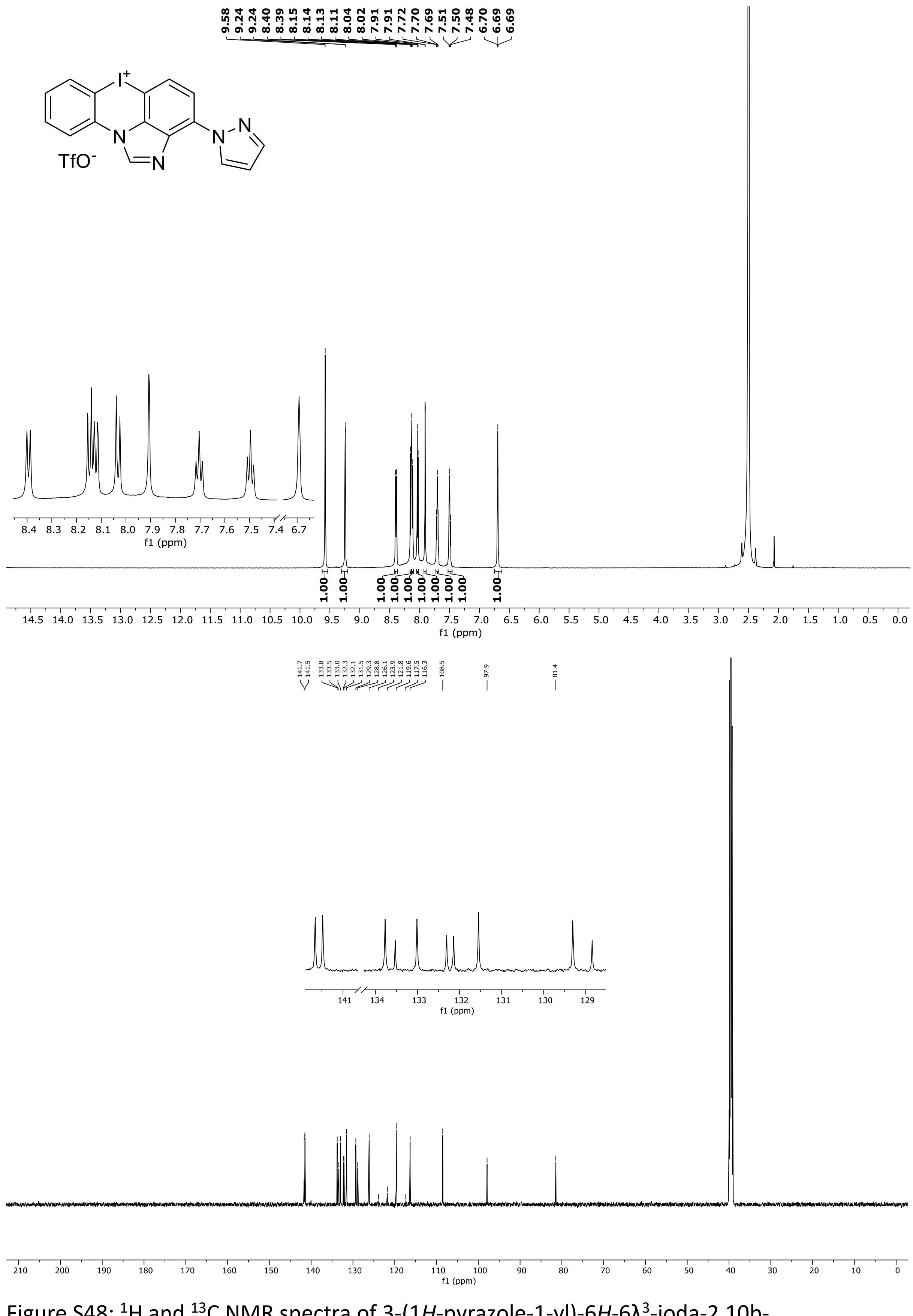

Figure S48: ^{1}H and ^{13}C NMR spectra of 3-(1*H*-pyrazole-1-yl)-6*H*-6λ^{3}-ioda-2,10b-diazaaceanthrylen-6-yl triflate (**5aq**) in DMSO-d_6.

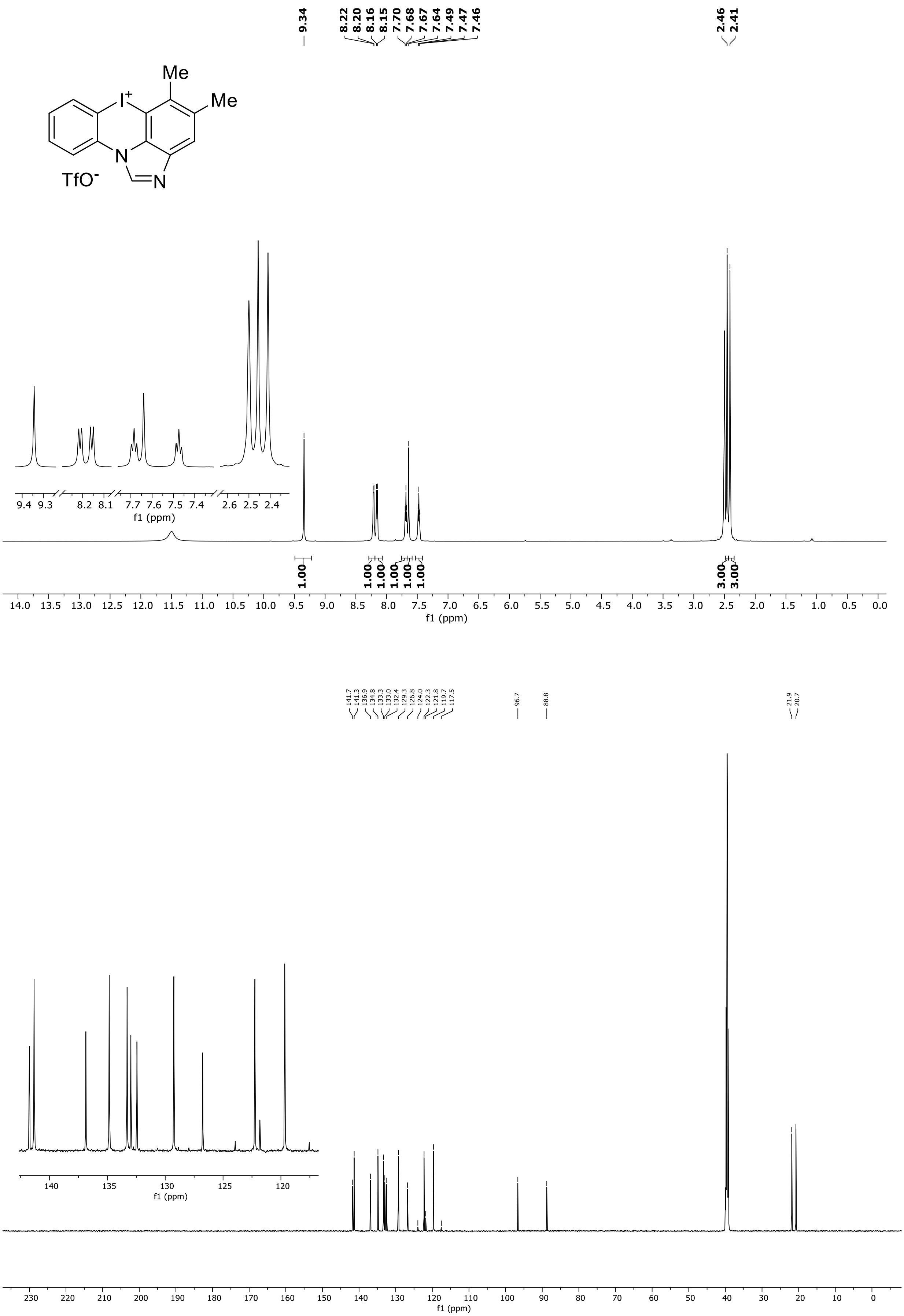

Figure S49: ^{1}H and ^{13}C NMR spectra of 4,5-dimethyl-6*H*-6λ^{3}-ioda-2,10b-diazaaceanthrylen-6-yl triflate (**5ar**) in DMSO-d_6.

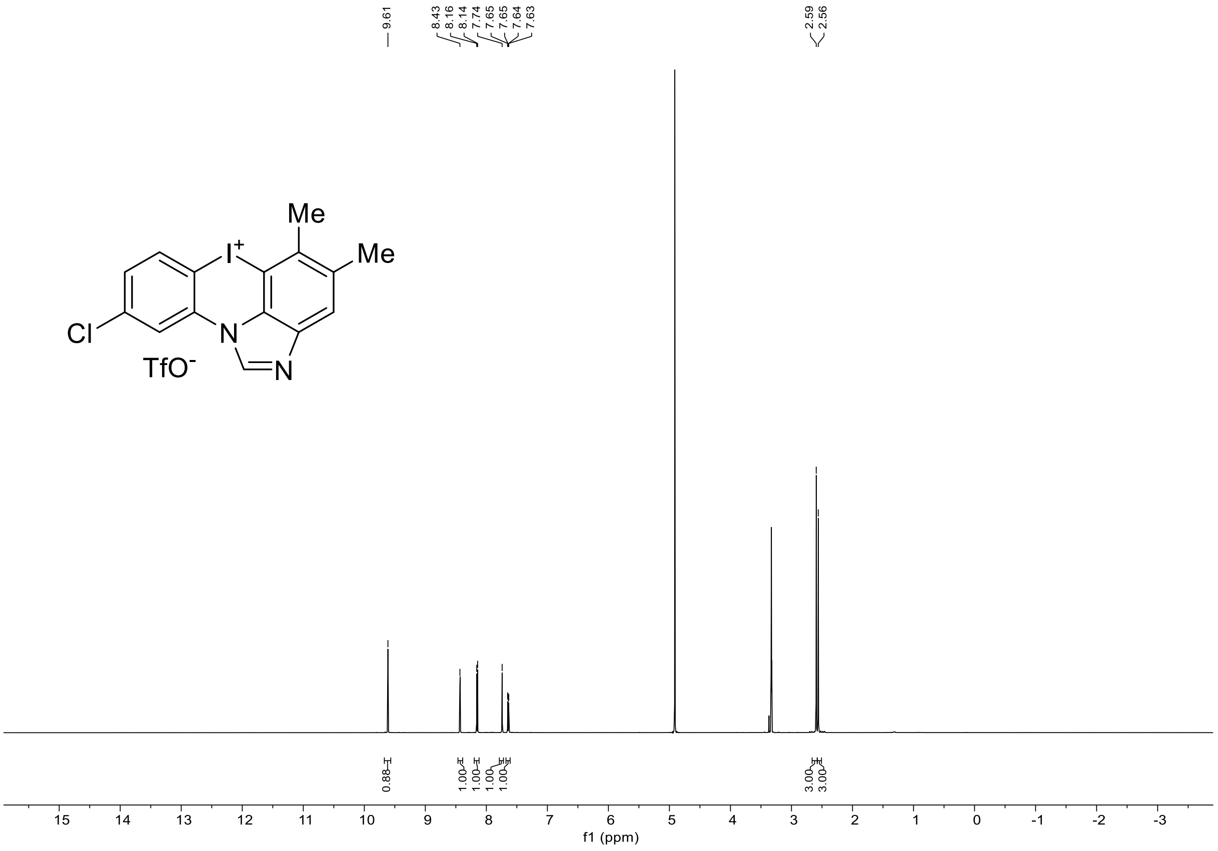

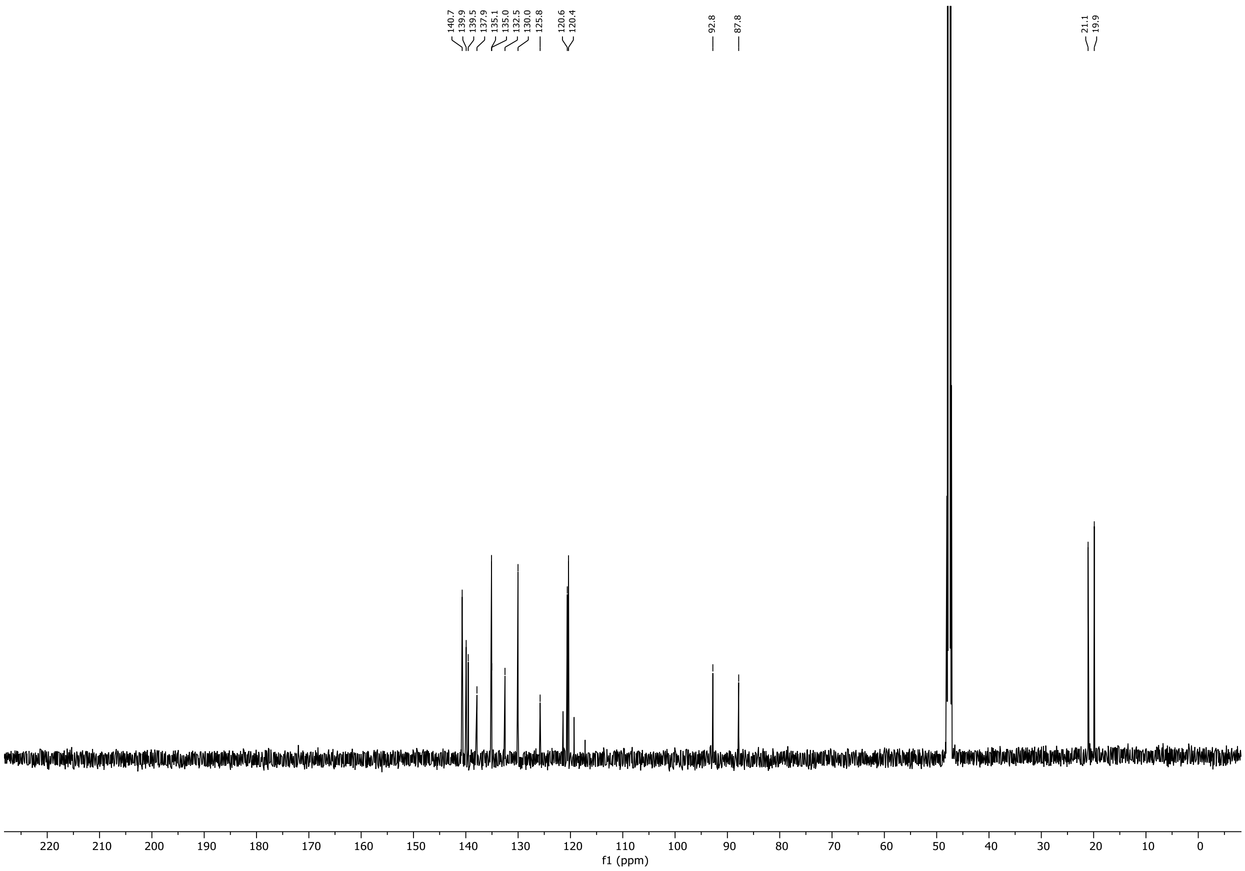

Figure S50: ^{1}H and ^{13}C NMR spectra of 9-chloro-4,5-dimethyl-6*H*-6λ^{3}-ioda-2,10b-diazaaceanthrylen-6-yl triflate (**5at**) in CD_3OD.

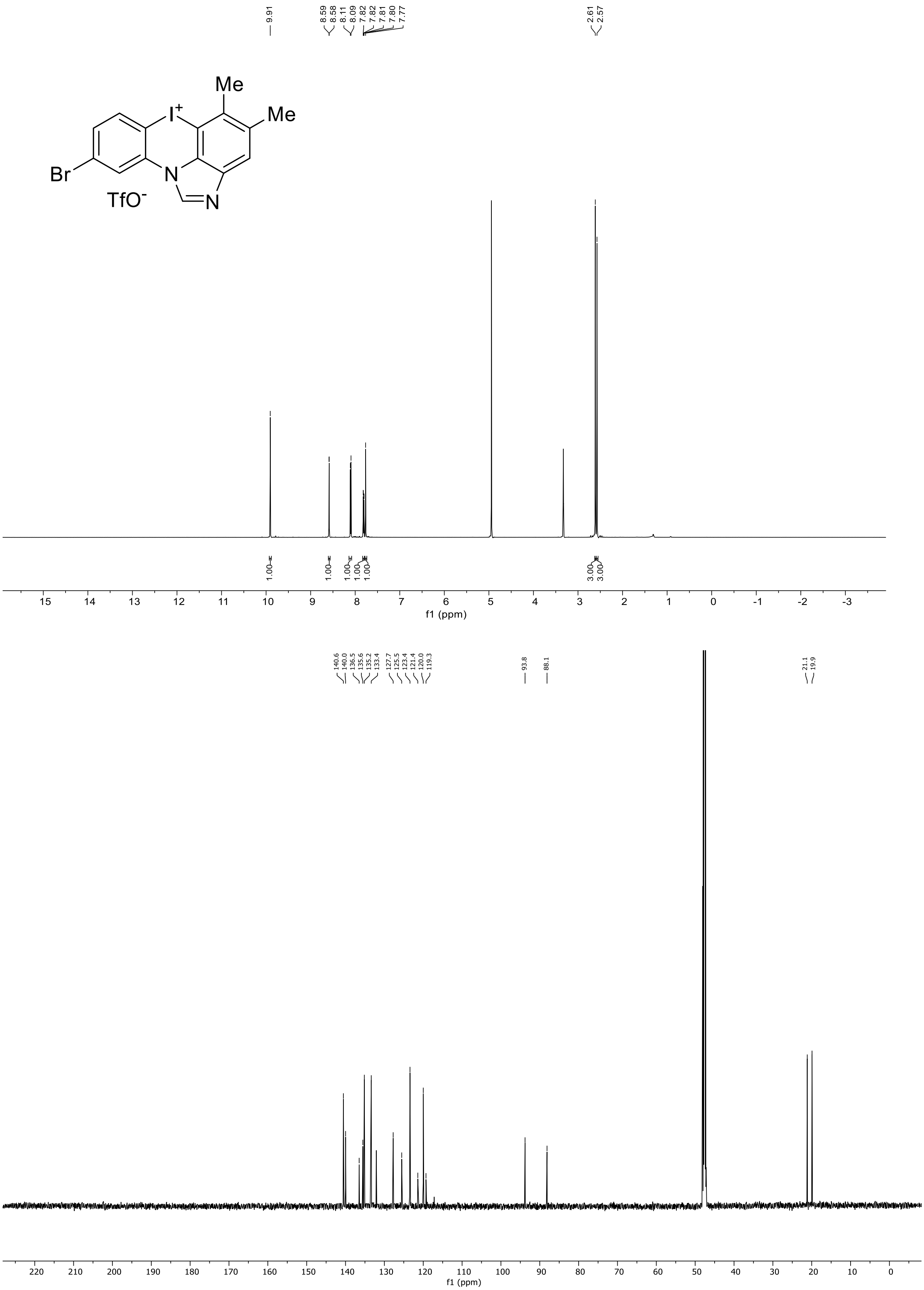

Figure S51: ^{1}H and ^{13}C NMR spectra of 9-bromo-4,5-dimethyl-6*H*-6λ^{3}-ioda-2,10b-diazaaceanthrylen-6-yl triflate (**5au**) in CD_3OD.

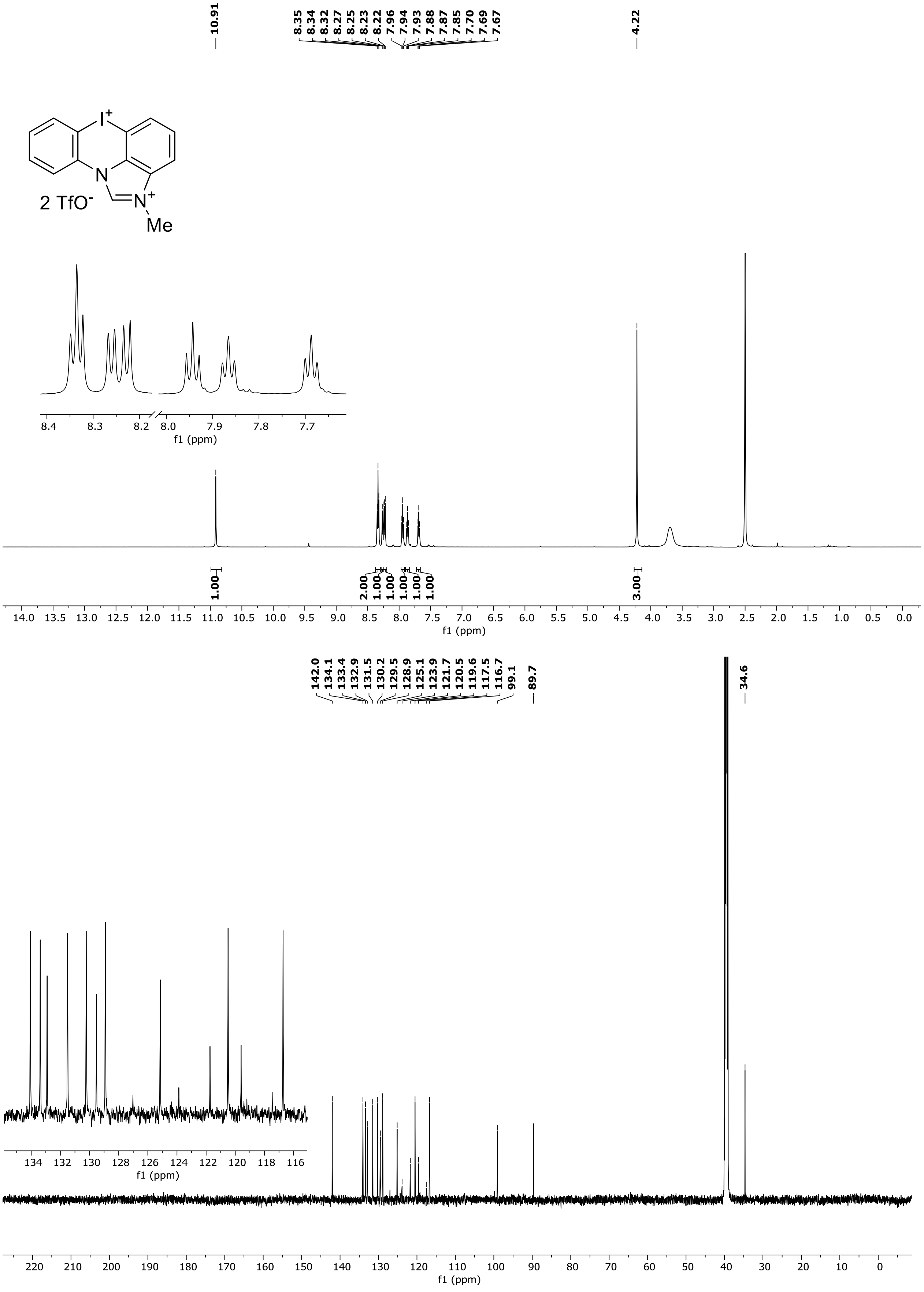

Figure S52: ^{1}H and ^{13}C NMR spectra of 2-methyl-6*H*-6λ^{3}-ioda-2,10b-diazaaceanthrylen-2,6-diium bistriflate (**5av**) in DMSO-d_6.

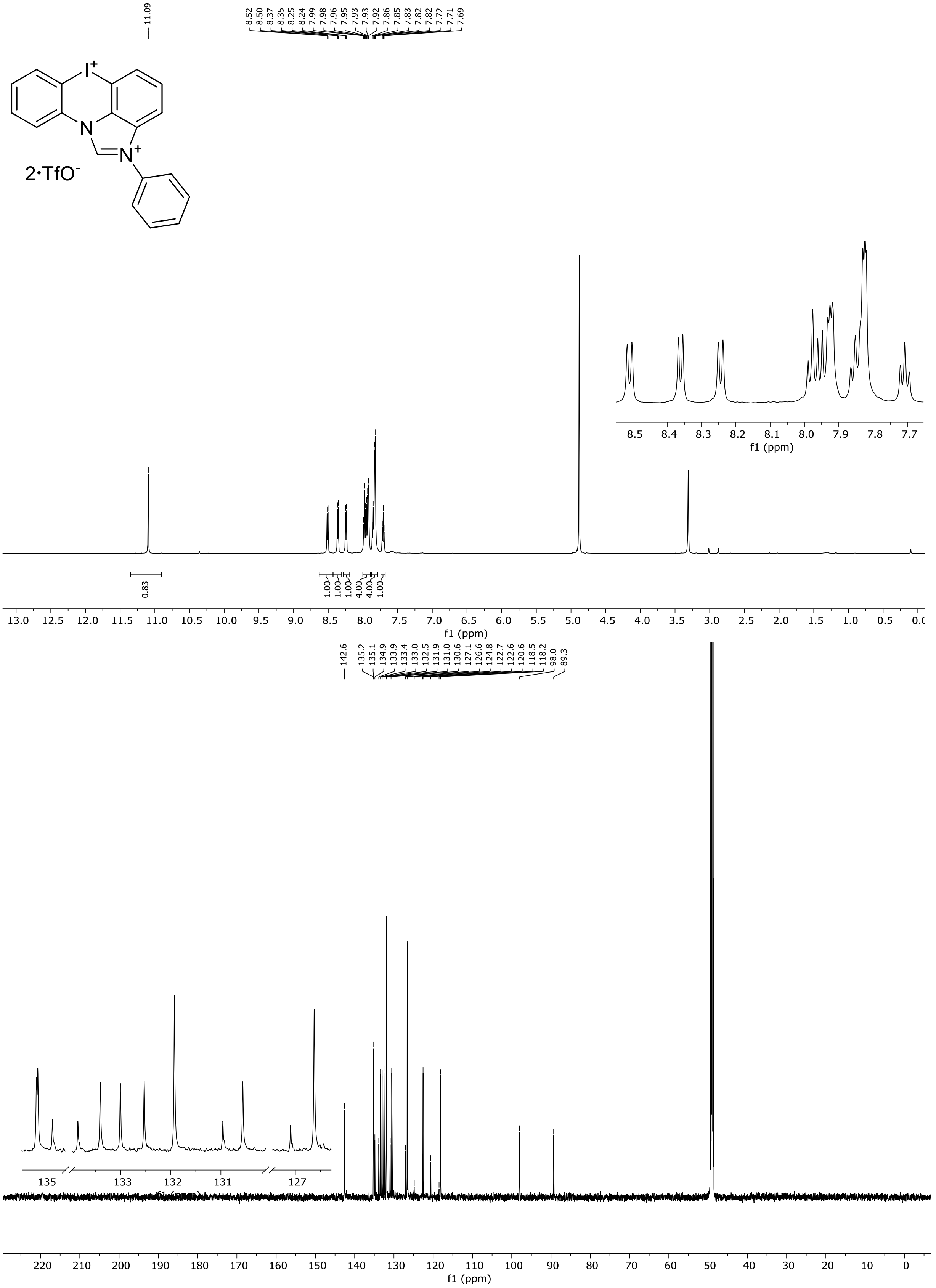

Figure S53: ^{1}H and ^{13}C NMR spectra of 2-phenyl-6*H*-6λ^{3}-ioda-2,10b-diazaaceanthrylen-2,6-diium bistriflate (**5aw**) in CD_3OD.

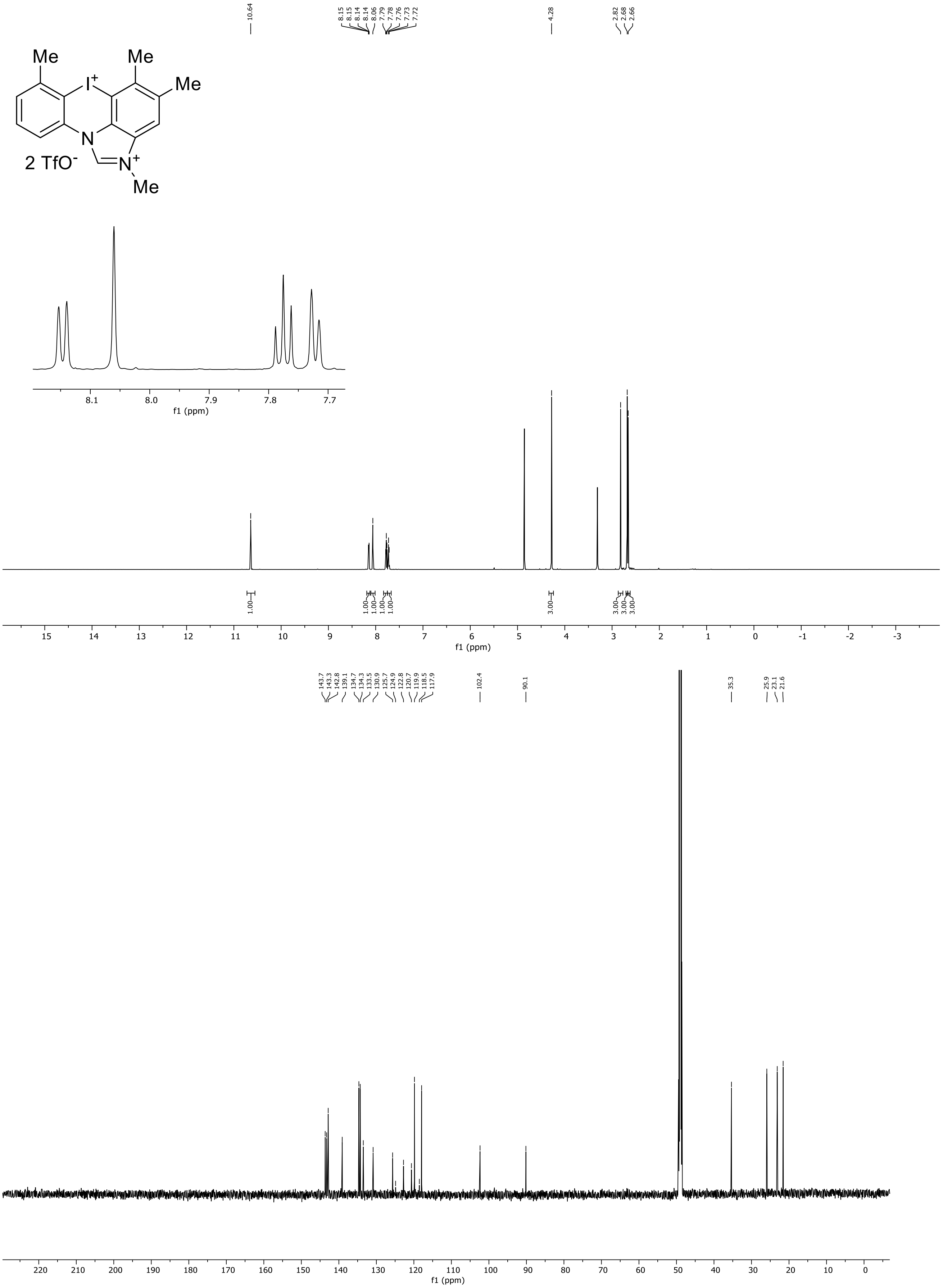

Figure S54: ^{1}H and ^{13}C NMR spectra of 2,4,5,7-tetramethyl-6*H*-6λ^3-ioda-2,10b-diazaaceanthrylen-2,6-diium bistriflate (**5ax**) in CD_3OD.

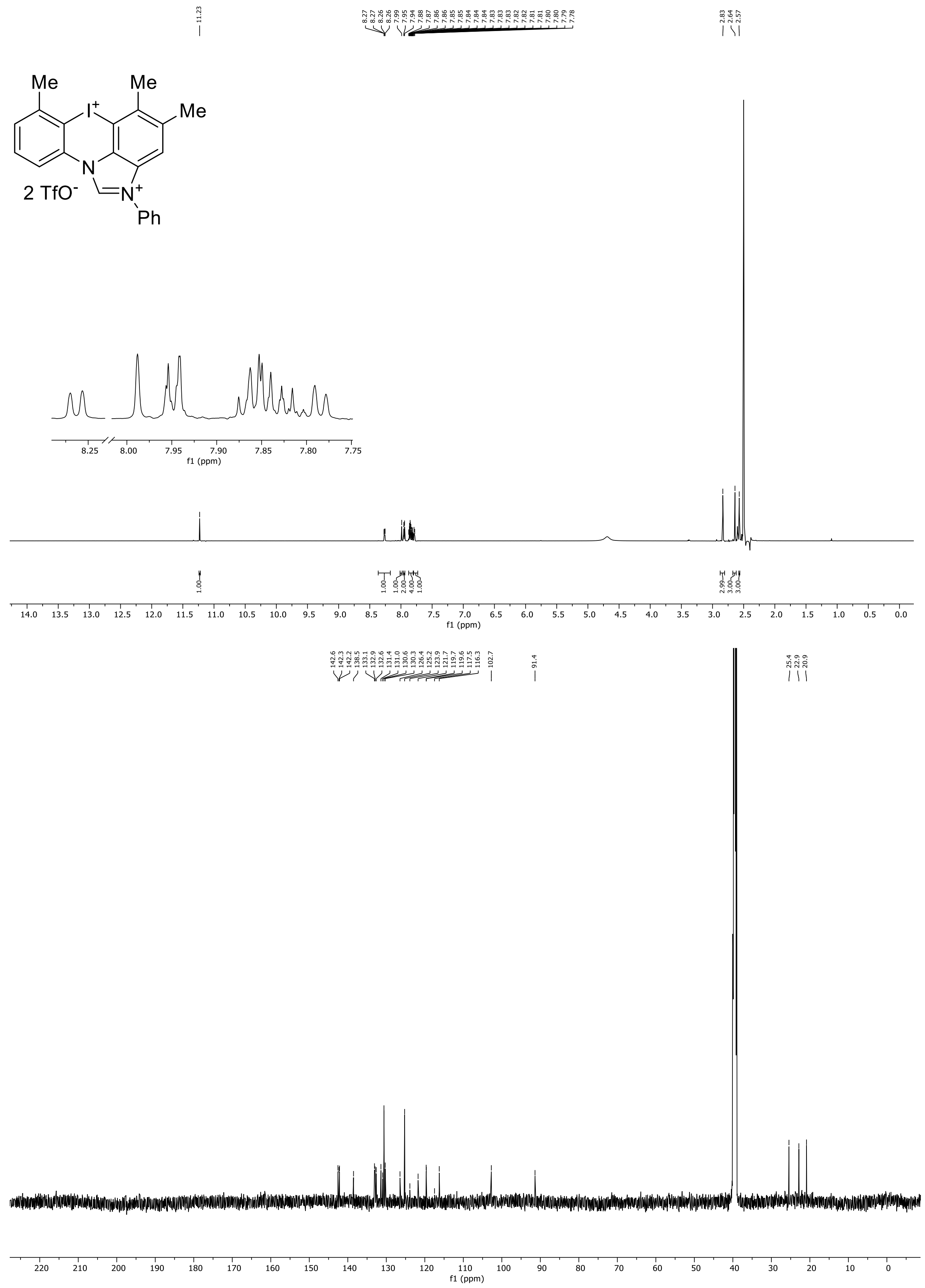

Figure S55: ^{1}H and ^{13}C NMR spectra of 4,5,7-trimethyl-2-phenyl-6*H*-6λ^{3}-ioda-2,10b-diazaaceanthrylen-2,6-diium bistriflate (**5ay**) in DMSO-d_6.

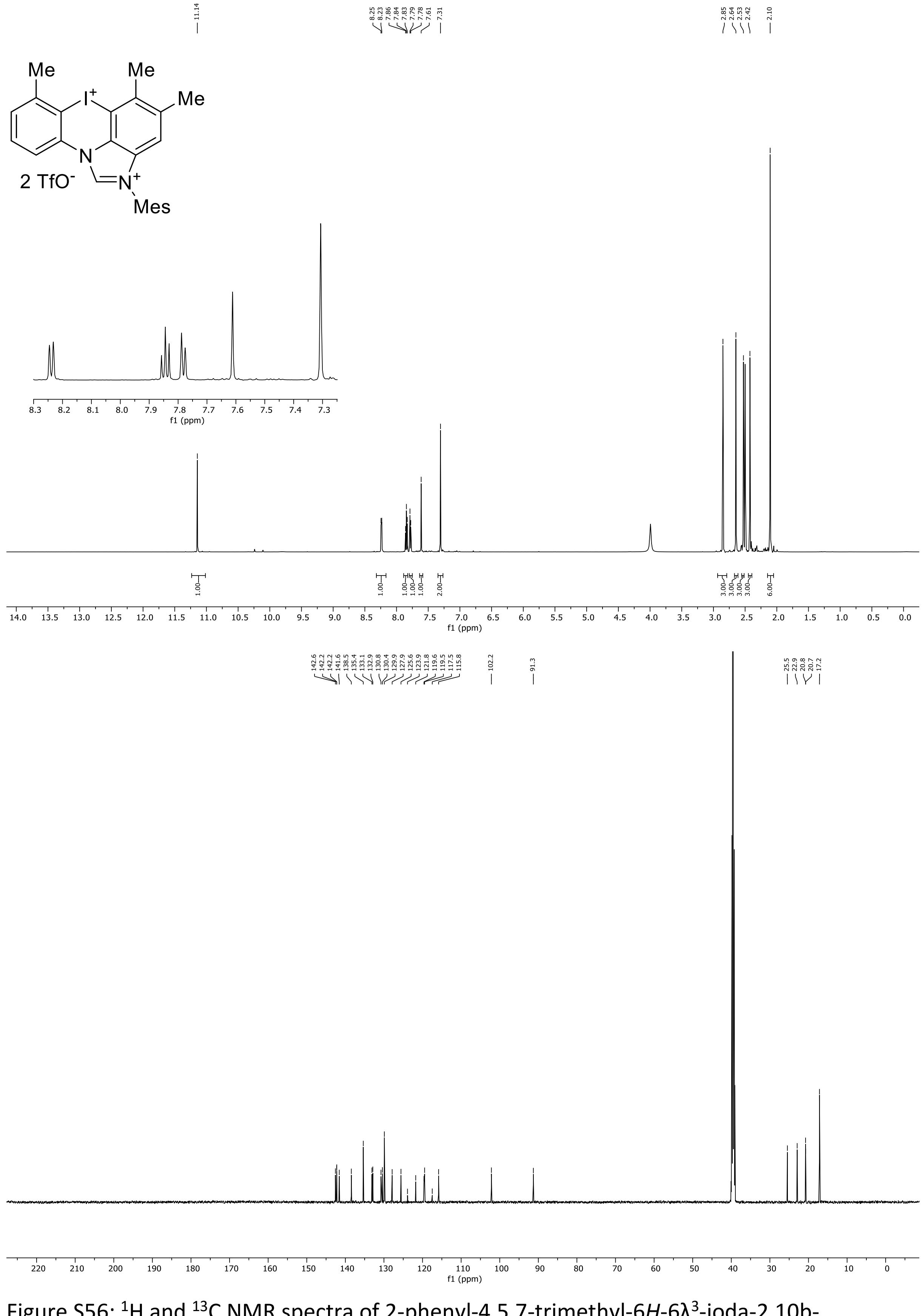

Figure S56: ^{1}H and ^{13}C NMR spectra of 2-phenyl-4,5,7-trimethyl-6*H*-6λ^{3}-ioda-2,10b-diazaaceanthrylen-2,6-diium bistriflate (**5az**) in DMSO-d_6.

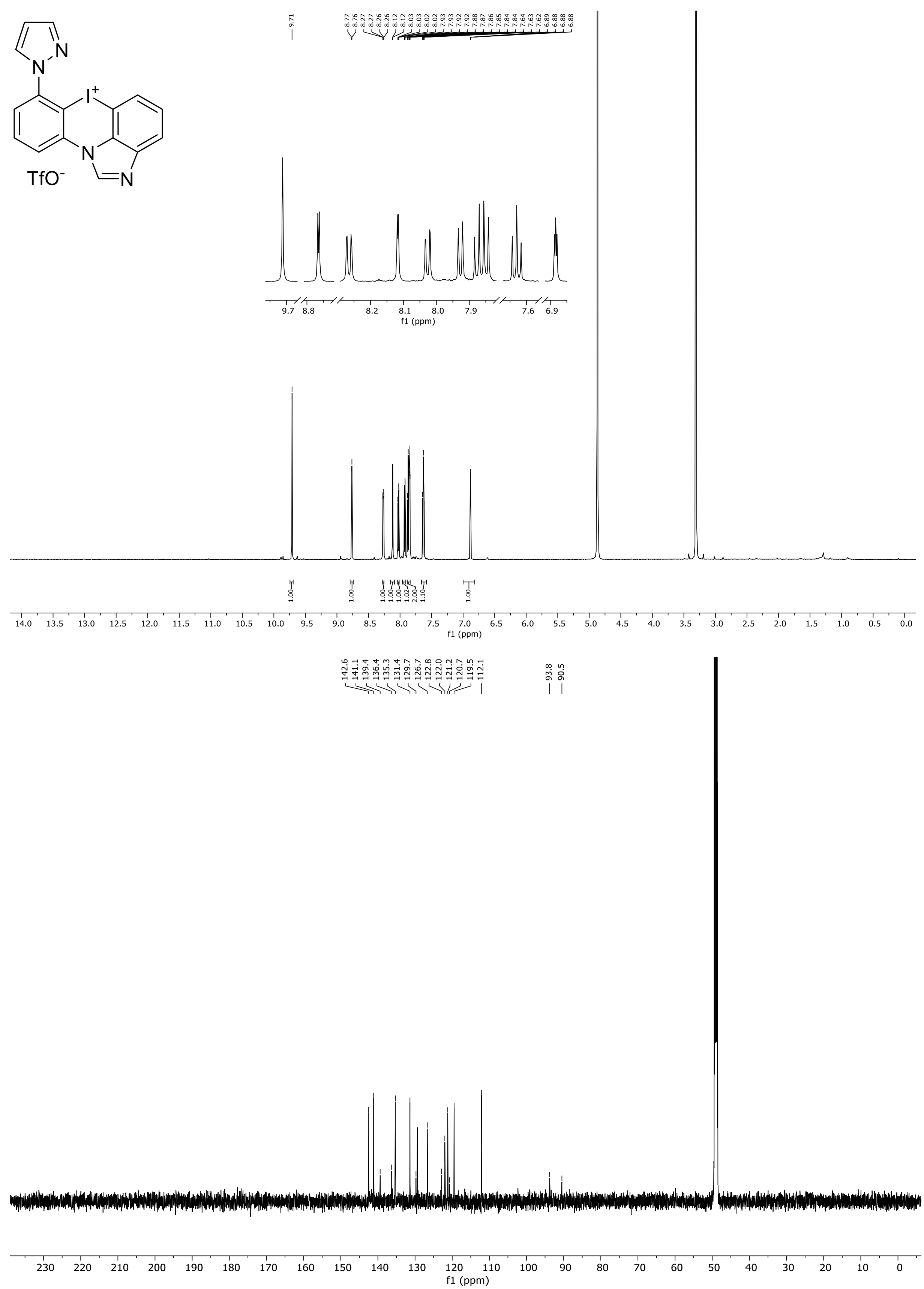

Figure S57: ^{1}H and ^{13}C NMR spectra of 7-(1*H*-pyrazol-1-yl)-6*H*-6λ^3-ioda-2,10b-diazaaceanthrylen-6-ium triflate (**5ba**) in CD_3OD.

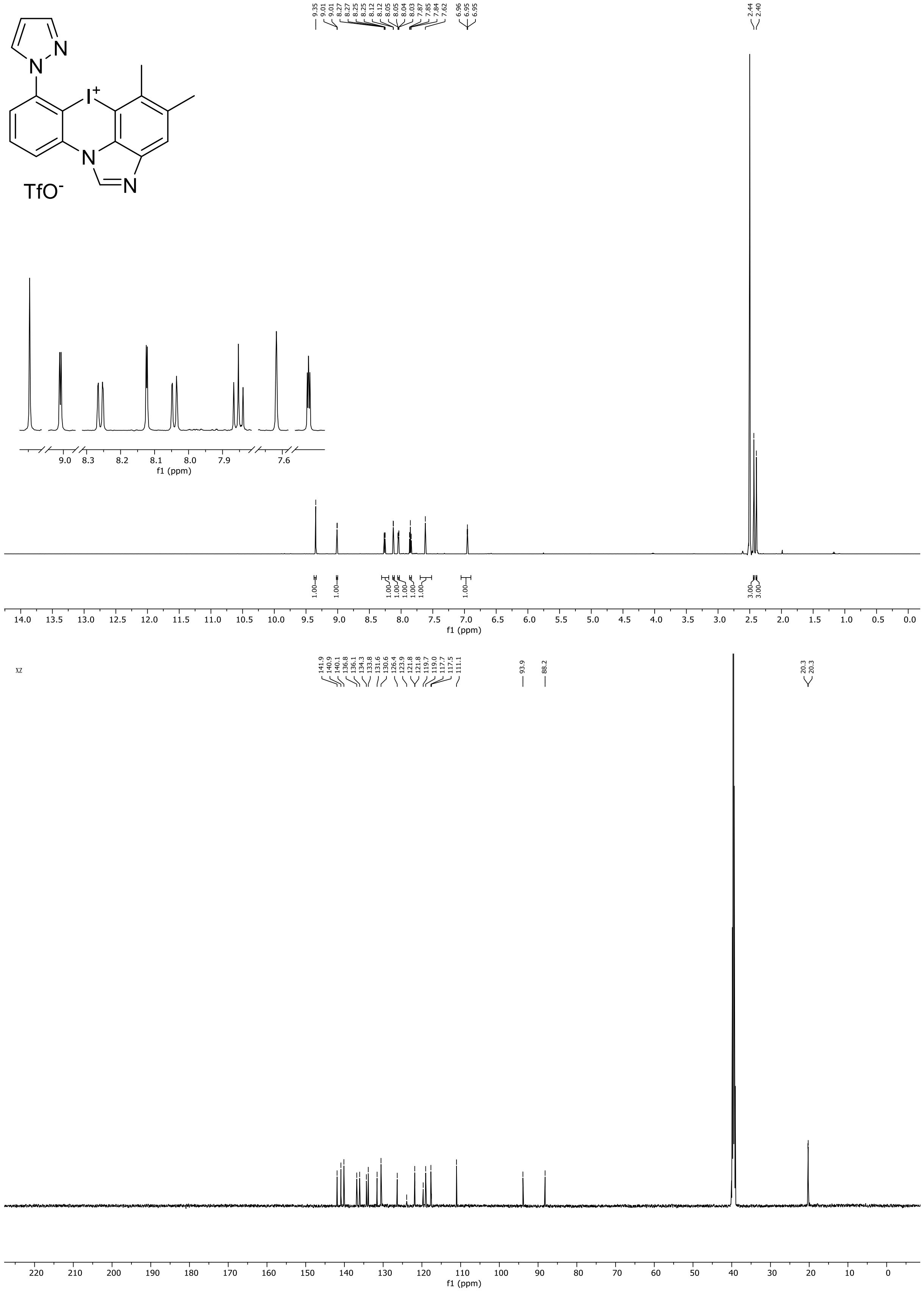

Figure S58: ^{1}H and ^{13}C NMR spectra of 4,5-dimethyl-7-(1*H*-pyrazol-1-yl)-6*H*-6λ^{3}-ioda-2,10b-diazaaceanthrylen-6-ium triflate (**5bb**) in DMSO-d_6.

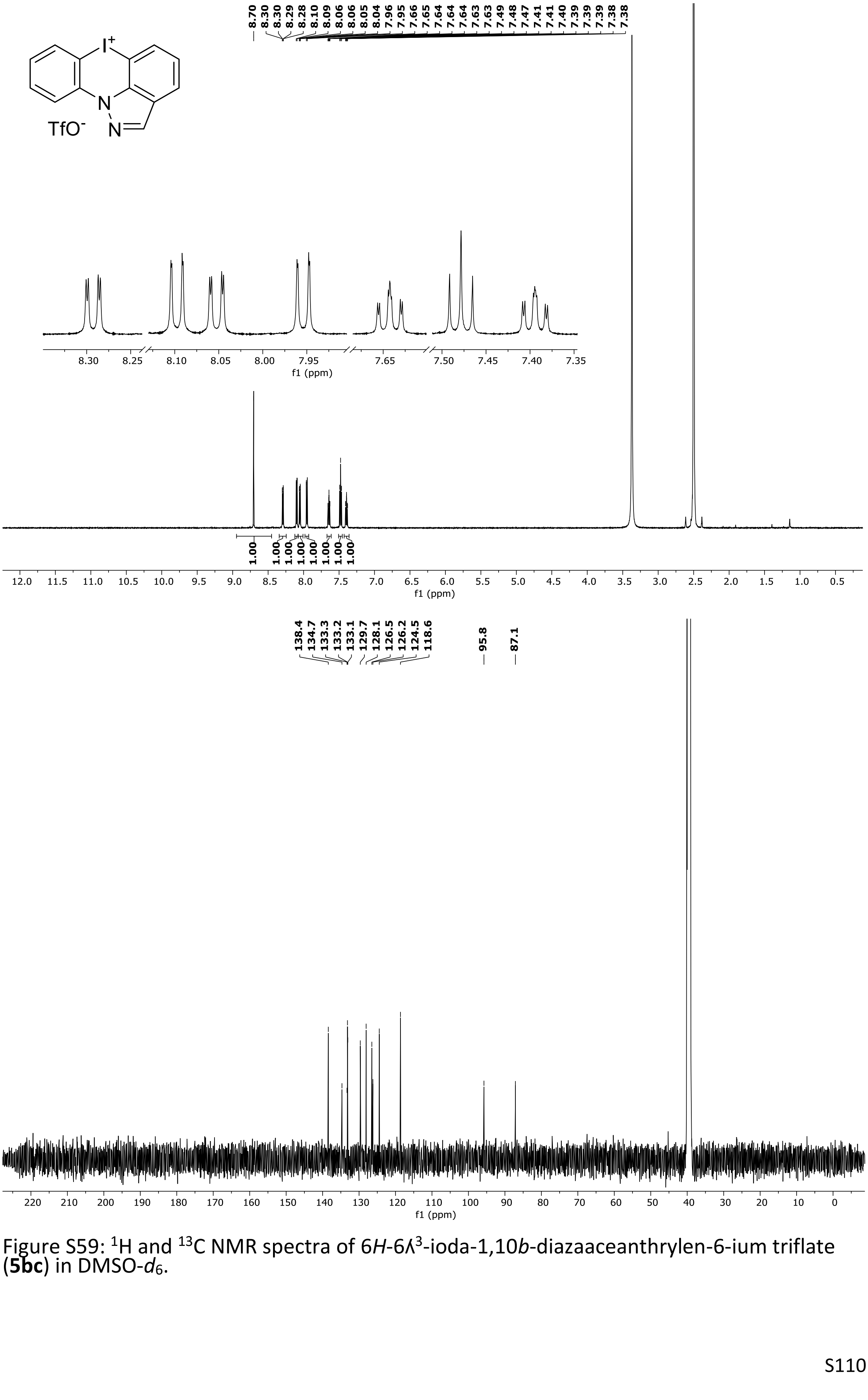

Figure S59: ^{1}H and ^{13}C NMR spectra of 6*H*-6λ^{3}-ioda-1,10*b*-diazaaceanthrylen-6-ium triflate (**5bc**) in DMSO-d_6.

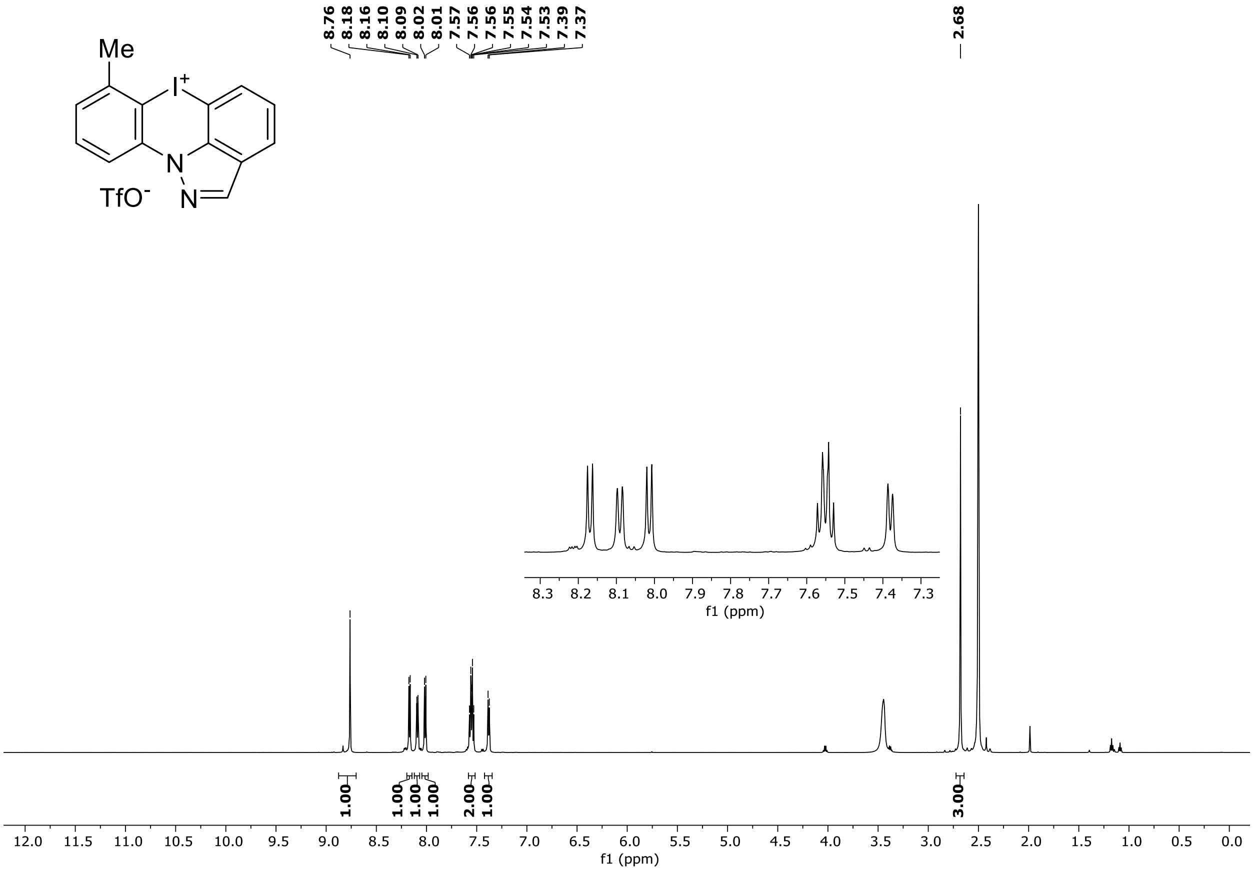

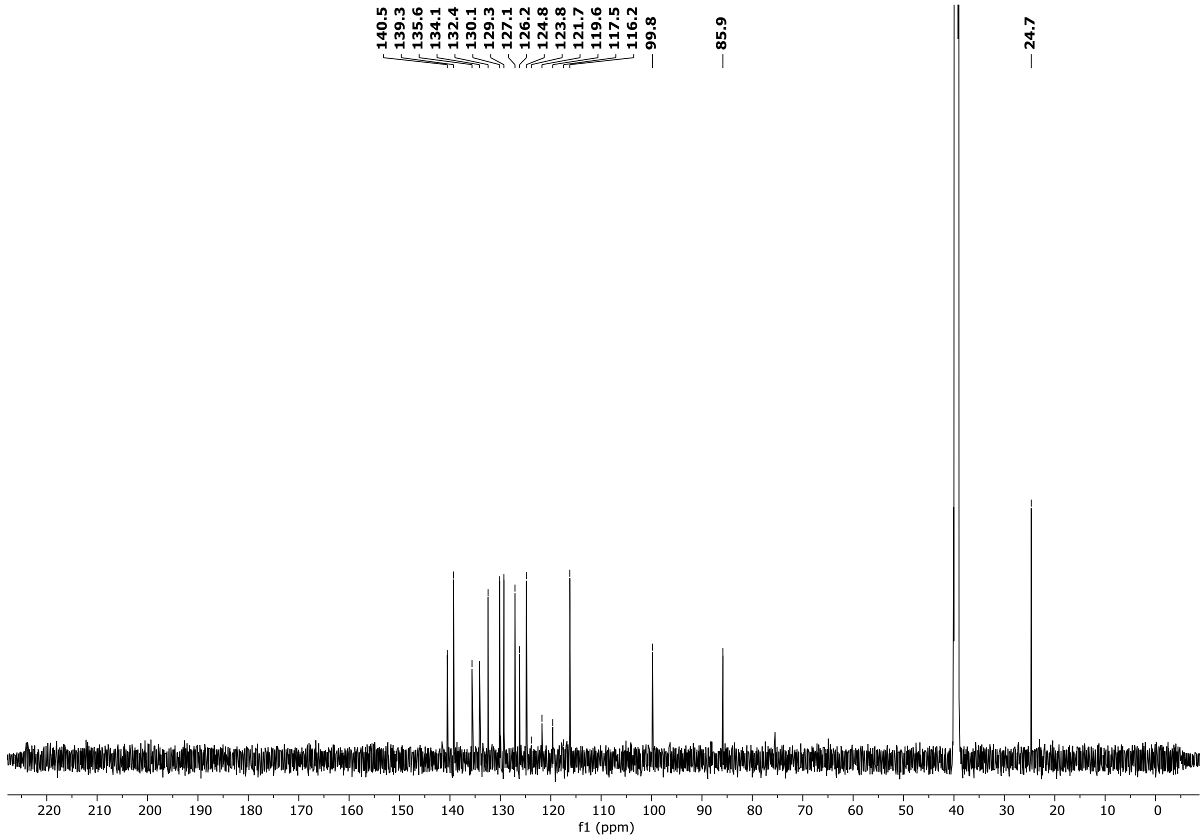

Figure S60: ^{1}H and ^{13}C NMR spectra of 7-methyl-6*H*-6λ^{3}-ioda-1,10*b*-diazaaceanthrylen-6-ium triflate (**5bd**) in DMSO-d_6.

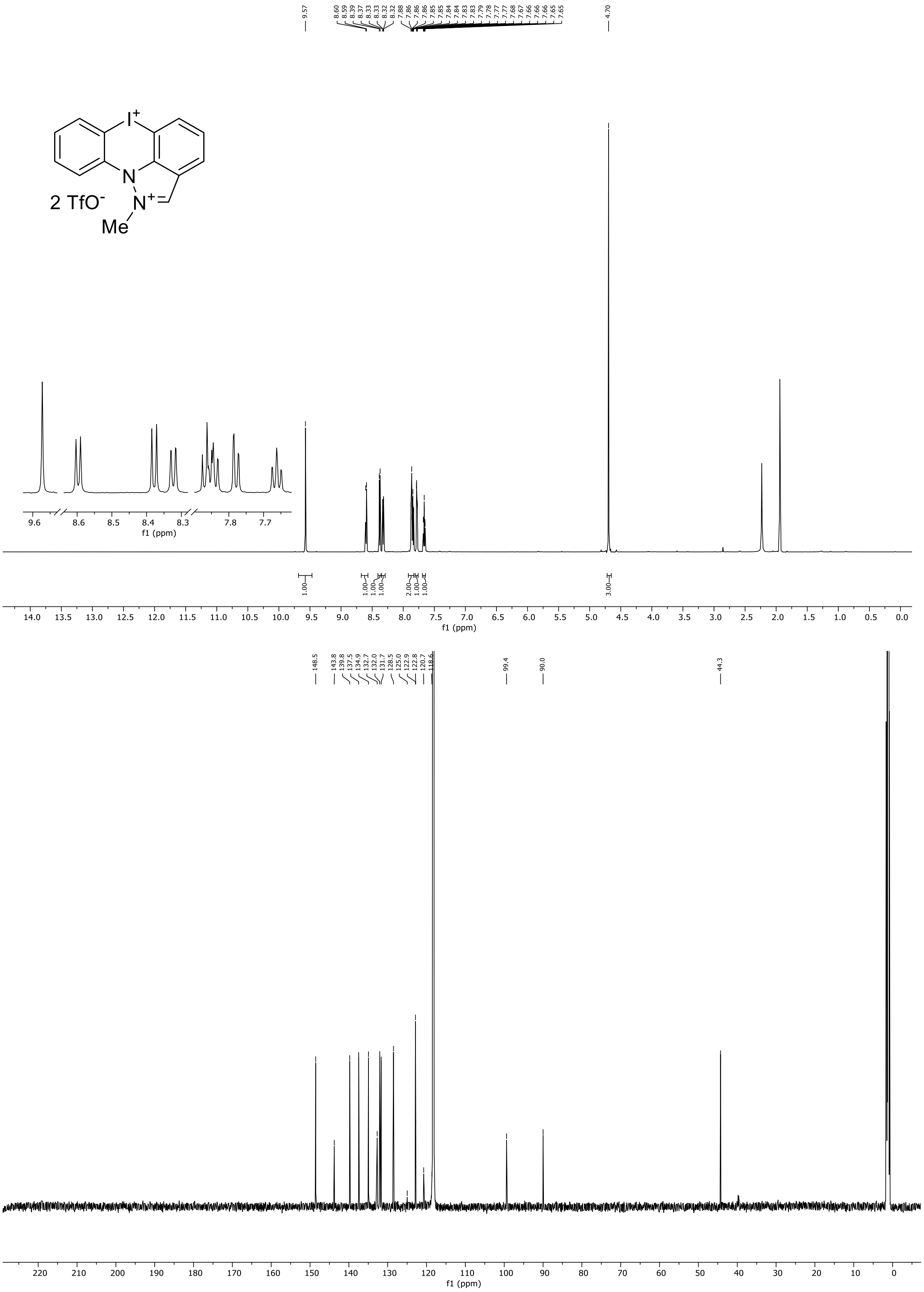

Figure S61: ^{1}H and ^{13}C NMR spectra of 1-methyl-6*H*-6λ^{3}-ioda-1,10*b*-diazaaceanthrylen-1,6-diium bistriflate (**5be**) in MeCN-d_3.

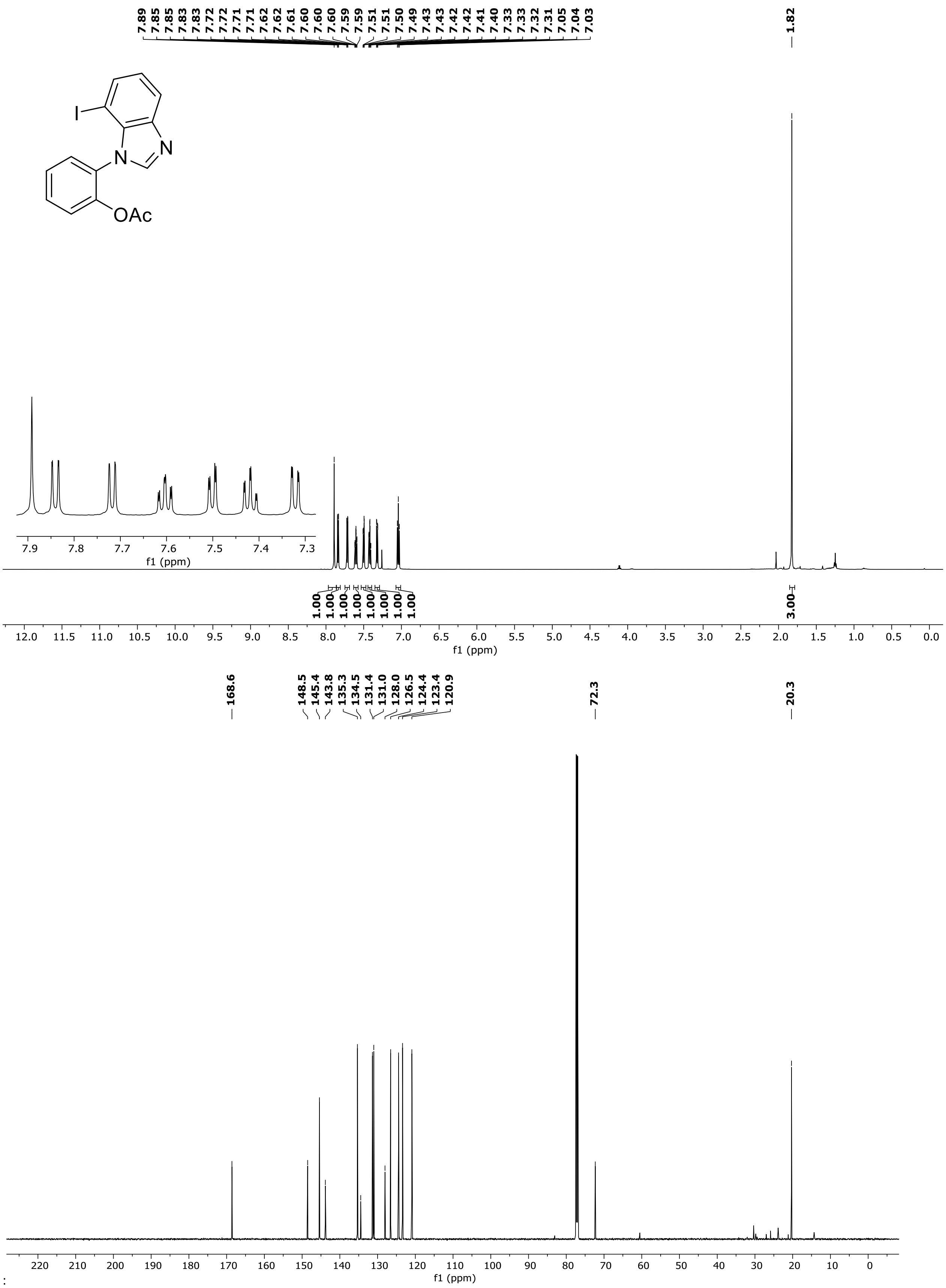

:

Figure S62: ^{1}H and ^{13}C NMR spectra of 2-(7-iodo-1*H*-benzo[*d*]imidazol-1-yl)phenylacetate (**6a**) in $CDCl_3$.

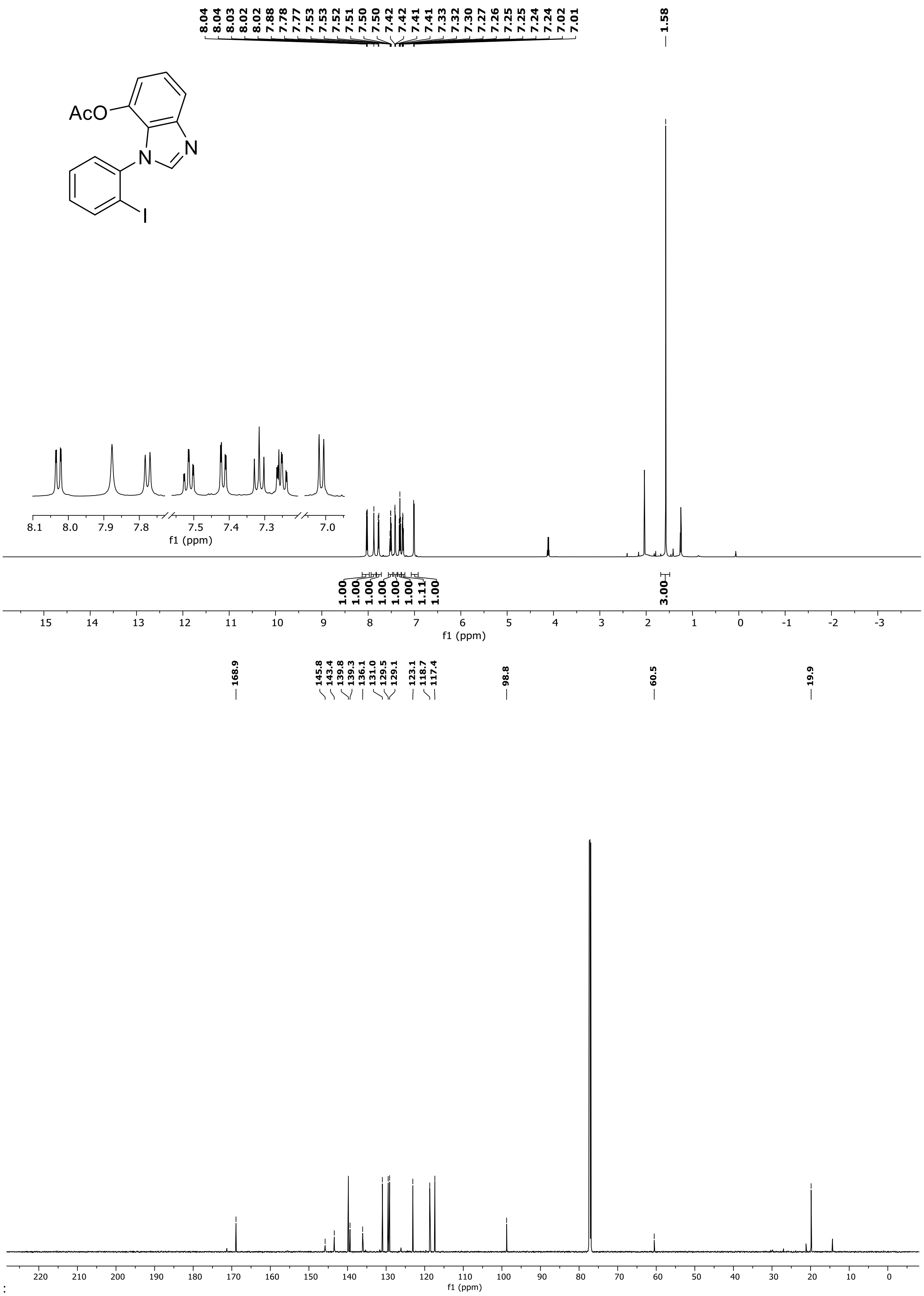

:

Figure S63: ^{1}H and ^{13}C NMR spectra of 1-(2-iodophenyl)-1*H*-benzo[*d*]imidazol-7-ylacetate (**6b**) in $CDCl_3$.

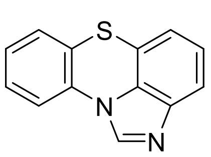

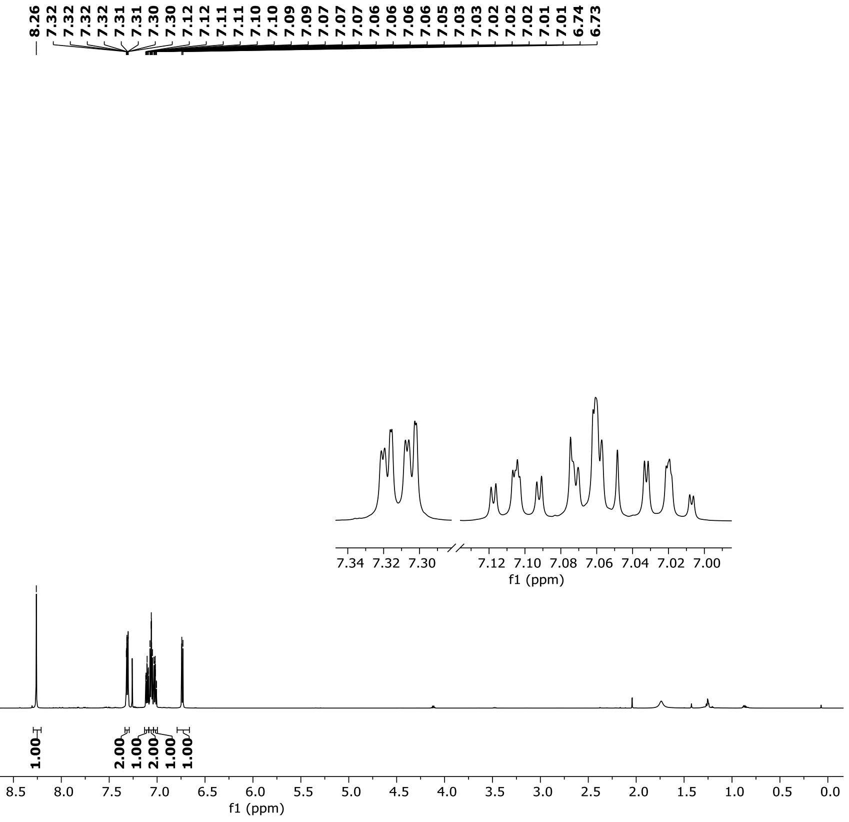

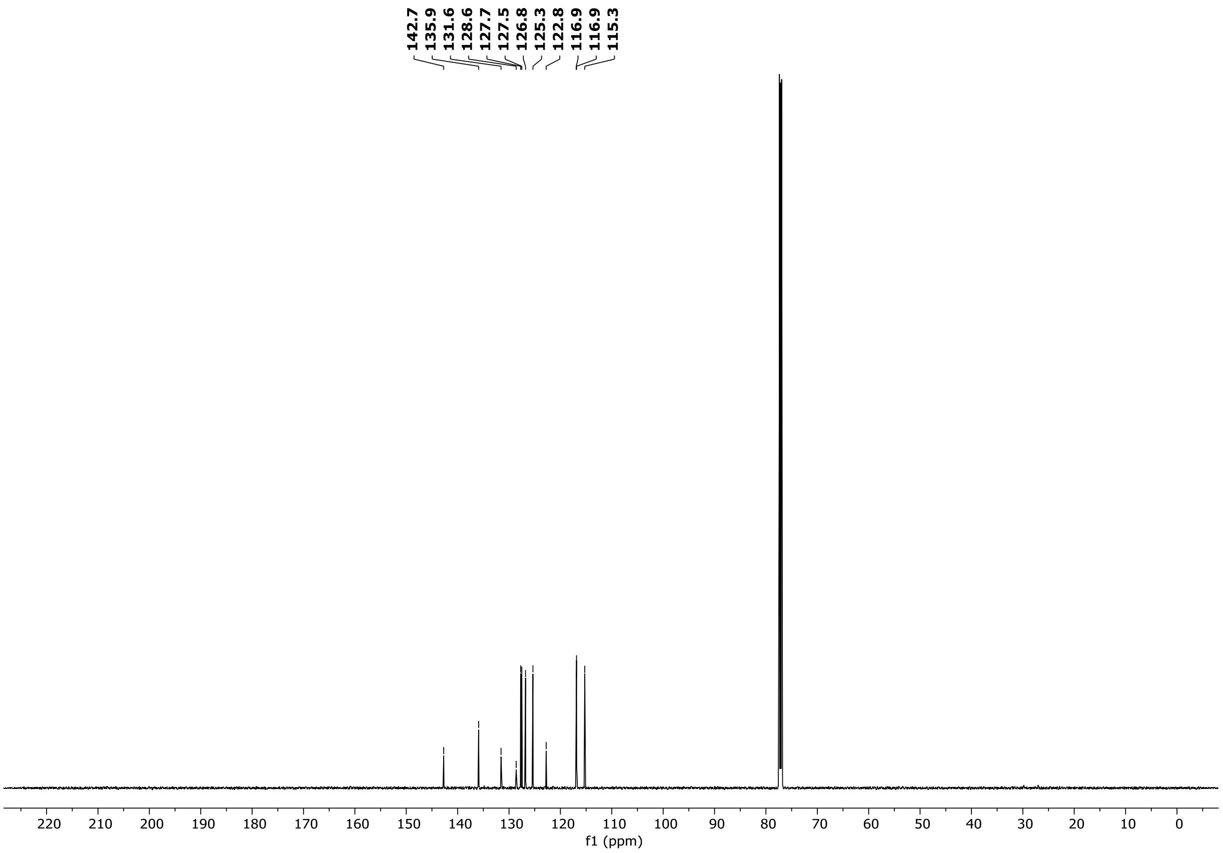

Figure S64: ^{1}H and ^{13}C NMR spectra of imidazo[4,5,1-*kl*]phenothiazine (**7a**) in $CDCl_3$.

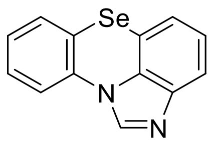

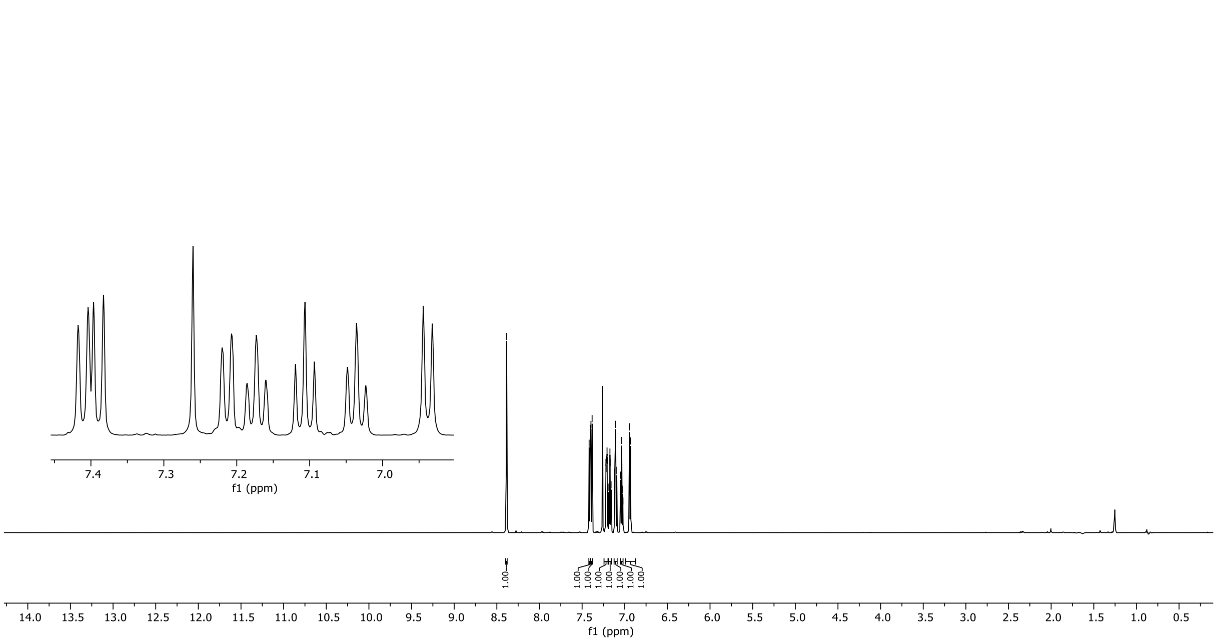

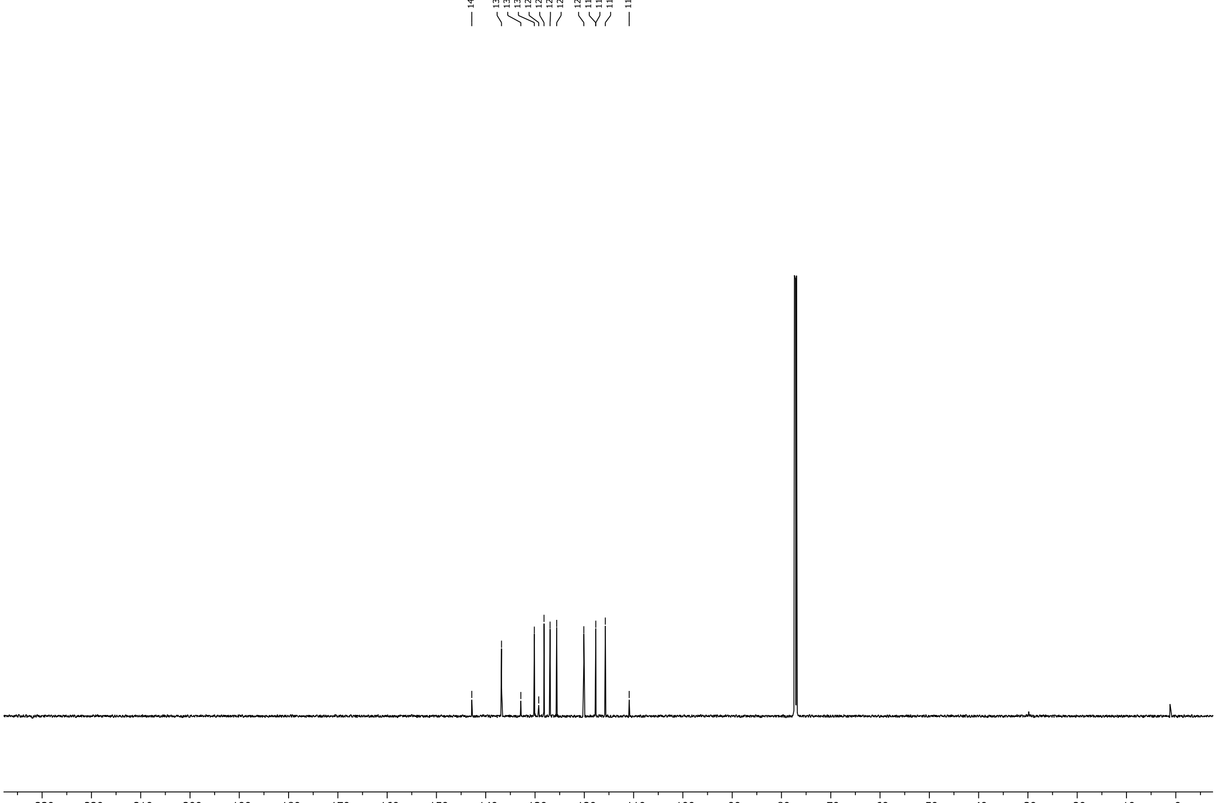

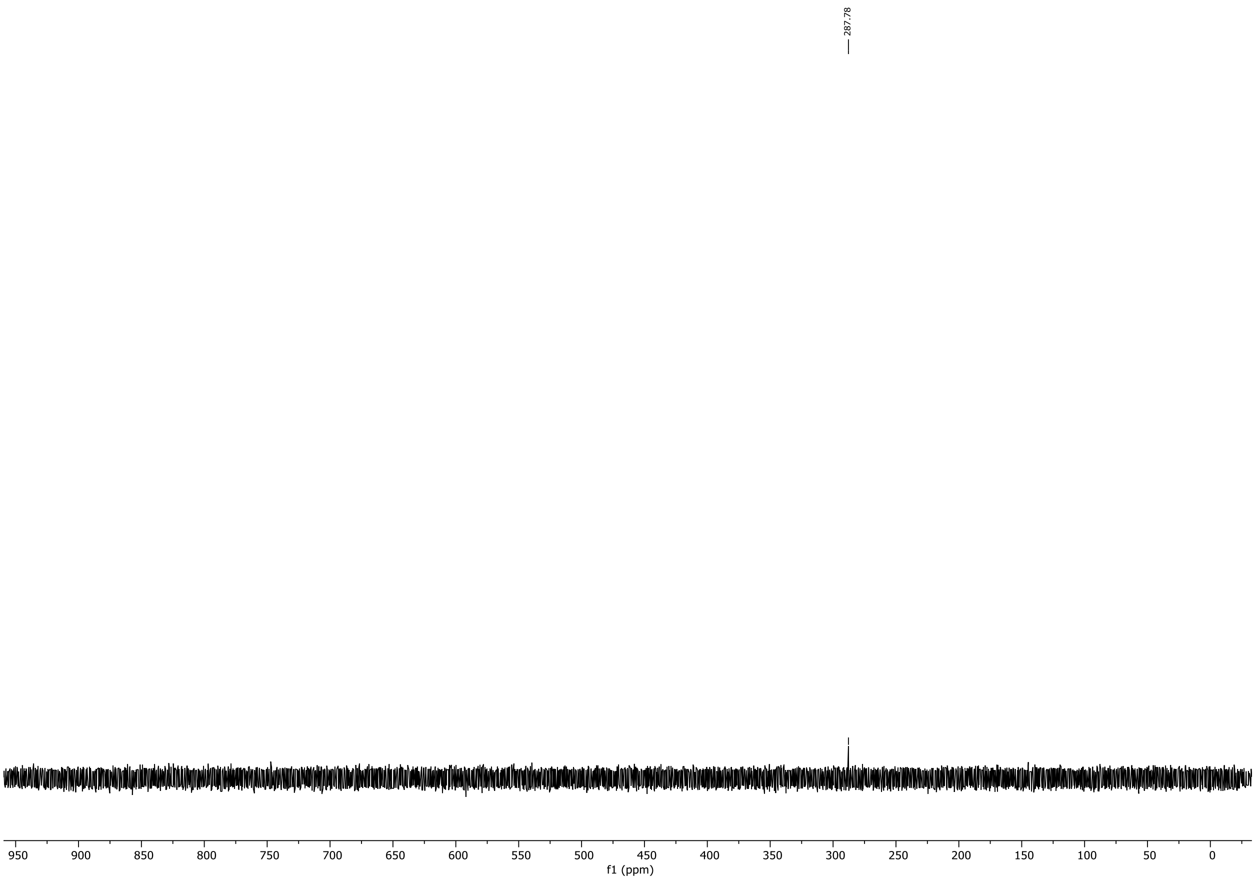

Figure S65: ^{1}H, ^{13}C and ^{77}Se NMR spectra of imidazo[4,5,1-*kl*]phenoselenazine (**7b**) in $CDCl_3$.

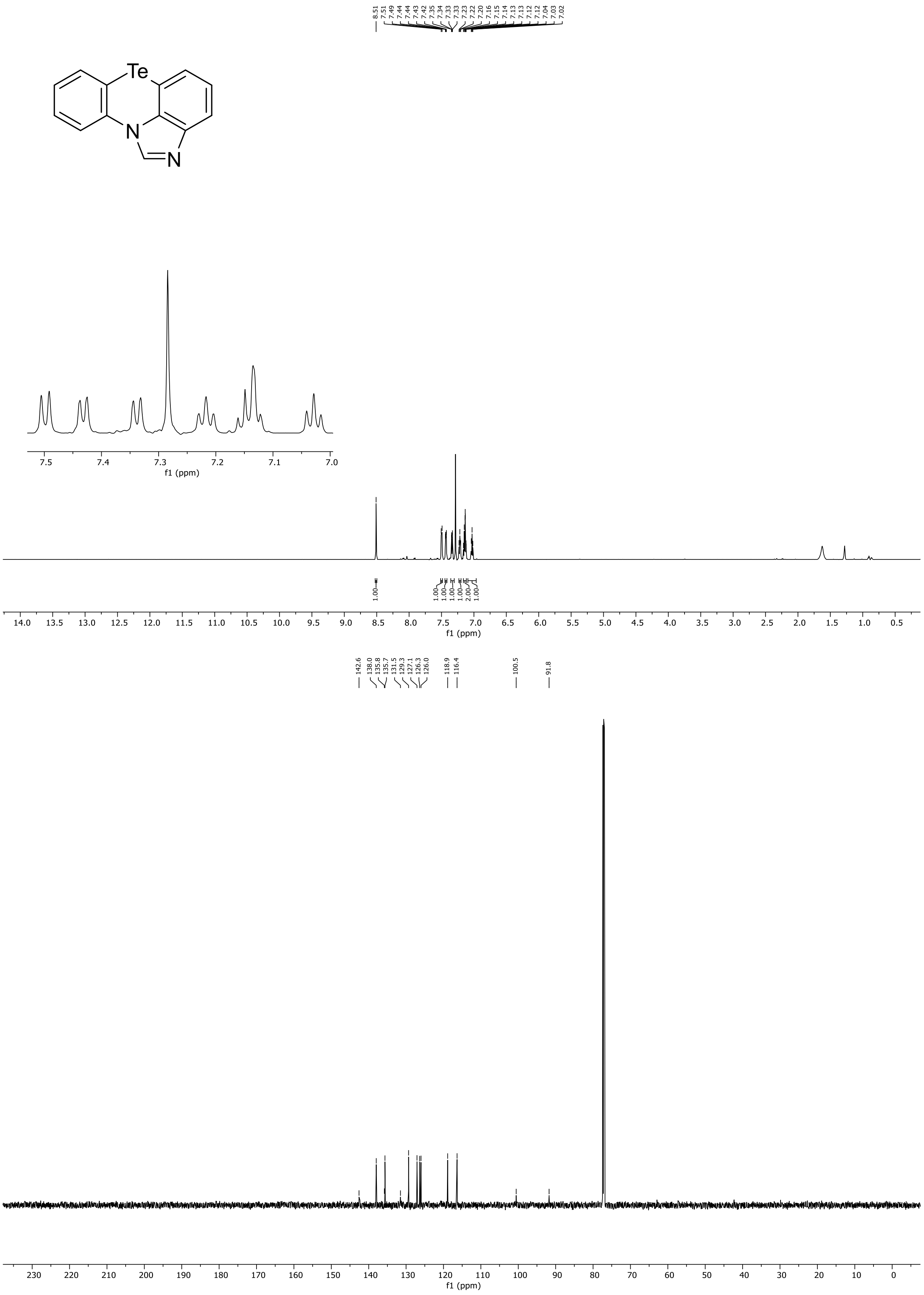

Figure S66: ^{1}H and ^{13}C NMR spectra of imidazo[4,5,1-*kl*]phenotellurazine (**7c**) in $CDCl_3$.

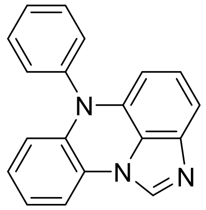

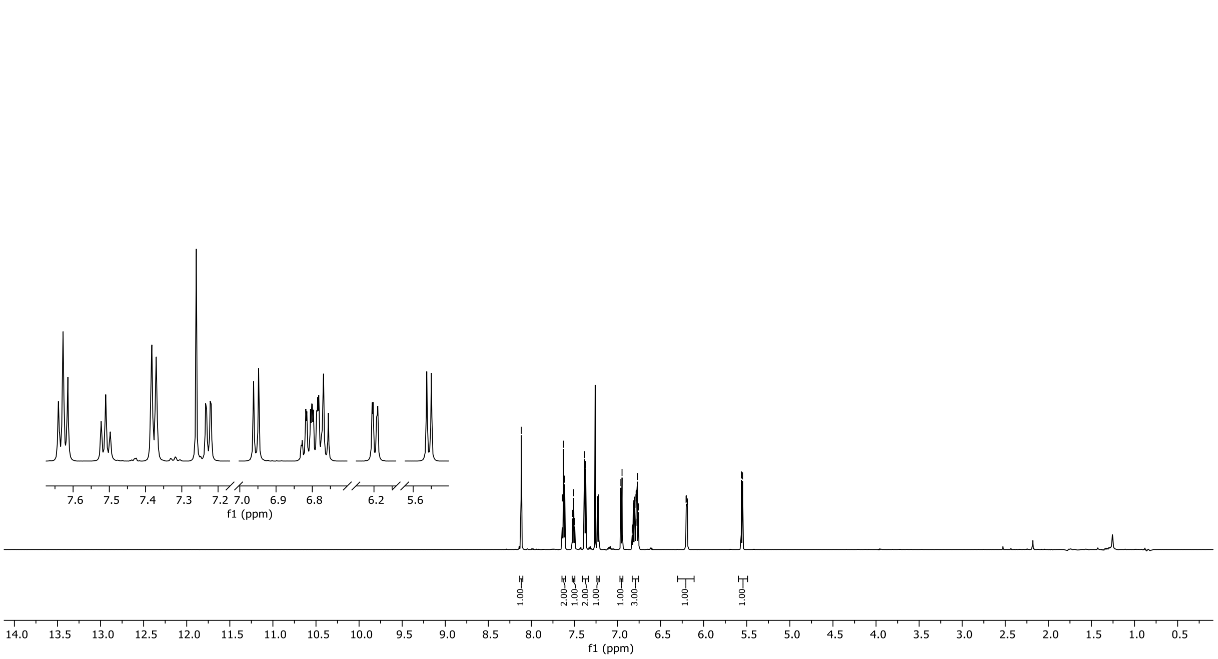

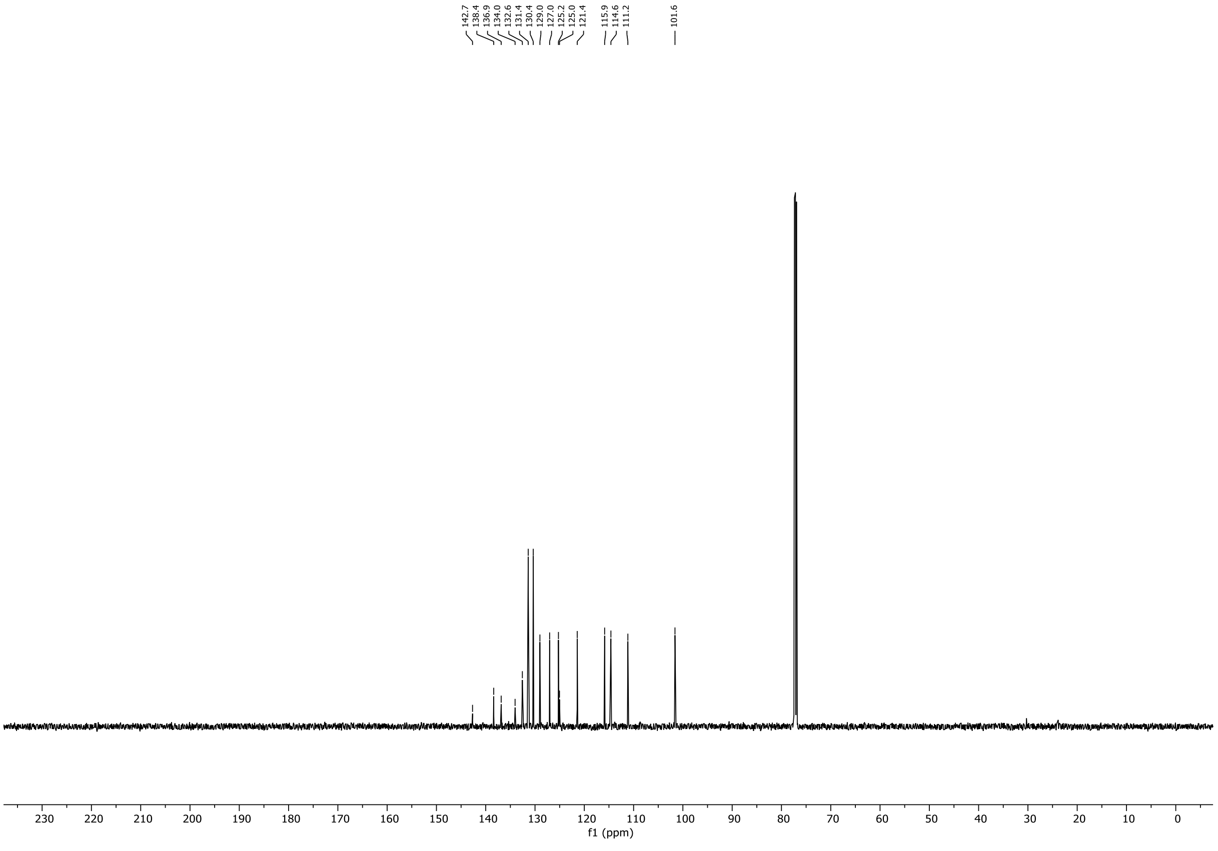

Figure S67: ^{1}H and ^{13}C NMR spectra of 6-phenyl-6*H*-imidazo[4,5,1-*de*]phenazine (**8**) in $CDCl_3$.

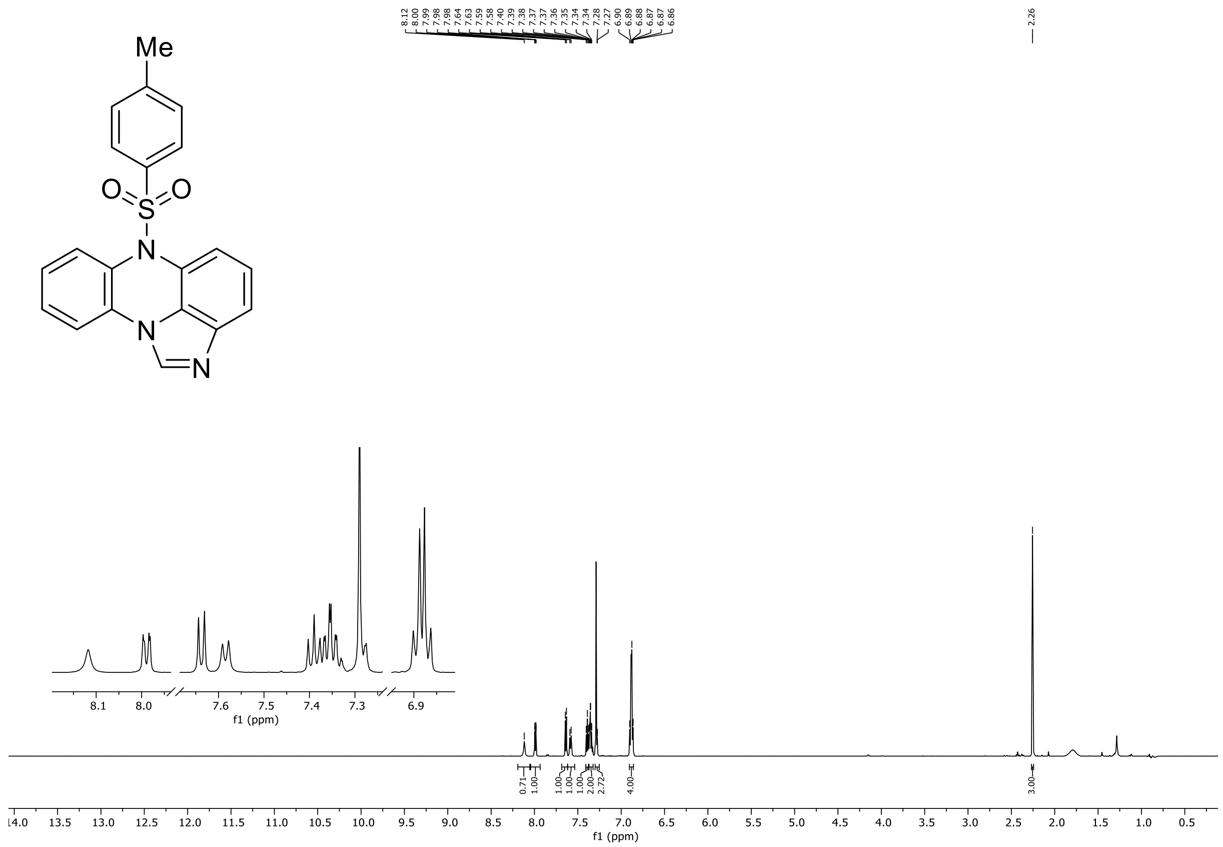

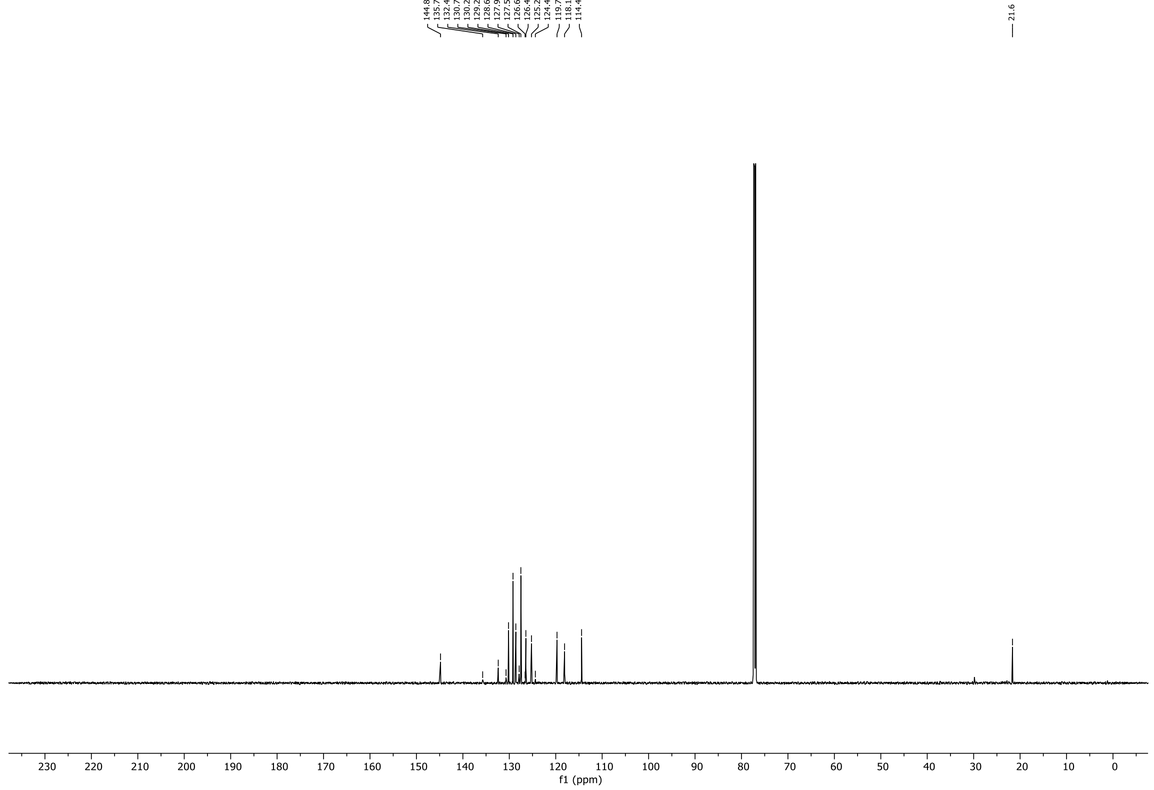

Figure S68: ^{1}H and ^{13}C NMR spectra of 6-tosyl-6*H*-imidazo[4,5,1-*de*]phenazine (**9**) in $CDCl_3$.

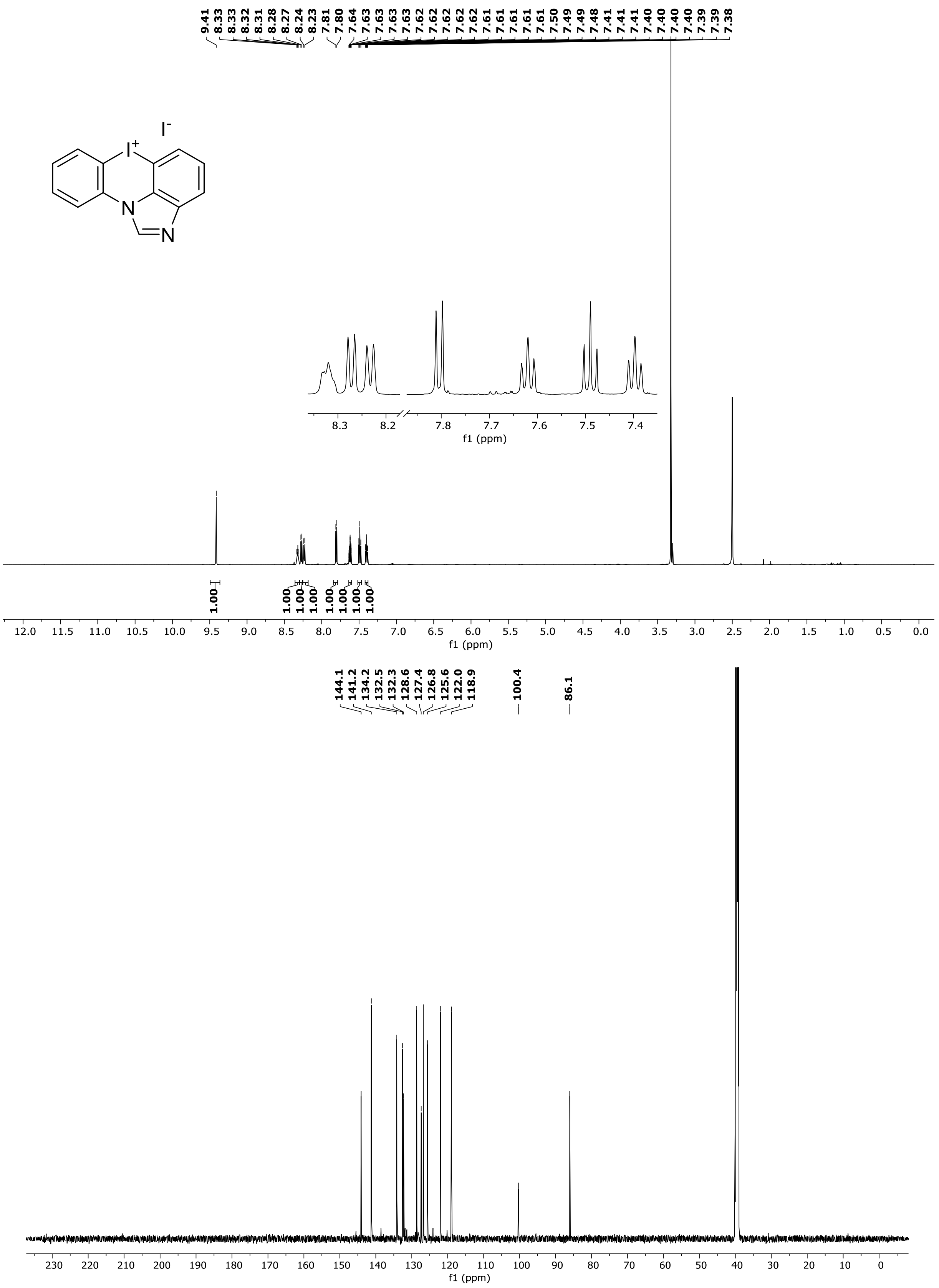

Figure S69: ^{1}H and ^{13}C NMR spectra of 6*H*-6λ^{3}-ioda-2,10b-diazaaceanthrylen-6-ium iodide (**10a**) in DMSO-d_6.

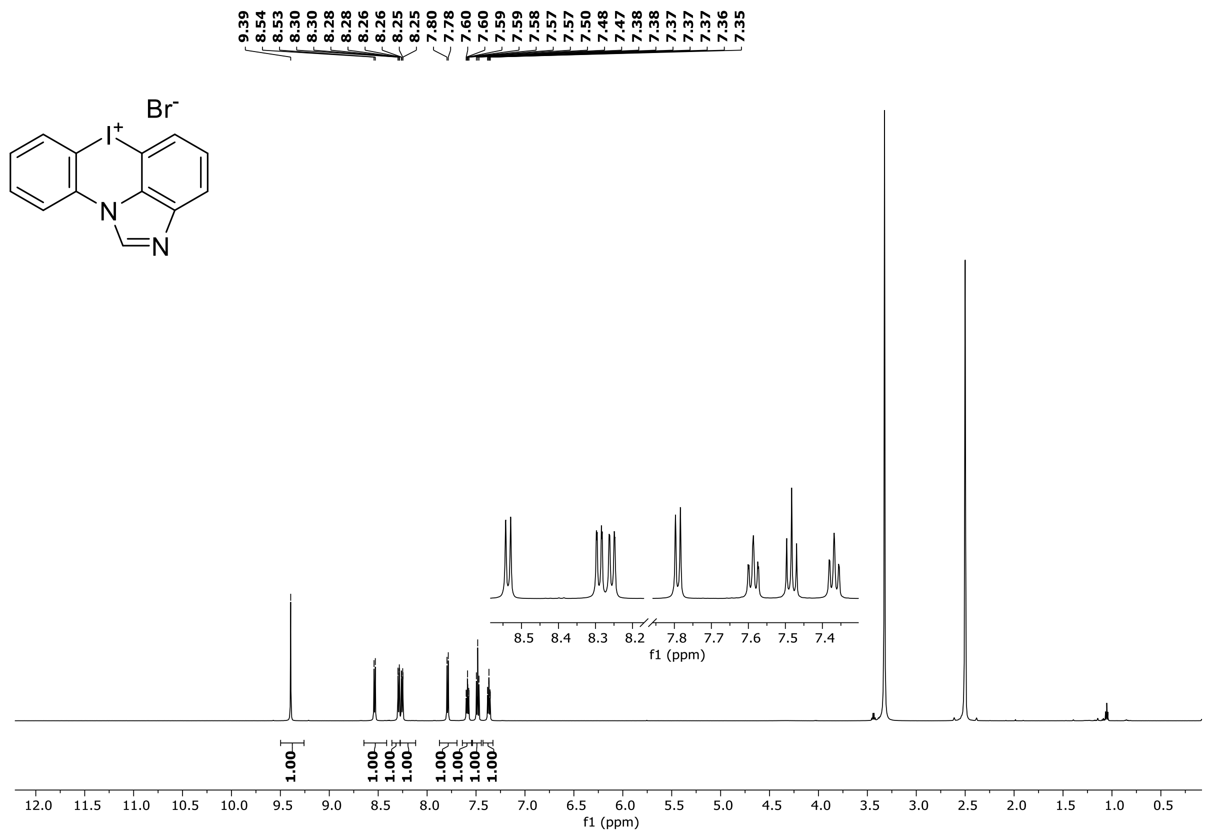

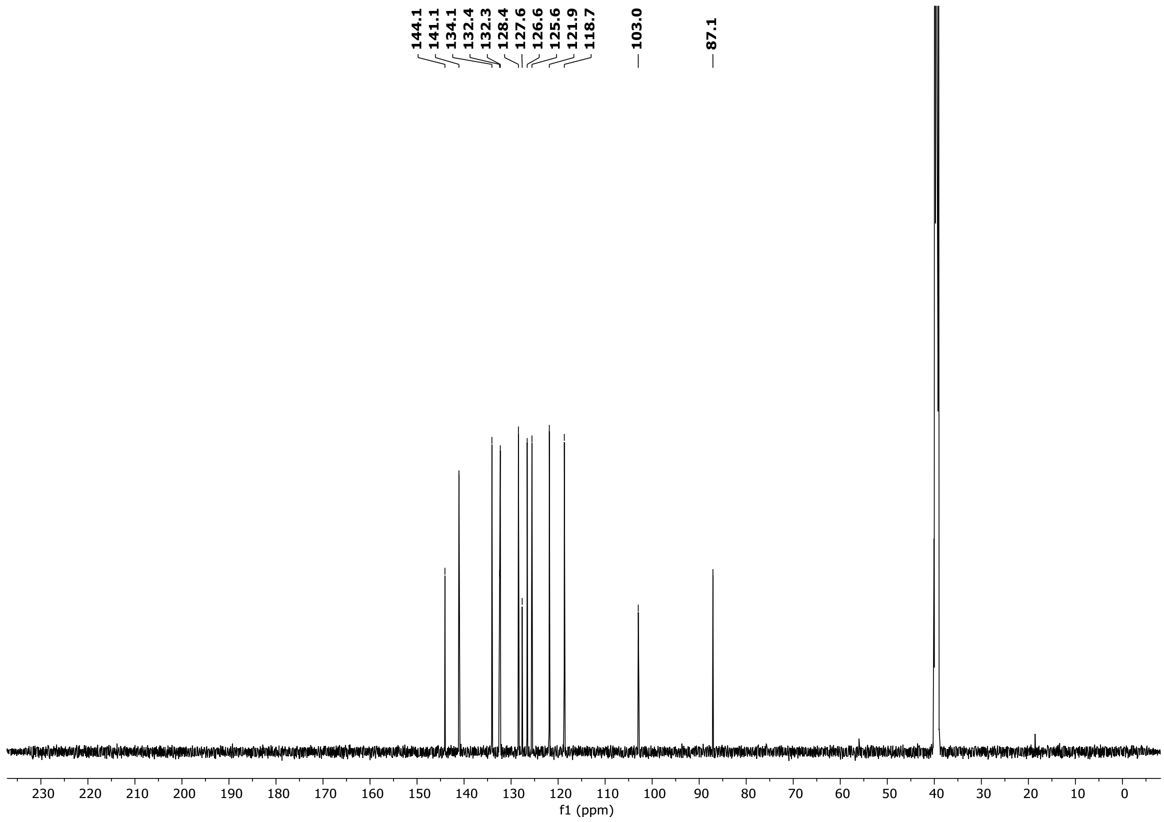

Figure S70: ^{1}H and ^{13}C NMR spectra of 6*H*-6λ^{3}-ioda-2,10b-diazaaceanthrylen-6-ium bromide (**10b**) in DMSO-d_6.

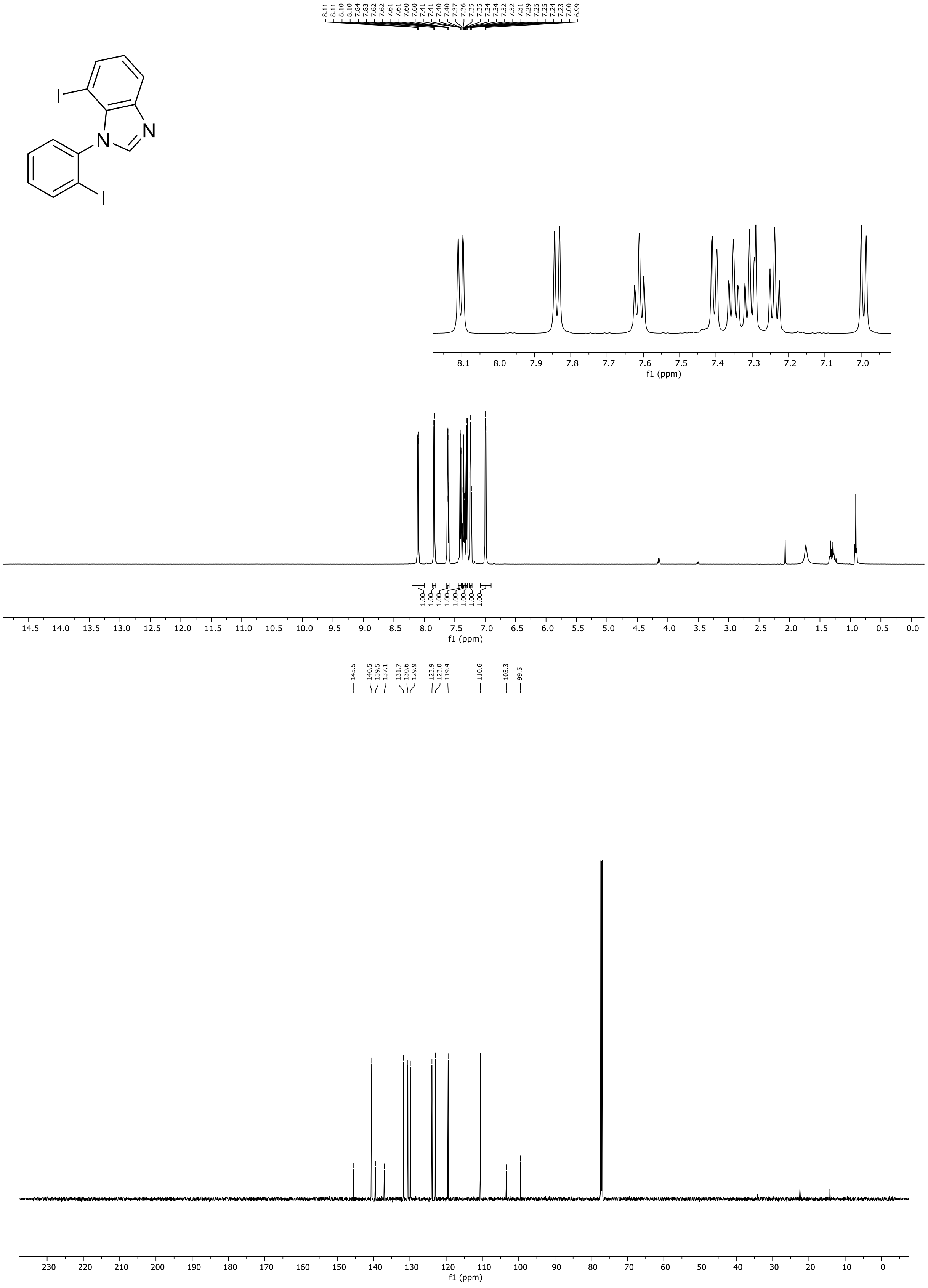

Figure S71: ^{1}H and ^{13}C NMR spectra of 7-iodo-1-(2-iodphenyl)-1*H*-benzo[*d*]imidazole (**11**) in $CDCl_3$.

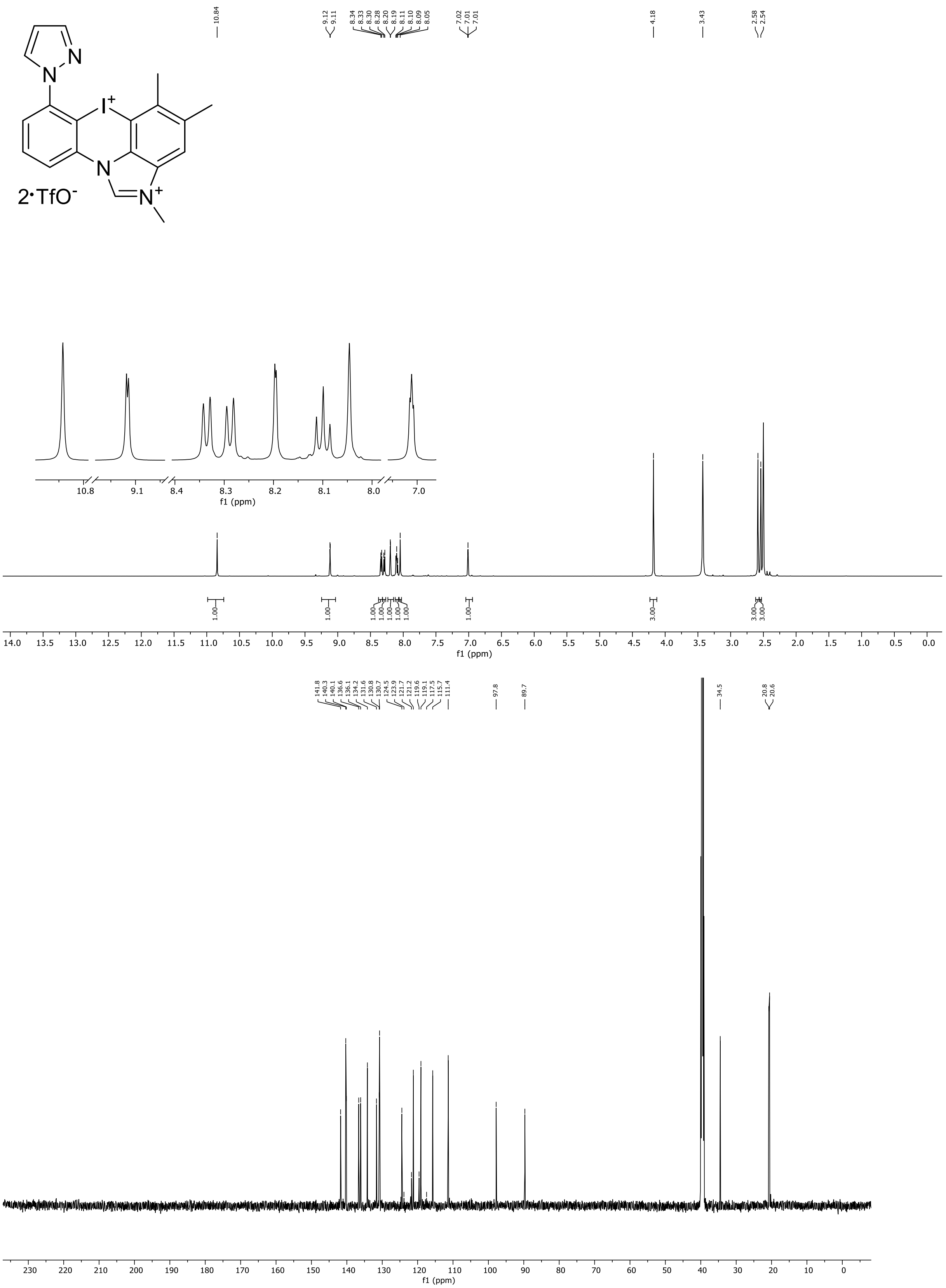

Figure S72: ^{1}H and ^{13}C NMR spectra of 2,4,5-trimethyl-7-(1*H*-pyrazol-1-yl)-6*H*-6λ^{3}-ioda-2,10b-diazaaceanthrylen-2,6-diium bistriflate (**12**) in $CDCl_3$.

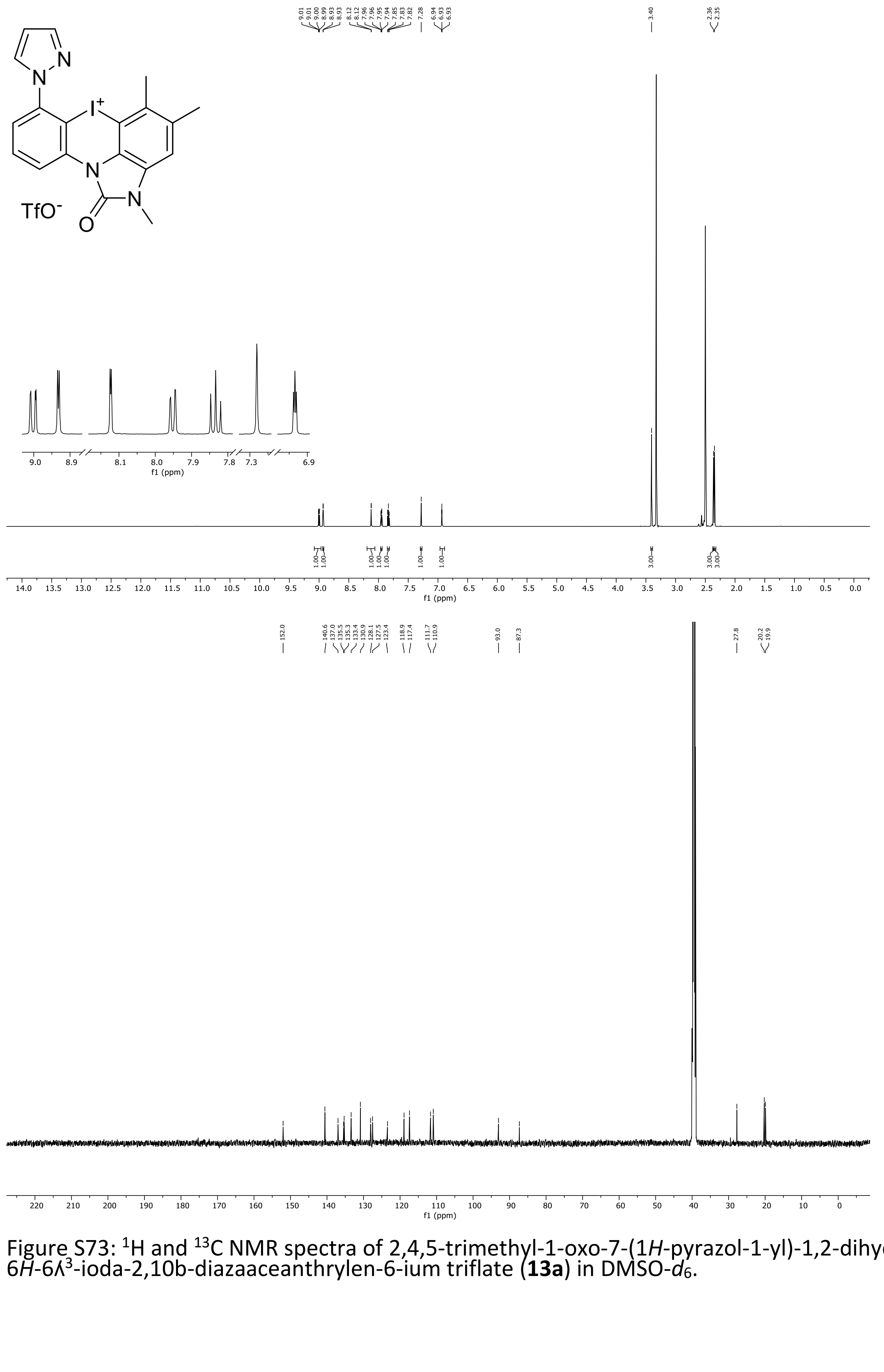

Figure S73: ^{1}H and ^{13}C NMR spectra of 2,4,5-trimethyl-1-oxo-7-(1*H*-pyrazol-1-yl)-1,2-dihydro-6*H*-6λ^{3}-ioda-2,10b-diazaaceanthrylen-6-ium triflate (**13a**) in DMSO-d_6.

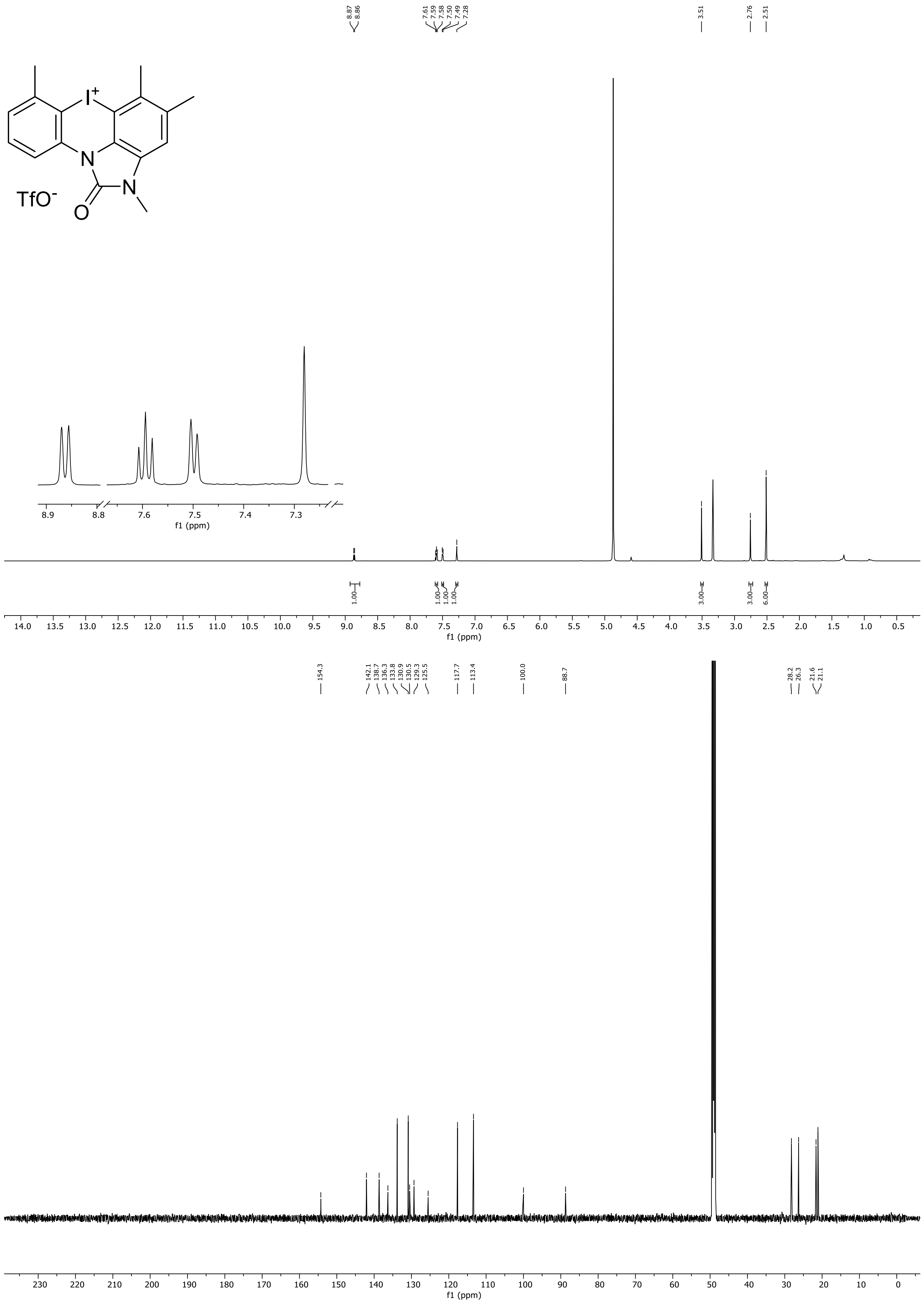

Figure S74: ^{1}H and ^{13}C NMR spectra of 2,4,5,7-tetramethyl-1-oxo-1,2-dihydro-6*H*-6λ^{3}-ioda-2,10b-diazaaceanthrylen-6-ium triflate (**13b**) in CD_3OD.

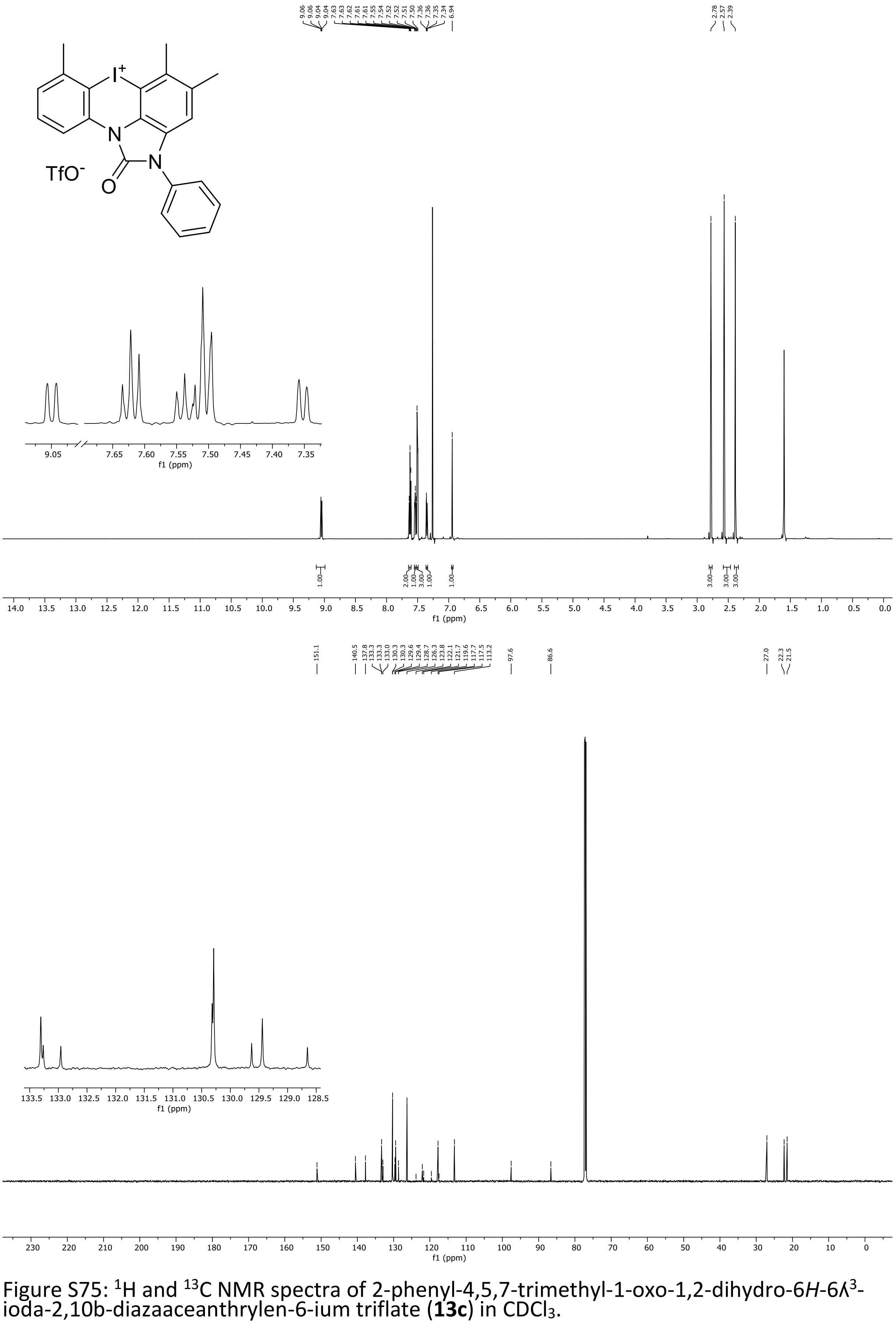

Figure S75: ^{1}H and ^{13}C NMR spectra of 2-phenyl-4,5,7-trimethyl-1-oxo-1,2-dihydro-6*H*-6λ^{3}-ioda-2,10b-diazaaceanthrylen-6-ium triflate (**13c**) in $CDCl_3$.

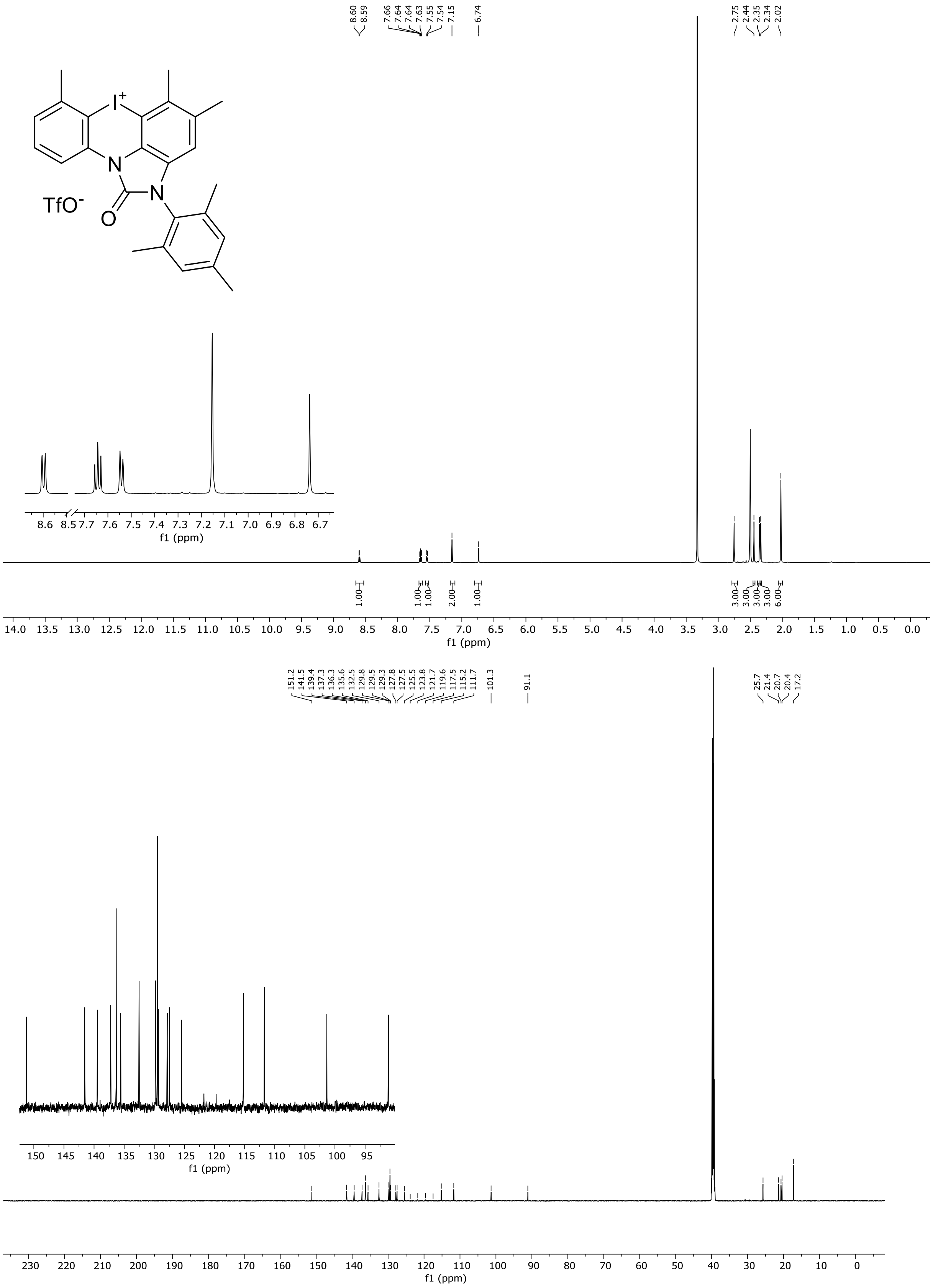

Figure S76: ^{1}H and ^{13}C NMR spectra of 2-mesityl-4,5,7-trimethyl-1-oxo-1,2-dihydro-6*H*-6λ^{3}-ioda-2,10b-diazaaceanthrylen-6-ium triflate (**13d**) in $CDCl_3$.

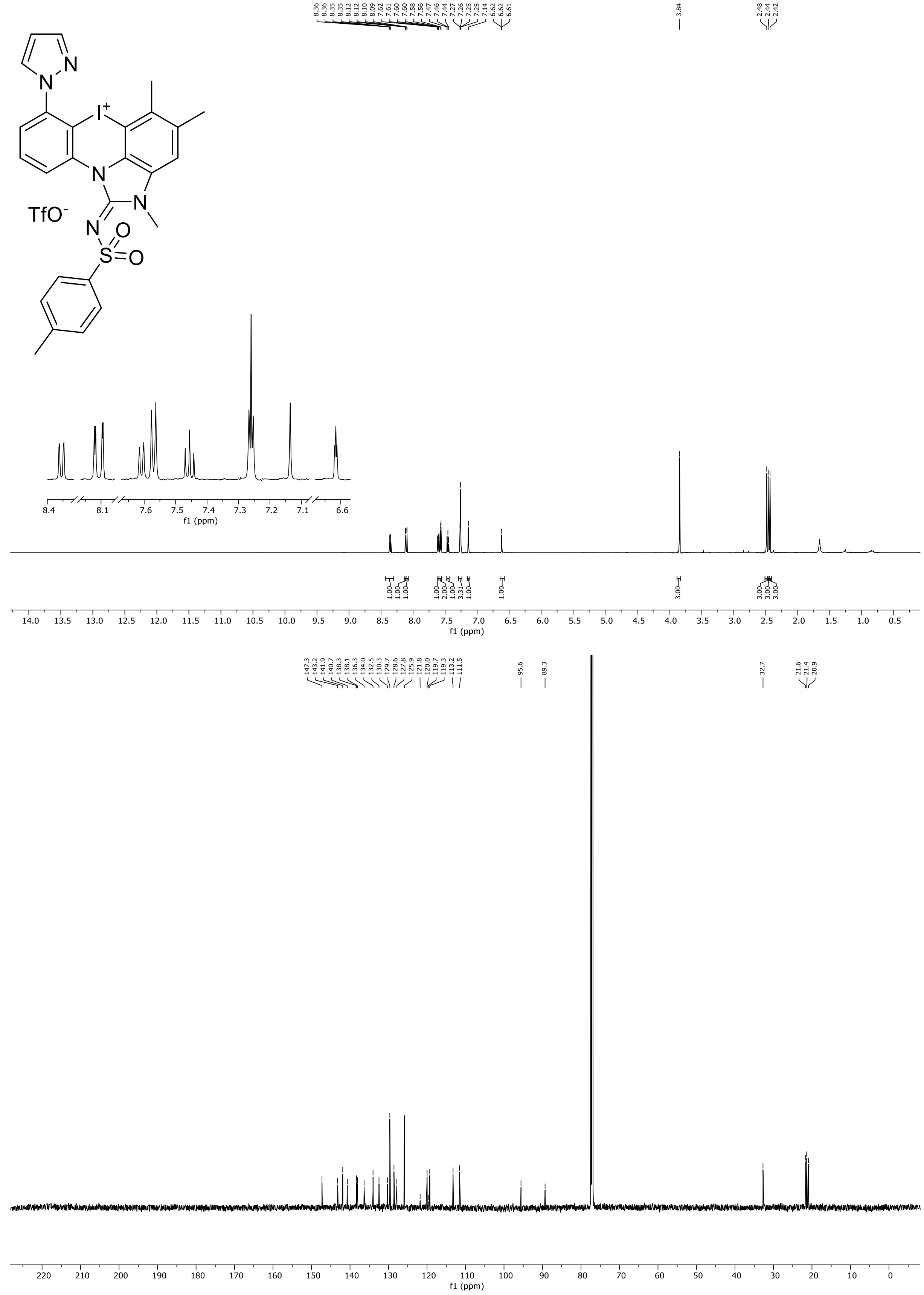

Figure S77: ^{1}H and ^{13}C NMR spectra of 2,4,5-trimethyl-7-(1*H*-pyrazol-1-yl)-1-(tosylimino)-1,2-dihydro-6*H*-6λ^{3}-ioda-2,10b-diazaaceanthrylen-6-yl triflate (**14a**) in $CDCl_3$.

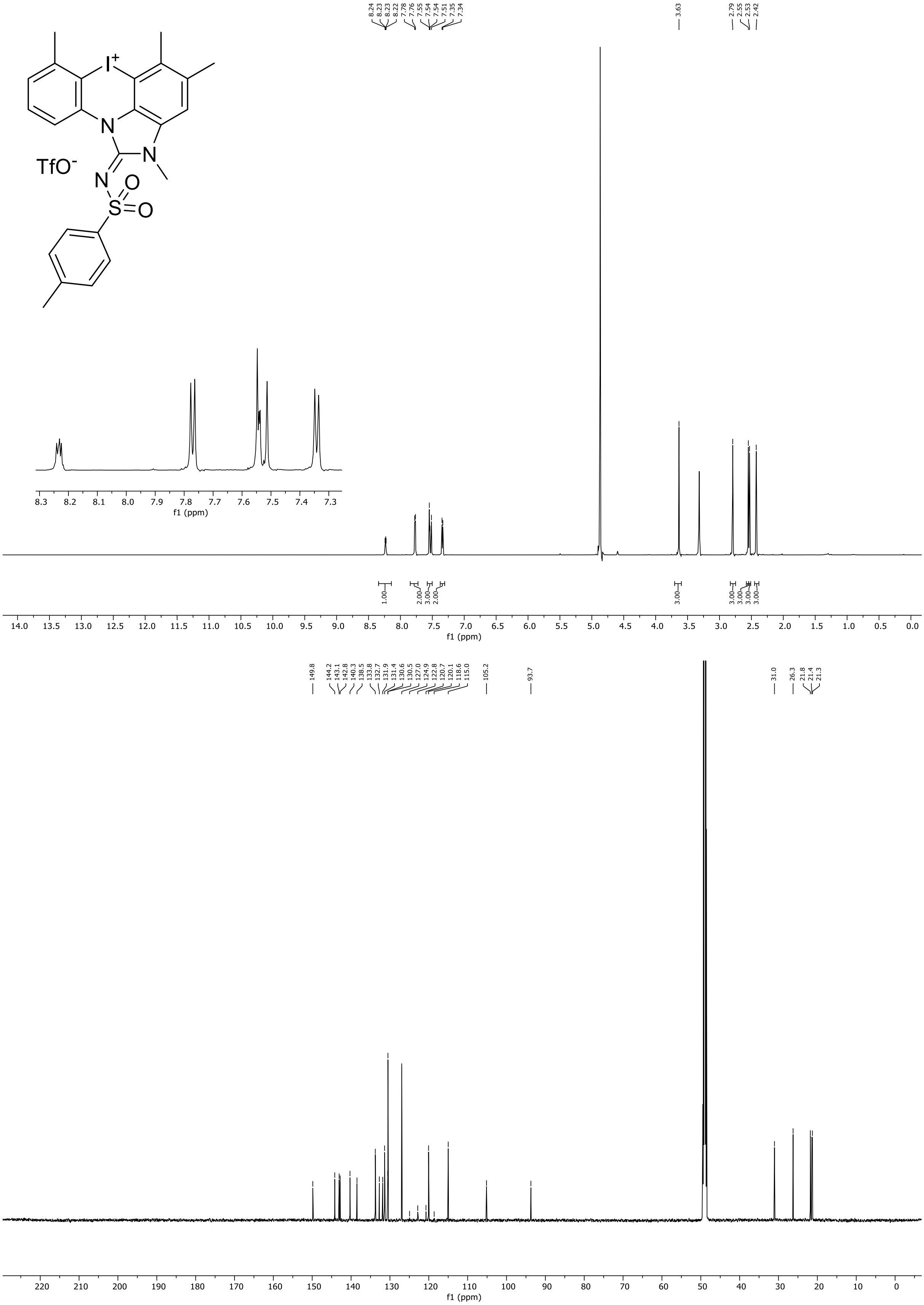

Figure S78: ^{1}H and ^{13}C NMR spectra of 2,4,5,7-tetramethyl-1-(tosylimino)-1,2-dihydro-6*H*-6λ^{3}-ioda-2,10b-diazaaceanthrylen-6-yl triflate (**14b**) in $CDCl_3$.

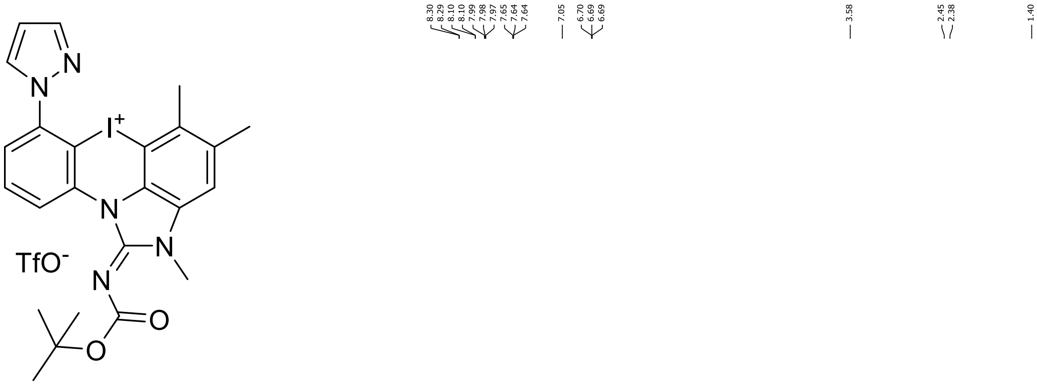

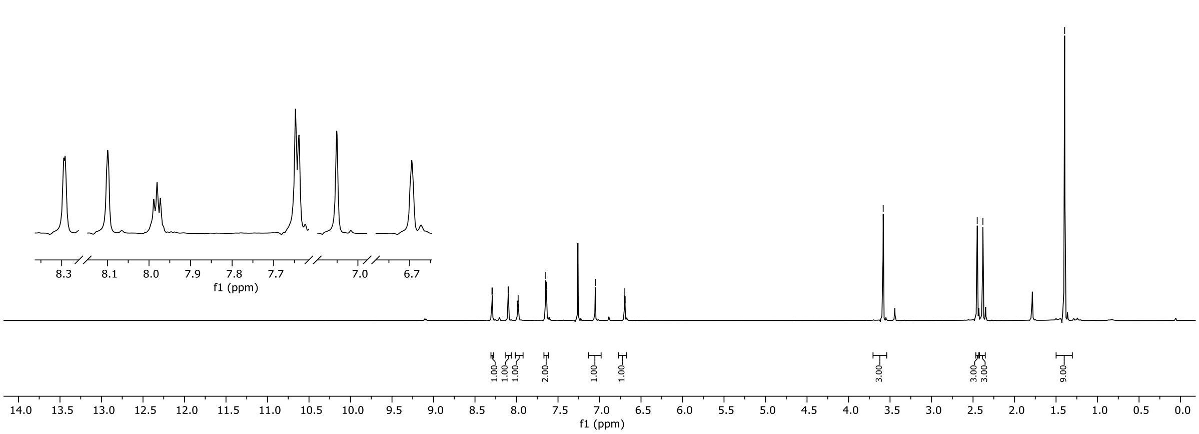

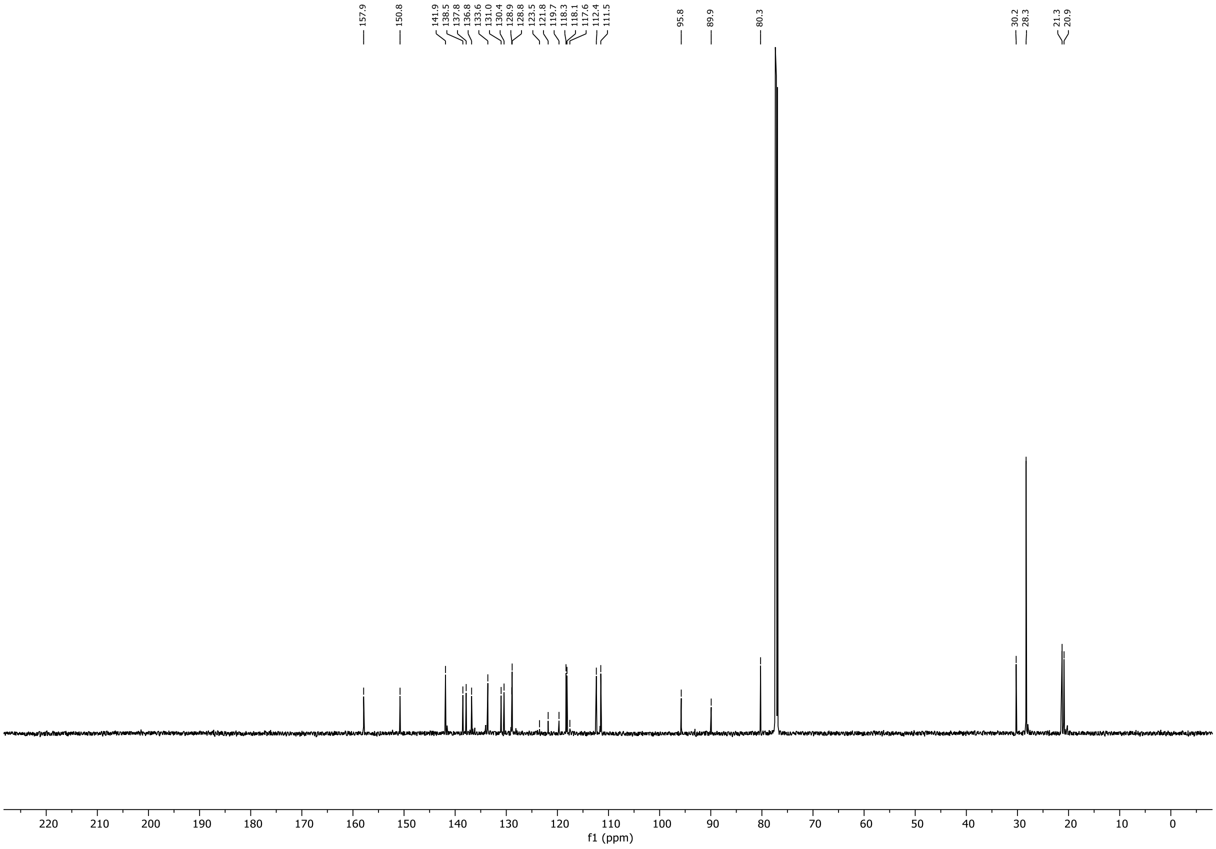

Figure S79: ^{1}H and ^{13}C NMR spectra of 2,4,5-trimethyl-7-(1*H*-pyrazol-1-yl)-1-(tosylimino)-1,2-dihydro-6*H*-6λ^{3}-ioda-2,10b-diazaaceanthrylen-6-yl triflate (**15**) in CDCl$_3$.

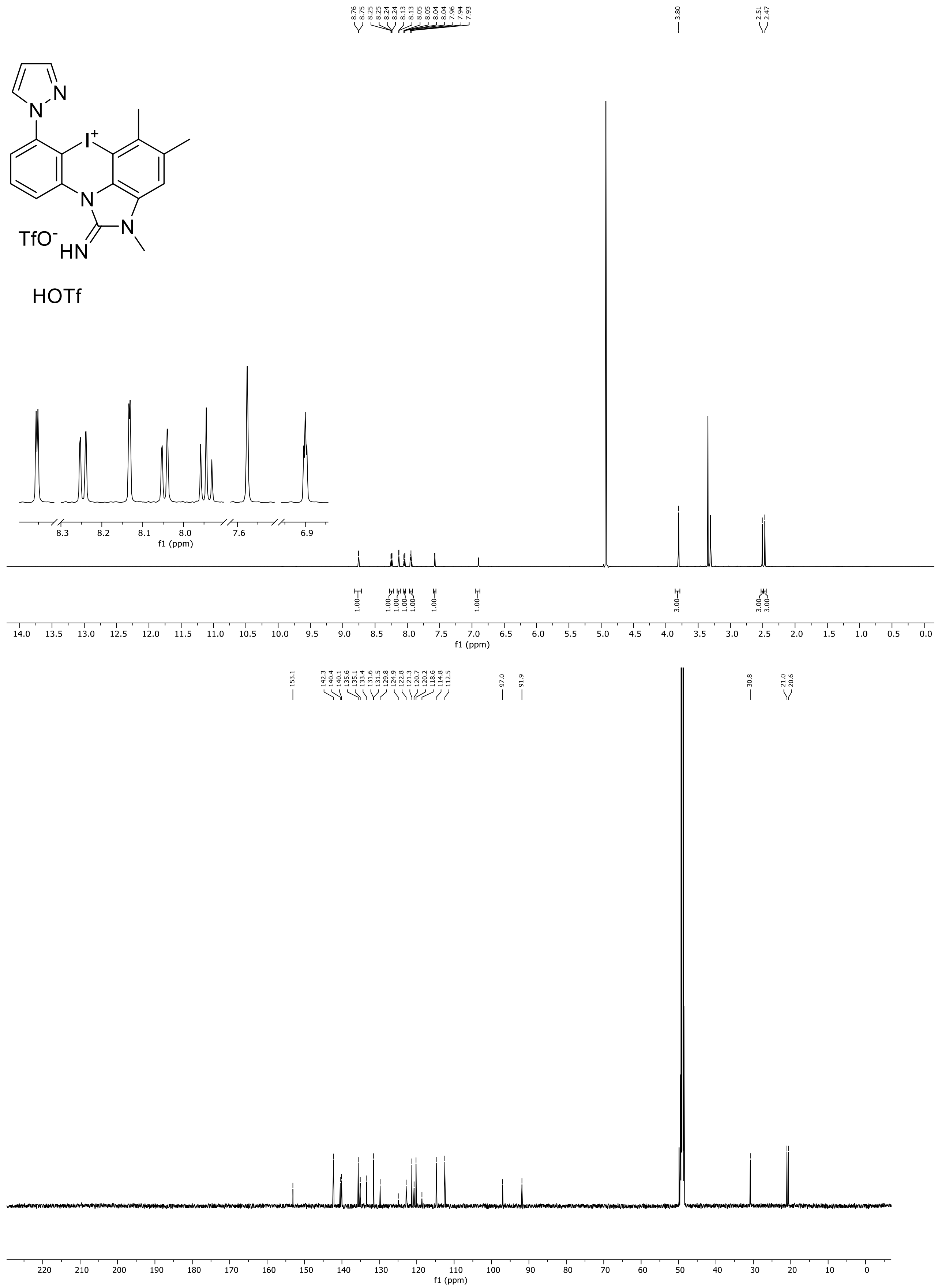

Figure S80: ^{1}H and ^{13}C NMR spectra of 2,4,5-trimethyl-7-(1*H*-pyrazol-1-yl)-1-(tosylimino)-1,2-dihydro-6*H*-6λ^{3}-ioda-2,10b-diazaaceanthrylen-6-yl triflate * HOTf (**16**) in CD_3OD.

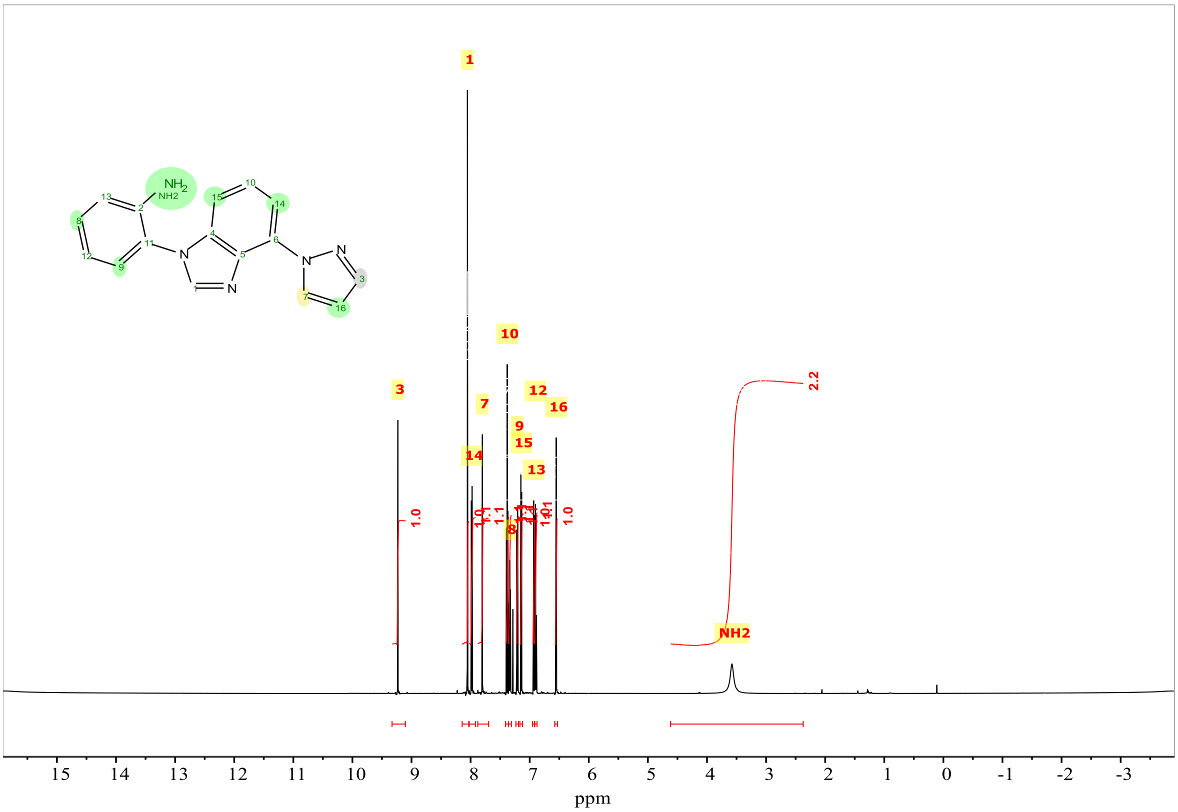
1
NH2
10
3
7
12
16
9
15
14
13
8
2.2
1.0
NH2
15 14 13 12 11 10 9 8 7 6 5 4 3 2 1 0 -1 -2 -3
ppm

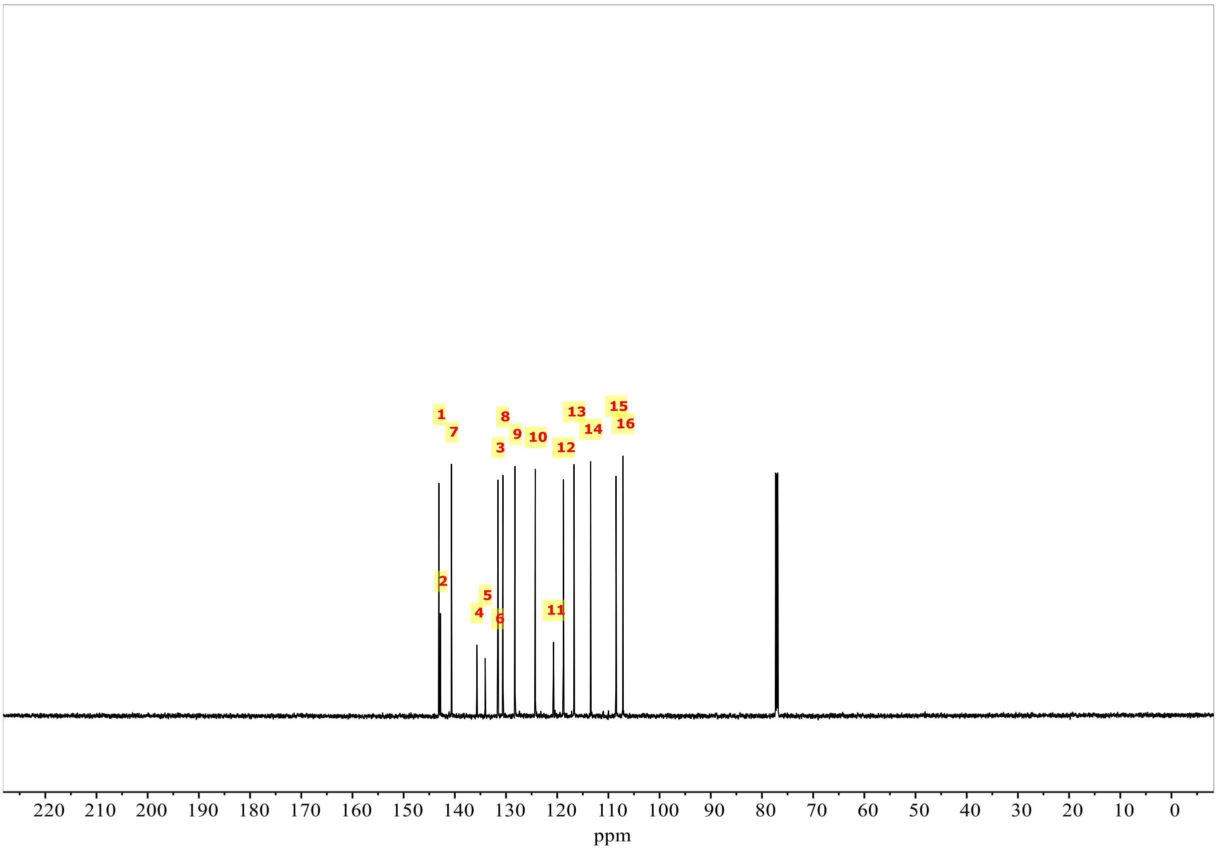
1
7
8
13
15
14
16
9
10
3
12
2
5
4
6
11
220 210 200 190 180 170 160 150 140 130 120 110 100 90 80 70 60 50 40 30 20 10 0
ppm

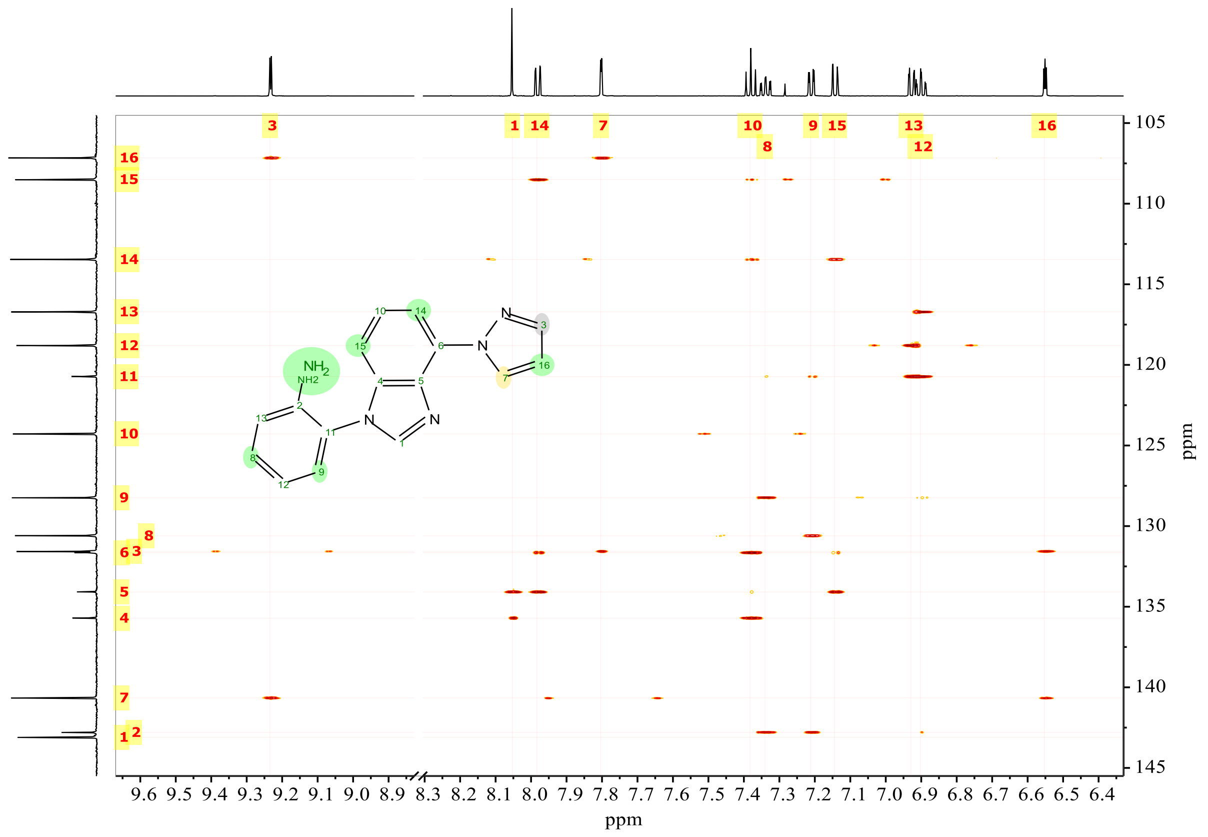

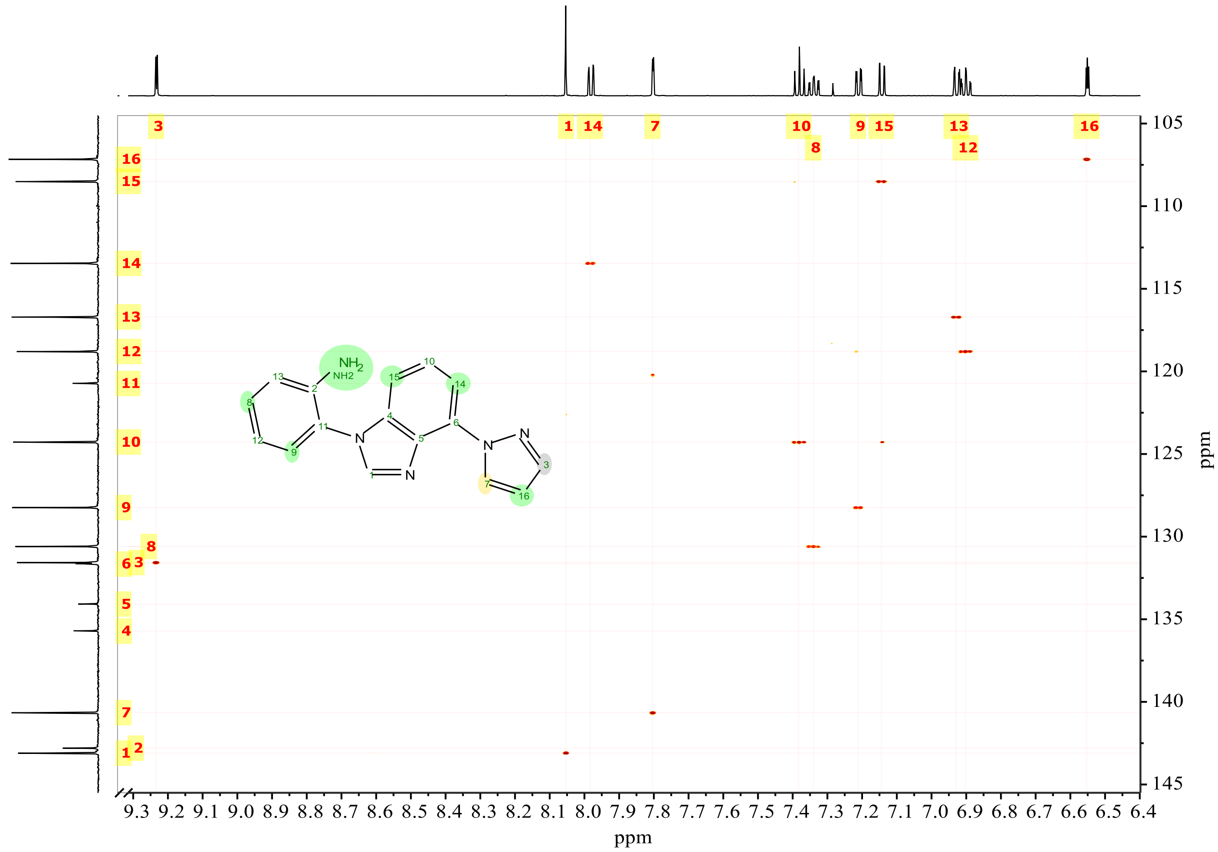

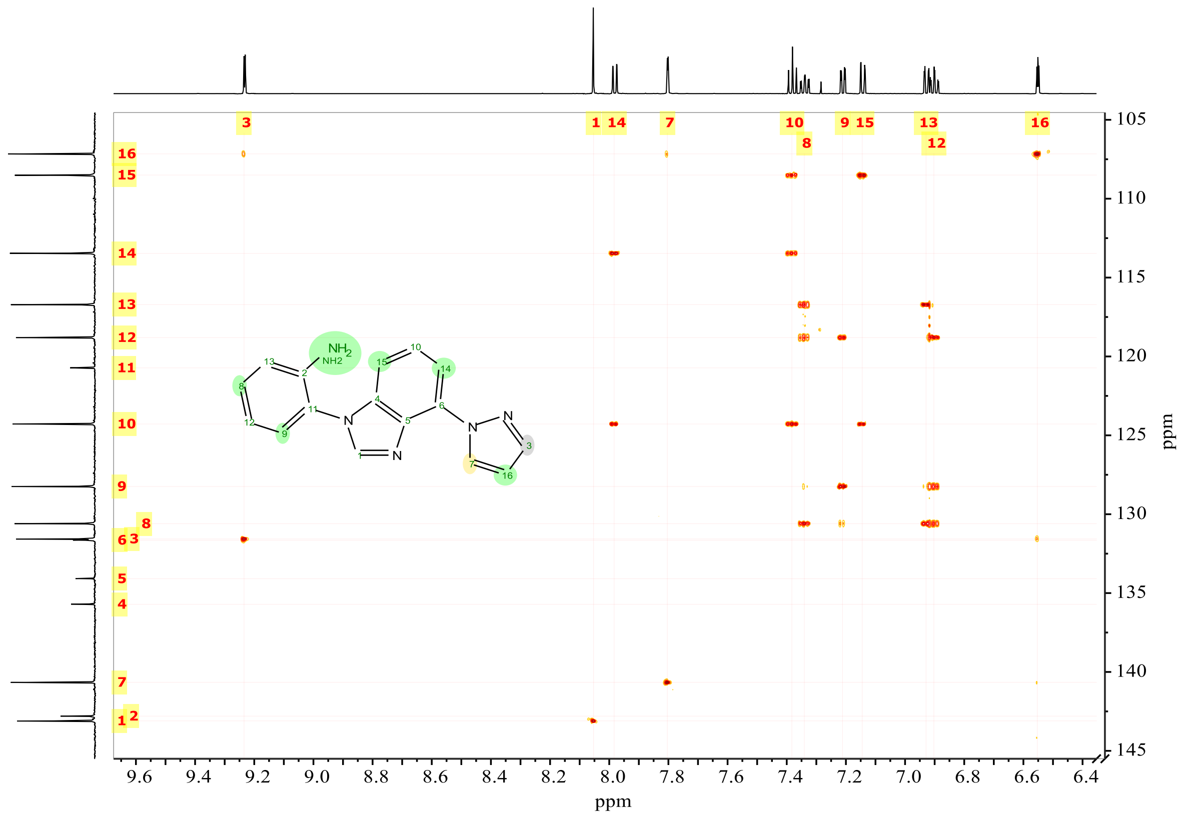

Figure S81: 2D NMR measurements for **S4aq1** to confirm the rearrangement reaction during reduction of the nitroarene **S4ba1**.

Crystal structures

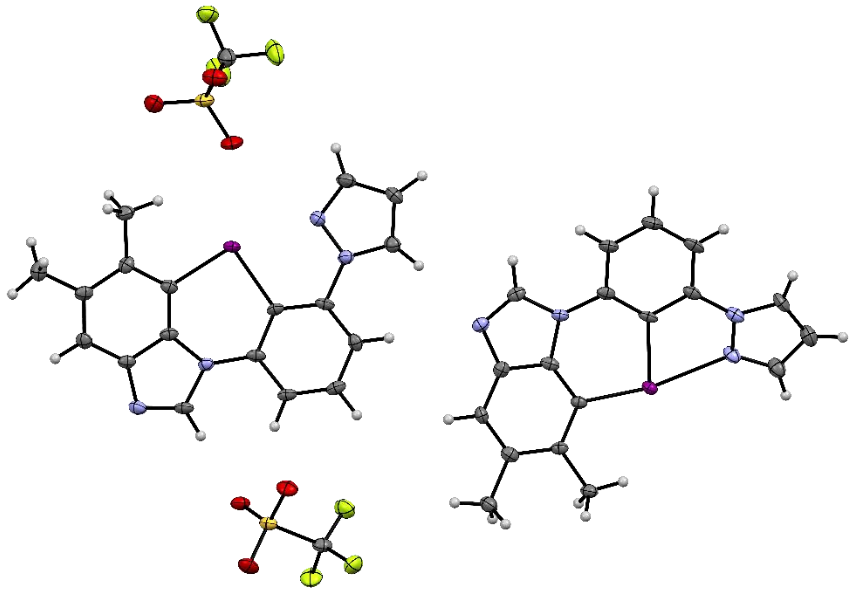

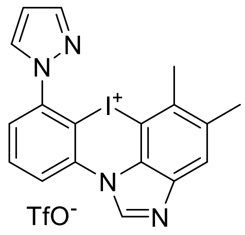

Table S1: Crystal data and structure refinement for **5bb**.	
Empirical formula	$C_{19}H_{14}F_3IN_4O_3S$
Formula weight	562.30
Temperature/K	100.00
Crystal system	triclinic
Space group	P-1
a/Å	7.8894(5)
b/Å	14.9151(9)
c/Å	18.1102(12)
α/°	107.447(4)
β/°	101.074(4)
γ/°	99.317(4)
Volume/Å^3	1939.6(2)
Z	4
ρ_{calc}g/cm^3	1.926
μ/mm^{-1}	1.819
F(000)	1104.0
Crystal size/mm^3	0.496 × 0.081 × 0.041
Radiation	MoKα (λ = 0.71073)
2Θ range for data collection/°	4.418 to 56.648
Index ranges	-10 ≤ h ≤ 10, -19 ≤ k ≤ 19, -24 ≤ l ≤ 24
Reflections collected	54586
Independent reflections	9679 [R_{int} = 0.0637, R_{sigma} = 0.0449]
Data/restraints/parameters	9679/0/563
Goodness-of-fit on F^2	1.051
Final R indexes [I>=2σ (I)]	R_1 = 0.0436, wR_2 = 0.0865
Largest diff. peak/hole / e Å^{-3}	5.09/-1.22

Table S2 Fractional Atomic Coordinates (×10[4]) and Equivalent Isotropic Displacement Parameters (Å[2]×10[3]) for **5bb**. U_{eq} is defined as 1/3 of the trace of the orthogonalised U_{IJ} tensor.

**Atom**	***x***	***y***	***z***	**U(eq)**
I_{001}	4280.3(4)	4952.2(2)	7813.5(2)	17.00(7)
I_{002}	7254.7(4)	7412.3(2)	6811.0(2)	17.32(7)
S_1	8957.4(15)	9229.3(8)	11805.5(7)	19.5(2)
S_2	2633.8(18)	3029.0(9)	5448.2(7)	27.4(3)
F_1	8708(4)	9216(2)	13216.2(17)	36.2(7)
F_3	8951(4)	7866(2)	12437.3(18)	35.4(7)
F_2	11237(4)	9057(2)	12990.9(18)	35.1(7)
F_5	656(5)	1348(2)	4479.2(19)	39.6(8)
O_2	7038(4)	8899(2)	11537.1(19)	24.5(7)
O_1	9592(5)	10259(2)	12145(2)	29.2(8)
F_4	2481(5)	1288(2)	5500(2)	46.1(9)
O_3	9894(5)	8725(2)	11264(2)	28.2(8)
N_5	8864(5)	7404(2)	8692(2)	17.6(7)
F_6	128(5)	1801(3)	5643(2)	49.4(9)
O_4	3851(5)	2887(3)	4956(2)	35.9(9)
N_6	10047(5)	6371(3)	9205(2)	20.4(8)
O_6	1292(5)	3502(2)	5193(2)	35.3(9)
N_4	5441(5)	3570(3)	8107(2)	21.6(8)
N_7	5841(5)	9305(3)	7303(2)	22.0(8)
O_5	3417(6)	3371(3)	6298(2)	43.3(10)
N_3	6638(5)	3953(3)	8820(2)	20.2(8)
N_8	6223(5)	8838(3)	6615(2)	22.6(8)
N_2	3574(5)	8324(3)	9494(2)	21.7(8)
N_1	4619(5)	6977(2)	9367(2)	17.1(7)
C_1	3719(6)	6294(3)	7858(3)	19.9(9)
C_{24}	8668(6)	6563(3)	8055(3)	17.3(9)
C_{33}	7312(6)	8332(3)	7972(3)	17.5(9)
C_5	3286(6)	7814(3)	8685(3)	17.3(9)
C_{15}	5673(6)	5467(3)	9052(2)	17.5(9)
C_{22}	9305(6)	5004(3)	7873(3)	18.6(9)
C_{19}	7965(6)	6319(3)	7240(3)	16.3(8)
C_{20}	7899(6)	5403(3)	6727(3)	18.0(9)
C_2	2988(6)	6469(3)	7167(3)	21.2(9)
C_{23}	9375(6)	5926(3)	8376(3)	18.8(9)
C_{28}	8096(6)	8197(3)	8676(3)	19.0(9)
C_{27}	9714(6)	7232(3)	9362(3)	20.1(9)
C_{21}	8565(6)	4731(3)	7057(3)	20.3(9)
C_6	3896(6)	6963(3)	8597(3)	19.3(9)
C_{13}	7619(6)	5181(3)	10114(3)	20.3(9)
C_4	2556(6)	8002(3)	8001(3)	21.1(9)
C_{11}	6671(6)	6665(3)	10379(3)	19.7(9)
C_{14}	6642(6)	4890(3)	9334(3)	18.9(9)

Table S2 Fractional Atomic Coordinates (×10[4]) and Equivalent Isotropic Displacement Parameters (Å[2]×10[3]) for **5bb**. U_{eq} is defined as 1/3 of the trace of the orthogonalised U_{IJ} tensor.

Atom	*x*	*y*	*z*	U(eq)
C_{32}	6577(6)	9130(3)	8005(3)	19.5(9)
C_{10}	5642(6)	6363(3)	9592(3)	18.4(9)
C_{12}	7639(6)	6077(3)	10631(3)	21.5(9)
C_3	2452(6)	7357(3)	7248(3)	20.8(9)
C_{17}	7084(7)	2516(3)	8278(3)	23.8(10)
C_{34}	4763(6)	9903(3)	7187(3)	24.5(10)
C_{16}	7636(7)	3332(3)	8940(3)	22.9(10)
C_8	1774(7)	7582(4)	6511(3)	26.1(10)
C_{25}	7170(6)	5109(3)	5836(3)	22.4(10)
C_7	2734(7)	5745(3)	6357(3)	26.6(10)
C_{29}	8095(6)	8852(3)	9409(3)	22.2(10)
C_{26}	8447(7)	3725(3)	6511(3)	25.6(10)
C_{36}	5363(7)	9129(4)	6061(3)	28.4(11)
C_{31}	6593(7)	9780(3)	8748(3)	25.2(10)
C_{30}	7340(6)	9634(3)	9439(3)	25.5(10)
C_9	4371(6)	7829(3)	9870(3)	21.3(9)
C_{35}	4444(7)	9801(4)	6398(3)	29.4(11)
C_{37}	9501(6)	8828(3)	12661(3)	23.7(10)
C_{18}	5694(7)	2700(3)	7763(3)	28.1(11)
C_{38}	1370(8)	1800(4)	5252(3)	31.8(12)

Table S3 Anisotropic Displacement Parameters (Å[2]×10[3]) for **5bb**. The Anisotropic displacement factor exponent takes the form: $-2\pi^2[h^2a^{*2}U_{11}+2hka^*b^*U_{12}+\ldots]$.

Atom	U_{11}	U_{22}	U_{33}	U_{23}	U_{13}	U_{12}
I_{001}	18.66(15)	12.96(13)	14.76(14)	-0.57(10)	4.48(11)	0.96(10)
I_{002}	19.79(15)	14.55(13)	17.98(14)	4.01(10)	7.60(11)	4.62(10)
S_1	23.9(6)	15.0(5)	16.9(5)	2.2(4)	6.8(4)	1.3(4)
S_2	31.7(7)	24.3(6)	18.4(6)	-0.9(4)	3.1(5)	4.4(5)
F_1	46.3(19)	45.9(18)	21.4(15)	9.3(13)	16.6(14)	19.0(15)
F_3	49(2)	21.2(14)	34.8(17)	11.0(13)	9.6(15)	4.6(13)
F_2	29.8(17)	36.5(17)	30.1(16)	4.5(13)	-2.3(13)	8.8(13)
F_5	50(2)	23.3(15)	34.2(17)	5.2(13)	-0.4(15)	-0.9(14)
O_2	23.3(17)	21.4(16)	19.8(16)	-2.1(13)	2.9(14)	1.5(13)
O_1	32(2)	14.6(15)	33.3(19)	3.0(14)	6.1(16)	-1.3(14)
F_4	66(2)	45(2)	47(2)	28.7(17)	22.6(18)	31.6(18)
O_3	35(2)	28.3(18)	22.5(17)	4.4(14)	15.0(15)	8.1(15)
N_5	19.4(19)	12.8(16)	16.5(18)	0.6(14)	5.1(15)	0.1(14)
F_6	52(2)	51(2)	64(2)	30.9(19)	34(2)	18.3(18)
O_4	43(2)	28.1(19)	31(2)	2.8(16)	13.7(17)	1.4(16)
N_6	17.9(19)	21.3(18)	20.0(19)	6.6(15)	4.0(15)	1.2(15)

Table S3 Anisotropic Displacement Parameters (Å^2×10^3) for **5bb**. The Anisotropic displacement factor exponent takes the form: $-2\pi^2[h^2a^{*2}U_{11}+2hka^*b^*U_{12}+\ldots]$.

Atom	U_{11}	U_{22}	U_{33}	U_{23}	U_{13}	U_{12}
O_6	42(2)	23.2(17)	34(2)	7.3(15)	0.7(17)	5.6(16)
N_4	25(2)	17.2(18)	20.2(19)	5.3(15)	2.9(16)	4.9(15)
N_7	26(2)	15.1(17)	26(2)	5.2(15)	13.8(17)	3.8(15)
O_5	61(3)	39(2)	17.1(18)	-5.2(16)	-0.9(18)	18(2)
N_3	23(2)	17.3(17)	17.2(18)	4.1(14)	2.3(15)	1.9(15)
N_8	28(2)	21.0(19)	24(2)	7.6(16)	14.4(17)	10.7(16)
N_2	27(2)	14.1(17)	21.4(19)	3.6(15)	6.6(16)	1.9(15)
N_1	18.3(19)	14.1(16)	15.8(18)	3.8(14)	2.5(15)	-0.3(14)
C_1	20(2)	16(2)	21(2)	3.8(17)	6.5(18)	2.1(17)
C_{24}	15(2)	13.8(19)	21(2)	1.1(16)	10.5(18)	-0.4(15)
C_{33}	21(2)	9.2(17)	20(2)	-0.2(15)	11.5(18)	-1.0(15)
C_5	14(2)	15.9(19)	18(2)	2.6(16)	5.1(17)	-1.3(16)
C_{15}	17(2)	15.3(19)	14(2)	1.1(16)	1.2(17)	-2.0(16)
C_{22}	18(2)	17(2)	24(2)	10.2(17)	6.7(18)	7.1(16)
C_{19}	17(2)	13.5(18)	17(2)	3.1(16)	6.0(17)	0.5(15)
C_{20}	19(2)	15.7(19)	16(2)	2.7(16)	5.3(17)	-0.4(16)
C_2	20(2)	25(2)	20(2)	10.2(18)	6.1(18)	4.6(18)
C_{23}	15(2)	20(2)	21(2)	8.1(17)	4.9(17)	1.5(16)
C_{28}	15(2)	14.6(19)	22(2)	1.3(17)	6.6(18)	-2.0(16)
C_{27}	18(2)	24(2)	16(2)	5.7(17)	5.9(17)	0.4(17)
C_{21}	19(2)	17(2)	25(2)	5.6(18)	9.0(19)	2.4(17)
C_6	18(2)	18(2)	17(2)	2.0(17)	4.3(17)	-0.9(16)
C_{13}	21(2)	16(2)	27(2)	11.4(18)	7.7(19)	3.8(17)
C_4	20(2)	16(2)	26(2)	7.8(18)	4.7(19)	1.6(17)
C_{11}	22(2)	16.1(19)	15(2)	0.4(16)	4.6(18)	-3.6(17)
C_{14}	22(2)	12.0(18)	19(2)	0.4(16)	9.3(18)	-1.2(16)
C_{32}	22(2)	12.3(19)	24(2)	3.6(17)	9.9(19)	1.4(16)
C_{10}	18(2)	13.6(19)	19(2)	1.1(16)	6.6(18)	-4.0(16)
C_{12}	21(2)	23(2)	14(2)	3.3(17)	2.3(18)	-3.8(18)
C_3	15(2)	20(2)	22(2)	3.0(18)	4.3(18)	-1.1(17)
C_{17}	29(3)	16(2)	29(3)	7.0(18)	12(2)	8.2(18)
C_{34}	25(2)	17(2)	37(3)	9.9(19)	14(2)	11.2(18)
C_{16}	28(3)	20(2)	21(2)	7.5(18)	6.6(19)	4.9(18)
C_8	23(3)	28(2)	29(3)	14(2)	6(2)	7(2)
C_{25}	27(2)	17(2)	19(2)	1.6(17)	5.4(19)	4.2(18)
C_7	34(3)	24(2)	17(2)	3.8(18)	4(2)	4(2)
C_{29}	25(2)	15(2)	20(2)	-1.2(17)	5.7(19)	0.0(17)
C_{26}	33(3)	19(2)	25(2)	6.0(19)	9(2)	7.6(19)
C_{36}	36(3)	26(2)	31(3)	14(2)	16(2)	12(2)
C_{31}	29(3)	10.7(19)	33(3)	1.5(18)	13(2)	1.0(17)
C_{30}	27(3)	17(2)	25(2)	-3.3(18)	9(2)	1.7(18)
C_9	26(2)	14.3(19)	17(2)	-1.0(16)	5.2(18)	-2.6(17)

Table S3 Anisotropic Displacement Parameters ($Å^2×10^3$) for **5bb**. The Anisotropic displacement factor exponent takes the form: $-2\pi^2[h^2a^{*2}U_{11}+2hka^*b^*U_{12}+…]$.

Atom	U_{11}	U_{22}	U_{33}	U_{23}	U_{13}	U_{12}
C_{35}	33(3)	25(2)	38(3)	16(2)	13(2)	13(2)
C_{37}	25(3)	22(2)	21(2)	1.3(18)	9(2)	4.2(19)
C_{18}	36(3)	18(2)	24(2)	0.6(18)	5(2)	3(2)
C_{38}	45(3)	27(3)	26(3)	10(2)	9(2)	12(2)

Table S4 Bond Lengths for **5bb**.

Atom	Atom	Length/Å	Atom	Atom	Length/Å
I_{001}	C_1	2.100(4)	N_1	C_{10}	1.413(6)
I_{001}	C_{15}	2.139(4)	N_1	C_9	1.392(6)
I_{002}	N_8	2.492(4)	C_1	C_2	1.390(6)
I_{002}	C_{33}	2.125(4)	C_1	C_6	1.374(6)
I_{002}	C_{19}	2.113(4)	C_{24}	C_{19}	1.384(6)
S_1	O_2	1.451(3)	C_{24}	C_{23}	1.389(6)
S_1	O_1	1.433(3)	C_{33}	C_{28}	1.393(6)
S_1	O_3	1.446(3)	C_{33}	C_{32}	1.397(6)
S_1	C_{37}	1.824(5)	C_5	C_6	1.404(6)
S_2	O_4	1.429(4)	C_5	C_4	1.391(6)
S_2	O_6	1.442(4)	C_{15}	C_{14}	1.384(6)
S_2	O_5	1.440(4)	C_{15}	C_{10}	1.406(6)
S_2	C_{38}	1.833(5)	C_{22}	C_{23}	1.388(6)
F_1	C_{37}	1.321(5)	C_{22}	C_{21}	1.387(6)
F_3	C_{37}	1.340(5)	C_{19}	C_{20}	1.389(6)
F_2	C_{37}	1.327(6)	C_{20}	C_{21}	1.430(6)
F_5	C_{38}	1.319(6)	C_{20}	C_{25}	1.505(6)
F_4	C_{38}	1.350(6)	C_2	C_3	1.430(6)
N_5	C_{24}	1.389(5)	C_2	C_7	1.492(6)
N_5	C_{28}	1.419(6)	C_{28}	C_{29}	1.392(6)
N_5	C_{27}	1.385(6)	C_{21}	C_{26}	1.505(6)
F_6	C_{38}	1.315(6)	C_{13}	C_{14}	1.373(6)
N_6	C_{23}	1.400(6)	C_{13}	C_{12}	1.378(6)
N_6	C_{27}	1.311(6)	C_4	C_3	1.391(6)
N_4	N_3	1.342(5)	C_{11}	C_{10}	1.396(6)
N_4	C_{18}	1.326(6)	C_{11}	C_{12}	1.373(7)
N_7	N_8	1.349(5)	C_{32}	C_{31}	1.399(6)
N_7	C_{32}	1.410(6)	C_3	C_8	1.493(7)
N_7	C_{34}	1.360(6)	C_{17}	C_{16}	1.365(6)
N_3	C_{14}	1.427(5)	C_{17}	C_{18}	1.416(7)
N_3	C_{16}	1.347(6)	C_{34}	C_{35}	1.358(7)
N_8	C_{36}	1.324(6)	C_{29}	C_{30}	1.385(7)
N_2	C_5	1.387(6)	C_{36}	C_{35}	1.393(7)

Table S4 Bond Lengths for **5bb**.

Atom	Atom	Length/Å	Atom	Atom	Length/Å
N_2	C_9	1.300(6)	C_{31}	C_{30}	1.371(7)
N_1	C_6	1.394(6)			

Table S5 Bond Angles for **5bb**.

Atom	Atom	Atom	Angle/˚	Atom	Atom	Atom	Angle/˚
C_1	I_{001}	C_{15}	93.32(17)	C_{21}	C_{20}	C_{25}	119.5(4)
C_{33}	I_{002}	N_8	74.28(15)	C_1	C_2	C_3	118.5(4)
C_{19}	I_{002}	N_8	166.30(14)	C_1	C_2	C_7	121.3(4)
C_{19}	I_{002}	C_{33}	92.76(16)	C_3	C_2	C_7	120.2(4)
O_2	S_1	C_{37}	102.8(2)	C_{24}	C_{23}	N_6	109.6(4)
O_1	S_1	O_2	114.4(2)	C_{22}	C_{23}	N_6	131.0(4)
O_1	S_1	O_3	116.2(2)	C_{22}	C_{23}	C_{24}	119.4(4)
O_1	S_1	C_{37}	103.5(2)	C_{33}	C_{28}	N_5	123.4(4)
O_3	S_1	O_2	114.4(2)	C_{29}	C_{28}	N_5	117.3(4)
O_3	S_1	C_{37}	103.0(2)	C_{29}	C_{28}	C_{33}	119.2(4)
O_4	S_2	O_6	114.0(2)	N_6	C_{27}	N_5	113.7(4)
O_4	S_2	O_5	115.6(3)	C_{22}	C_{21}	C_{20}	120.2(4)
O_4	S_2	C_{38}	103.6(2)	C_{22}	C_{21}	C_{26}	120.4(4)
O_6	S_2	C_{38}	103.4(2)	C_{20}	C_{21}	C_{26}	119.4(4)
O_5	S_2	O_6	115.0(2)	N_1	C_6	C_5	106.6(4)
O_5	S_2	C_{38}	103.0(3)	C_1	C_6	N_1	131.3(4)
C_{24}	N_5	C_{28}	127.0(4)	C_1	C_6	C_5	122.1(4)
C_{27}	N_5	C_{24}	105.1(4)	C_{14}	C_{13}	C_{12}	119.0(4)
C_{27}	N_5	C_{28}	127.1(4)	C_5	C_4	C_3	120.1(4)
C_{27}	N_6	C_{23}	104.9(4)	C_{12}	C_{11}	C_{10}	120.3(4)
C_{18}	N_4	N_3	106.4(4)	C_{15}	C_{14}	N_3	120.7(4)
N_8	N_7	C_{32}	118.8(4)	C_{13}	C_{14}	N_3	117.3(4)
N_8	N_7	C_{34}	111.0(4)	C_{13}	C_{14}	C_{15}	122.0(4)
C_{34}	N_7	C_{32}	130.2(4)	C_{33}	C_{32}	N_7	121.3(4)
N_4	N_3	C_{14}	118.7(4)	C_{33}	C_{32}	C_{31}	119.7(4)
N_4	N_3	C_{16}	111.1(4)	C_{31}	C_{32}	N_7	119.0(4)
C_{16}	N_3	C_{14}	130.2(4)	C_{15}	C_{10}	N_1	122.6(4)
N_7	N_8	I_{002}	106.2(3)	C_{11}	C_{10}	N_1	118.3(4)
C_{36}	N_8	I_{002}	142.2(3)	C_{11}	C_{10}	C_{15}	119.1(4)
C_{36}	N_8	N_7	106.0(4)	C_{11}	C_{12}	C_{13}	120.9(4)
C_9	N_2	C_5	105.7(4)	C_2	C_3	C_8	119.0(4)
C_6	N_1	C_{10}	127.9(4)	C_4	C_3	C_2	120.5(4)
C_9	N_1	C_6	104.4(4)	C_4	C_3	C_8	120.5(4)
C_9	N_1	C_{10}	126.8(4)	C_{16}	C_{17}	C_{18}	104.7(4)
C_2	C_1	I_{001}	121.6(3)	C_{35}	C_{34}	N_7	106.4(4)
C_6	C_1	I_{001}	118.0(3)	N_3	C_{16}	C_{17}	107.7(4)
C_6	C_1	C_2	120.0(4)	C_{30}	C_{29}	C_{28}	120.5(5)

Table S5 Bond Angles for **5bb**.

Atom	Atom	Atom	Angle/°	Atom	Atom	Atom	Angle/°
C_{19}	C_{24}	N_5	131.6(4)	N_8	C_{36}	C_{35}	110.2(5)
C_{19}	C_{24}	C_{23}	121.7(4)	C_{30}	C_{31}	C_{32}	119.9(4)
C_{23}	C_{24}	N_5	106.7(4)	C_{31}	C_{30}	C_{29}	120.6(4)
C_{28}	C_{33}	I_{002}	123.6(3)	N_2	C_9	N_1	114.0(4)
C_{28}	C_{33}	C_{32}	120.1(4)	C_{34}	C_{35}	C_{36}	106.3(4)
C_{32}	C_{33}	I_{002}	116.2(3)	F_1	C_{37}	S_1	110.9(3)
N_2	C_5	C_6	109.2(4)	F_1	C_{37}	F_3	107.5(4)
N_2	C_5	C_4	132.2(4)	F_1	C_{37}	F_2	108.4(4)
C_4	C_5	C_6	118.6(4)	F_3	C_{37}	S_1	110.6(3)
C_{14}	C_{15}	I_{001}	118.4(3)	F_2	C_{37}	S_1	112.1(3)
C_{14}	C_{15}	C_{10}	118.6(4)	F_2	C_{37}	F_3	107.3(4)
C_{10}	C_{15}	I_{001}	123.0(3)	N_4	C_{18}	C_{17}	110.0(4)
C_{21}	C_{22}	C_{23}	120.1(4)	F_5	C_{38}	S_2	111.2(3)
C_{24}	C_{19}	I_{002}	118.0(3)	F_5	C_{38}	F_4	107.2(4)
C_{24}	C_{19}	C_{20}	119.7(4)	F_4	C_{38}	S_2	109.2(4)
C_{20}	C_{19}	I_{002}	122.1(3)	F_6	C_{38}	S_2	111.7(4)
C_{19}	C_{20}	C_{21}	118.9(4)	F_6	C_{38}	F_5	109.9(5)
C_{19}	C_{20}	C_{25}	121.6(4)	F_6	C_{38}	F_4	107.6(4)

Table S6 Torsion Angles for **5bb**.

A	B	C	D	Angle/°	A	B	C	D	Angle/°
I_{001}	C_1	C_2	C_3	172.9(3)	C_2	C_1	C_6	C_5	2.9(7)
I_{001}	C_1	C_2	C_7	-6.1(6)	C_{23}	N_6	C_{27}	N_5	-0.2(5)
I_{001}	C_1	C_6	N_1	9.6(7)	C_{23}	C_{24}	C_{19}	I_{002}	-171.8(3)
I_{001}	C_1	C_6	C_5	-169.7(3)	C_{23}	C_{24}	C_{19}	C_{20}	2.4(6)
I_{001}	C_{15}	C_{14}	N_3	-3.1(6)	C_{23}	C_{22}	C_{21}	C_{20}	1.1(7)
I_{001}	C_{15}	C_{14}	C_{13}	177.8(3)	C_{23}	C_{22}	C_{21}	C_{26}	-178.6(4)
I_{001}	C_{15}	C_{10}	N_1	3.0(6)	C_{28}	N_5	C_{24}	C_{19}	11.7(7)
I_{001}	C_{15}	C_{10}	C_{11}	-176.4(3)	C_{28}	N_5	C_{24}	C_{23}	-169.3(4)
I_{002}	N_8	C_{36}	C_{35}	148.3(4)	C_{28}	N_5	C_{27}	N_6	169.9(4)
I_{002}	C_{33}	C_{28}	N_5	-3.1(6)	C_{28}	C_{33}	C_{32}	N_7	177.4(4)
I_{002}	C_{33}	C_{28}	C_{29}	178.7(3)	C_{28}	C_{33}	C_{32}	C_{31}	-1.6(6)
I_{002}	C_{33}	C_{32}	N_7	0.1(5)	C_{28}	C_{29}	C_{30}	C_{31}	-0.5(7)
I_{002}	C_{33}	C_{32}	C_{31}	-178.9(3)	C_{27}	N_5	C_{24}	C_{19}	-177.9(5)
I_{002}	C_{19}	C_{20}	C_{21}	173.9(3)	C_{27}	N_5	C_{24}	C_{23}	1.1(5)
I_{002}	C_{19}	C_{20}	C_{25}	-5.2(6)	C_{27}	N_5	C_{28}	C_{33}	177.8(4)
O_2	S_1	C_{37}	F_1	-59.6(4)	C_{27}	N_5	C_{28}	C_{29}	-4.0(6)
O_2	S_1	C_{37}	F_3	59.5(4)	C_{27}	N_6	C_{23}	C_{24}	1.0(5)
O_2	S_1	C_{37}	F_2	179.2(3)	C_{27}	N_6	C_{23}	C_{22}	-178.1(5)
O_1	S_1	C_{37}	F_1	59.7(4)	C_{21}	C_{22}	C_{23}	N_6	-179.8(4)
O_1	S_1	C_{37}	F_3	178.9(3)	C_{21}	C_{22}	C_{23}	C_{24}	1.2(7)

Table S6 Torsion Angles for **5bb**.

A	B	C	D	Angle/°	A	B	C	D	Angle/°
O_1	S_1	C_{37}	F_2	-61.5(4)	C_6	N_1	C_{10}	C_{15}	-18.2(7)
O_3	S_1	C_{37}	F_1	-178.8(3)	C_6	N_1	C_{10}	C_{11}	161.2(4)
O_3	S_1	C_{37}	F_3	-59.7(4)	C_6	N_1	C_9	N_2	0.5(5)
O_3	S_1	C_{37}	F_2	59.9(4)	C_6	C_1	C_2	C_3	0.6(7)
N_5	C_{24}	C_{19}	I_{002}	7.1(6)	C_6	C_1	C_2	C_7	-178.4(4)
N_5	C_{24}	C_{19}	C_{20}	-178.7(4)	C_6	C_5	C_4	C_3	-0.4(6)
N_5	C_{24}	C_{23}	N_6	-1.4(5)	C_4	C_5	C_6	N_1	177.5(4)
N_5	C_{24}	C_{23}	C_{22}	177.9(4)	C_4	C_5	C_6	C_1	-3.1(7)
N_5	C_{28}	C_{29}	C_{30}	-178.9(4)	C_{14}	N_3	C_{16}	C_{17}	178.0(4)
O_4	S_2	C_{38}	F_5	-54.7(4)	C_{14}	C_{15}	C_{10}	N_1	-176.8(4)
O_4	S_2	C_{38}	F_4	63.4(4)	C_{14}	C_{15}	C_{10}	C_{11}	3.9(6)
O_4	S_2	C_{38}	F_6	-177.8(4)	C_{14}	C_{13}	C_{12}	C_{11}	1.4(7)
O_6	S_2	C_{38}	F_5	64.5(4)	C_{32}	N_7	N_8	I_{002}	19.7(4)
O_6	S_2	C_{38}	F_4	-177.5(3)	C_{32}	N_7	N_8	C_{36}	179.6(4)
O_6	S_2	C_{38}	F_6	-58.6(4)	C_{32}	N_7	C_{34}	C_{35}	179.9(4)
N_4	N_3	C_{14}	C_{15}	-11.9(6)	C_{32}	C_{33}	C_{28}	N_5	179.8(4)
N_4	N_3	C_{14}	C_{13}	167.2(4)	C_{32}	C_{33}	C_{28}	C_{29}	1.6(6)
N_4	N_3	C_{16}	C_{17}	0.6(5)	C_{32}	C_{31}	C_{30}	C_{29}	0.5(7)
N_7	N_8	C_{36}	C_{35}	0.9(6)	C_{10}	N_1	C_6	C_1	11.4(8)
N_7	C_{32}	C_{31}	C_{30}	-178.5(4)	C_{10}	N_1	C_6	C_5	-169.3(4)
N_7	C_{34}	C_{35}	C_{36}	0.0(6)	C_{10}	N_1	C_9	N_2	170.8(4)
O_5	S_2	C_{38}	F_5	-175.5(4)	C_{10}	C_{15}	C_{14}	N_3	176.6(4)
O_5	S_2	C_{38}	F_4	-57.4(4)	C_{10}	C_{15}	C_{14}	C_{13}	-2.4(7)
O_5	S_2	C_{38}	F_6	61.4(4)	C_{10}	C_{11}	C_{12}	C_{13}	0.1(7)
N_3	N_4	C_{18}	C_{17}	0.8(5)	C_{12}	C_{13}	C_{14}	N_3	-179.3(4)
N_8	N_7	C_{32}	C_{33}	-15.8(6)	C_{12}	C_{13}	C_{14}	C_{15}	-0.2(7)
N_8	N_7	C_{32}	C_{31}	163.2(4)	C_{12}	C_{11}	C_{10}	N_1	177.8(4)
N_8	N_7	C_{34}	C_{35}	0.6(5)	C_{12}	C_{11}	C_{10}	C_{15}	-2.8(7)
N_8	C_{36}	C_{35}	C_{34}	-0.6(6)	C_{34}	N_7	N_8	I_{002}	-160.8(3)
N_2	C_5	C_6	N_1	-2.0(5)	C_{34}	N_7	N_8	C_{36}	-0.9(5)
N_2	C_5	C_6	C_1	177.4(4)	C_{34}	N_7	C_{32}	C_{33}	164.9(5)
N_2	C_5	C_4	C_3	179.0(4)	C_{34}	N_7	C_{32}	C_{31}	-16.1(7)
C_1	C_2	C_3	C_4	-4.0(7)	C_{16}	N_3	C_{14}	C_{15}	170.9(5)
C_1	C_2	C_3	C_8	176.1(4)	C_{16}	N_3	C_{14}	C_{13}	-10.0(7)
C_{24}	N_5	C_{28}	C_{33}	-13.7(7)	C_{16}	C_{17}	C_{18}	N_4	-0.4(6)
C_{24}	N_5	C_{28}	C_{29}	164.5(4)	C_{25}	C_{20}	C_{21}	C_{22}	177.5(4)
C_{24}	N_5	C_{27}	N_6	-0.6(5)	C_{25}	C_{20}	C_{21}	C_{26}	-2.9(6)
C_{24}	C_{19}	C_{20}	C_{21}	-0.1(6)	C_7	C_2	C_3	C_4	175.0(4)
C_{24}	C_{19}	C_{20}	C_{25}	-179.1(4)	C_7	C_2	C_3	C_8	-4.9(7)
C_{33}	C_{28}	C_{29}	C_{30}	-0.6(7)	C_9	N_2	C_5	C_6	2.2(5)
C_{33}	C_{32}	C_{31}	C_{30}	0.5(7)	C_9	N_2	C_5	C_4	-177.2(5)
C_5	N_2	C_9	N_1	-1.7(5)	C_9	N_1	C_6	C_1	-178.4(5)
C_5	C_4	C_3	C_2	3.9(7)	C_9	N_1	C_6	C_5	0.9(5)

Table S6 Torsion Angles for **5bb**.

A	B	C	D	Angle/˚	A	B	C	D	Angle/˚
C_5	C_4	C_3	C_8	-176.2(4)	C_9	N_1	C_{10}	C_{15}	173.6(4)
C_{19}	C_{24}	C_{23}	N_6	177.8(4)	C_9	N_1	C_{10}	C_{11}	-7.0(6)
C_{19}	C_{24}	C_{23}	C_{22}	-3.0(6)	C_{18}	N_4	N_3	C_{14}	-178.6(4)
C_{19}	C_{20}	C_{21}	C_{22}	-1.7(6)	C_{18}	N_4	N_3	C_{16}	-0.9(5)
C_{19}	C_{20}	C_{21}	C_{26}	178.0(4)	C_{18}	C_{17}	C_{16}	N_3	-0.1(5)
C_2	C_1	C_6	N_1	-177.9(4)					

Table S7 Hydrogen Atom Coordinates (Å×10^4) and Isotropic Displacement Parameters (Å^2×10^3) for **5bb**.

Atom	*x*	*y*	*z*	U(eq)
H_{22}	9764.84	4559.78	8089.01	22
H_{27}	10027.18	7695.44	9887.29	24
H_{13}	8271.2	4771.8	10295.48	24
H_4	2128.76	8570.96	8047.15	25
H_{11}	6700.87	7281.43	10742.97	24
H_{12}	8332.7	6290.86	11169.19	26
H_{17}	7533.34	1949.27	8182.71	29
H_{34}	4321.43	10310.97	7579.49	29
H_{16}	8557.5	3442.88	9403.11	28
H_{8A}	2721.26	7643.9	6237.59	39
H_{8B}	1391.85	8190.4	6660.1	39
H_{8C}	764.5	7059.05	6153.34	39
H_{25A}	6612.75	5607.02	5714	34
H_{25B}	6283.76	4493.12	5641.33	34
H_{25C}	8139	5035.96	5572.18	34
H_{7A}	3403.2	5256.1	6401.91	40
H_{7B}	3162.64	6071.04	6009.04	40
H_{7C}	1468.69	5431.59	6127.22	40
H_{29}	8616.73	8761.85	9891.58	27
H_{26A}	8968.98	3359.47	6825.09	38
H_{26B}	9097.73	3761.37	6106.69	38
H_{26C}	7198.61	3400.68	6245.01	38
H_{36}	5370.74	8912.19	5512.18	34
H_{31}	6085.85	10320.89	8774.22	30
H_{30}	7340.38	10073.66	9942.18	31
H_9	4750.17	8031.31	10436.67	26
H_{35}	3735	10123.16	6130.08	35
H_{18}	5040.36	2265.11	7247.67	34

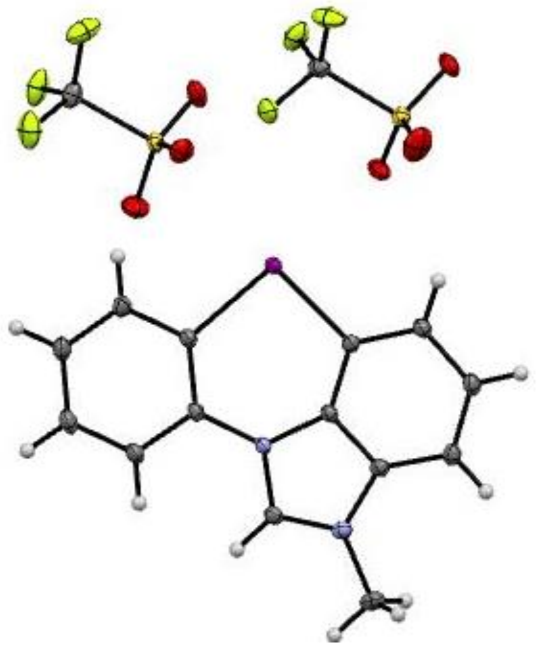

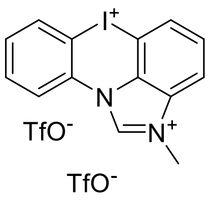

Table S8 Crystal data and structure refinement for **5av**.

Empirical formula	$C_{16}H_{11}F_6IN_2O_6S_2$
Formula weight	632.29
Temperature/K	100.0
Crystal system	monoclinic
Space group	C2/c
a/Å	21.6489(9)
b/Å	12.9181(5)
c/Å	18.2639(12)
α/°	90
β/°	125.0140(10)
γ/°	90
Volume/Å^3	4183.3(4)
Z	8
ρ_{calc}g/cm^3	2.008
μ/mm^{-1}	1.822
F(000)	2464.0
Crystal size/mm^3	0.22 × 0.2 × 0.16
Radiation	MoKα (λ = 0.71073)
2Θ range for data collection/°	5.446 to 66.488
Index ranges	-33 ≤ h ≤ 33, -19 ≤ k ≤ 19, -28 ≤ l ≤ 28
Reflections collected	71202
Independent reflections	8024 [R_{int} = 0.0325, R_{sigma} = 0.0184]
Data/restraints/parameters	8024/0/300
Goodness-of-fit on F^2	1.048
Final R indexes [I>=2σ (I)]	R_1 = 0.0215, wR_2 = 0.0524
Final R indexes [all data]	R_1 = 0.0262, wR_2 = 0.0543
Largest diff. peak/hole / e Å^{-3}	1.15/-0.75

Table S9 Fractional Atomic Coordinates (×10[4]) and Equivalent Isotropic Displacement Parameters (Å[2]×10[3]) for **5av**. U_{eq} is defined as 1/3 of the trace of the orthogonalised U_{IJ} tensor.

Atom	*x*	*y*	*z*	U(eq)
I1	7001.6(2)	1341.5(2)	7715.4(2)	12.42(3)
N1	6129.6(6)	3088.4(8)	6018.0(7)	12.11(19)
N2	5549.1(7)	2975.7(9)	4568.5(8)	15.0(2)
C1	5819.1(8)	3619.9(10)	5249.6(9)	14.5(2)
C2	5674.0(7)	1965.0(10)	4883.9(9)	13.2(2)
C3	5501.6(8)	1016.6(11)	4444.1(9)	16.1(2)
C4	5744.8(8)	144.9(11)	4977.8(9)	16.6(2)
C5	6154.3(8)	197.6(10)	5916.7(9)	15.0(2)
C6	6307.1(7)	1152.0(10)	6329.1(9)	12.5(2)
C7	6056.2(7)	2040.3(10)	5808.0(8)	11.7(2)
C8	6841.3(7)	2958.7(10)	7655.6(9)	12.9(2)
C9	7159.6(8)	3420.4(11)	8487.3(9)	17.0(2)
C10	7072.2(8)	4476.2(11)	8534.2(10)	20.0(3)
C11	6654.6(8)	5058.3(11)	7757.8(10)	19.5(3)
C12	6332.1(8)	4591.9(10)	6930.5(9)	16.1(2)
C13	6435.8(7)	3538.4(10)	6872.0(9)	12.5(2)
C14	5202.8(10)	3290.1(13)	3639.6(10)	25.0(3)
S1	8175.3(2)	3438.9(3)	6872.8(2)	14.99(6)
F1	8085.1(6)	3242.4(8)	5388.9(7)	28.4(2)
F2	7114.0(5)	3962.9(9)	5216.8(6)	27.4(2)
F3	8151.1(6)	4821.1(7)	5809.4(6)	23.89(19)
O1	7909.7(7)	4252.6(9)	7174.7(7)	22.3(2)
O2	8979.9(7)	3383.4(12)	7336.6(9)	34.5(3)
O3	7788.4(7)	2468.0(8)	6706.0(8)	23.4(2)
C15	7865.4(8)	3886.7(11)	5764.0(9)	16.6(2)
S2	6030.9(2)	6752.9(2)	5043.2(2)	14.39(6)
F4	5652.4(6)	6490.4(10)	3413.5(7)	33.4(2)
F5	5012.5(5)	7671.4(9)	3536.8(7)	30.5(2)
F6	6160.3(6)	7967.9(9)	3989.9(8)	33.0(2)
O4	5972.8(7)	7629.4(9)	5483.9(8)	26.4(2)
O5	6790.3(6)	6414.2(9)	5395.2(7)	22.1(2)
O6	5508.8(6)	5917.7(8)	4844.0(8)	23.1(2)
C16	5696.7(8)	7242.2(12)	3934.3(10)	19.9(3)

Table S10 Anisotropic Displacement Parameters (Å[2]×10[3]) for **5av**. The Anisotropic displacement factor exponent takes the form: $-2\pi^2[h^2a^{*2}U_{11}+2hka^*b^*U_{12}+\ldots]$.

Atom	U_{11}	U_{22}	U_{33}	U_{23}	U_{13}	U_{12}
I1	13.58(4)	9.46(4)	12.08(4)	0.62(2)	6.12(3)	0.43(3)
N1	12.4(5)	8.6(4)	13.1(5)	-0.2(3)	6.0(4)	0.7(4)
N2	14.1(5)	14.1(5)	12.9(5)	1.5(4)	5.4(4)	1.4(4)

Table S10 Anisotropic Displacement Parameters ($Å^2 \times 10^3$) for **5av**. The Anisotropic displacement factor exponent takes the form: $-2\pi^2[h^2a^{*2}U_{11}+2hka^*b^*U_{12}+…]$.

Atom	U_{11}	U_{22}	U_{33}	U_{23}	U_{13}	U_{12}
C1	13.7(5)	12.1(5)	15.4(6)	1.6(4)	7.1(5)	1.4(4)
C2	10.9(5)	12.7(5)	13.8(5)	0.3(4)	5.7(4)	0.6(4)
C3	14.0(6)	16.4(6)	14.7(6)	-3.4(5)	6.5(5)	-0.9(5)
C4	16.5(6)	12.9(5)	18.0(6)	-4.1(4)	8.4(5)	-1.8(4)
C5	15.0(5)	10.7(5)	17.5(6)	-1.0(4)	8.3(5)	-0.2(4)
C6	11.0(5)	11.6(5)	12.6(5)	-0.5(4)	5.5(4)	0.1(4)
C7	10.5(5)	10.0(5)	13.2(5)	-0.7(4)	5.9(4)	-0.3(4)
C8	11.5(5)	10.2(5)	15.2(5)	-0.8(4)	6.6(5)	0.0(4)
C9	15.4(6)	16.0(6)	14.7(6)	-2.7(4)	5.8(5)	-0.5(5)
C10	18.9(6)	17.1(6)	19.1(6)	-6.3(5)	8.0(5)	-0.9(5)
C11	19.4(6)	12.3(5)	23.9(7)	-5.2(5)	10.9(6)	-1.8(5)
C12	16.5(6)	10.4(5)	19.0(6)	-0.5(4)	8.8(5)	0.7(4)
C13	11.0(5)	10.8(5)	13.9(5)	-1.9(4)	6.1(4)	-1.5(4)
C14	29.9(8)	22.1(7)	14.1(6)	4.3(5)	7.4(6)	3.7(6)
S1	14.93(14)	12.69(13)	14.99(14)	1.50(11)	7.21(12)	1.05(11)
F1	34.1(5)	31.1(5)	32.6(5)	-16.3(4)	26.4(5)	-12.1(4)
F2	16.3(4)	37.9(5)	19.1(4)	5.6(4)	4.9(4)	-1.3(4)
F3	31.0(5)	19.4(4)	23.8(4)	0.8(3)	17.2(4)	-7.1(4)
O1	36.6(6)	13.6(4)	25.6(5)	-4.3(4)	22.9(5)	-3.8(4)
O2	15.0(5)	46.8(8)	28.8(6)	9.8(6)	5.0(5)	5.2(5)
O3	33.7(6)	10.5(4)	29.9(6)	0.6(4)	20.4(5)	-1.3(4)
C15	15.8(6)	18.3(6)	16.5(6)	-2.1(5)	9.6(5)	-3.3(5)
S2	13.98(14)	11.36(13)	14.61(14)	1.31(10)	6.33(12)	1.37(10)
F4	28.6(5)	49.1(7)	19.2(5)	-7.0(4)	11.9(4)	4.5(5)
F5	18.4(4)	41.0(6)	28.3(5)	17.9(4)	11.3(4)	13.5(4)
F6	29.4(5)	35.5(6)	40.8(6)	14.5(5)	24.2(5)	1.2(4)
O4	33.6(6)	20.8(5)	28.1(6)	-5.3(4)	19.7(5)	0.8(5)
O5	12.7(4)	23.9(5)	19.0(5)	1.0(4)	2.9(4)	4.0(4)
O6	19.7(5)	14.4(5)	29.9(6)	5.3(4)	11.1(4)	-0.8(4)
C16	15.4(6)	24.5(7)	19.4(6)	5.8(5)	9.7(5)	4.9(5)

Table S11 Bond Lengths for **5av**.

Atom	Atom	Length/Å	Atom	Atom	Length/Å
I1	C6	2.0886(13)	C10	C11	1.386(2)
I1	C8	2.1102(13)	C11	C12	1.387(2)
N1	C1	1.3454(17)	C12	C13	1.3935(18)
N1	C7	1.3912(16)	S1	O1	1.4504(11)
N1	C13	1.4225(17)	S1	O2	1.4365(13)
N2	C1	1.3218(18)	S1	O3	1.4404(11)
N2	C2	1.3896(17)	S1	C15	1.8239(15)

Table S11 Bond Lengths for **5av**.

Atom	Atom	Length/Å	Atom	Atom	Length/Å
N2	C14	1.4648(19)	F1	C15	1.3275(17)
C2	C3	1.3933(18)	F2	C15	1.3375(17)
C2	C7	1.3929(18)	F3	C15	1.3370(16)
C3	C4	1.381(2)	S2	O4	1.4360(12)
C4	C5	1.4094(19)	S2	O5	1.4474(11)
C5	C6	1.3824(18)	S2	O6	1.4498(11)
C6	C7	1.3872(17)	S2	C16	1.8285(15)
C8	C9	1.3917(19)	F4	C16	1.3237(19)
C8	C13	1.3921(18)	F5	C16	1.3396(17)
C9	C10	1.386(2)	F6	C16	1.3340(18)

Table S12 Bond Angles for **5av**.

Atom	Atom	Atom	Angle/˚	Atom	Atom	Atom	Angle/˚
C6	I1	C8	94.06(5)	C8	C13	N1	121.99(11)
C1	N1	C7	107.42(11)	C8	C13	C12	118.74(12)
C1	N1	C13	125.08(11)	C12	C13	N1	119.27(12)
C7	N1	C13	127.42(11)	O1	S1	C15	102.64(7)
C1	N2	C2	109.00(11)	O2	S1	O1	115.03(8)
C1	N2	C14	124.86(12)	O2	S1	O3	116.27(8)
C2	N2	C14	126.10(12)	O2	S1	C15	102.69(7)
N2	C1	N1	110.30(11)	O3	S1	O1	113.71(7)
N2	C2	C3	131.54(13)	O3	S1	C15	104.03(7)
N2	C2	C7	106.00(11)	F1	C15	S1	111.20(10)
C7	C2	C3	122.44(12)	F1	C15	F2	107.63(12)
C4	C3	C2	116.27(12)	F1	C15	F3	107.94(11)
C3	C4	C5	122.57(12)	F2	C15	S1	111.58(10)
C6	C5	C4	119.53(12)	F3	C15	S1	110.80(10)
C5	C6	I1	123.08(10)	F3	C15	F2	107.51(12)
C5	C6	C7	119.13(12)	O4	S2	O5	115.59(7)
C7	C6	I1	117.44(9)	O4	S2	O6	114.90(7)
N1	C7	C2	107.25(11)	O4	S2	C16	104.01(7)
C6	C7	N1	132.70(12)	O5	S2	O6	114.19(7)
C6	C7	C2	120.00(12)	O5	S2	C16	102.89(7)
C9	C8	I1	113.83(10)	O6	S2	C16	102.90(7)
C9	C8	C13	121.12(12)	F4	C16	S2	111.21(11)
C13	C8	I1	125.01(9)	F4	C16	F5	108.21(13)
C10	C9	C8	119.30(13)	F4	C16	F6	108.15(13)
C9	C10	C11	120.16(13)	F5	C16	S2	110.80(10)
C10	C11	C12	120.26(13)	F6	C16	S2	110.87(11)
C11	C12	C13	120.36(13)	F6	C16	F5	107.46(13)

Table S13 Torsion Angles for **5av**.

A	B	C	D	Angle/°	A	B	C	D	Angle/°
I1	C6	C7	N1	-5.2(2)	C9	C10	C11	C12	-1.0(2)
I1	C6	C7	C2	171.72(9)	C10	C11	C12	C13	-1.2(2)
I1	C8	C9	C10	-178.02(11)	C11	C12	C13	N1	-177.01(13)
I1	C8	C13	N1	-4.75(18)	C11	C12	C13	C8	2.7(2)
I1	C8	C13	C12	175.59(10)	C13	N1	C1	N2	-176.41(12)
N2	C2	C3	C4	176.78(14)	C13	N1	C7	C2	175.41(12)
N2	C2	C7	N1	1.76(14)	C13	N1	C7	C6	-7.4(2)
N2	C2	C7	C6	-175.85(12)	C13	C8	C9	C10	-0.2(2)
C1	N1	C7	C2	-1.45(14)	C14	N2	C1	N1	-177.18(13)
C1	N1	C7	C6	175.74(14)	C14	N2	C2	C3	-2.0(2)
C1	N1	C13	C8	-170.90(13)	C14	N2	C2	C7	176.26(14)
C1	N1	C13	C12	8.75(19)	O1	S1	C15	F1	177.91(10)
C1	N2	C2	C3	-179.76(14)	O1	S1	C15	F2	-61.91(12)
C1	N2	C2	C7	-1.47(15)	O1	S1	C15	F3	57.85(11)
C2	N2	C1	N1	0.59(16)	O2	S1	C15	F1	58.24(12)
C2	C3	C4	C5	-0.9(2)	O2	S1	C15	F2	178.43(11)
C3	C2	C7	N1	-179.75(12)	O2	S1	C15	F3	-61.81(12)
C3	C2	C7	C6	2.6(2)	O3	S1	C15	F1	-63.35(11)
C3	C4	C5	C6	1.7(2)	O3	S1	C15	F2	56.84(12)
C4	C5	C6	I1	-173.41(10)	O3	S1	C15	F3	176.60(10)
C4	C5	C6	C7	-0.3(2)	O4	S2	C16	F4	-174.35(11)
C5	C6	C7	N1	-178.65(13)	O4	S2	C16	F5	-53.95(13)
C5	C6	C7	C2	-1.75(19)	O4	S2	C16	F6	65.28(12)
C7	N1	C1	N2	0.54(15)	O5	S2	C16	F4	64.74(12)
C7	N1	C13	C8	12.8(2)	O5	S2	C16	F5	-174.86(11)
C7	N1	C13	C12	-167.59(12)	O5	S2	C16	F6	-55.63(12)
C7	C2	C3	C4	-1.3(2)	O6	S2	C16	F4	-54.19(12)
C8	C9	C10	C11	1.7(2)	O6	S2	C16	F5	66.21(12)
C9	C8	C13	N1	177.67(12)	O6	S2	C16	F6	-174.56(10)
C9	C8	C13	C12	-2.0(2)					

Table S14 Hydrogen Atom Coordinates (Å×10^4) and Isotropic Displacement Parameters (Å^2×10^3) for **5av**.

Atom	*x*	*y*	*z*	U(eq)
H1	5797.03	4353.31	5203.25	17
H3	5231.87	972.29	3812.34	19
H4	5631.63	-516.31	4701.35	20
H5	6324.15	-418.37	6263.61	18
H9	7433.81	3016.26	9017.3	20

Table S14 Hydrogen Atom Coordinates (Å×10^4) and Isotropic Displacement Parameters (Å^2×10^3) for **5av**.

**Atom**	***x***	***y***	***z***	**U(eq)**
H10	7298.99	4801.61	9099.69	24
H11	6589.22	5779.5	7792.78	23
H12	6038.8	4992.84	6400.77	19
H14A	5484.54	2985.89	3422.74	38
H14B	5213.11	4046.45	3606.42	38
H14C	4679.2	3048.23	3268.17	38

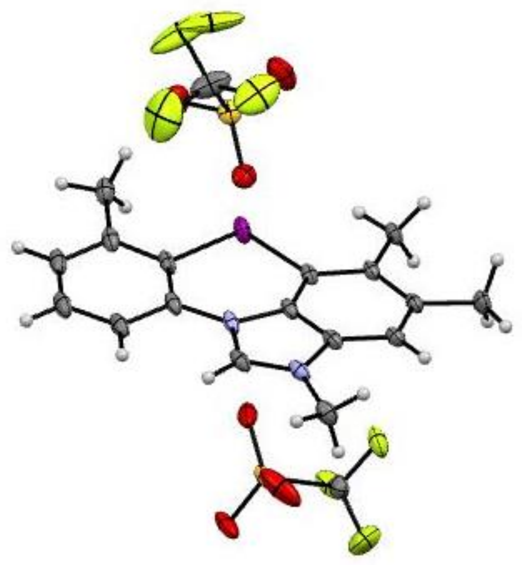

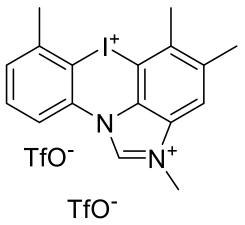

Table S15 Crystal data and structure refinement for **5ax**.

Empirical formula	$C_{37.97}H_{34}F_{12}I_2N_4O_{12}S_4$
Formula weight	1348.37
Temperature/K	100.00
Crystal system	triclinic
Space group	P-1
a/Å	10.2904(7)
b/Å	11.4588(8)
c/Å	11.7246(8)
α/°	63.137(3)
β/°	69.729(3)
γ/°	80.021(3)
Volume/Å^3	1156.71(14)
Z	1
ρ_{calc}g/cm^3	1.936
μ/mm^{-1}	1.654
F(000)	664.0
Crystal size/mm^3	0.2 × 0.2 × 0.2

Radiation	MoKα (λ = 0.71073)
2Θ range for data collection/°	4.09 to 67.13
Index ranges	-15 ≤ h ≤ 15, -17 ≤ k ≤ 17, -18 ≤ l ≤ 18
Reflections collected	96714
Independent reflections	8966 [R_{int} = 0.0417, R_{sigma} = 0.0217]
Data/restraints/parameters	8966/64/339
Goodness-of-fit on F^2	1.049
Final R indexes [I>=2σ (I)]	R_1 = 0.0327, wR_2 = 0.0804
Final R indexes [all data]	R_1 = 0.0423, wR_2 = 0.0863
Largest diff. peak/hole / e Å^{-3}	1.76/-1.59

Table S16 Fractional Atomic Coordinates (×10^4) and Equivalent Isotropic Displacement Parameters (Å^2×10^3) for **5ax**. U_{eq} is defined as 1/3 of the trace of the orthogonalised U_{IJ} tensor.

Atom	*x*	*y*	*z*	U(eq)
I1	7976.1(2)	1419.1(2)	9570.1(2)	21.23(4)
S2	11084.1(6)	377.7(5)	7218.3(5)	23.74(10)
S1	4235.4(5)	5166.5(7)	7647.0(6)	28.37(12)
F3	5868.4(16)	6430.0(17)	7966.0(18)	37.5(3)
F2	3946.5(16)	5850(2)	9565.0(16)	40.0(4)
O4	10197.2(17)	1479.7(17)	6727.4(17)	28.1(3)
N2	7706.6(17)	5064.7(19)	4996.6(17)	21.0(3)
F1	3954(3)	7444.2(18)	7720(3)	62.4(6)
O1	2751.6(16)	5025(2)	8140(2)	36.6(4)
N1	7327.1(17)	3063.0(18)	6562.8(17)	19.2(3)
O2	5012.5(18)	4013(2)	8250(3)	48.2(6)
O3	10393(2)	-826(2)	8186(2)	41.0(4)
O5	12182(2)	642(2)	7565(2)	48.2(5)
C00F	7984.6(18)	3703(2)	6975.3(19)	18.0(3)
C8	8368.4(18)	3333.4(19)	8113.9(18)	17.0(3)
O6	4781(2)	5864(3)	6210(2)	60.1(8)
C00I	8825(2)	5893(2)	6100(2)	20.1(3)
C15	7178(2)	3931(2)	5374(2)	21.9(4)
C11	8220.1(19)	4972(2)	5987.9(19)	19.0(3)
C1	7080.1(19)	892(2)	8491(2)	20.7(4)
C10	9176.3(19)	5516(2)	7258(2)	19.3(3)
C9	8969.0(18)	4213.4(19)	8287.6(19)	16.9(3)
C12	9388(2)	3837(2)	9513(2)	19.8(3)
C6	6707(2)	-419(2)	9146(2)	23.6(4)
C7	7060(2)	-1365(2)	10397(2)	29.0(4)
C3	6124(2)	1311(2)	6749(2)	25.0(4)
C2	6844.0(19)	1758(2)	7285(2)	20.4(3)
C14	7649(2)	6267(2)	3804(2)	26.5(4)
C5	6008(2)	-831(2)	8561(3)	27.6(4)
C16	4518(2)	6280(2)	8255(2)	25.2(4)

Table S16 Fractional Atomic Coordinates (×10[4]) and Equivalent Isotropic Displacement Parameters (Å[2]×10[3]) for **5ax**. U_{eq} is defined as 1/3 of the trace of the orthogonalised U_{IJ} tensor.

Atom	*x*	*y*	*z*	U(eq)
C4	5707(2)	28(2)	7394(3)	28.8(5)
C13	9760(2)	6515(2)	7446(2)	26.8(4)
F7	12699(3)	1175(2)	4750(2)	71.7(7)
F4	11207(4)	-139(3)	5229(3)	89.6(9)
F5	12653(13)	-1018(11)	5995(10)	103(4)
C17	12048(4)	111(3)	5716(3)	46.1(9)
F6	13146(10)	-677(11)	5920(11)	97(4)

Table S17 Anisotropic Displacement Parameters (Å[2]×10[3]) for **5ax**. The Anisotropic displacement factor exponent takes the form: $-2\pi^2[h^2a^{*2}U_{11}+2hka^*b^*U_{12}+\ldots]$.

Atom	U_{11}	U_{22}	U_{33}	U_{23}	U_{13}	U_{12}
I1	20.77(6)	28.82(7)	16.58(6)	-12.72(5)	1.13(4)	-11.92(5)
S2	24.8(2)	28.4(2)	15.93(19)	-9.86(18)	-1.69(17)	-3.63(18)
S1	13.13(19)	49.0(3)	41.2(3)	-37.1(3)	-4.07(19)	-1.7(2)
F3	27.3(7)	47.8(9)	48.5(9)	-29.7(8)	-5.4(6)	-14.2(6)
F2	25.7(7)	75.2(12)	35.2(8)	-38.7(8)	-9.4(6)	4.4(7)
O4	22.4(7)	28.7(8)	25.4(7)	-11.0(6)	2.1(6)	-2.8(6)
N2	16.3(7)	32.7(9)	20.2(7)	-15.9(7)	-5.8(6)	-1.7(6)
F1	69.7(14)	28.4(9)	92.7(17)	-23.7(10)	-38.9(13)	14.1(9)
O1	13.3(6)	62.4(12)	54.2(11)	-45.3(10)	-4.8(7)	-1.8(7)
N1	14.5(6)	29.6(8)	20.1(7)	-18.2(6)	0.3(5)	-6.5(6)
O2	18.4(8)	37.1(10)	101.2(19)	-47.2(12)	-8.2(10)	1.0(7)
O3	47.3(11)	32.4(9)	28.7(9)	-0.3(7)	-8.2(8)	-9.0(8)
O5	44.2(12)	60.6(14)	40.4(11)	-12.2(10)	-20.4(9)	-13.9(10)
C00F	11.2(7)	27.9(9)	20.6(8)	-16.7(7)	-1.5(6)	-2.8(6)
C8	13.1(7)	24.1(8)	17.2(7)	-13.4(7)	-0.1(6)	-4.7(6)
O6	26.4(9)	138(3)	37.8(11)	-56.0(15)	-4.8(8)	-9.6(12)
C00I	15.7(8)	25.9(9)	22.1(8)	-12.1(7)	-6.6(6)	-1.6(6)
C15	15.1(8)	35.8(10)	22.6(9)	-19.5(8)	-3.7(6)	-3.3(7)
C11	12.8(7)	29.8(9)	19.7(8)	-15.3(7)	-3.9(6)	-1.6(6)
C1	14.5(7)	31.6(10)	23.4(8)	-20.1(8)	0.7(6)	-7.4(7)
C10	14.9(7)	24.8(9)	23.7(8)	-14.0(7)	-6.2(6)	-2.3(6)
C9	11.7(7)	24.5(8)	19.6(8)	-14.5(7)	-2.0(6)	-3.1(6)
C12	18.8(8)	26.2(9)	20.3(8)	-13.3(7)	-6.0(6)	-4.6(7)
C6	16.9(8)	31.1(10)	28.3(9)	-21.6(8)	1.8(7)	-6.6(7)
C7	27.8(10)	29.5(10)	30.8(11)	-15.6(9)	-1.3(8)	-10.9(8)
C3	18.1(8)	38.2(11)	32.2(10)	-27.3(9)	-5.0(7)	-2.9(8)
C2	13.9(7)	30.6(10)	24.6(9)	-20.6(8)	-0.7(6)	-4.4(7)
C14	25.4(10)	34.7(11)	22.6(9)	-12.7(8)	-8.2(8)	-5.4(8)
C5	20.7(9)	34.0(11)	37.8(11)	-27.1(10)	-1.0(8)	-6.4(8)

Table S17 Anisotropic Displacement Parameters (Å^2×10^3) for **5ax**. The Anisotropic displacement factor exponent takes the form: -2π^2[h^{2}a*^{2}U$_{11}$+2hka*b*U$_{12}$+…].

Atom	U_{11}	U_{22}	U_{33}	U_{23}	U_{13}	U_{12}
C16	25.3(10)	27.6(10)	30.8(10)	-18.1(8)	-10.3(8)	0.2(8)
C4	21.7(9)	39.0(12)	40.7(12)	-30.9(10)	-5.3(8)	-4.9(8)
C13	30.1(10)	25.2(10)	33.9(11)	-13.7(8)	-16.4(9)	-4.6(8)
F7	75.9(15)	64.9(13)	32.6(9)	-15.1(9)	21.8(10)	2.6(11)
F4	142(3)	96(2)	60.5(15)	-55.1(15)	-30.4(17)	-12.2(18)
F5	193(11)	65(4)	33(3)	-34(3)	-35(6)	75(6)
C17	64(2)	40.8(15)	21.3(11)	-13.3(10)	-10.2(11)	22.2(13)
F6	88(5)	108(8)	40(3)	-18(4)	-14(3)	80(5)

Table S18 Bond Lengths for **5ax**.

Atom	Atom	Length/Å	Atom	Atom	Length/Å
I1	C8	2.082(2)	C00F	C8	1.383(3)
I1	C1	2.1226(18)	C00F	C11	1.385(3)
S2	O4	1.4376(18)	C8	C9	1.389(2)
S2	O3	1.436(2)	C00I	C11	1.388(3)
S2	O5	1.442(2)	C00I	C10	1.384(3)
S2	C17	1.830(3)	C1	C6	1.396(3)
S1	O1	1.4426(17)	C1	C2	1.390(3)
S1	O2	1.438(2)	C10	C9	1.426(3)
S1	O6	1.441(2)	C10	C13	1.510(3)
S1	C16	1.820(2)	C9	C12	1.497(3)
F3	C16	1.334(3)	C6	C7	1.501(3)
F2	C16	1.318(3)	C6	C5	1.401(3)
N2	C15	1.319(3)	C3	C2	1.396(3)
N2	C11	1.391(2)	C3	C4	1.381(3)
N2	C14	1.464(3)	C5	C4	1.382(4)
F1	C16	1.318(3)	F7	C17	1.315(4)
N1	C00F	1.396(2)	F4	C17	1.316(5)
N1	C15	1.341(3)	F5	C17	1.284(10)
N1	C2	1.422(3)	C17	F6	1.337(8)

Table S19 Bond Angles for **5ax**.

Atom	Atom	Atom	Angle/°	Atom	Atom	Atom	Angle/°
C8	I1	C1	94.54(8)	C2	C1	C6	123.03(18)
O4	S2	O5	115.25(13)	C00I	C10	C9	121.40(17)
O4	S2	C17	102.70(11)	C00I	C10	C13	119.16(19)
O3	S2	O4	115.65(12)	C9	C10	C13	119.42(17)
O3	S2	O5	113.38(13)	C8	C9	C10	118.06(17)

Table S19 Bond Angles for **5ax**.

Atom	Atom	Atom	Angle/°	Atom	Atom	Atom	Angle/°
O3	S2	C17	105.80(15)	C8	C9	C12	122.13(18)
O5	S2	C17	101.86(17)	C10	C9	C12	119.81(16)
O1	S1	C16	103.69(10)	C1	C6	C7	122.33(18)
O2	S1	O1	115.16(13)	C1	C6	C5	116.8(2)
O2	S1	O6	115.26(15)	C5	C6	C7	120.9(2)
O2	S1	C16	103.03(12)	C4	C3	C2	120.0(2)
O6	S1	O1	114.17(13)	C1	C2	N1	122.96(17)
O6	S1	C16	103.22(14)	C1	C2	C3	118.2(2)
C15	N2	C11	108.63(18)	C3	C2	N1	118.8(2)
C15	N2	C14	126.26(17)	C4	C5	C6	121.1(2)
C11	N2	C14	124.85(18)	F3	C16	S1	110.91(15)
C00F	N1	C2	126.96(17)	F2	C16	S1	111.46(17)
C15	N1	C00F	107.04(17)	F2	C16	F3	108.39(18)
C15	N1	C2	125.94(16)	F1	C16	S1	111.70(17)
C8	C00F	N1	133.57(19)	F1	C16	F3	108.1(2)
C8	C00F	C11	119.10(17)	F1	C16	F2	106.1(2)
C11	C00F	N1	107.30(17)	C3	C4	C5	120.7(2)
C00F	C8	I1	117.41(13)	F7	C17	S2	110.7(2)
C00F	C8	C9	121.19(18)	F7	C17	F4	105.4(3)
C9	C8	I1	121.39(14)	F7	C17	F6	99.0(6)
C10	C00I	C11	117.85(19)	F4	C17	S2	111.2(3)
N2	C15	N1	110.80(17)	F4	C17	F6	119.6(6)
C00F	C11	N2	106.23(17)	F5	C17	S2	112.5(5)
C00F	C11	C00I	122.37(18)	F5	C17	F7	121.2(6)
C00I	C11	N2	131.4(2)	F5	C17	F4	94.1(6)
C6	C1	I1	112.73(15)	F6	C17	S2	110.1(5)
C2	C1	I1	124.21(14)				

Table S20 Torsion Angles for **5ax**.

A	B	C	D	Angle/°	A	B	C	D	Angle/°
I1	C8	C9	C10	-178.62(13)	O6	S1	C16	F2	173.60(17)
I1	C8	C9	C12	1.0(2)	O6	S1	C16	F1	55.1(2)
I1	C1	C6	C7	-6.3(2)	C00I	C10	C9	C8	-1.6(3)
I1	C1	C6	C5	175.13(15)	C00I	C10	C9	C12	178.82(18)
I1	C1	C2	N1	7.5(3)	C15	N2	C11	C00F	0.9(2)
I1	C1	C2	C3	-173.81(14)	C15	N2	C11	C00I	-177.3(2)
O4	S2	C17	F7	56.9(3)	C15	N1	C00F	C8	177.7(2)
O4	S2	C17	F4	-59.8(3)	C15	N1	C00F	C11	-0.2(2)
O4	S2	C17	F5	-164.0(7)	C15	N1	C2	C1	177.51(19)
O4	S2	C17	F6	165.3(7)	C15	N1	C2	C3	-1.1(3)
O1	S1	C16	F3	175.11(18)	C11	N2	C15	N1	-1.1(2)

Table S20 Torsion Angles for **5ax**.

A	B	C	D	Angle/°	A	B	C	D	Angle/°
O1	S1	C16	F2	54.25(19)	C11	C00F	C8	I1	179.56(13)
O1	S1	C16	F1	-64.2(2)	C11	C00F	C8	C9	0.7(3)
N1	C00F	C8	I1	1.8(3)	C11	C00I	C10	C9	2.0(3)
N1	C00F	C8	C9	-177.01(19)	C11	C00I	C10	C13	-176.54(18)
N1	C00F	C11	N2	-0.4(2)	C1	C6	C5	C4	-0.1(3)
N1	C00F	C11	C00I	178.00(17)	C10	C00I	C11	N2	176.89(19)
O2	S1	C16	F3	54.7(2)	C10	C00I	C11	C00F	-1.1(3)
O2	S1	C16	F2	-66.12(19)	C6	C1	C2	N1	-174.59(18)
O2	S1	C16	F1	175.4(2)	C6	C1	C2	C3	4.1(3)
O3	S2	C17	F7	178.6(2)	C6	C5	C4	C3	1.9(3)
O3	S2	C17	F4	61.8(3)	C7	C6	C5	C4	-178.6(2)
O3	S2	C17	F5	-42.3(8)	C2	N1	C00F	C8	0.4(3)
O3	S2	C17	F6	-73.0(7)	C2	N1	C00F	C11	-177.55(17)
O5	S2	C17	F7	-62.7(3)	C2	N1	C15	N2	178.18(17)
O5	S2	C17	F4	-179.5(2)	C2	C1	C6	C7	175.56(19)
O5	S2	C17	F5	76.4(7)	C2	C1	C6	C5	-3.0(3)
O5	S2	C17	F6	45.7(7)	C2	C3	C4	C5	-0.8(3)
C00F	N1	C15	N2	0.8(2)	C14	N2	C15	N1	-175.47(18)
C00F	N1	C2	C1	-5.7(3)	C14	N2	C11	C00F	175.38(18)
C00F	N1	C2	C3	175.70(18)	C14	N2	C11	C00I	-2.8(3)
C00F	C8	C9	C10	0.2(3)	C4	C3	C2	N1	176.60(18)
C00F	C8	C9	C12	179.78(17)	C4	C3	C2	C1	-2.1(3)
C8	C00F	C11	N2	-178.67(16)	C13	C10	C9	C8	176.95(18)
C8	C00F	C11	C00I	-0.3(3)	C13	C10	C9	C12	-2.7(3)
O6	S1	C16	F3	-65.5(2)					

Table S21 Hydrogen Atom Coordinates (Å×10^4) and Isotropic Displacement Parameters (Å^2×10^3) for **5ax**.

Atom	*x*	*y*	*z*	U(eq)
H00I	8991.39	6754.05	5404.72	24
H15	6748.68	3753.19	4871.76	26
H12A	8844.47	4354.51	9999.77	30
H12B	10375.65	4003.89	9246.53	30
H12C	9218.55	2904.45	10095.78	30
H7A	8068.63	-1459.96	10197.08	44
H7B	6662.33	-2217.09	10722.05	44
H7C	6677.79	-1033.82	11088.14	44
H3	5920.79	1890.54	5941.55	30
H14A	7463.88	6049.56	3157.7	40
H14B	8537.25	6710.31	3393.83	40
H14C	6907.35	6845.36	4063.8	40

Table S21 Hydrogen Atom Coordinates (Å×10^4) and Isotropic Displacement Parameters (Å^2×10^3) for **5ax**.

**Atom**	***x***	***y***	***z***	**U(eq)**
H5	5736.09	-1716.82	8974.02	33
H4	5207.26	-267.15	7030.03	35
H13A	10670.82	6210.64	7575.22	40
H13B	9133.38	6632.69	8235.84	40
H13C	9854.9	7351.05	6648.95	40

Table S22 Atomic Occupancy for **5ax**.

**Atom**	***Occupancy***	**Atom**	***Occupancy***	**Atom**	***Occupancy***
F5	0.5	C17	0.983(8)	F6	0.5
